# Multiple independent evolutionary solutions to core histone gene regulation

**DOI:** 10.1186/gb-2006-7-12-r122

**Published:** 2006-12-21

**Authors:** Leonardo Mariño-Ramírez, I King Jordan, David Landsman

**Affiliations:** 1 Computational Biology Branch, National Center for Biotechnology Information, National Institutes of Health, 8600 Rockville Pike, Bethesda, Maryland 20894-6075, USA; 2 School of Biology, Georgia Institute of Technology, 310 Ferst Drive, Atlanta, Georgia 30332-0230, USA

## Abstract

**Background:**

Core histone genes are periodically expressed along the cell cycle and peak during S phase. Core histone gene expression is deeply evolutionarily conserved from the yeast *Saccharomyces cerevisiae *to human.

**Results:**

We evaluated the evolutionary dynamics of the specific regulatory mechanisms that give rise to the conserved histone regulatory phenotype. In contrast to the conservation of core histone gene expression patterns, the core histone regulatory machinery is highly divergent between species. There has been substantial evolutionary turnover of cis-regulatory sequence motifs along with the transcription factors that bind them. The regulatory mechanisms employed by members of the four core histone families are more similar within species than within gene families. The presence of species-specific histone regulatory mechanisms is opposite to what is seen at the protein sequence level. Core histone proteins are more similar within families, irrespective of their species of origin, than between families, which is consistent with the shared common ancestry of the members of individual histone families. Structure and sequence comparisons between histone families reveal that H2A and H2B form one related group whereas H3 and H4 form a distinct group, which is consistent with the nucleosome assembly dynamics.

**Conclusion:**

The dissonance between the evolutionary conservation of the core histone gene regulatory phenotypes and the divergence of their regulatory mechanisms indicates a highly dynamic mode of regulatory evolution. This distinct mode of regulatory evolution is probably facilitated by a solution space for promoter sequences, in terms of functionally viable cis-regulatory sites, that is substantially greater than that of protein sequences.

## Abstract 2

An analysis of the evolutionary dynamics of the regulatory mechanisms that give rise to the conserved histone regulatory phenotype indicates a substantial evolutionary turnover of cis-regulatory sequence motifs along with the transcription factors that bind them

## Background

Core histone genes encode four families of proteins that package DNA into the nucleosome, which is the basic structural unit of eukaryotic chromosomes [1]. The four core histones are H2A, H2B, H3 and H4, and each nucleosome consists of 146 base-pairs (bp) of DNA wrapped around an octameric core containing two copies of each histone protein. Comparative studies of core histones have revealed that their sequences are among the most evolutionary conserved of all eukaryotic proteins [2]. For instance, the human H4 protein (NP_003539) is 92% identical to its yeast *Saccharomyces cerevisiae *ortholog (NP_014368) [3]. The high levels of core histone sequence conservation are thought to be due to severe structural constraints imposed by their assembly into the histone octamer [4] as well as the similar functional constraints across species associated with the compact binding of DNA [5].

Most of the packaging of genomic DNA by core histones occurs primarily during the S phase of the cell cycle, when DNA is being actively replicated; stoichiometrically appropriate levels of histone proteins are required to bind DNA immediately following replication [6]. As such, the expression of core histone genes is tightly regulated and peaks sharply during S phase [7]. Much like the histone sequences, this histone gene expression pattern is highly conserved among eukaryotes ranging from human to the yeasts *Saccharomyces cerevisiae *and *Schizosaccharomyces pombe *[8-13].

**Figure 1:**
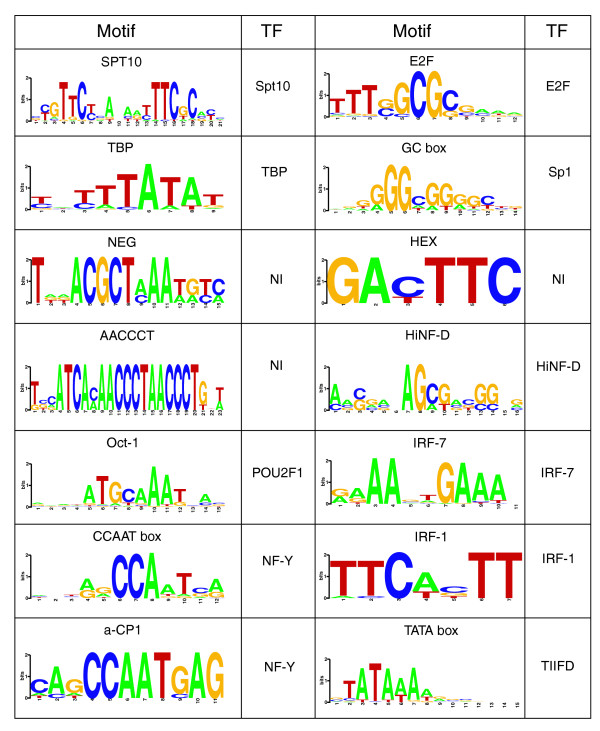
Core histone gene cis-regulatory sequence motifs and transcription factors. Experimentally verified cis-regulatory motifs and their transcription factors were taken from the literature as described in the Introduction section (see text). Sequence logos for the cis-motifs show information content (conservation) per position. Unidentified transcription factors are indicated by NI. TF, transcription factor.

The mechanisms that underlie the cell cycle specific regulation of core histone genes have been intensively studied [6,7]. Although most of this work has focused on the regulation of transcription via the interaction of cis-regulatory elements and transcription factors, a number of studies have also addressed the role of post-transcriptional regulation of core histone synthesis. Here, we focus exclusively on the regulation of core histone gene expression at the transcriptional level. Numerous studies have characterized core histone cis-regulatory sites and their cognate transcription factors [7,14-23]. Sequence logos representing 14 experimentally verified cis-regulatory motifs, along with the names of the transcription factors that bind them, are shown in Figure 1.

The studies that resulted in the characterization of these motifs and transcription factors have led to the elucidation of core histone gene regulation in model experimental systems such as *S. cerevisiae*. For example, the yeast transcription factor Spt10p was recently demonstrated to activate core histone gene expression [16]. Interestingly, the *SPT10 *gene was originally identified as a suppressor of Ty insertion mutations [24,25] and as a global regulator of core promoter activity [26]. However, despite the fact that Spt10p affects the expression of hundreds of yeast genes, it specifically binds cis-regulatory sequences, referred to as upstream activating elements, which are found only in core histone gene promoters. Thus, the global regulatory properties of Spt10p are based solely on changes in levels of core histone gene expression. In support of this model of histone gene regulation, the DNA-binding domain of Spt10p was recently characterized and shown to mediate sequence-specific interaction with the core histone gene upstream activating element [27]. There are a number of such examples, from *S. cerevisiae *and other model systems, of efforts to characterize experimentally the mechanisms of core histone gene regulation. In addition, efforts are underway to investigate core histone promoters among different species computationally [28].

Despite the substantial body of knowledge on the regulation of core histone genes, little is known about the evolutionary dynamics that have given rise to these regulatory mechanisms. We present here an evolutionary analysis of core histone gene regulatory mechanisms. The emphasis of this work is placed on understanding the evolution of cis-regulatory sites along with their cognate transcription factors. We analyzed the phyletic distributions of 14 experimentally verified core histone cis-regulatory elements among 24 crown group eukaryotes. The evolution of core histone gene cis-regulatory sites and transcription factors is considered in light of core histone protein sequence and structure evolution. Despite the highly conserved core histone sequences and expression patterns, the mechanisms of histone gene regulation were found to be highly divergent and lineage specific. The implications of this dissonance with respect to the evolution of gene regulatory systems are explored.

## Results and discussion

### Gene expression patterns

**Figure 2:**
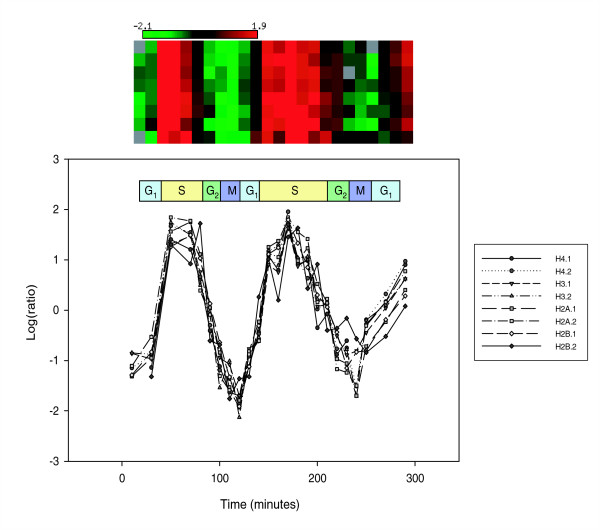
Cell cycle (S phase) specific expression patterns of core histone genes. A cluster of eight core histone genes and their relative expression levels are plotted along the progression time of the cell cycle for the yeast *S. cerevisiae*.

The expression of core histone genes is tightly regulated during the cell cycle and peaks specifically during S phase, concomitant with DNA replication (Figure 2). This is thought to be due to the requirement for histone proteins to bind DNA immediately after its synthesis. A number of recent studies have revealed the extent to which this S phase specific pattern of core histone gene expression is conserved among eukaryotic species; the histone expression pattern has been demonstrated for human core histone genes as well as for histones from *S. cerevisiae *and *S. pombe *[8-13]. This highly conserved regulatory phenotype (the expression pattern) is consistent with the deep conservation of histone protein sequences and further underscores the strong functional (selective) constraint that histone genes are subject to. Considering the highly conserved regulatory phenotype of core histone genes, it would seem to follow that their regulatory mechanisms are similarly conserved.

### Lineage-specific cis-regulatory mechanisms

**Figure 3:**
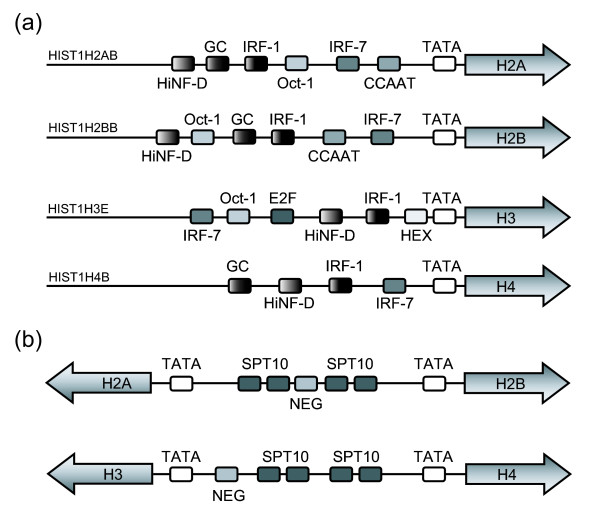
Schema for core histone gene promoters. **(a) **Four different human core histone gene promoters are shown along with the relative locations of predicted cis-binding motifs. Official gene names are indicated for each promoter. These are examples of genes that are not divergently transcribed because a pair of divergently transcribed genes share identical motifs. **(b) **Yeast (*S. cerevisiae*) bidirectional core histone promoters and cis-binding motifs. The promoter sequences and, accordingly, the location/presence of the cis-motifs of individual members of each family may vary for each gene. Not drawn to scale.

Contrary to the expectation that core histone genes would have conserved regulatory mechanisms across species, the best studied core histone genes - namely human and *S. cerevisiae *(yeast) - have different promoter architectures; in fact, they are regulated quite differently [7]. The human and yeast core histone promoters, many of which are bidirectional, are illustrated in Figure 3. Human core histone gene promoters contain more known cis-regulatory binding sites, relative to yeast promoters, which is consistent with the involvement of more transcription factors and the greater complexity of human histone gene regulation. Out of the 14 experimentally characterized cis-regulatory sites that are known to be involved in histone gene regulation in the two species, only one site, the TBP/TATA box, is shared between the two species (Table 1). Furthermore, the phyletic distributions (the presence/absence among species) of the trans-regulatory binding proteins that interact with these sites tend to be lineage specific (Table 2).

In order to evaluate the evolution of core histone promoter cis-regulatory sites in more detail, the phyletic distribution of all 14 experimentally characterized DNA binding motifs among 24 crown group eukaryotic species was assessed. To do this, position frequency matrices (PFMs) of the cis-regulatory motifs (Figure 1) were taken from the TRANSFAC database [29] or were generated from the binding site alignments reported in the original citation. Intergenic promoter regions of core histones (H2A, H2B, H3, and H4) for all 24 species were then searched for the presence of the 14 cis-regulatory motifs using the program CLOVER [30]. CLOVER uses the cis-regulatory site PFMs to evaluate the promoter sequences for statistically significant over- or under-representation of motif elements. For any given promoter sequence (*P*_*i*_), CLOVER assigns a numerical value (raw score) to each cis motif (*j*) indicating its over- or under-representation in that sequence. The distribution of cis-regulatory motifs in that promoter is then represented as a vector, *P*_*i *_= (*P*_*i*1_, *P*_*i*2 _... *P*_*i*14_], of sequence- and motif-specific CLOVER scores (*P*_*ij*_). The CLOVER-generated vectors were then compared using the Pearson correlation coefficient (*r*). High *r *values would thus represent two promoter sequences with similar cis-regulatory binding sites. The *r *values were transformed into pair-wise promoter distances using the following formula: *d *= 1 - (*r *+ 1)/2.

**Figure 4:**
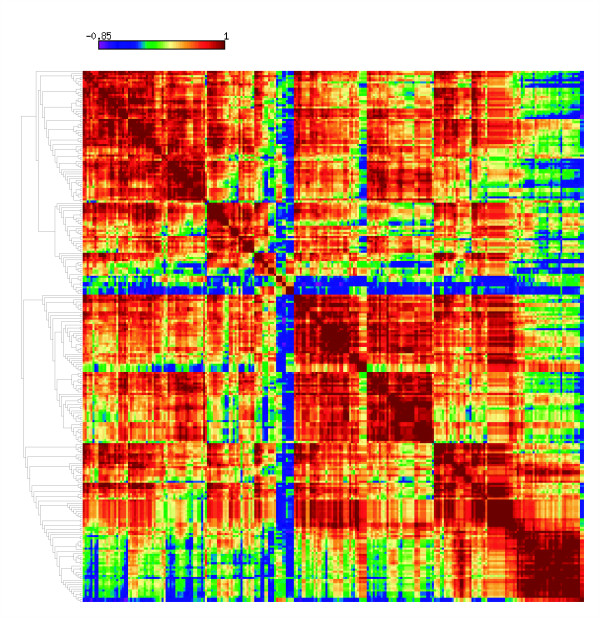
Relationships among core histone gene promoter sequences. Promoter sequences are related by comparisons of cis-regulatory motif vectors, as described in the text. Individual promoters are ordered by similarity along each axis. Pair-wise correlations between promoter-specific vectors are color coded according to the scale bar shown. The block color structure along the diagonal reveals clusters of related promoter sequences.

A total of 254 core histone promoter sequences were compared in this way, resulting in a matrix of 32,131 pair-wise distances. This distance matrix was evaluated using a fast implementation of the neighbor-joining algorithm [31,32] to determine the evolutionary relationships, based on cis-regulatory binding sites, among the core histone promoter sequences (Figure 4). Surprisingly, when histone promoter sequences are related in this way, they tend to form clusters that are relatively lineage specific with respect to the species from which they are derived rather than their family of origin. For instance, there are fairly well defined clusters of histone promoters that are fungi specific and others that are metazoan specific (see red blocks in Figure 4). Importantly, these distinct clusters contain promoters from all four histone gene families. In general, histone promoter sequences from different families are completely intermixed on the tree (they do not tend to group into gene family specific clusters). This suggests that some core histone promoter regions may be evolving in concert within evolutionary lineages, perhaps due to similar lineage-specific regulatory constraints.

**Figure 5:**
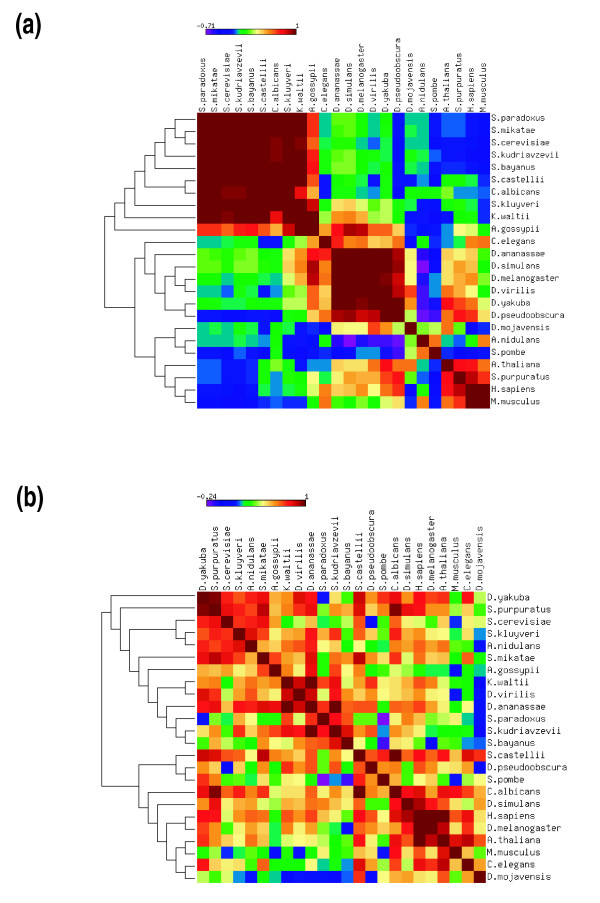
Relationships among species-specific cis-regulatory motif sets. **(a) **Species are related by comparisons of cis-regulatory motif vectors as described in the text. Individual sets of promoters are grouped by species, which are then ordered by similarity along each axis. Pair-wise correlations between species vectors are color coded according to the scale bar shown. **(b) **Randomized promoter sets preserving both mono- and dinucleotide sequence composition is shown for comparison.

The lineage-specific nature of core histone promoter sequence evolution was further explored by generating a species distance matrix analogous to the sequence distance matrix described above. For the species distance matrix, CLOVER scores were calculated for sets of all promoter sequences from individual species. Species-specific CLOVER vectors calculated in this way were compared using *r *value distances, and the resulting distance matrices were used to compute a neighbor joining tree (Figure 5a). As a control, the same comparison was done using promoter sequences that were randomly permuted with preservation of their mono- and dinucleotide frequencies (Figure 5b). Although the topology of the control tree shows no relationship to the species phylogeny, the topology of the tree generated from the observed data is in general agreement with the species phylogeny and thus underscores the within-species coherence of the core histone promoter cis-regulatory motifs. There are, however, some interesting exceptions to this trend. For example, *S. pombe *and *Aspergillus nidulans *are found in a cluster that includes *Drosophila mojavensis*; in addtition, *Arabidopsis thaliana *is nested close to vertebrates as opposed to being an outgroup to the entire ensemble, as would be expected.

### Motif evolutionary dynamics

**Figure 6:**
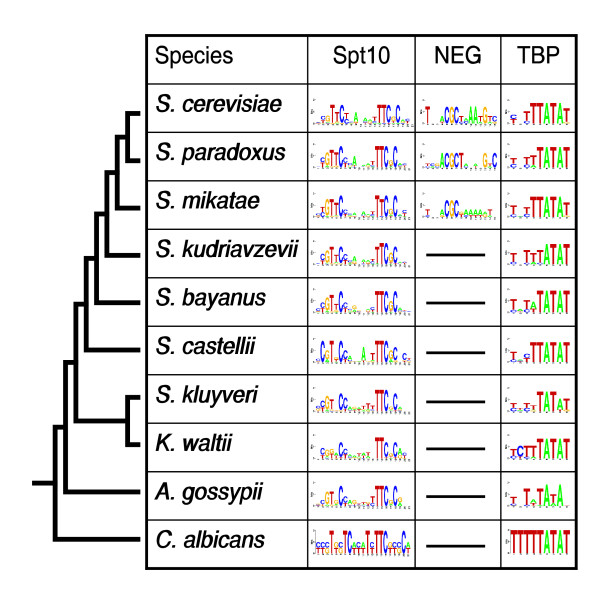
Distribution of cis-regulatory motifs among Saccharomycetales. Species are ordered according to their taxonomic relationships and presence/absence of three motifs is shown, along with their sequence logos.

Further examination of the cis-regulatory motif distribution within the yeast group of species (order Saccharomycetales) shows that different combinations of motifs have distinct evolutionary trajectories, suggesting lineage-specific mechanisms of regulation (Figure 6). For instance, Spt10p and TBP combine to regulate core histones among all Saccharomycetales species evaluated here, whereas the NEG element exerts its negative regulatory effects exclusively among *S. cerevisiae *and its two closest relatives. Furthermore, the position-specific sequence conservation of cis motifs is coherent within species but divergent between species. The information content along positions of the motif sequences changes slightly between lineages, and visual inspection of these changes suggest that they are not always in accordance with the phylogenetic relationships among species (Figure 6).

**Figure 7:**
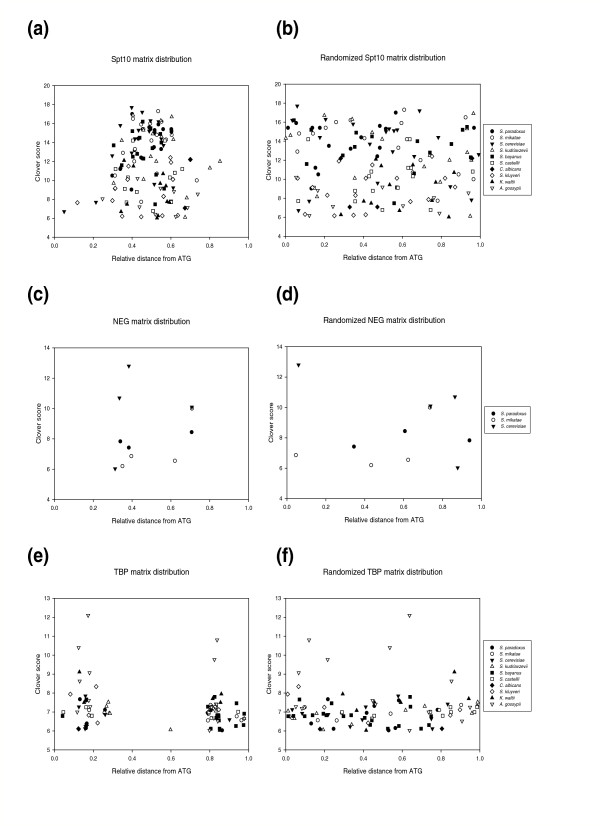
Relative positions of cis-regulatory motifs among Sacchromycetales core histone gene promoters. The relative location of each motif is shown along with its raw CLOVER score; only motifs with scores ≥6 are shown. Relative positions are shown for **(a) **Spt10, **(c) **NEG, and **(e) **TBP. **(b, d, f) **Randomized distributions of motif positions are shown for comparison.

The position of cis-regulatory motifs in the proximal promoter sequences is also critical to histone gene regulation as demonstrated by the conserved relative positions of the motifs in particular species contexts (Figure 7). Spt10p has four experimentally characterized binding sites for each bidirectional promoter in *S. cerevisiae *(Figure 3). Accordingly, when the relative position of Spt10p cognate sequence motifs are evaluated among all species where they are present, they exhibit a marked clustering in the center of the promoter regions (compare Figure 7 panels a and b). On the other hand, NEG and TBP are excluded from the centers of the core histone promoters and tend to map closer to the translational start sites (Figure 7c-f).

### Sequence and structure evolution

The lineage-specific pattern of core histone promoter evolution revealed by the comparative analysis of cis-regulatory motif sequences stands in contrast to the evolution of core histone protein sequences and structures. There are four families of core histone proteins, namely H2A, H2B, H3 and H4, and these families are present in all eukaryotes, indicating that they probably evolved via three ancient gene duplication events that preceded the diversification of the eukaryotic lineage. Given this evolutionary scenario, it can be expected that all protein sequences (structures) of a given family will be more closely related to one another, regardless of the species from which they are derived, than they are to members of other families. Straightforward sequence comparison methods, such as BLASTP [33], bear this expectation out (data not shown). In fact, although sequences within families are highly conserved, it is not possible to identify members of different families using pair-wise BLASTP comparisons. On the other hand, despite its low sequence similarity among core histones, the histone fold domain (HFD) is present in all four core histones [34,35].

**Figure 8:**
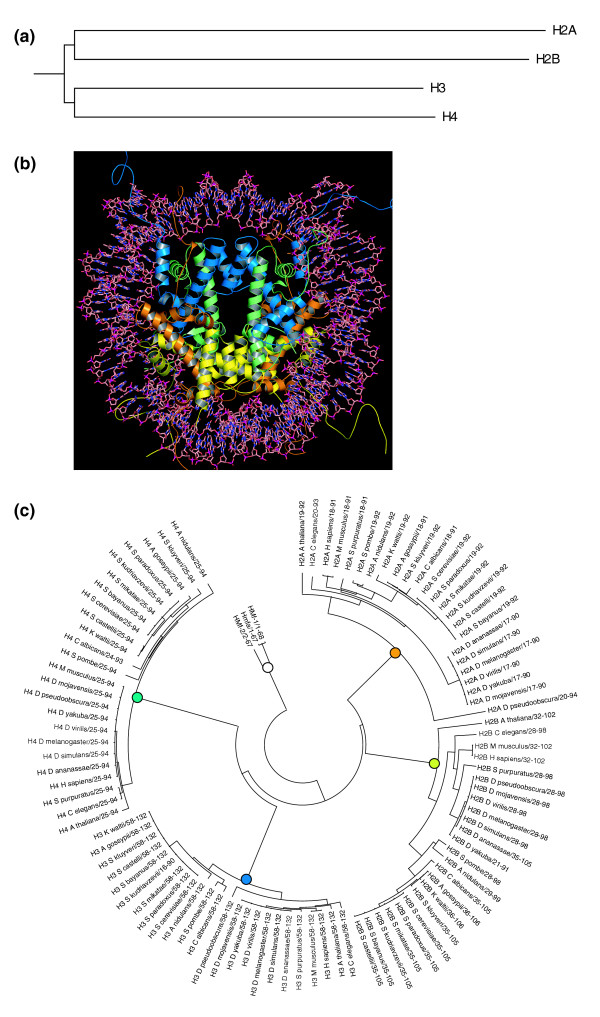
Core histone protein structure and sequence evolution. **(a) **Structural relationships between the four core histone protein families. **(b) **Three-dimensional nucleosome structure is shown with each core histone chain colored: H2A, orange; H2B, yellow; H3, blue; and H4, green. **(c) **Sequence relationships within and between the four core histone protein families. Internal nodes that set-off each core histone family are color coded using the same scheme used for the nucleosome structure. The tree is rooted with archaeal histone-like sequences (white internal node).

In order to explore the sequence/structure relationships within and among core histone protein families, sensitive methods of comparison are needed. For instance, comparisons of three-dimensional protein structures [36] can often reveal deep evolutionary relationships that are not apparent when protein sequences alone are compared. A high-resolution structure of the *Xenopus laevis *nucleosome exists, and structural comparison of the individual histone units, which correspond to distinct histone families, was performed using similarity scores from the DALI database [37]. The statistically significant similarity scores observed indicate that the signal of common ancestry among all histone families is preserved at the structural level. For each histone variant, its pair-wise DALI *Z *scores were normalized by the self-comparison of *Z *scores (Z_*ij*_/Z_*ii*_) to yield a relative *Z *score (*Z*_*r*_), and the distance was taken as d = 1 - *z*_*r*_. The resulting pair-wise distance matrix was used to build a neighbor joining tree for the four histone families (Figure 8a). This tree shows that H2A and H2B form one related cluster, whereas H3 and H4 form another. Interestingly, these evolutionary relationships are reflected in the structure (Figure 8b) and assembly dynamics of the histone octamer [38]. H3 and H4 first form dimers that come together as a tetramer. Meanwhile, H2A and H2B form dimers separately and these H2A-H2B dimers join the H3-H4 tetramer to form the octamer.

A more detailed analysis of the evolutionary relationships within and between histone protein families was performed using a comparative analysis of the HFD. The HFD is represented in the Pfam database, and an alignment of its representative members has been used to generate a hidden Markov model (HMM) that captures the position-specific sequence variation characteristic of the domain. In order to build a multiple sequence alignment that unites members of all four families, representative members of each family from the 24 species analyzed here were aligned in register to the HFD-HMM. This HFD multiple sequence alignment was then used to calculate all pair-wise distances, within and between families, and to build a HFD phylogeny (Figure 8c). As expected, all members within any given family are more closely related to one another than to members of any other family. The phylogenetic relationships within families are largely consistent with the established taxonomic relationships of the species from which the sequences were derived. However, the relatively high within-family sequence identities, as well as the level of resolution afforded by the between-family HMM approach, do not lend themselves to robust delineation of evolutionary relationships within families. Perhaps most germane is the fact that the between-family relationships illustrated by the HFD-HMM approach are identical to those seen in the DALI structural comparison. It is worth reiterating that these family-specific protein sequence relationships are totally discordant with the largely lineage-specific promoter sequence element relationships.

## Conclusion

We have demonstrated a striking dissonance between the deep evolutionary conservation of core histone regulatory phenotypes and the profound divergence of their regulatory mechanisms. Core histone genes exhibit similar cell cycle (S phase specific) expression patterns from the yeast *S. cerevisiae *to human (Figure 2). This regulatory conservation is consistent with the high levels of sequence conservation among core histone proteins. Nevertheless, the regulatory mechanisms that are used to achieve the conserved expression patterns of core histone genes are almost entirely lineage specific. The cis-trans machinery involved in core histone gene regulation has changed substantially between lineages through gain and loss of transcription factor proteins and their cognate binding sites. This suggests that, for families like the core histone genes, phylogenetic footprinting [39] may have limited utility for identifying functional regulatory elements across all but the most closely related species.

**Figure 9:**
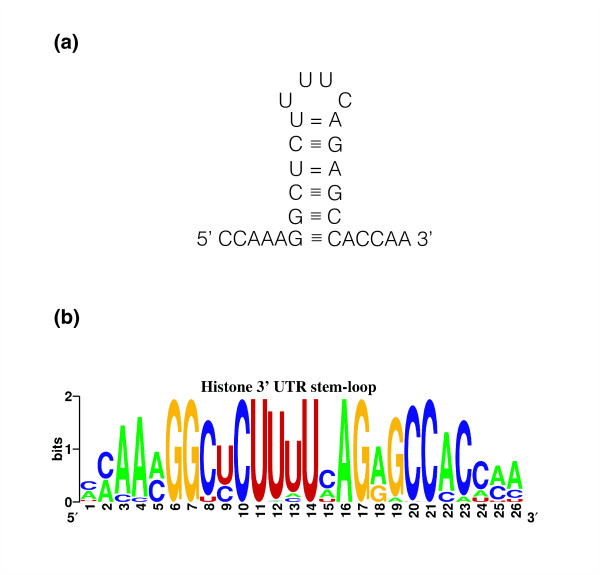
Structure and conservation of the histone 3'-UTR stem loop. **(a) **Schema of the 3'-UTR stem loop structure present in metazoan mRNAs. **(b) **Sequence logo representation of the histone 3'-UTR stem loop. The sequences (accession number: RF00032) were obtained from the Rfam database [68]. UTR, untranslated region.

In addition to the divergence of cis sites and trans factors, a distinct level of post-transcriptional regulation of core histones emerged along the metazoan evolutionarily lineage [40]. Core histone gene 3'-untranslated regions encode a stem loop structure (Figure 9a) that, when bound by protein, greatly increases mRNA stability. This mechanism is responsible for 70% of the upregulation of core histones in S phase. The sequence that forms the stem loop is conserved across metazoans (Figure 9b). The emergence of this mechanism may have allowed for some of the turnover of the cis-trans regulatory machinery among metazoan genomes subsequent to their divergence from the yeast evolutionary lineage.

There are additional regulatory elements that may help to achieve coordinated regulation of core histone genes in metazoans. For instance, a sequence found in core histone gene encoding regions is important for their expression and may serve as an internal promoter element common to the mammalian lineage [41-43]. In addition, the transcription factor NPAT has been implicated as a global regulator of core histone gene expression among metazoans even though it does not seem to bind any DNA sequence directly [44-46]. This may provide yet another global lineage specific regulatory mechanism that distinguishes the metazoan mode of core histone gene regulation from that of yeast.

Even though the four core histone gene families (H2A, H2B, H3, and H4) diverged before the species studied here, the regulatory mechanisms are more similar for different family members within species than for the same family members between species (Figures 4 and 5). Thus, there is a kind of concerted regulatory evolution operating between members of different core histone gene families. This pattern stands in stark contrast to the pattern of core histone sequence evolution, whereby members of the same family are more similar to one another across species reflecting their more recent common ancestry (Figure 8). This suggests that very different modes of evolution exist for histone gene regulation versus protein sequence and structure. The solution space for promoter sequence evolution (the space of functionally viable cis-regulatory binding site sequences) may be far more vast than that of core histone protein sequences. This results in a much more dynamic evolutionary paradigm for promoter sequences and the transcription factors proteins that bind them. Purifying selection may be less efficacious at eliminating variants of cis-regulatory sites because a number of sequence variants may bind transcription factors with similar affinities. In addition, new cis-regulatory sites, which are short and degenerate by nature, may arise relatively quickly through mutation along the promoter. It is possible that these new variants can lead to an exploration of expression space and rapid fixation of adaptive variants by positive selection. Adaptive expression changes of this type may be facilitated by the emergence of intermediate redundant regulatory programs that maintain the ancestral expression pattern and function while simultaneously allowing for selective testing of novel expression patterns [47]. Such an evolutionary mode, with less pronounced purifying and more prominent adaptive selection, could explain the observation that novel cis-trans combinations are subject to substantial turnover and may be regularly reinvented among evolutionary lineages. In addition, the inherent evolutionary flexibility of regulatory systems may allow for coordinated within-species changes that respond to epistatic pressure from other regulatory pathways in the same lineage that share transcription factors.

It is currently unclear whether the turnover of regulatory mechanisms, in the face of conserved expression patterns, is unique to core histones or also occurs for other gene families. Some studies on the evolution of gene regulation do report evidence of conserved regulatory sequences and expression patterns [47,48], whereas others indicate that gene regulatory networks do in fact diverge rapidly [49-51]. However, regulatory divergence usually leads to distinct expression patterns [51-53]. Interestingly, although yeast core histone transcripts include polyA tails, core histone transcripts are unique among metazoan transcripts in that they lack polyA tails. The absence of polyA tails, which are often bound by poly(A)-binding proteins to promote translation initiation, may necessitate, to some extent, species-specific solutions to core histone gene regulation.

The comparative genomics of core histone gene regulation reveal a novel evolutionary mode, which we dub 'circuitous evolution'. Circuitous evolution of core histone gene regulation is distinct from convergent evolution, because the conservation of the core histone gene regulatory patterns suggests that the same pattern existed in the last common ancestor of all species analyzed here. After divergence from the last common ancestor, the core histone expression patterns remained unchanged but the regulatory mechanisms that give rise to the conserved phenotype diverged dramatically. Thus, with respect to core histone gene regulation, where you are from and where you are are far more important than how you get there.

As an addendum, during revision of the manuscript we became aware of a recently published paper [54], which confirms that the specific periodic pattern of core histone gene expression is uniquely evolutionarily conserved. The report by Jensen and coworkers also demonstrates how many different regulatory solutions have evolved to control the periodic expression of integrated biological systems that function in the cell cycle.

## Materials and methods

### Promoter sequences

Core histone protein coding sequences were obtained from the histone database [3]. A list of species from which the sequences were obtained is provided in Additional data file 3. Core histone protein coding sequences were used as queries in a series of tblastn [33] searches against species-specific National Center for Biotechnology (NCBI) Entrez Genome project databases [55] in order to locate the precise genomic regions of core histones. Entrez Genome project species-specific databases include complete Reference Sequence (RefSeq) genomes when available or whole genome shotgun sequence entries when RefSeq versions are unavailable. Core histone proximal promoter sequences were taken as 1 kilobase upstream of the annotated translational start site. For bidirectional promoters, the entire intergenic regions (range 133 to 970 bp; average 413 bp) were taken for analysis. Promoter sequences are provided as Additional data file 1. The nomenclature reported by Marzluff and coworkers [56] was used for the human and mouse core histone genes.

### Cis-regulatory binding sites

The DNA binding subunits of the transcription factor proteins and their cognate cis-regulatory binding sites were taken from the published literature as described in the Introduction and Results and discussion sections. PFMs of the cis-regulatory motifs were taken from the TRANSFAC database [29] or, when not available, generated from the binding site alignments reported in the original citation. The PFMs were used with the program CLOVER [30] to search the core histone promoter regions for the presence of the cis-regulatory motifs. The complete set of CLOVER predictions is provided in Additional data file 4. CLOVER output was used to construct promoter-specific vectors composed of scores of over-represented and/or under-represented cis sites for each sequence; the vectors were then used to compare promoter sequences with pair-wise Pearson correlation coefficients. CLOVER also gives the position of each predicted motif and these positions were normalized by the length of the promoter sequence to give the relative lengths shown in Figure 7 panels a, c and e. Locations were randomly sampled from a uniform distribution in order to generate the negative control plots shown in Figure 7 panels b, d and f.

The TFBS Perl modules [57] were used to further analyze cis-regulatory sequence binding motifs. For each cis-regulatory motif, sequences of all the motif sites predicted by CLOVER were extracted and aligned. These alignments were used to construct PFMs, which were converted to position weight matrices by normalizing with background nucleotide frequencies. Information content per cis-regulatory sequence motif position [58], taken from the position weight matrices, were used to build sequence logos with the program WebLogo [59].

### Protein sequence and structure

Core histone protein sequences were taken from the histone database [3]. Protein sequences are provided in Additional data file 2. A probabilistic HMM representing the HFD found in all core histone proteins [34,35] was taken from the Pfam database [60]. The HFD (Pfam accession number: PF00125) was extracted from each core histone protein sequence using the HMM with the program HMMER [61]. The core histone HFD multiple sequence alignment was built by aligning each HFD sequence back to the PF00125 HMM, thus preserving the same structural register for all HFD domain sequences. The program QuickTree [31] was used to build a neighbor joining tree [32] from the HFD multiple sequence alignment.

A three-dimensional structure of the nucleosome core, PDB ID:1KX5 [62], used for comparison was taken from the RCSB Protein Data Bank [63]. Structural comparisons between the individual core histone proteins were performed using the fold classifications computed in the Dali database [37,64]. *Z *scores between individual core histone proteins were taken and converted to pairwise distances (*d*) by normalizing with the self-similarity *Z *score using the following equation:

dij=1−ZijZii
 MathType@MTEF@5@5@+=feaafiart1ev1aaatCvAUfeBSjuyZL2yd9gzLbvyNv2Caerbhv2BYDwAHbqedmvETj2BSbqee0evGueE0jxyaibaiKI8=vI8tuQ8FMI8Gi=hEeeu0xXdbba9frFj0=OqFfea0dXdd9vqai=hGuQ8kuc9pgc9s8qqaq=dirpe0xb9q8qiLsFr0=vr0=vr0dc8meaabaqaciGacaGaaeqabaqadeqadaaakeaacaWGKbWaaSbaaSqaaiaadMgacaWGQbaabeaakiabg2da9iaaigdacqGHsisldaWcaaqaaiaadQfadaWgaaWcbaGaamyAaiaadQgaaeqaaaGcbaGaamOwamaaBaaaleaacaWGPbGaamyAaaqabaaaaaaa@3EC0@

Pair-wise distances were used to calculate a neighbor joining tree [32] using the program MEGA [65].

### Gene expression

Gene expression data were taken from reports published elsewhere [8-13]. For Figure 2, relative expression levels (log_2 _ratios) for *S. cerevisiae *were plotted against cell cycle time points, and visualization was done using matrix2png [66].

## Additional data files

The following additional data are available with the online version of this article. Additional data file 1 contains the promoter sequences of core histone genes used in the study. Additional data file 2 contains the core histone protein sequences used in the study. Additional data file 3 contains the list of species used in the study. Additional data file 4 contains the CLOVER predictions for all core histone gene promoters used in the study.

## Supplementary Material

Additional data file 1 Promoter sequences of core histone genes used in the study Click here for file

Additional data file 2 Core histone protein sequences used in the study Click here for file

Additional data file 3 List of species used in the study Click here for file

Additional data file 4 CLOVER predictions for all core histone gene promoters used in the study Click here for file

## Figures and Tables

**Table 1:** Distribution of core histone regulatory motifs among human and yeast

*Motif*^*a*^	*Human*^*b*^	*Yeast*^*b*^
Spt10	-	+
NEG	-	+
TBP/TATA box	+	+
CCAAT box	+	-
Alpha-CP1	+	-
Oct-1	+	-
IRF-7	+	-
GC box	+	-
HEX	+	-
E2F	+	-
HiNF-D	+	-
IRF-1	+	-

^a^Name of the cis-regulatory binding motif/transcription factor. ^b^Presence (+) or absence (-) of the element in human or yeast (*S. cerevisiae*).

**Table 2:** Phyletic distribution of core histone transcription factors

*Transcription factor*	*RefSeq accession (protein name)*^*a*^	*Phyletic distribution*^*b*^
E2F	NP_005216 (E2F1)	Metazoans and plants
	NP_009042 (TFDP1)	Metazoans and plants
TBP	NP_950248 (TBPL2)	Eukaryota
Sp1	NP_038700 (SP1)	Metazoans
HiNF-D	NP_853530 (CUTL1)	Metazoans
Oct-1	NP_002688 (POU2F1)	Metazoans
IRF-7	NP_058546 (Irf7)	Vertebrates
IRF-1	NP_032416 (Irf1)	Vertebrates
NF-Y	NP_002496 (NFYA)	Eukaryota
	NP_006157 (NFYB)	Eukaryota
	NP_055038 (NFYC)	Eukaryota
Spt10p	NP_012408 (SPT10)	Ascomycota

^a^Accession identifier from the NCBI Reference Sequence (RefSeq) database along with official protein name for the DNA-binding protein. ^b^Deepest taxonomic node(s) that covers the phyletic distribution of the transcription factor.
