# Supplementary material for: Multiple independent evolutionary solutions to core histone gene regulation
Source: Genome Biol. 2006 Dec 21;7(12):R122. doi: 10.1186/gb-2006-7-12-r122 (PMC1794435; doi:10.1186/gb-2006-7-12-r122)
Supplement: Additional data file 1 — Promoter sequences of core histone genes used in the study [file gb-2006-7-12-r122-S1.doc]

>H4.1_H3.1_IR_A_gossypii

CCATTTTGCTTGCTTGCTGTCGTAGAAGGCATTCCCGGAGCTCCAACCGCAGACTTTAAATATGCTCCAGCGCGTCCTGCGCCGCGGCCGTATCGCAGCGCTTTGCCGCAGCTCTGTAAGCGCTGCCGCGGCCGTGCAGCCAGAAGCAACTGGACCGCGGATTCGTGCTGGCACGCATGGCGCGCGAAAAAACTGGTCCGCTCATACCAGTTTCTTCGCGCAGCACGGCACGGCTAACATGACGCTTCAGGGCGTGCTGTGCGTTTCTCAATGCCTGGGACTACGCGTATCCTGCGGTACGGCGTGCACGCTGGCCCGCTCGCGCACACGCTACGCAATAGGGGCTAGTGGGCTGTTGTTCGCAGATTGTGTATATAAGGCGGCGTACGAGCTTAGGCTGAGTTCTCGATATCAGAGGTTGTACAAACACAACAAATCAACACAACAACAAAGAACAATGT

>H3.2_H4.2_IR_A_gossypii

TGTTTGCTGCGGGGAGGCAGAGGGGAACAAACCCGCGCGCCCGCCCCGTGTTTATATACGCCCGCCCCTTTCTTTTTCCCGCCGTGTCTACCCATACTAATCGGCCGCTTCGCTAGCGTGCGCGGCCGCGGGCCGCAGCGCCCGGTACCGCGCCTTCGCCGCGGGGGGGCGAAGCGCGCGGGCCGCCGGTCCCCCTGCACGGCGCGCGCGCGCGAAAAAACTGGACCGCCCCTGCCGTATAAATAGCCCGCGCGCGCGCCGTGGCCCCCACGAGCACCAAGCA

>H2A.2_H2B.2_IR_A_gossypii

AGACATTATTTGTGTTGTGTGTGTGTGTGTGTGTTTAGTGTGAACTGCGTGTGCTATGAGAAAACACTACGCTGAAACTGCTAAATAATCCAGACAGGTCCCCCCACCGCAAAGGATCCACGCTATACTTCTCTCTACATATTTATACTTGTCCTTTTGCCTTCTAATCCTCGATCGTACGCGTCTGACGCTTCAACAGACGCTTCACCTAGACGCTCGACCTGTGCGGCCTGGTTTTTTCGCATGACATGTCCGTGCTGGTTTTTTCGCGCTGAAAAGGAAAGCGCGTGGCTCCCAGCACCAGAGCCGTACTAGCTCTTTCGCGTGCTGTCCTATGTGCACGCGAAATTTTCATACTGTAGAGTGTGCCATCAGCTTCACAGAGTACAAACGGTAGGCGAGTGGATACGCGTCTTGTAGCCGGACGTGAATGGCAGAACTTTTTGGCAGTCGCGTAATCTTAGATTGAAAGTATTTAAGTGGAACGTATAAAACAAAAGTTCGGGCTGAAGAGGACCTCTTTTGGCGTCTGCTACTTCCCAGTTATCTGTTGGATACTAAGCATATCGAACTCTAATTGCAATTCTAAAG

>H2B.1_H2A.1_IR_A_gossypii

GGTTGCGGTTGCGCTGGCCCGCTGGTGGCTAGCGGCTGCCCCTGTGTTTATATAGTTTTCGCCGTGCAAGGGGCGTGCCGGGGGCTTCGGTCGCGAAGCTGCGGGCACGCGCGCAGATTTGGCAGAATTACCCGAATAGGCAGGCGCGATGGCCGCCCGTAGTTAAGCCGGGGCGGGCAATTACGGCAGACACGGCTTGGCTGCGACCGGAAGCGCCACGCCAGCATGGTGCCAGTTTTTTCACAGAGGCTGCGAAACGCGTGGCACAGGGGCACGGGCGCGCGCGCCGCAGAGCGGTATATAAAGGCCCCCTGGGAGGACACCGGCAGGTTAGGCAGAGCAGCATCGAACAAGAACATGTCA

>H4.1_H3.1_IR_A_nidulans

GCGTTAGGATGATTGAGATAGCCTCATGGTGATATGGATGTCACTTACGTCCAGACATGTTGATGTGTGAAGAGATTTAAGGTTAAAGTTGTGAGGTGGAGATAAATTAAACGTCAAGTGGACGGCGAGAGATGTTGAGAATGGAAGACCGGATGGGCTCCCACGGGACGGCAGATATTTGTATAGAAGACGGAAGCGCGTGGGGAGAGAGCTGGAATCACTCACTGTCGGAGGATCGACCAATCAGAGGGCGGGGCATGTTGATCTGATGCGCGGCCCTAGACGGGCGGTAACGTGACCATGACGCAGTTGATCTTGCAAGAGGAATTTGATCGCGGCGCGAGGACGCGTCAAGACGCGGATTTGAGATTTGAGAGTGCATAGTAAATTCCTACGTACTACAGAGACTCCTGGAATTCTTCTGATATCCTAAAAATTCAGGGATGGGGTTGGGATCAGTATGTAACTGGGGGACATTCCAGATCAATGATCCTGAATGCTTATTGGCTGCCCGGGAAAGTCTCGCTTAGCGCAACTGCCAGCCTGACCGCCCTGGCCCGCAACCCTTTCGATCTTGCCAGCAAAACTGGGCCGCGAAATCACTGCTTATGGTGTGCTGGTTTCCGGACGGTTTTTTTGATCAAAGGAAAGGGCGCTAAGACCACCCGGCTTTGCAGCCAAACCACAGGATCACCACCCGCCTCTCGCTTACTACAAATACCATCCCCCACCCGCCCCCTCCATCTTTCTTTCATCTATCCTTTCCGACATCCTATATTCGGCCATCGACTTTCCATCAATTCTATACCATTTTCATCTCAAAGCCCCACTAATTCCATCAAAACTCATCGTCAATAATGGCTCGCACTAAGCAGACTGCCCGTAAGTCCTCACTTTGTTGCCCTACCTCTCGTGTCGTGGTCGTGTCACGGTCGCGTCTCCCAACTGCTTCACGATGCTAACTTTACCCCAACAGGCAAGTCTACTGGTGGCAAGGCTCCCCGTAAGCAGCTCGCGTCCAAGGCTGCCCGTAAGGCCGCTCCCTCCACTGGAGGTGTCAAGAAGCCTCACCGCTACAAGCCTGGTAAGTAATACCTATGACTTGGTTCGTGGTTGCGTCTCTAACGTACTCCAA

>H3.2_H4.2_IR_A_nidulans

TTGGAGTACGTTAGAGACGCAACCACGAACCAAGTCATAGGTATTACTTACCAGGCTTGTAGCGGTGAGGCTTCTTGACACCTCCAGTGGAGGGAGCGGCCTTACGGGCAGCCTTGGACGCGAGCTGCTTACGGGGAGCCTTGCCACCAGTAGACTTGCCTGTTGGGGTAAAGTTAGCATCGTGAAGCAGTTGGGAGACGCGACCGTGACACGACCACGACACGAGAGGTAGGGCAACAAAGTGAGGACTTACGGGCAGTCTGCTTAGTGCGAGCCATTATTGACGATGAGTTTTGATGGAATTAGTGGGGCTTTGAGATGAAAATGGTATAGAATTGATGGAAAGTCGATGGCCGAATATAGGATGTCGGAAAGGATAGATGAAAGAAAGATGGAGGGGGCGGGTGGGGGATGGTATTTGTAGTAAGCGAGAGGCGGGTGGTGATCCTGTGGTTTGGCTGCAAAGCCGGGTGGTCTTAGCGCCCTTTCCTTTGATCAAAAAAACCGTCCGGAAACCAGCACACCATAAGCAGTGATTTCGCGGCCCAGTTTTGCTGGCAAGATCGAAAGGGTTGCGGGCCAGGGCGGTCAGGCTGGCAGTTGCGCTAAGCGAGACTTTCCCGGGCAGCCAATAAGCATTCAGGATCATTGATCTGGAATGTCCCCCAGTTACATACTGATCCCAACCCCATCCCTGAATTTTTAGGATATCAGAAGAATTCCAGGAGTCTCTGTAGTACGTAGGAATTTACTATGCACTCTCAAATCTCAAATCCGCGTCTTGACGCGTCCTCGCGCCGCGATCAAATTCCTCTTGCAAGATCAACTGCGTCATGGTCACGTTACCGCCCGTCTAGGGCCGCGCATCAGATCAACATGCCCCGCCCTCTGATTGGTCGATCCTCCGACAGTGAGTGATTCCAGCTCTCTCCCCACGCGCTTCCGTCTTCTATACAAATATCTGCCGTCCCGTGGGAGCCCATCCGGTCTTCCATTCTCAACATCTCTCGCCGTCCACTTGACGTTTAATTTATCTCCACCTCACAACTTTAACCTTAAATCTCTTCACACATCAACATGTCTGGACGTAAGTGACATCCATATCACCATGAGGCTATCTCAATCATCCTAACGC

>H2A.2_H2B.2_IR_A_nidulans

TTTGATTGATTTGGAGAATCAGGTTATTCGAAAAGTAGCTGAGATGCGAAAACGACGCGTAGGTGTCGGTGTAAGAGGGTAGGAGCAAGAAATGATAAGTGGAAAATGGGAAGGGACGACAGCTATCGCGAAGGAGCGGAGGGCATATATAACGACACCATGAAAGTAACGCCGGGCAGTCCAGACGCGTTTTGGATCACCCTCTCGCTTAGCGGGGTCAACCAATCAATGAGCGGATCATCATCGATTGTACCAGTTGGACGGTGTCGTCATGTTACAATACCAGATTGAGAAAGGTGCCCAGACTAATCGAAAAGCCTTACCGTACCGTATCCTTGGCAAGCTCCTGCCGCCAATCTTGCCGCGTTTCGCGCATTCTGAGCTTTCTAATCATACAAATCATACAATTTCAATACCGTATCGAAAAACTGCCTCCTTTGTGCTTTTTGGATCAACAGGGCGTTTGGAGCCAAGTATAAGTAGCGCGCTTCCCCTCGTCACCGCGTATTCTTAACTTTCATCCTCCTACTCATTCCACCCCACTACTCTCACGCGTCAACCAGTGCTCTCTTGCATCCCCATTCATAGTCAAGTCACTCATCCATTACCGAATTCAACTTTCGAATTTATCAACTTTTAAAATGCCTCCCAAAGCTGCCGAGAAGAAGCCT

>H2B.1_H2A.1_IR_A_nidulans

TGCCGCCAGTGCTAGGCTTCTTCTCGGCAGCTTTGGGAGGCATTTTAAAAGTTGATAAATTCGAAAGTTGAATTCGGTAATGGATGAGTGACTTGACTATGAATGGGGATGCAAGAGAGCACTGGTTGACGCGTGAGAGTAGTGGGGTGGAATGAGTAGGAGGATGAAAGTTAAGAATACGCGGTGACGAGGGGAAGCGCGCTACTTATACTTGGCTCCAAACGCCCTGTTGATCCAAAAAGCACAAAGGAGGCAGTTTTTCGATACGGTATTGAAATTGTATGATTTGTATGATTAGAAAGCTCAGAATGCGCGAAACGCGGCAAGATTGGCGGCAGGAGCTTGCCAAGGATACGGTACGGTAAGGCTTTTCGATTAGTCTGGGCACCTTTCTCAATCTGGTATTGTAACATGACGACACCGTCCAACTGGTACAATCGATGATGATCCGCTCATTGATTGGTTGACCCCGCTAAGCGAGAGGGTGATCCAAAACGCGTCTGGACTGCCCGGCGTTACTTTCATGGTGTCGTTATATATGCCCTCCGCTCCTTCGCGATAGCTGTCGTCCCTTCCCATTTTCCACTTATCATTTCTTGCTCCTACCCTCTTACACCGACACCTACGCGTCGTTTTCGCATCTCAGCTACTTTTCGAATAACCTGATTCTCCAAATCAATCAAAA

>H4.1_H3.1_IR_C_albicans

CCATTGTGAATGTTGATATAAAATGATAATTAAAAAAAAAATAAAAAAATAATTACTGATCTTTTGGGATGGAATCAAAGGAGTTTAAATATTGATTTGTTGATTTGGGATCTTTCTTTCAATTCTGTGTAAGTAACGATAGCGTGAATGTTTGTCCTTTGTTTTTTTTTTTTGCAATTTCTTTTCTCTTCTCTCTCTTCCTCTCTCCTTCTCTCTCGTTCTTTCGCGCGTTGGTAGTAAAAAGTTTGTTTTTCGTTTCCTGAGGGTGTGTGTGTCTTTAGTGGTCGACTAAAGAACTAATGCGCGCCCAAAAAAGCTCTTTTGTTACCAACAAAAGACACGCTTTGGTCTCTCTCTCTCTCTCCGTCTTTCTCTCTCTTGTGTACAAAATACTCCACAACAAGTAATTTGCAGATTACTCTTGTAATTATACGCCACACACACACCCCAAGATGAGAATAGATGGAACGTGTTTCCAGATTGAAGACCCCGAAACTACACGATACAGACTTGTACTTATTAATAAACTATACTACTTATGTGATAAGCTGTATAAAGTAATCCTAATGATAAATTAACAGTCTTTTGTTCCACACAAAAGATTCAAGATTGTTTCGCGGACTCAGTTTTACGCTCACCACACAAAAAAAGGTTGTCTTCTTTTTGTTCTCTTTCACTGTCGTGCTCTTATTTTCGCGCACTCTTCCAAGTGATCATGGCTTTTGTTTTTGTTTTGCACATTACGCCAAATATTTGAAATAGTCCAAACCAAACCAAACACACAAAGATCACGGTTTGGATAGTCATTCCATCAAACTGATTTGAACTATAAATATATTACTATCCGCTCATAATTTTTGGATTATAATTCTTTCTTTTTTTTTTATCTAATCAACTAAGTTTTCATTGAAGCTTTAATATTCTCCTTTAAAAACTTTTTAATTTTATAAATCAATCAACAATGTCAGGT

>H3.2_H4.2_IR_C_albicans

TTGTGAATGTTGATATAAAATGATAATTAAAAAAAAAATAAAAAAATAATTACTGATCTTTTGGGATGGAATCAAAGGAGTTTAAATATTGATTTGTTGATTTGGGATCTTTCTTTCAATTCTGTGTAAGTAACGATAGCGTGAATGTTTGTCCTTTGTTTTTTTTTTTTGCAATTTCTTTTCTCTTCTCTCTCTTCCTCTCTCCTTCTCTCTCGTTCTTTCGCGCGTTGGTAGTAAAAAGTTTGTTTTTCGTTTCCTGAGGGTGTGTGTGTCTTTAGTGGTCGACTAAAGAACTAATGCGCGCCCAAAAAAGCTCTTTTGTTACCAACAAAAGACACGCTTTGGTCTCTCTCTCTCTCTCCGTCTTTCTCTCTCTTGTGTACAAAATACTCCACAACAAGTAATTTGCAGATTACTCTTGTAATTATACGCCACACACACACCCCAAGATGAGAATAGATGGAACGTGTTTCCAGATTGAAGACCCCGAAACTACACGATACAGACTTGTACTTATTAATAAACTATACTACTTATGTGATAAGCTGTATAAAGTAATCCTAATGATAAATTAACAGTCTTTTGTTCCACACAAAAGATTCAAGATTGTTTCGCGGACTCAGTTTTACGCTCACCACACAAAAAAAGGTTGTCTTCTTTTTGTTCTCTTTCACTGTCGTGCTCTTATTTTCGCGCACTCTTCCAAGTGATCATGGCTTTTGTTTTTGTTTTGCACATTACGCCAAATATTTGAAATAGTCCAAACCAAACCAAACACACAAAGATCACGGTTTGGATAGTCATTCCATCAAACTGATTTGAACTATAAATATATTACTATCCGCTCATAATTTTTGGATTATAATTCTTTCTTTTTTTTTTATCTAATCAACTAAGTTTTCATTGAAGCTTTAATATTCTCCTTTAAAAACTTTTTAATTTTATAAATCAATCAACAATGTCAGGTA

>H2A.2_H2B.2_IR_C_albicans

TATTGTTAATATGCTATTATTTGATTTGAATATACTAAGTTTCAATTAAAATAAACTGAAGAAGAAATATAAAAAGGAAAAGCAAAAAGAAAGAATGTTTTCTTCAAAGTGGCAAACTATGCTGTATTTAAAACATTTGATTACTACTTTCTATTGTGTGCGTGCGAGCGTGTTGTGTGTGTTGTTTTCATTTGTCTATATGTTCCTCTGGTCTCAGATTTACGTAGTGCGATGGAGTATGGGAGAAAAGGGAGAGAAGAGTGGTGGAGAGAAAAAAAAAATAAAAAAGAGAATGAACAAGAGAGAAAGAGAGGGAGAGAAAGAGAGTTGATACTGGCACGGTCACGACTATTCTCTCCTTCTCTCTCTTGTCACTTCTTCTTCCTCAAAACAACAGAACTTGGTATTTGGTTATAAATAGATCAAACTTATCTTTCTTTTTTATAAAAACCATTTCCTACTTGCCACTTTTTTTTTGTTTTCTTAATTTCCATAACTTTATCGTTTCAGATTTATATCACAACCAAACAACTAATACTTCACA

>H2B.1_H2A.1_IR_C_albicans

TTATTGTTAATATGCTATTATTTGATTTGAATATACTAAGTTTCAATTAAAATAAACTGAAGAAGAAATATAAAAAGGAAAAGCAAAAAGAAAGAATGTTTTCTTCAAAGTGGCAAACTATGCTGTATTTAAAACATTTGATTACTACTTTCTATTGTGTGCGTGCGAGCGTGTTGTGTGTGTTGTTTTCATTTGTCTATATGTTCCTCTGGTCTCAGATTTACGTAGTGCGATGGAGTATGGGAGAAAAGGGAGAGAAGAGTGGTGGAGAGAAAAAAAAAATAAAAAAGAGAATGAACAAGAGAGAAAGAGAGGGAGAGAAAGAGAGTTGATACTGGCACGGTCACGACTATTCTCTCCTTCTCTCTCTTGTCACTTCTTCTTCCTCAAAACAACAGAACTTGGTATTTGGTTATAAATAGATCAAACTTATCTTTCTTTTTTATAAAAACCATTTCCTACTTGCCACTTTTTTTTTGTTTTCTTAATTTCCATAACTTTATCGTTTCAGATTTATATCACAACCAAACAACTAATACTTCACAA

>HIST1H2BC_HIST1H2AC_H_sapiens NM_003526-NM_003512 hg17_dna range=chr6:26232111-26232352 NM_003526 NM_003512

TCTTAAAACACCAGAAATGTGTCGAAAGTAAAGAGCGGATTTCTGCTACTTATAGGGCTTTTATGCTAATGAGGGATGGAGAGTACCTCTTAGTTAATTGGAAGACAAACTGCACAGTTGTCATCCGTGGGCAGAGCTATGCAAATGAGGTATGAAAGTACAGCTTTTCTATTGGCTATCTGACTAGCATTTGCTACCGACCAATCAAAAAGTCGGATTTACTCCCCAGGAACTACCTATAA

>HIST1H2AM_HIST1H2BO_H_sapiens NM_003514-NM_003527 hg17_dna range=chr6:27968942-27969182 NM_003514 NM_003527

TGAAAGGAACCTACAGGGACGCGCATTTTATAGCAGCTGCTGGGCGCGAAAAAGAAGCTGGGCCATTGGCTAAGCTTGCAGCTTCCTCTTAATGCAAATGAGCTTTCTGAATATACGTGTTTTGATTGGACTATATTGATATTAACGTCATCTGAGTAACTTCCAATCAGACAGAAGAATTTCTCAATCTCATCTGCATATAGACTTGTAAATAAATAGGGCATAACCCAGCTTGTCCTCA

>HIST1H2AK_HIST1H2BN_H_sapiens NM_003510-NM_003520 hg17_dna range=chr6:27914096-27914419 NM_003510 NM_003520

TGACGTAAAAAATTCAATCAGTAACGTTCCTGAGACTGACGTAACGCTAAAGCTCCGCTACTTATAGTCAACAGAGGCACGAAAACTAAGCTGTGCTATTGGCTAACATTACAGTTTCGCTTTAACCAATGGGATTGCGGTTTTGAAAAACACTTATTTTGATTGGACAAAGTTAATATACGTTTCCAGGACTCACCACTGGTTAAACGCACAACTTCATTCTCTACCCCACTTGCGTTAAGAAGCAGTGAATAAGCGGTAGGTTGACAGAGCTACCGTCTTCCTGTTTTTTTCCTCCAATTTTCCGGCAGTTACTCCCAGTCA

>HIST1H2AJ_HIST1H2BM_H_sapiens NM_021066-NM_003521 hg17_dna range=chr6:27890497-27890801 NM_021066 NM_003521

TGGCAAAAGGTCTATTACCTTTACGGTCAAGAAAGACTGAAATGAAATTGGAAAAACGTACATTTTATAGCCTCTACTGGGCGCGAAAAGGAAGCTGTGCGATTGGCTTACGTTTTATTTTTATTTAGACCAATAGGACTCGAGTATGCAGGATACCTATTTTGATTGGGCAAAACTGGCATCTGACGTCATCCACGGATAATCACCAATCAGCACTGACCTATTCTACTCCTAATTTGCATAATAACATTTAAATAAAGAGGACGAAACAGCCCTAAGGTTGTCTTTTATTTTGTTTTCCACCA

>HIST1H2BL_HIST1H2AI_H_sapiens NM_003519-NM_003509 hg17_dna range=chr6:27883688-27883956 NM_003519 NM_003509

AAACAAGAAGTCGGTCTCCTCTTTTTATATAATAGTTTATGCGGCCGAGGTAGTGGGAAGGTCTCTGCTGATTGGTAATTATCCGTGGATGACGACAGATGCCAGTTTTGCCCAATCAAAATAGGTATCCTGCATAATCGAGTCCTATTGGTCTAAATAAAAATAAAACGTAAGCCAATCGCACAGCTTCCTTTTCGCGCCCAGTAGAGGCTATAAAATGTACGTTTTTCTAGTTTCACTTCAGTCTTTCTTGACCGTATAGATAATAG

>HIST1H2BJ_HIST1H2AG_H_sapiens NM_021058-NM_021064 hg17_dna range=chr6:27208554-27208800 NM_021058 NM_021064

GAGCAGGGTATGACAAGGCGCTTTTATATAGAATCGCTTATGCAAATAAGGTGAAGAGTTGAAGTCTTGTGTCTGATTGGTAGTTATTCAGGGTAACGTCAGAGGTCAGGTCTGCCCAATCAGGATTCGCAAATCCAGAAGACGCACTACTATTGGTTGAAATTAAACTGCAGCCCTAACCAACAACACGTCTTCTTTTTCGCGCCCAATAGTGTTTATAAAAAGCGCCGCCTTTCCCGTTCACTTT

>HIST1H2AA_HIST1H2BA_H_sapiens NM_170745-NM_170610 hg17_dna range=chr6:25834769-25835116 NM_170745 NM_170610

CATTTCTAGGGCTGCTACTGGGCCTATTTATAGTCTGACTGAGGTTGGCATTTGCTATCTGATTGGCTGATGGCCGTCTACCCAATCAGAAAGTCGTACTAGAATCGCCTCATTTGCATTCACGCCACTTCCCATTGTCCAATCAGATTTTGGATACCGAACGCGGCGTTTGAGGGCCGTGCCTATAAATACCGCATCTTTCATCCTCCAGTTCTGTTTGTTTACTTGGCGAGACTTGGAGCTGAGGTCATTTGGAGCTGTTTAATACTGAAGAGCTGTTGAGCACTGGAAAGTGCTGTGTAACCCTGGAAAAGAACCGTGTAACGCTGCAGAAGTGTGTGGTAGCTA

>HIST1H2BK_HIST1H2AH_H_sapiens NM_080593-NM_080596 hg17_dna range=chr6:27222598-27222887 NM_080593 NM_080596

GAAAACGGGAAGTAATGGGAGCAAGGTACCAGGAGTCGTTTTTATATAGGACCTCTCATGCAAATAAGGTGAAGAGTGAAAGTCCTGTATCTGATTGGTGGTTATTAGGGTGACGTCAGAGGTTAGTTATACCCAATCAACCCAATCTGCAAATCCAAAAGACGTACTTCCATTGGTTAAAACTAAGCTACAACCCTAACCAATGACATATCTTCTTTTTCGCGCCCAATAGTGTTTATAAAAGGCGCTGCCTTTCCTCGTTGGCTACTTTCAGTAAGTTGTGACCAGTA

>HIST3H2A_HIST3H2BB_H_sapiens NM_033445-NM_175055 hg17_dna range=chr1:224952295-224952543 NM_033445 NM_175055

GACTAAAAACAAGAGGGCAGTGAAGGCCAGCGAGCCCTTATGTATGGTACAGAGGTAGGCTAGACCGCGGCGTTCGATTGGATGGCTATGGTAGCCAATCAGAAAAAGAACCTGGCACTCCTAATTTGCGTATTCCTTTCCCAGCGATGACGTAGAACAACGTTTGATCCAATCAGAAGTGAGCAAATCCTGAGCCTTCATTTGAATACAAAACGTACAAATAGAGTTACTCCGAGCGCCGCGCGTTTC

>HIST2H2BE_HIST2H2AC_H_sapiens NM_003528-NM_003517 hg17_dna range=chr1:146671305-146671598 NM_003528 NM_003517

TAAGAGAATGGGCGGGCCTGATTCTTTTATAACCACCTTATGCAAATTAGGGCTCCGAAAGTCGTTCATTTCCATTGGTCCGTGTGCGGACCTTGCGTTTTCAGCAATGCGTACGTAACAACACCGACTCTTGACTTGATTGGCCGGTTCTGAAGCCATATTAGGACCAATGAAAAACTCGTTCTTGACCCCACCCCTAGGAAAGCTTATAAAGGCTTTCCTCTTGGTTCTCTTTCAATCTTATTTTGTTGCGAGGTTCTGAGCGTTGTCTGTGTTTAACCTTGATTTCAGTCA

>HIST1H2AD_HIST1H2BF_H_sapiens NM_021065-NM_003522 hg17_dna range=chr6:26307450-26307766 NM_021065 NM_003522

TTTTGAATTCTTAAAAACGATGTTAAGCAATGAAGACAAAAATGTAAAAGTGAATTTTGTTAGCAAGTGAGAAACTATTATACTTGGAGGTCGAATTGTAAATAACGCTATTTGATTGGCTAGAACAACCAACCAATTGCAAAGAAACGCTAGAATCACCTAATTTGCATACCACAGGGCACACAGAGACAATTCATCCAATCAATGTGTAAGCTTTTAAATGCCAGTTTACGATAGGAGCTCTACAAATATTACCAGCTGCAGAGTGAGGACACTTGCATTTCTCTTTAGGTTGTGGACGAAGTGTTTATTTATCA

>HIST1H2BG_HIST1H2AE_H_sapiens NM_003518-NM_021052 hg17_dna range=chr6:26324851-26325127 NM_003518 NM_021052

GCTGTCAGAAAACAATAACAGCAGTGAGAATGAACGCACTTAAATAAAAGCTCGTGTCTAGAGTCTCTCCTTTTATAGGCCTTTCATGCAAATAAAGAATTCAAAATATCCAGCTCTGATTGGGCAATGTGTTAGTGACGCATACATGTAAAATAGCCTTCACCTTATTTCCTTTCTAATTGGTTGGCTCGTCAAAGAACAATTTTAACCAATCAAATTGCGCCTTTCACAATTCTACCGATGACTATAACTAGCTTCTTATTCCTCCATCGAGCCC

>HIST1H3I_HIST1H4L_H_sapiens NM_003533-NM_003546 hg17_dna range=chr6:27948072-27948917 NM_003533 NM_003546

TGGCTAAGTAGACGTACCTACTCAACACAAATGAGAGAAAAAAACTGATCATCCCGGTATTTATAGCTGCCCTCGCGTTCTGATTGGACAGTAGACTGTGCCATGCCACGTCTCACACACGGAATTGGCTCCCAGTTTAATCTGCTAGTGAAAGGGAGACATTCCGCCTCTGTTTTGGGTCAAGAAACGTAAAATGGTTTTGCTAGCTAAAGAAAAACAAAAACGGAAACAAAAAAAAATCCCCCAAAAGTCTAGGATTTTTCATGCATCAAAATCCTGTTTAAATATAAGCTTGATAAATCTAAACTTGCCTTGCACTATTTTAATTACGAGGTGTCATTACTGATTTGACAAAAAGTTAAAGAACGTAAAGGAAAAGTGAAACTAGAGTAAATTTGTAAATGGAGGCATTGAAGGGGATATGAAATTCGAGCGTTGTGGTATGCCAGTATTTCTCAAATTTAAACGTACTTAATGTTTTAGAAGTTGGCCGACTTTTTCTGTAAAGGGCGATATTAAAACATTTTGGGCGTTGCGGACCACAGTGTCATAGTTACACAGCCCTCGCATTGGTAGCGGTAAAGTAATCGATACAAGATAAATGAGACCGGCTGTTTCATTATGACTTTTGCAAAAACCAGAGAGGCGTTATAGATTTGGCCGTGTATCAGAGAAGCCGGCACTTTTAACCACTAAGTAATGCTGAAAGGGGCACCGAACCTCAAACTTCAGCGACTGCTTATGTGGAATCGCTAATTTGCCAAAACTGCCATACGTTTAAGTGTTCTTAAGAAATTAACTATCTTTAGTCTACAGCTCTTTCACAGACTTCATGGGTGGCCCTGA

>HIST1H3D_HIST1H2BF_H_sapiens NM_003530-NM_003522 hg17_dna range=chr6:26307443-26307770 NM_003530 NM_003522

CCGGACATTTTGAATTCTTAAAAACGATGTTAAGCAATGAAGACAAAAATGTAAAAGTGAATTTTGTTAGCAAGTGAGAAACTATTATACTTGGAGGTCGAATTGTAAATAACGCTATTTGATTGGCTAGAACAACCAACCAATTGCAAAGAAACGCTAGAATCACCTAATTTGCATACCACAGGGCACACAGAGACAATTCATCCAATCAATGTGTAAGCTTTTAAATGCCAGTTTACGATAGGAGCTCTACAAATATTACCAGCTGCAGAGTGAGGACACTTGCATTTCTCTTTAGGTTGTGGACGAAGTGTTTATTTATCATGCC

>H4.1_H3.1_IR_K_waltii

TATTATAATATGTGTTGTTTGATTGTAGAATGCGTTATTCTTACTTATTGTGAATTAAATTTGATATGGTACCTTTGGGTTCGTATTGCCGGTCTTTATATGAGAAAAAATTGCCACCATATCCTATGCGCGCGCCCCGCCCGGCGCTCCTATCCGAACCTTGCGCTTTCGTTTGTCAAATTCAGGTTTATTTTCATAGTCCGTGTTCTTCGCGTTACACGCGCGAAGAACGTGGTCCGGGCTCATGTTTTGAAAGTTCACTACCCATCATACACGGCAGATTAATTATGTCGGCCTGAGATTTACGCTTGAGACAGCTGTTAGTATAAAATGATGAGAAGAAACTGGACCGGGTTCTTTTGCAGACCAAAATCTTCGCATACAGGAAGCCTCGTTTTTACGCTAGCGCGAACTATCAATCAATCGGGGTCACATTCCAGGCCGTAGTTGAAACCCGGATTTACTATGGGACGTGGCGCTCGGCAGCTAAATTGGCCGCAAAGACGAAAAAGGAGCTTTAAGCTATATAAAGAGGAAAATATTTCATCTCACAATTTTCGTCATTACATCTTACTCATAAAGTATACCAAACTCGATTCAAACTAGTAATA

>H3.2_H4.2_IR_K_waltii

TTATTGTATGACTTTGAGTGTTTTTAGTATTGCTAGAAGGAATACGGAGAACTACTACTTGGACAAAAACTTCCTCTCTTTTTATACAGTTCCTTATCACATTTTGATCCAGACTACATTACCTTCAGTTCGTATTGGCGCAATCTACGGTCATAGTAATCTCGTATAACGGCAGTTTTGGGACCACGAAAAAAAGTGAGGTTTTGCGCGAAAATATGAGTCCGCACGCGAAACTACCGGTCCATTTAAATTAGATGCCCTGATGCGGAAGTTTTTGGGGGACACAAGGTGAAATGAAGGCGTAAATACTGGTCCAGGTTTCGGTCCGTTTTATTCACGCGTGAGGGCCATGCGAATATTGGGGCACGAACTGCGGACGGGAATTTTCACACGCGTAAATTGGGCCACCTTTTTCGCGAACGGTGCCGATTCACCATAGTAAACGCGACCTCAGGGGAATCGCAACTATTGCAAGGAGGTGGAAGCGTTTTTTGGTAGATTAGGATATCGAATATATAAAGAGGCACATACTGGTTTGTTAAGGTAAAGAGTCTTGTTGCAATTTAAAAGTATTCACTAAACAAAACAAATCAGAAAACAA

>H2A.2_H2B.2_IR_K_waltii

TTTTTCTAAGAGTTAATGATATCTATTGATACTTCCAAAGCTACTTTAGCCTAAATGTGGCGGACCTTGTACATGTTCAGTAATTTATATACTCCTTCGTACGCGTCTTGCTTAACTTTGGCGTTTAAGGGAGCTGTCCAGACCGAAATATTCGGCCTCGCGTGAGCTTCTGGAACGGGTGAGAATTAATTAGAACGACTACCGTACTCAATTCTTCGCGTGGTACTTTCAGCCAAGAAAAAAATCAAATGAAATATATAACCCCGTAAAACCTGGTACACTTTGCGGACCAAGAACTTCGCAGTGGGGTAAATTCATGAGCATTTTCGCGTGCCGCAATTAATTTGAGTTGAAGCTTCGCGCGATGATAACAGGGATCAAGGGGAAAACGAAATGTATATAAGGGACCGAAAACTGGGACCATACTGGGTTACTAGAATACACAAAGTCTTTCCAATCTCGGATAATCACACGTATCTCAAAATCACGAATCAAA

>H2B.1_H2A.1_IR_K_waltii

GTTAGATTGTGTAATAGATGCTTATGTGGCTGGTTCTACTTACTCTGCTCTGTTTCCGGCTACATCCCTGCTCTCTTTATATACGCCTTTGCCGGCCGCCTTACGTGAGCCAACGCCAAAACTAACAATCAATGAGGACCAGAAACTTCGCAACGCGACTCTGATATCGCGCGAACACACTGGTCCGGATACCCATCGTATATTAATTTTTTCTCACCAAAATTTCCCTTTTCGGAAGCCAGGACAACTCCCCCATAATGAGCGGAGACCTCGCGTTTATTACAGTATGAGATTCCTGGCGATGGAAAGCGGCGCGAGGTCGGTGTTCCAGGAAGTTCGCACGAAGGGTGAACAAAACAGAACGACGGTGCGAAGCATCACAAAAACGGGAGAACACAGTGAGCGCAAGGGGTGGCGTGGACCGCAAAGGAGGCGCGAGGCAGGTGGCGAGCCACTTGGGGGTGAAAGTATATAAAGGAGGTAGGTGGTGTTACAGTAGGTGGATTCATCTTGAGTTGGTGAAGTATAGTAAGGATATTACAACAACAACAAACAATTACACAATAGAAAAATGTCA

>H4.1_H3.1_IR_M_grisea

TGTGATTGATTTGTGATTGATGAAAAATATGAAGTTATGGAGGTCGCGATTGTTGATGGCGAAAAGGCGATGTTGATTGATGAATGTTGTTGATTGTTGGTTGGTTGAGTGGGTGATGATGATGTAGAAGGGAAGGTTGGAGAGTGGTGGGGAGGCGGGCGAAAGGTTTAAGTAGGATGGCGGAGCCGGCAGTCACGGGCTGGCTGATTTGATTTCACCGGGTGGCGTGGGGCGGTGTGTTGAGTGAGCGTGTGCGCGCGCGCCTGAAAACCTCGATCAAAAAAAAAAAACCGCGCCTGTTTTTGGAAAGCACCGACCGGGGTTGGTTTGAGTGCCAAAAGGTGTCAGATTTGGCTGGCAAAAAAAGCGGGTAAGTTTGGGACAAACAGAGGTCCCCTTGGCGCAAAAACCCGCCTCATTGATAAAAAATTCGGATCTGGCCCAAGCGTTCAAGACATATTGCAGCACTGCAACTCCACATGCGGACGTCCTTATTTCGGCTTGCAGATTTTTGATTGGACAGTTTGTTTGTCGGCCGAATTAGGTAAGGTCGTCCCCCACGAATCACACTCTGCCAGCTTGGTATTTCCTTTTGACGCGCTTGGGTTCATAGGCAACAAAATCACTACAGGGGCTGGGTGAGTGGAGGTCAAGCGCGACTATGCTCAAGATGATCGCGTCTGGTAAAGTGCTCTCTCCCTAAAATCTGCAGGTGCGAGACAGGCCACAGGTACTGCTGTGCCCATCAGATATTCTAACCTATTCGTAGATGCAATCCTAGTTGTTTTTTTAATTCATAGTACCGCATCTGCTGCACAGCCGCCATCTACCTGAATATGTTAAACAAGTTACATTGCTTCCCTAGAGTCCAGAGCACCTTAGGGGAGCGAAATCCTCCAAACCATGACAGTCAGTATCTGAGAGACATCCGACGCGTCACAACTCAAGTGACCCTTGTCTCATGGGGGTTGGTAAATGGCATGGGTTGAGCCACCAGGTCTCGGTTTCCTTTCCTCATAATGTGGTTCGGAATGTGGCTTGCGTTCAATAATGCACAGTGGATCTCTTGCTTTGCAATAATTAGGACCAATCAAAATATTCGGGCGCCTTTGATATGCGGGGAGGCTGGTAAAGTGCCGCCAATGTCCGTGCCGAAAGCGAGCAGAAAGAAAATACACAGAGAAAAACCTAAATCAGGGGTGGGTGCAGGGGCCGTTTGAGTGACGTGCACGCGACAGAAGCGTTTAGGTGGGGCGGCAACCTCCAAATCACTTGAGCTGACTGATCCGCGTCAGAAACAGTCTTGTACTTAAGAAGAGCTTCCCCACGCTCGACTTCCATCAGCTCTATATCCCAATCATCTTTCCACCCCCAAACATACCAACCAAAAAACATATACATTCATCCTAGTCTGTGAACCCCAAGAGCCGCATCTTGAGCCTTCATTTAACCTTCTCTCAACCCACAACACCTCAATCAAC

>H3.2_H4.2_IR_M_grisea

TGTGATTGATTTGTGATTGATGAAAAATATGAAGTTATGGAGGTCGCGATTGTTGATGGCGAAAAGGCGATGTTGATTGATGAATGTTGTTGATTGTTGGTTGGTTGAGTGGGTGATGATGATGTAGAAGGGAAGGTTGGAGAGTGGTGGGGAGGCGGGCGAAAGGTTTAAGTAGGATGGCGGAGCCGGCAGTCACGGGCTGGCTGATTTGATTTCACCGGGTGGCGTGGGGCGGTGTGTTGAGTGAGCGTGTGCGCGCGCGCCTGAAAACCTCGATCAAAAAAAAAAAACCGCGCCTGTTTTTGGAAAGCACCGACCGGGGTTGGTTTGAGTGCCAAAAGGTGTCAGATTTGGCTGGCAAAAAAAGCGGGTAAGTTTGGGACAAACAGAGGTCCCCTTGGCGCAAAAACCCGCCTCATTGATAAAAAATTCGGATCTGGCCCAAGCGTTCAAGACATATTGCAGCACTGCAACTCCACATGCGGACGTCCTTATTTCGGCTTGCAGATTTTTGATTGGACAGTTTGTTTGTCGGCCGAATTAGGTAAGGTCGTCCCCCACGAATCACACTCTGCCAGCTTGGTATTTCCTTTTGACGCGCTTGGGTTCATAGGCAACAAAATCACTACAGGGGCTGGGTGAGTGGAGGTCAAGCGCGACTATGCTCAAGATGATCGCGTCTGGTAAAGTGCTCTCTCCCTAAAATCTGCAGGTGCGAGACAGGCCACAGGTACTGCTGTGCCCATCAGATATTCTAACCTATTCGTAGATGCAATCCTAGTTGTTTTTTTAATTCATAGTACCGCATCTGCTGCACAGCCGCCATCTACCTGAATATGTTAAACAAGTTACATTGCTTCCCTAGAGTCCAGAGCACCTTAGGGGAGCGAAATCCTCCAAACCATGACAGTCAGTATCTGAGAGACATCCGACGCGTCACAACTCAAGTGACCCTTGTCTCATGGGGGTTGGTAAATGGCATGGGTTGAGCCACCAGGTCTCGGTTTCCTTTCCTCATAATGTGGTTCGGAATGTGGCTTGCGTTCAATAATGCACAGTGGATCTCTTGCTTTGCAATAATTAGGACCAATCAAAATATTCGGGCGCCTTTGATATGCGGGGAGGCTGGTAAAGTGCCGCCAATGTCCGTGCCGAAAGCGAGCAGAAAGAAAATACACAGAGAAAAACCTAAATCAGGGGTGGGTGCAGGGGCCGTTTGAGTGACGTGCACGCGACAGAAGCGTTTAGGTGGGGCGGCAACCTCCAAATCACTTGAGCTGACTGATCCGCGTCAGAAACAGTCTTGTACTTAAGAAGAGCTTCCCCACGCTCGACTTCCATCAGCTCTATATCCCAATCATCTTTCCACCCCCAAACATACCAACCAAAAAACATATACATTCATCCTAGTCTGTGAACCCCAAGAGCCGCATCTTGAGCCTTCATTTAACCTTCTCTCAACCCACAACACCTCAATCAAC

>Hist3h2bb_Hist3h2a_M_musculus NM_206882-NM_178218 mm6_dna range=chr11:58680142-58680315 NM_206882 NM_178218

TCACTCCCTTTGATTGAATACAATTTTACATTACGTCATCGCGGGGTAAGGCATATGCAAATTAGGAAGGCTCCGCGTTCTTTTCTGATTGGTTCTTGTTGGCATCCAATAGGAAGGTGCAGTCTAGCCTCCCTCCTGATCATATATAAGAGCTTGCTGGCGCTTACGAATTCG

>Hist1h2ac_Hist1h2bc_M_musculus NM_178189-NM_023422 mm6_dna range=chr13:23163837-23164077 NM_178189 NM_023422

TACCAAGACTGTACAGACGCGGCAGCTTTTATAGTAGGTTCCTGGGTAGTAAACGCTACTACTTGATTGGTTACAAGTGAGCTCATTGATATAGCCAATAGAAAATCTGCTTCTGGAAACTTCATTTGCATAATCCCACCCATGAACCCACTACTTTTTCTTCTTCCAATTAGCTGAGACTGGTTCGAAATCCTTCATTAGCATAAACGCCCTATAAGTAGGAGAAGTCCGTTCCCAGCCC

>Hist1h2ae_Hist1h2bg_M_musculus NM_178187-NM_178196 mm6_dna range=chr13:23051289-23051465 NM_178187 NM_178196

TGATTGGTTAAAACTATCTTTCCTCTGCAGCCAATAAGGAAAGAGGTACAATACAATCTAATTTGGGTAATGGTGTCACTAACTGGCCTTCAAATCAGAGTTGACTACTTTTGAGTCCCTTATTTGCATAGAACCTCTATAAAATGGCAAGAACGAAGCTTCTCCCACATCTCATTT

>Hist1h2ag_Hist1h2bj_M_musculus NM_178186-NM_178198 mm6_dna range=chr13:21523014-21523295 NM_178186 NM_178198

GTGACAACTGTACGTTCCCAAAGAGCAAGAGCTTGGTCCTTATAGTTTTCTCTGGGCGCGAAAAGGAACGTTCGGGATTGGCTGCGTTCTTTTCATCTAGACCAATGGAAAGCGATTATAGCAACAAGTATTTTGATTGGGCCAAAGCCTATATGACGTCATCAAACCAATCACCCAATCATCATTTTGCTTTCCTAAGCTTTATTTGAATAAGGCCTCTTTAAAAGGGAAGAGCCGATCAGCTTCTGTGGTATAGCTCTACGTAGAGGTCCTGCTTCACTA

>Hist1h2an_Hist1h2bp_M_musculus NM_178184-NM_178202 mm6_dna range=chr13:21267283-21267580 NM_178184 NM_178202

TTCTAAACAAAAAGAAAGAACTGACAACTGCAAAGGCCAAAAGTGGAGGAGCTTGGCCTTTATAGGATTCTCCAAGCGCGAAAAAGAGCCTACGCGATTGGCTGGCTTCTTCTTTTCGTCTAGACCAATGGGAGGCGTTTATGCCGAAGCGTAGTTCCATTGGGCGCTACGCCATGAAAATTTGAAAATTTTAACCAATCAGAAATGTGTATCTCTAAGCCTCATTTGAATAACGCCTTATTAAAAAGCGAAGGGCTCACGGCGCAAGTTGTAGCGTGGTAGAGTTCCTGACCTAACA

>Hist1h2bf_Hist1h2ad_M_musculus NM_178195-NM_178188 mm6_dna range=chr13:23054259-23054540 NM_178195 NM_178188

TGGTTGGTGAGAATTTCTGGAACTTTCACGGAGGCAACTGGTCTTGTCTAGTTGATGAGGTCTTATTCAAATGAGGGATGTGAAAGGGCACCTTTGATTGGTTTATGACGGATGGCATACGAAGTCTTGCCCAATCAACGTGCGCCCCGAATGCTTCTCATTGGTCTAGGAAAAAAGACGTCAGCCAATGGCATAGGTCCTTTTTCGCGCCCAGCGAATAATATAAAGGCTACGTTCTTGTTTCAAAACCATTAGTTCTTAGTAACTGCTTAATCTGTACAA

>Hist1h2bl_Hist1h2ai_M_musculus NM_178199-NM_178182 mm6_dna range=chr13:21196208-21196487 NM_178199 NM_178182

TACTGAGAGGATGAAGTGAACTAAGTTGAAAAAGGATAACTAAAAGTTAATGACTGTTCTGGCTGCATTTTAAACAAACTTACGGCTATGCCGAACCTGAATCACCATACGTCATGTACTAACAGTCCAATCAAAACAAGGGATTTTCAAAACCAGGGCGCCATTGGTAACCAATGTGTAACCAATGAAATCTCTCCGTTTTCGCGTCCAGCCTTGACTATATATACTATGCGTATACCGTTTTTGCTTCTTACTGCGGTGGTTATCTACAGCTGAGTTA

>Hist1h2ak_Hist1h2bn_M_musculus NM_178183-NM_178201 mm6_dna range=chr13:21233892-21234188 NM_178183 NM_178201

TGGTAGAACCTAGAATATAAGCCTGCTCAGTAAGTGGTTTGAAGGCAAAGCTGACAGTATTTATAGTCAATGCAGGGCATGAAAAAGTAGGAATTTCATTGGTTACATAGAAGCATTGCACCTAGACCAATAACAGAGTTGCATTGAAAAGCCCTTGCTATGATTGGGTATTTGGTGGATGACGACTGCTAATTCGCGATAGCTATAGCCACAAGTTTGTTTAAAAGGCAGCCTGTGCAGACACACTTTACATTTCTTTTCTTTCTGTATCTTTAGGCTTTTGGTCAGTTCCTACCA

>Hist1h2bh_Hist1h3f_M_musculus NM_178197-NM_013548 mm6_dna range=chr13:23023417-23024030 NM_178197 NM_013548

TAGTGAACAGAGAATGAAGGTAGGCCAAGAAAAGAGATATTGAACTGTTCCGTTCCTTTCCTGCTCTTTAAATAAAGCGGTGGCTGTCCTTGTCAGAGAGTCTGCAAACGTCAAGCACTAACTGTCCAATCAAAGGAAGCATATTCGCTCAGTGGTTAGTTGCGTAAGTATCACGGAGAAGCGGACCGAACTTTACAGGGCTCTATTCAAATGAAGACTCGTTTCTTTCTTCTGATTGGCCAAGTTTAGCCAATAGACTCTATGAAAACTAGACGAAATAGTTTTTAGGAAAAAGTTGGTTTGATACTGTACTAAAGTCAAAGCCATTTTTCTTTTAAAAGTTATTTTGTTAGTAGTATCAATAAAAATCTCTCCCTAGATACAAAATTCATATATAAATTCTAGCTACATGTAAAGTTGGTTTCCTGTTTTGAAATTGTCTATGAAGAAAGATTAAGAATGGGAGACATCACATACTATTCACCTAGAGCTTCCAGAAATAATTATTGTACAGTCCGGCTAAAGTAGGCATGCTTTTCATTTGGCCCAAGAACCCTTAGAGGTACTGCCACGCTACAACAGTTGTGCAATTTCAATGAATCCTGTACATAGTT

>Hist1h2ah_Hist1h2bk_M_musculus NM_175659-NM_175665 mm6_dna range=chr13:21515617-21515952 NM_175659 NM_175665

TATTGTACAACTTAAAACTTCCCTGGTAAGCGTTAGCTAAACAGCAGAAACAAAGGGAAACTCTGTAGATTTTTATAGTCAAGCCAGGGCGCGAAAAAGGAGGCCCGTCATTGGTTAGCATTGTGAGTTCAACTTAACCAATGGAAACGAGGTTTTGGGAGTTGTGTATTCTCATTGGGTAAAAGCAGGCTCAGCCTTTTCTCTGAGTGACCACTACCCAGACAAGGGCATTTCACTTTATTTGCATACGAAGTTCTAGATAAAGAGTACCCGTGGTGTTCCTATCTGCTTGTCCTGTTGGGTGTTCAGCTTTGTTTTCTATTCATTTCTACGCCA

>Hist2h2ac_Hist2h2bb_M_musculus NM_175662-NM_175666 mm6_dna range=chr3:95708677-95708954 NM_175662 NM_175666

TAACTTAGAGTATAATGTAGGACACACCGAGGTGACAACTTCTGTAAAGCTAGGTTGATTCGTCTCTAAGGCCTTTATAGGCGCTCCTAGGGGTGGGGTGAGGAGGGAGCTTTTCATTGGTGGTTATTTGGCCCCTGAGAAGTCCAATGAAATCTGAAATTTTAGGCTTGTACTGAAAACGCAAGTTTCGCGTATGGGCCAATGGAAATTAAAAGTACTTTGAGACCTAATTTGCATAAGGCGTTCATAAAGGGCTCAAGTCCTCCGGGTCCCTGGTT

>H4.1_H3.1_IR_S_bayanus

TATTGTATTGTATTTGTTGTTGTTTGTTTTTGTTGTTATTTTAGTATCTATGTAACAGTATATAATAAGAGAACAAGAAAAACAAATCGGTAGGGGAAAACACGCCTGTATTTATAAGAAAGGAACAGGACCATTCCAACAAACAATCTACACACCATATAGGCGTTTTACGCTGTGAGCGGAGAACGGGGGTGGCAATTCACCACCGTATTCGCGGGCGTGTATGAAAGTAAACAACTTCGTTTTTTCCCGCCAACTATTATGGGGCGAAGCGGCTGGAACAGTTTAACTATGCGAAGATGCGAATTTCTCAGAACGCGGTTACTGTATTCGGGCGGTGCGATACAAAATGGTTTTGCCCTCGTGCTCACAACTTCGCATTGCCCCATACATTACCGTTCTCGCAACTTCGCACATTTCCTAGCTCAGCACCTTTGGATTCCTGGCCGTAATATCTCCCCTTGACTTTTAGCGTGGCAGATAACGAAAACGCCCGAGTGTTTTTCTCTACGGTTCTCTCCACGCCTCCTTGCTGCATCCCATATAAAAGAGAGTGTTTGTTCCTCGGAATACAGCTTTCTTCTTCTTCTTCTCATATACTTTTCCTCTTCCAATATATAAGAAAAGTATCGTACACACATATATAAACACAAGCAA

>H3.2_H4.2_IR_S_bayanus

TTGTAAAGTGTTTGTTTGTTTCTTCCAGTACAAAAGAGAAGTAAAGAGGAAGAAACTACAATCCAAGATTAAAACCAACACACTACAACACTTGTAAGAAAGGAAACAAGATTACTTATATACTTTTGTGGCCACGAGAGTTCTACGCGACGGCACGCCAACTTAGCACGCCATAACGCAACGATTGTGTTGGACTTTCGTCCCGTCATTACTGCTTGCACGACGCGCTGGGCGGTAATCTACGCCAAATTACCATTCCGAAACAGCCCCCTCTGGCGCGTTCCGGCAGTTTCACGCGCCCGATGCGAAGTGCCCGGAACGGGCAAGTGCGGTCGTTTGGCGCCGCGAAAGTGGTCGTGGACTGGGTGCTTCGCCTTTGTATGGCGAGACGTGCTGGGCGCTTCGCAGCTCCAACTTGTCCGGGCGCGAAAAGTAATGCGGACCAGAACAAAACAACACATACAGAAGGGTGTTTTAATATGCTGTTGATTCGCGCTTGCGTTGTTGCTATCCGCAGTATGTATCCGTGTGTGCGTGCCACCGGATTGGTATGCACATATATTTGTGTATGTATCTATATATATATATATGGAAGATTAGGTTGTATAGTGTTTTTTTCTCTTCTTCCTCTTAGATAGTTTCTTGTAAAATAGTTTTTTTGTACAACTCATCAAAAACAAAACAACAA

>H2A.2_H2B.2_IR_S_bayanus

TTATGTATTATATTTGTGTTTGTTTTGTTTTTGTTTACTTTATTACATTAAAGGATAAGAATTATATACAATTAAAATAAACATTGAATGTAGAAAACAATCAGAAAAAAGATCGGATAGCCAGAAGGAAAATCCAGGCATATAAAACTTCAAGTAAGAAATGTATAGCGGTTTCAAAAACGCTTAATCCTTCAACCTTATATAGAAATCAACTCCACTGTTAATGTTCATATTGACGCCAGTAGTTAACTCACAAAACAATAGAAAAAAACTAGCGTGTCGCGTTTATAGCCCTGCCATCATCTGTTCTATAAAATTCGCACCTTGTTGAGAAAAAATGGGAACGACTTTCGCGTTGGCTATGCCCACCGCTTCGCCTATCAACGCGCTGTTCTGGGAATTTCTCATCGTCCTCAGGTTACGAGCACTAACTAAGCTGCTGCAAGTCAAGTCGCGCTTGCAAAAATAGTCAACAAACGACGCGTAATGCAATTTCAGAAAGCCACTTGTGGTATATAAGGTAATAGAACTGGTGTGTCGTTGATCAAGGAAATTATTTCGGTGGTCTATATATACACATTTGTTAGTGTCAAAAGTATTTCTTTCTTGTTTATAGTTTGTTCAGTTTTTTTAGTCTTCTTCTTTTTCTTATAGAGTCTTTTCTACTTGGTTTCCATCAAAACCAACCACTCTCTATAGTAACAACACAAATA

>H2B.1_H2A.1_IR_S_bayanus

TATTTGTGTGGTTTGTTTGTTTGTGATTATGTTTACTTATATATTACAGGAAATAAATAATGTAAGAAGTAATATAATTAGAAAGAGAACAGATGTTGGTTGAACTTGATAAGCCAAGACAGTCAAACCCTGTTGTTTATATACATCAAAAAAAAATTGTAATGCCCCTTCTTTACTAGAACATAACAAGATAACCAACCAAGTTAGTAATTGCCACTATGAAGGCCAATTTGTCTTGATTTAAACCTGCCGTTCCCGGTTTTTCGCGTGGTACACCCTTTTGCCCGCGAAAAAACGGGAACAGCCTTCCCTTTCGGGCAATGGAGAGCGTCTGACCATACTTAACGACCCGACCGCGTTTTCTTGAAATTCAAACTCGCCGAGCTCACAAGTAACTCATTAGCGCTGTTCCAAGTTTTTCGCCCCGCTGTGCGAAGCCGTTGGCATGGACTGTTTTGGTGGCTCAGAAAGACAGCAGCATAGATAGGCCACCGTTGTGATAGAAACTCGCGTAGAGGTGCGTGCTTTCTCATGCTGTTTGTTGTTATTGCCCACCTTGTTGATTCCACACCAGTTTGCTGCCCCCCCTGTTCACGAAGCTAGTGATTGAACAAGAAGTTACGACTAAGTCTTGTTTGAAAAAAATATATAAATAAGGTGCTTAATTCTGTGATTAGTTTCTTGATCTGTCAACATCTCTTTCTATTTTCACTCCTTTCTTTGTTCTGTTGTGATACAATAACAGCATATCGATTTGATTATTTCTAAACAAATAAACAAACCAAAACCAAACAAATTTCATTCACACAAAATATATAA

>H3.2_H4.2_IR_S_cerevisiae

TTGTGGAGTGTTTGCTTGGATCCTTTAGTAAAAGGGGAAGAACAGTTGGAAGGGCCAAAGTGGAAGTCACAAAACAGTGGTCCTATATAAAAGAACAAGAAAAAGATTATTTATATACAACTGCGGTCACAAGAAGCAACGCGAGAGAGCACAACACGCTGTTATCACGCAAACTATGTTTTGACACCGAGCCATAGCCGTGATTGTGCGTCACATTGGGCGATAATGAACGCTAAATGACCAACTCCCATCCGTAGGAGCCCCTTAGGGCGTGCCAATAGTTTCACGCGCTTAATGCGAAGTGCTCGGAACGGACAACTGTGGTCGTTTGGCACCGGGAAAGTGGTACTAGACCGAGAGTTTCGCATTTGTATGGCAGGACGTTCTGGGAGCTTCGCGTCTCAAGCTTTTTCGGGCGCGAAATGCAGACCAGACCAGAACAAAACAACTGACAAGAAGGCGTTTAATTTAATATGTTGTTCACTCGCGCCTGGGCTGTTGTTATTCGGCTAGATACATACGTGTTTGTGCGTATGTAGTTATATCATATATAAGTATATTAGGATGAGGCGGTGAAAGAGATTTTTTTTTTTTCGCTTAATTTATTCTTTTCTCTATCTTTTTTCCTACATCTTGTTCAAAAGAGTAGCAAAAACAACAATCAATACAATAAAATAA

>H2A.2_H2B.2_IR_S_cerevisiae

TTATATATTAAATTTGCTCTTGTTCTGTACTTTCCTAATTCTTATGTAAAAAGACAAGAATTTATGATACTATTTAATAACAAAAAACTACCTAAGAAAAGCATCATGCAGTCGAAATTGAAATCGAAAAGTAAAACTTTAACGGAACATGTTTGAAATTCTAAGAAAGCATACATCTTCATCCCTTATATATAGAGTTATGTTTGATATTAGTAGTCATGTTGTAATCTCTGGCCTAAGTATACGTAACGAAAATGGTAGCACGTCGCGTTTATGGCCCCCAGGTTAATGTGTTCTCTGAAATTCGCATCACTTTGAGAAATAATGGGAACACCTTACGCGTGAGCTGTGCCCACCGCTTCGCCTAATAAAGCGGTGTTCTCAAAATTTCTCCCCGTTTTCAGGATCACGAGCGCCATCTAGTTCTGGTAAAATCGCGCTTACAAGAACAAAGAAAAGAAACATCGCGTAATGCAACAGTGAGACACTTGCCGTCATATATAAGGTTTTGGATCAGTAACCGTTATTTGAGCATAACACAGGTTTTTAAATATATTATTATATATCATGGTATATGTGTAAAATTTTTTTGCTGACTGGTTTTGTTTATTTATTTAGCTTTTTAAAAATTTTACTTTCTTCTTGTTAATTTTTTCTGATTGCTCTATACTCAAACCAACAACAACTTACTCTACAACTAA

>H2B.1_H2A.1_IR_S_cerevisiae

TTGTATGTGTGTATGGTTTATTTGTGGTTTGACTTGTCTATATAGGATAAATTTAATATAACAATAATCGAAAATGCGGAAAGAGAAACGTCTTTAATAAATCTGACCATCTGAGATGATCAAATCATGTTGTTTATATACATCAAGAAAACAGAGATGCCCCTTTCTTACCAATCGTTACAAGATAACCAACCAAGGTAGTATTTGCCACTACTAAGGCCAATTCTCTTGATTTTAAATCCATCGTTCTCATTTTTTCGCGGAAGAAAGGGTGCAACGCGCGAAAAAGTGAGAACAGCCTTCCCTTTCGGGCGACATTGAGCGTCTAACCATAGTTAACGACCCAACCGCGTTTTCTTCAAATTTGAACTCGCCGAGCTCACAAATAATTCATTAGCGCTGTTCCAAAATTTTCGCCTCACTGTGCGAAGCTATTGGAATGGAGTGTATTTGGTGGCTCAAAAAAAGAGCACAATAGTTAACTCGTCGTTGTTGAAGAAACGCCCGTAGAGATATGTGGTTTCTCATGCTGTTATTTGTTATTGCCCACTTTGTTGATTTCAAAATCTTTTCTCACCCCCTTCCCCGTTCACGAAGCCAGCCAGTGGATCGTAAATACTAGCAATAAGTCTTGACCTAAAAAATATATAAATAAGACTCCTAATCAGCTTGTAGATTTTCTGGTCTTGTTGAACCATCATCTATTTACTTCCAATCTGTACTTCTCTTCTTGATACTACATCATCATACGGATTTGGTTATTTCTCAGTGAATAAACAACTTCAAAACAAACAAATTTCATACATATAAAATATAAAA

>H4.1_H3.1_IR_S_cerevisiae

TATTTTACTATATTATATTTGTTGCTTGTTTTTGTTTGTTGCTTTAGTACTATAGAGTACAATAATGCGACGGAAACCATCATATAGAAAAAATATCTCGGTATTTATAGGAAAAAGAATTAGACCTTTTCCACAACCAATTTATCCATCAAATTGGTCTTTACCCAATGAATGGGGAAGGGGGGGTGGCAATTTACCACCGTATTCGCGGGCATTTGCTAAAGTAAACAACTTCGGTTTTTACCACTAACCATTATGGGGAGAAGCGCTCGGAACAGTTTTACTATGTGAAGATGCGAAGTTTTCAGAACGCGGTTTCCAAATTCGGCGGGGAGATACAAAAAAGATTTTTGCTCTCGTTCTCACATTTTCGCATTGTCCCATACATTATCGTTCTCACAATTTCTCACATTTCCTTGCTCTGCACCTTTGCGATCCTGGCCGTAATATCTCTCCTTGACTTTTAGCGTGGAAGATAACGAAATGCCCGGGCGATTTTTCTTTTTGGTACCCTCCACGGCTCCTTGTTGAAATACATATATAAAAGACTGTGTATTCTTCGGGATACATCTCTTTCCTCAACCTTTTATATTCTTTCTTTCTAGTTAATAAGAAAAACATCTAACATAAATATATAAACGCAAACAA

>H4.1_H3.1_IR_S_castellii

TATTATATTATTATTTGTTGTTGTTGTTGTTAAGCAAGAAAGGGGAAATAGAAAACAGAAAATGAAGAGGCAGCAATGAATAACACAGTTGCCATGTTATATAAGAATGACGAGATGACCAGATCCAGTGCCAAACTGTTTGTTTGTTTGTTGAAATCGCAAAGACGCGAAACGAAAAGGGAAACAGGCGCGAAAAAAGGGGCACGTTTGGCCATACTAAAGCGAATCTGCCAGCACGGGCTCAATTCCACTCAGATTGAGCCGTTCCAGAATTTTCGGTCTCTGTGGGTGAAATCTACGGTATGGATAGCCATCTTTCCCTGCGAAACTCCTGGTACGGATAGACACGTTTCTGTCACTCTCTTGTCGTTTGTGGTTTGCTGTTGATTGGTCGCTTGAGGCAAAGTATATATAAGGATCATCATTGCGGAACAACTTGACTACTTCTTTGAAATTATTCCCTTTTATTTTACATTTGCCTTTCAAATAAAGTGTCACACAACAAACCATACTACA

>H3.2_H4.2_IR_S_castellii

TATTATATTATTATTTGTTGTTGTTGTTGTTAAGCAAGAAAGGGGAAATAGAAAACAGAAAATGAAGAGGCAGCAATGAATAACACAGTTGCCATGTTATATAAGAATGACGAGATGACCAGATCCAGTGCCAAACTGTTTGTTTGTTTGTTGAAATCGCAAAGACGCGAAACGAAAAGGGAAACAGGCGCGAAAAAAGGGGCACGTTTGGCCATACTAAAGCGAATCTGCCAGCACGGGCTCAATTCCACTCAGATTGAGCCGTTCCAGAATTTTCGGTCTCTGTGGGTGAAATCTACGGTATGGATAGCCATCTTTCCCTGCGAAACTCCTGGTACGGATAGACACGTTTCTGTCACTCTCTTGTCGTTTGTGGTTTGCTGTTGATTGGTCGCTTGAGGCAAAGTATATATAAGGATCATCATTGCGGAACAACTTGACTACTTCTTTGAAATTATTCCCTTTTATTTTACATTTGCCTTTCAAATAAAGTGTCACACAACAAACCATACTACA

>H2A.2_H2B.2_IR_S_castellii

TATATTATATTTGTATTTGTGTGTGTGTTGAGTTGTTTGTTGTTGATGTTTAAAACTTAAACCAATGTAACTTAACTTGCTGTATCTGAAACAGACGAAAGAAAGAAAGATTTGAAGACTGATAATTCACTAGAGGAATTAAGATCGTAGTTTATATATCATTGGTAAGTTATTGTTGTTACAACATAGAGAAAGACAGACGAATTTTGTGGAATGGGGCCCTCAGCAATTTCTTCGCGGTGTTCCCCTTTTTTTCCTTGCCATGCTCTGACGTTCGCCCCCCAGTGTGAAATTTTTGGAACGCCGAATACAAGTTTGTTTTCATCGCAAGAAGATCAATTTTGAAAAGAACGCGCTATCGCTCTTAACTATGGTCAGACGCCCTGTAGCCCGAAAGGGAAATTAATTTTCGCCCTGTCATGCCAGAAGTTTCGCGCGAGGTGATCATGTTCGCCGCGACAAAGTGTGAAACTTGTGGAACGGGACTGTCTCATCCTGTAAATTGAGGGAGAAACCCGTTAGTGCACAAAACTATCTATCAACACCGACACGATGGCATGTCCTAAATAACTTCCTTTCATATATAAATGCGTAATATATTCTTGTCATCACCGTGTTTCTTCTCATAAATTATTCTTTCCCTAATATTCGGATAGGAAACTAATATTTATATTCCCAAACACAACTAACACACAAATCAATA

>H2B.1_H2A.1_IR_S_castellii

TTATTGATTTGTGTGTTAGTTGTGTTTGGGAATATAAATATTAGTTTCCTATCCGAATATTAGGGAAAGAATAATTTATGAGAAGAAACACGGTGATGACAAGAATATATTACGCATTTATATATGAAAGGAAGTTATTTAGGACATGCCATCGTGTCGGTGTTGATAGATAGTTTTGTGCACTAACGGGTTTCTCCCTCAATTTACAGGATGAGACAGTCCCGTTCCACAAGTTTCACACTTTGTCGCGGCGAACATGATCACCTCGCGCGAAACTTCTGGCATGACAGGGCGAAAATTAATTTCCCTTTCGGGCTACAGGGCGTCTGACCATAGTTAAGAGCGATAGCGCGTTCTTTTCAAAATTGATCTTCTTGCGATGAAAACAAACTTGTATTCGGCGTTCCAAAAATTTCACACTGGGGGGCGAACGTCAGAGCATGGCAAGGAAAAAAAGGGGAACACCGCGAAGAAATTGCTGAGGGCCCCATTCCACAAAATTCGTCTGTCTTTCTCTATGTTGTAACAACAATAACTTACCAATGATATATAAACTACGATCTTAATTCCTCTAGTGAATTATCAGTCTTCAAATCTTTCTTTCTTTCGTCTGTTTCAGATACAGCAAGTTAAGTTACATTGGTTTAAGTTTTAAACATCAACAACAAACAACTCAACACACACACAAATACAAATATAATATAA

>H4.1_H3.1_IR_S_kluyveri

TATTGATTGTGTGTTTTGTGTGTGTGTGTGTGTGTGTGTGTGTTGAATGAAGAGAAATAAGAATCTTCTGGCGGGTTAAAAGAAGAAAAAACGAGAGCAAAAACAAGGGGCGAACGAGACACGGAAGGAAGGCGAAAAAGAGCTTTCTTATATACAGGTTGCACGGTGGCCAAAGACAGCGGCAGGTTAGTATGGCTGTTTGCGTTCCTGTTTTTTCTCGCGCAGAGCGAGATTGCATGCGAAGAAACTGGTCCGGGTCGATGCATAGTTTCCGTATACGGCAATGAGTTCTGGCGCGTTCGCGCCTGAGAGCCGCGCACGCGAAAAAACCGGTCTGCAGATAGTGCGCGCGGCGGGCGTTTTCCTCCCAGCGGCGCGTGCGCGGACCAGCTTTTTCCCGGCGCGCCGTGCCCGCAGCTTCGCACCCCTTGCCCACCGCGCAAACTGCCGCATTTGGCGTGCTTCCGGCACTGCCCTTCATTACTGTGTCTCGCGCTGCGGTCTCGCTCTGCGGCCTCGCAAACTAAATTGCTCTGCAGACCCACACATCCGCCGCCGTTGCCGCTCCCGGCTATATAAACTCGTCGTCCCTGCTGTTTTCGCCTCTTCTTCCCTTTACTTCTCTTTCTCTTTTGCTCTTCCTCTCTCTCCCGCTCACTCCAAACGTCAAACAATA

>H3.2_H4.2_IR_S_kluyveri

TATTGTAATTAGTTTATTTTATTTGTTTGTTGATGAGAGAACAGGAAAAACTAAAAAGAAGACTACTCAACTGAATATTTACAATCGATCCAAGCCGTCTGTTCTTATATACCCATCCAAGTGTCCTCCCACCACGGCATAAAAACTTTGTTTTTGCTGCGACGCAAACACGAAACACGAAAGACCCCGTAGCGGCTACGCGTAATCACTGGTCCATAGCCCGTTCCGAAATTTTCACAGTACGTTTTCCCCGTTTTTGCTGGGAAATTTATGGTCCGGAACCCATTCCCACTTTTTCCCGAATAAATCGCAGCCGTGTCTTGTGCGAATAAACTGGGATGACGGCCGTTCTCAGTGGTTCGCAGCTCTATGGTGTTTTTTTCGCGCTTCCTTTTTCCTTCTTTTTTTACACGATGGCAACCACGTAAACGCTACGGAACCTATCCCTTGCCGAAATCAGCTGCGAAAACATATTTAAACAGACACCAACCTCATTAGACTGCTCTAAACAACTTTATCTGTTTTCTCTCTTGTATCATACAAATCATCCACTACACACATAAACACACACAACCAACA

>H2A.2_H2B.2_IR_S_kluyveri

TATTTCTTAATATAGTATTAGTTTCTGTTAAAGATATGCAAAAATTTGCGGCTGGTAAATCCAGGAATTATCAGTAACTTTCTAACATATCTGCTACCTGTTTATATATTTATCATTATTTTACTGGCAAACGATATAACGCGACATGTCTTGCGCGTGCCAGTTTCTTCGCGGGAGAGAATAAAAAGCGCGAAAAATCTGGTACAGTGGGTATTTTTTGGCACAGCCTTAAGACCGATTTTTTCGCGGTCGACCTCCCTGCGGAAATTTCTTAGACAGATGTGAAATCTATGGCACACATGGCGTTCCAAAGTTTTCGCCAAATAGCAATTCGTGTCTCTCATCCCCTTCTGCGTGGGGACGCGTATCGATATGTGTGCATCTGTCAATTGAAAAGATATATAAATGAGAAATACATGATTGCTCAAGAATTGATTTATAATTGACTTTGTGTTCTGTTGCTAATACCACCCAATAATAACTTTATTTGACGAAA

>H2B.1_H2A.1_IR_S_kluyveri

TTATTTCTTAATATAGTATTAGTTTCTGTTAAAGATATGCAAAAATTTGCGGCTGGTAAATCCAGGAATTATCAGTAACTTTCTAACATATCTGCTACCTGTTTATATATTTATCATTATTTTACTGGCAAACGATATAACGCGACATGTCTTGCGCGTGCCAGTTTCTTCGCGGGAGAGAATAAAAAGCGCGAAAAATCTGGTACAGTGGGTATTTTTTGGCACAGCCTTAAGACCGATTTTTTCGCGGTCGACCTCCCTGCGGAAATTTCTTAGACAGATGTGAAATCTATGGCACACATGGCGTTCCAAAGTTTTCGCCAAATAGCAATTCGTGTCTCTCATCCCCTTCTGCGTGGGGACGCGTATCGATATGTGTGCATCTGTCAATTGAAAAGATATATAAATGAGAAATACATGATTGCTCAAGAATTGATTTATAATTGACTTTGTGTTCTGTTGCTAATACCACCCAATAATAACTTTATTTGACGAAAA

>H4.1_H3.1_IR_S_kudriavzevii

TATTGTATTATATTAGTTATTGTTTATTGTTTATTTGCTGTTTTAGTATAAATAACGGTTAGAAGTGAAGAGACGAAAAAGAGCTTCCTGTAAGAAAAATTACCGCCGTATTTATAAGAAAAGAATAGAGACATTTCGCACAAGCAATTTACCTACCATGTAGGCGTTTTACGTTATGAGCGGAGAACGGGGGGTGGCAATTTACCACCGTATTCGCGAGTGTTCATCAATCTAACAACTTCGTTTTTCCCCGGTAGCCATTATGGGCGAAGCGCTCGGAACAGTTTTATTATGCGAAGATGCGAATTTTTCAGAACGCGGTTACCGATTCGGTGGAACGATACAAAGTGATTTTGTTCGCGTTCTCACAACTTCGCAATGTTCCATACATTATCGTTCCCACAATTTCGCACTTTTCCTTGCTCAGCATCTGTGGGATCCTGGCCGTAATATCTCCCCTTGACTTTTAGCGTGGGAGATACGAAAACGTCCGAGTATTTTTCTTTATGGTTAAATCGACGGCTCCTTGCCCGCATATCATATATAAGAGATCGTTTATTAGTCGGAATACAAAATTTCCTTCCTCTTCTTCATGCCTTTCTTCCAGAGAAGTACAAAAAGGTAACGAACGTAAATATATAAACACAAACAA

>H3.2_H4.2_IR_S_kudriavzevii

TATATTATTGTTTTGTTTTTAGCTGTTGATTACTTCTTTGTTAATGCTACAGGAAACAAATAAGATGTTTCTGAGGGATAGAATATTGAAAGAACGAAAAAGCAATATTTACAACTCCACCCGTACGTGTTTATATATATTATATTTTGTACACGCAAACGCACGCAAACACACGAACATATGGCCCCGAATAGCAGCAACCCAAACGCGAATCAACAACGTATTAAGCACCCTTCCAACAGCTGTTCTGGGCCGCTTCGCGTTTCGCGCCAGAGATATGAAGATGCGAAGCTTTTAGAACGTCCTGCCATACAAATGCGAAACACCCGGTCCAGGACCTCTTTCCAAGCGCCAAACGATCATATTTGCACGTTCCGGGCACTTCGCATGATGCGGGTGAAGCTGCCGGAACGCGCCGAGGGGTTACTACGGGGATAGGATATCTGGCGTCGATTACCGCCC

>H2A.2_H2B.2_IR_S_kudriavzevii

TATATATTATATTTGCGTTTGTTTTGTGCTTATTTGCCTCTACATCAAAAACAAGAAATGTATAGAAATATCTAAATGTAGGAAACAAATGCAAGAAAAATATGTCGTAACCGAAAGCGAGAGGAAGTAAAGGAGACTTCAATTGAGGGAACACTTAAGCTTTTGAAAAGAGCACACTTCTCCATCCTTTATATAGAACCTATCTTCATTGTCGCTACTTACATCGAAGCCTATATATTTAAACCGCAGAACAATCGAAAATAGGCAGCGTGTCGCGACTACGGTTGAAGTTCATCTGTTCTATAAAATTCGCACCACTCCAAGAAATAATGGGAACGCTTTCCGCGTGGTCTGTGCCCAACGCTTCGCCTAATAACGTGCTGTTCTCAAAATTTCGCCTATCCTCGGGATCACAAGTATAATGAGCTTCTGGTAAATCGCGTTTCCCAAAAGAAGCCATCAAGCAACGCGTTAAACAATATGGGAACGTCGTTTGCCATATATAAGCATACTGAATTGATGTGCTGTTATTGAAAAGTTCTAATTATCTTTTAAGAGCAAGTGTAGTTTAGGGTTTGAGCAACTTATCTTGTCTAATTTGTCCTTGTTTTCTCAGTACGTTCAGACTTTTTCATTTAAGATAATCTAACTTCTGTGTACATAAACCAAGAACTTCCCTTAAAAGTGATATAAAT

>H2B.1_H2A.1_IR_S_kudriavzevii

TTGTATATTTGTGTGGTTTGTTTGTGGTTTTGTTTAGTTATTTAAATTAAGAAATATTCCATAAATAAGATTAAAGGTACAAGGAAAGGAACATTCGGTAGTTGAACTCGAACATCCAAGACAGCCAAACCCTCTTGTTTATATACATCAGAAAATCTAAGATGCCCCTTCATTACCAAGACATTACAAGATAACCACACCAAGGTAGTAATTGCCACTACAAAGGCAGTTCTCTTAATTTAAAACCGCCGTTCTCATTTTTTCGCTGGCAAAAGGGTGCAGCACGCGAAAAAATGAGAACAGCTATCCCTTTCGGGCGATGGAGAGCGTCTGACCATAGTTAACGACCCAACCGCGTTTTCTTGAAATTTGAACTCGGCGAGTTCACATGTAACTCATTAGCGGCGTTCCAGATTTTTCGCCCGGCTGTGCGAAGCTATTGGTATGGATTGTATTGGTGGCTCAAGAAAACAGCAATACAGATAGTTCTTCGCCGTTAGAGAAACCCGCGTAGGGATGCGTGGTTTCCCGTGCTATTACTTACTATTGCCCACTTGTTGATTTTCAAAACTAAGTTGTTGTCGCCCCCTTGTTTACGAAACTAATCAGTGGATCAAATATTATCAGTAAGTCTTACCTGAAGGAAAAATATATAAATAAGGCGCTTGATCCCGTAGTTGCTTTTCTGATCTTGTGGAACACTCTTCCCATTTCTCTTTATTTTCTACTCTGATACTACAACAGCATTCAGATTTGATTATTTCTAAGCAAATAAACAAATTTAAAACAAACAAATTTCATCTATATAAAATAAAA

>H4.1_H3.1_IR_S_mikatae

TATTTTATTATATTGTATTGATTGTTTTTATTTGTTGCTTTGTTATGATTACAGGGTAAAGAAAATAAGACAACGAGCATAATGATCCAGTGAACAAAACATGTCCGTATTTATAAGAAAAGGATTGGCTAATTTCCCATGATCAATTTACCCATCAAGCAGGCTTTTTACTCAGTAAACGGAGAACGGGGGTGGCAATTTTACCACCGTATTCGCGCCATTTGCCAAAGTAAACAACTTCAGTTTCTACTATTAGCCATTGTGGGGTGAAGCGCTCGGAACAGTTTTACTATGGGAAGATGCGAATTTTTCAGAACGCGGTTACCAAATTCGGCGGGCAGTTAAAGGTGGTTTTGCTCCCGTTCTCATAACTTCGCACTGTCCCATACATTATCGTTCCCACAATTTCTCACTTTTCCTTGCTTAACACCTCTGGGATCCTGGCCGTAATATCTCCCCTTGACTTTTAGCGTGGAAGATAACGAGAATGCCCGGAGGATTTTCTTCTTGGTCCACTCTACGGTACTTTGTTGAAAACCATATATAAGAGACGGTGTATTCTTCGGGATGCAACTCTTTCTTCCTTTTCTTGCTTCACTTCCTACTATTCACTATAAAGAATCATCAAACATAAATACATAAACACAAATAA

>H3.2_H4.2_IR_S_mikatae

TTTCGTAGTACTCTACTATCCTATTTTGTTAAATAAAGGAAAAAAAAAGAAGAAATCTTAATATTCCTAAAAGAAAAGATTACTACAACTACTATTCTGTGTGAGAATGGGAAAAGAGATTACTTATATACGGTTGCGGTCACAAGAAGCTGCGCGCGACAGTTCAACATGCCGTTATCACGCTAACGACATTCTACCACAGAAATCATAAACATAGTTGCGCGACGCGCTGGGCGGTAATTGATGCTAATGACAGCCTCCTGTAGAAGCCCCTTAGCGTGTTCCAGTAGCTTCACGCGCCTAATGCGAAGTGCTCGGAACGGACAAGTGTAGTCGTTTGACACCGAATAAGTTGTCCTGGACCAAGCGTTTCGCATTTGTATGGCAGGACGTTCTGGGAATTTCGCATCTCCAGCTCTCCGGGCGCGAAATGTAAAGCGGACCAGAACAAAACAACTGACAAGAGGGTGTTTAGTTTAATGCGTTGTGCATTCGCGTTTTGGTTGGTGACATTCCGTCACATAGGTACGTGTTTGTGTGTACCTATTTATATTATATTTCAATATATATAGGAGGATGTGTTATTACCGGTTTTTCTACAGCCTGCTATGTTGTCTTTATCTCTAAATTATTTTATTATTGTAGTTTAGTAATATAAACAAACAAAACAAATACAAA

>H2A.2_H2B.2_IR_S_mikatae

TTATATTTGCGTTTGTTGTTCTTGATTAATTTTGTATTTAAGAAGACAAGATATGTATAAAATATCTAAATATCAAACACAGCTATAAGAAAAGGGATATTTAGTCAAAGATGGAAAGTAAAAAATTAAAGGAGATACCGTCGTAAACACTTGAAGAGTTAGGACAAAGTACTCTTTCCCATCCCTTATATAGAATTACTTCCACTTTCAAGAGCCTTATTGAAGTCTGTAGCTACTAGACTATAGACAGAATAGAAAATGAGTAGCGTGTCGCGTTTATGGCCCCAGATTCATGTGTTCTCTAAAATTCGCATCGCTTAGAGAAATAATTGGAACACCTTACGCGTGAGCTGTGCCCACCGCTTCGCCTAATAAATCACTGTTCCCAGAATTTCACCCCAAACTCATGGTCACGAGCGCCATTGTGCTCCGGTAAATCGCGTTTACGAGTACAACCAACAAACAACAACGCGTAATGCAATAATAGAAGGTCTGTTGTCCTATATAAGGTTATAGGATAGGCCGCCTTTAAATACAGAACTCCTAACAGTTTATATGTGAATATAAATATTAATATTTATATATATACGAATTTGCGTTGAAGTAATTTCTTTTTTCGTTTTTCTTGATACTTAGAATACTCTTTTATCCTTGTCTATTCTATTTTCATCTGTGCAGATACAAACCAACAACAGCATTCTCTACAAATAA

>H2B.1_H2A.1_IR_S_mikatae

TTATATATGTGTGTGTGTGTGTGTGTGTGTTTGTTTGTGGTTTAACTTATCTGTGTTGATTAAAAGATATTCAATAAAAATCTGATTCGTAGAAGAGAGAACATATATATTAATCAAATTTTGTCACCCAAGATATCAAAACCTTCATGTTTATATACATCAGAAACTAAGAAGGCCCCTTTCTTACCAAACATTACTAGATAATCAACTAAAGTAATAATTGCCACTATTAGGGCTACTTCTCTTCATTTAAATTCATCGTTCTCCTTTTTTCGCGCGGTGTACCCTTCTGTACGCGAAAAAAGGAGAACGGGCTTCCCTTTCGGGCGATTAAGAGCGTCTAACCATAGTTAACGACCTAACCGCGTTTTCTTTAAATTCAAACTCGCCGAGCTCACAAGTAACTCATTAGCGCTGTTCCAAAACTTTCGCCCCACTGTGCGAAGCTATTGGAATGGATTGTATTTGGTGGCTCAAAAAAAACGGTAGAATATAGCTTGTCGTTGTTAGAGAAACCCGCGTAGGGAAGTGTGGTTTCCCATGCTGTTGCTTGCTATTGCCCACCTTGTTGATTTTAGAACGAGTTATTGTCCCCCCTGTTTGCATAACTGTTCGATGAATTCCGCATGTTAATACTAGTGGGCCTTGTTTAAAAAATATATAAATAAGGTGCTTGATTTAGTTGTTGACTATCTTGTACATCTACCTATTTCTCTTTTCCTCCTATTTCTAAGTTCTATCTTGATACTATAGCATCATACAAATTTGATTATTTCTAAACAAATAAATAAATTTAAAACAGATAAATTTCATACAAATAAAATATAAAA

>H4.1_H3.1_IR_S_paradoxus

TATTATATTATATTTATTGCTTGTTTTTGCTTGTTGCTTTTGTTTTATAGAATAGAAATATGAAAGAAGGCGACGAAAATCATCCCATAGAAAAAAGACGTCTGTATTTATAAGAAAAGGGATTGTGCCATTACCACAGCCAATTTACCCATCAAACAAGTTTTTTACCCAATGAATGGAGAAGGGGGTGACAATTTACCACCGTATTCGCGGGCATTAGCCAAAGTAAACAACTTCGGTTTTTACCACTAACCATTGTGGGGAGAAGCGCTCGGAACAGTTTTACTATGTGAAGATGCGAATTTTTCAGAACGCGGTTACCGAATTCGGCGGGGGGATACAAAAGAGGTTTTTTCTCATTCTCACAATTTCGCATTGTCCCATACATTATCGTTCCCACAATTTCTCACATTTCCTTGCTCAGCACCTTTGGGAACCCGACCGTAATATCTCTCCTTGACTTTTAGCGTGGGAGATAACGAAAATGCCCGGGCGTTTTTTCTTTTTGATACACTCCACGGCTCCTTGCTGAAATACATATATAAAAGACCGTATATTCCTCGGGATGAAACACTTTCTTCCCTCTTCTCATATACTTTCCTTTAGTTAGTAAAAAAGGCATCGAACATAGACATTTAAACACAAACAA

>H3.2_H4.2_IR_S_paradoxus

TTACGGAGTGTTTGCTTAGTTTCTTCAGTACAAAAGAAAAAGTACAAAAGTAAAAGATTTAAAGTGAAAGTAACAAAGCAGTGGCTCTATATAAAAACAGGAAAAGAGATTACTTATATATAATTGCGGCCACAAGAAGCAACGCGGAGACAGCACAACACGCTGTTATCACGCCAACGACGTTTTGGCACCGAGCCATAGCCATAGTTGCGCGACACGCTGGGTGGTAATCGACGCTAGATGACCCACTGCCGTAGACGCCCCTTAGGGCGTGCCAAAGGTTTCACGCGCTTAATGCGAAGTGTTCGGAACGGGCAACTGTGGTCGTTTGGCGCCGGGAAAGTGGTCTTGGTCCGAGAGTTTCGCATTTGTATGGCAGGACGTTCTGAGAGCTTCGCATCTCCAGCTTTTCCGGGCGCGAAATGCAGAACGGACCAGAACAAAACAACTGGTAAGAGGGCGTTTAGTTTAATATGTTGTTCATTCGCGCTTAGACTGCAGCTATTCGGCTACATACGTACGTGTTTGTGCATATCTAGGTATGTCATATATAAGTATATGTAGGATGAGGTAGTGAAAAGTCTTTTTTTTCTCGCTTCGTTTGTTCTTTTCACTGTTTTCTTTTCTTGTTTTTGCCACAAGACAGCAATAAAACAACAACAATCAATACAATACAATAA

>H2A.2_H2B.2_IR_S_paradoxus

TTATATATTAAATTTGTTCTTGTTCTGTACTTGTTTAACTTTCTTATGTAAAAAGACAAGAATGTTATGATAGTATCAAAAGATCATACAGGGAAAGCATCATACAGTCGAAACCAAGAGCGGAAACGAAACTCTGTTCGGAAGATACTTGAGACCCTAGAAAAAAGCATACATCTTCATCCCTTATATAGAGTTATGTTTACTATCAGTAGTCGTGTTGGAAGTTGTGGCCGAAGGTATAAGCAATAAAAATGAGTAGCGTGTCGCGTTTATGGCTCCAGCTCCATGTGTTCTCTAAAATTCGCATCACTTCGAGAAATAATGGGAACACCTTACGCGTGAGCTGTGCCCACCGCTTCGCCTGATGAAGCGGTGTTCTCAAAATTTCACCCCATTGTCAGGATCACGACCGCCATTTGGTTCTGGTAAATCGCGCTTATGAGAATAACGAAAAAGAAACAACGCGTAATGCAATAGTAGAAGACCTGTTGCCATATATAAGGTTATAGAATCAGTGGCCGTTCCTTGAGGATAACCCGTATACTTGAATTTATATATATTATATTATAGGTGTATAATTTTGCGTTAGCGTAATTTTTCTGTTTATATCTTTTTCGTATCTTAAAGTTTCCCTTATTCTTGTTTGATTTTTACTGATTCGTTTATACACAAACCAACAACTTACTATACAAATAA

>H2B.1_H2A.1_IR_S_paradoxus

TTATATGTGTGTATGGTTTGTTTGTGGTTTCGCTTGTTTACGTAGGAGATATATACTACGAAAAAATTTAAAATGTAAATAAAAGAGACGTATATTTAACAAAACAAATCATCCGAGATGATCGAACCCTGTTGTTTATATACATCAAAAAAACTGAGATGCCCCTTTCTTCTAAACGTTACAAGATAACAAACCAAAGTAGTATTTGCCACTACTAAGGCTAATTCTCTTGATTTATCTCCATCGTTCTCCTTTTTTCGCGCGCTGCACCCTTTCACCCGCGAAAAAAAGAGAACAGGCTTCCCTTTCGGGCGACGAAGAGCGTCTAACCATAGTTAACGACCCAACCGCGTTTTCTTCAAATTTGAACTCGCCGAGCTCACAAGTAATTCATTTGCGCTGTTCCAAAATTTTCGCCCCACTGTGCGAAACTATTGGAATGGATTGTATTTGGTGGCTCAAAAAACAGCAAAATAGTTAACTAATTGTCACTGAAGAAACGCGCGTAGGGATATATGGTTTCTCGTGCTGTTACTCGTTATTGCTCACCTTATTGACTTCAAAACCTTTTCTCCCCCCTTCTCCGTTCACGAAACCAGTCAGTGAATTGTTAATGTTAGCAGTAGGTCTTGGTCTAAAAAAAATATATAAATAAGAGGCTTGATCTGTTTGTAGATTTTCTGACCTTGTTTAACTATCCTCTTATTTTTGCTTTCATTCTTACATTCTCTCTTGATATTACAACGTCATACAGATTTGGTTATTTCCACGCAAATAAACAAGTTCAAAACAAACAAATTTCATACATATAATATAAAA

>H4.1_H3.1_IR_S_pombe

GTTAAGAAGTAGAAAAAATTAAAATCTACGCAAAAAAACGCGTAATTCTTTAAGTATAATAAAGATAAAGGGGTTTTCCGTTGACTAACAAAGGAACAATGAATTTATAGATGTCAACAGATGTTAACGGTAGGATAAAAGTCAATGCAAATTGACAAAGGATTTTATCCGTATTCAAAAACAATTAAAAGAAATGATTGTCTGAAGAGAGGGAAGTTGGGGCGACGCTGTCTATTTGTTTTGAATCTGCATCAAAACCCTAACCCTGCTTTTGATGAGATCGGGTGGATTCAAAACATATATATAGCAAATATCTGCAGTACGCTTGCGTTTCCATTAATTCTAAAGATCAACAATTGGCAAAAGTAGCACAACAGCTATTTTTTTCTCGATTGTCTTTTTATTTGATATTCATTCTACTAGCTTGATATA

>H3.2_H4.2_IR_S_pombe

TCTTATATGAGTAATGGGATTCCTGTAAGTTCAAACAAACTGACTACCAATTGATCAATTTACAATATCAAAAACTAATTTTCAAGCGCAAACACTGAAATTCCTATGATTTGAATTTCAATGGCTTCTCGTACTAGTCGTAGTAGTTACTGTGTAAGTGCTGTAGTTAGGCGTCCATCTCCATATATATATACATTTTTTCAGGTAGCGGGGAAGCCGAAATCGCAATCTTAAATCAGGGTTAGGGTTGTGATTGACTGAGGTATATATAGCAGGAAGTGTCCACACCCGACGTGGAAAGAACCTTTTTGTAAGTTTATTTACCGAATTACGTTA

>H2A.2_H2B.2_IR_S_pombe

GACGAAAAATACTGAAACTAAATAAAAAAAATAAAAGTTTGAGTTAAAAAAAAAAGATTTTTCCTTCTTTAATGAAAAAAGATAACAGTCAACGATGAAGCAGCGAATGGATCAAATTTGGTGAAATTAACAGAATTTTCTCGCAATTTCAGCCTTTACGGACAATCAATCTCCTTATCAACTAAGGATCAATTGTTTATATCAATTTCAATACGTGTATGGATCAAAAGGTATCCTAAACAACGAAAAGTCGCGATATAGGGAAAAGGAGACATAGGAGCGCGCACCCTTCATATATATACTAATTCTAATCTTCCGTATAAGCACGTCTAACGCGTTCTTGTAGATGACGTTTCCGCGTCGCATGGCTAAACACCAGGGTTAGGGTTGTGATGCCCAAGTTGTATATAACCATCGCGTCGCCTGCAGGCATGTTAGTTAATACCAACAACTTGATATTGCACGAAAGTTTAGGATTATCAAATTCCTACAATTGCAATTACTTAGTTCAGTCCATCAATTTTGAGATTCTCTCGACAACCAATTTTTAAATCGTAAAAAATG

>H2B.1_H2A.1_IR_S_pombe

TGACGAAAAATACTGAAACTAAATAAAAAAAATAAAAGTTTGAGTTAAAAAAAAAAGATTTTTCCTTCTTTAATGAAAAAAGATAACAGTCAACGATGAAGCAGCGAATGGATCAAATTTGGTGAAATTAACAGAATTTTCTCGCAATTTCAGCCTTTACGGACAATCAATCTCCTTATCAACTAAGGATCAATTGTTTATATCAATTTCAATACGTGTATGGATCAAAAGGTATCCTAAACAACGAAAAGTCGCGATATAGGGAAAAGGAGACATAGGAGCGCGCACCCTTCATATATATACTAATTCTAATCTTCCGTATAAGCACGTCTAACGCGTTCTTGTAGATGACGTTTCCGCGTCGCATGGCTAAACACCAGGGTTAGGGTTGTGATGCCCAAGTTGTATATAACCATCGCGTCGCCTGCAGGCATGTTAGTTAATACCAACAACTTGATATTGCACGAAAGTTTAGGATTATCAAATTCCTACAATTGCAATTACTTAGTTCAGTCCATCAATTTTGAGATTCTCTCGACAACCAATTTTTAAATCGTAAAAA

>HIST1H4L_H_sapiens hg17_dna range=chr6:27949268-27950267 NM_003546 NP_003537 H4 1 bp downstream - 999 bp upstream -

GTCTTGTACTAAACAAAATGCAACACGGCAAGCCTCGCAGCAGTACTTTATATAGTAATAAATCGGACCTGATTGGGAACTCTCCACACCAGCTGCATCCGGTCGGCCGATTCACAGACCTTGCGCCCTGCCCACGACTTCAGGAAAAAATGGAAAAGGGGGTACTTAAACTCCAGTTTCTAAAAATGATGCCCAGTTAAAAATTACTCAGCTCATCTTGCCCTTCTACAGTGTTTTAGGCTGACCACCGCTGCATTCCATTGTCTGGGTCTTTGCAGCCTGTTTTTCCTGTCACCTCTTAGCATCGATTGTTCTAGTGCTTTGTGGCTGCTGGAATCAAATGGCGCCATCTCTAGAGGGGTAATCACAGTCGTTTCGGGCATTTGTGAAATAGCGTGTAGAAACTGCTTCCGTACAAACTCGAAGTGATCAGCCTATGGAGTGGCTGACTGCAAAAGACGACGCCTGATGATAGCTCCAAGGTGGGATTCCTCACCTTTCTCAAATTTGGTTCTCTATTCTTTTCCTTATATTTTACTCTTTCTGTAGCTGTCCTTTCTGTTCCTTTCTCTCCCTCGCCAACCACATTTTTGGGGCAAGAACTTTCTATTTTTTTTTCCTCATTCCACCTAGAAACACTTCAGGAAGCTGTTTGAGAAAGAAAGGTGGCTTTTACCCTGTATTTCCTTAGAATTGTGAAGGTGCCGCTTGAGCTCCCTCATGTTCCTCTTTTCTGTTGAACTAGGAAATCAGCTGTGCCCAGAAACCCTGTCTTTTACATTTACAAATAAAGTGCAGGAGGAAAAGACCGAGGATGACTTTATTCCAAGAAAAAGAGTTTCAAATGGCCGGACGCGGTGGCTCACGCCTGTAATCCCAGCCAGAAGGGAGGCCGATGCATGTGGATCACAAGGTCAGGAGTTCGAGACCAGCCTGGCTAAGATGGCGAAACCCCTTCTCTACTAAAAATAAAGAAAATTGGCCTGGCGTGGTGGAGGGC

>HIST1H4K_H_sapiens hg17_dna range=chr6:27907284-27908283 NM_003541 NP_003532 H4 1 bp downstream - 999 bp upstream -

TGACGAGCAAGAGGAGTCTCACCCAACGCTTTGTGAGGACTCTGGCCTGAGGCAGCGCCTTTATACGACAGTTGGCGGACCGAACTGAGAACCTGAAAGAAGTCGGCGGGAAGTCCCGCCCCGGTGGGGGAGGGGAAATCTAAAGGGCCAAACCGAAATAGGGGGAAAAAAAAAGCGAGCTTCTTGTTTCCGTGTTCTGAATTTTGTAACGTGCATAGTATTTTGTTACCACGTTATGAGGCTTTAAAAAATTGCTTTTGAACGCAGAAGATATACATCAATACTGTGGGAAATACAAGAAAGGACAAGAAATTAAGAAACTACAATGTTATCCCATCACACAGGCTAGTTAATCATGTATTTTGCAGAGCAGTTGCACATATTTTTCCAAGAAAATGTATACAGTGTTGTATATGGAGTTTTGTAACCTCCTTATATTGATTATAATTTAACCAATTTCTATTAAAGAGATAAAAGTGATGTTTTGGTGTCTATGTTTCTTAGGAATTATCAATAGTTATAATCAGTTCCCCAGCAATTTTTTAATCGGCTGTATTTTAAAAATAATGTTTTCCACATTCAACATAAATGTACTTTTTCTCTATACTTGGGACCAATATTGAAATTTATGATTTTATTACACCAAAATTTAAATTTTATTACATTAATATTTAAAATTGTATTAGAGGTCTCATGATTTGGTACTACGGGTCTCCGCATTATTTCCTTTCCAAATTTCCTAATCTGTTTCACCAAGGTTTCTGGACAACTTTAGAGACCTTTTGTGAAGTTTGAATAAAATCTCTTCGAGATTTTGATAATTGCATTAGCTTTAGGACTTAATTGGAATAGAATTAAAATCCTTAAAACAAGCTCTTATAACTAGAAAATTGGTGTTTGTAGGTTTTGTGTGTGGGGTTTTTTTTTTTTTTTGGAAGGAGTCTCGCTCTGTCGTCAGGCCGGAGGGCAGTGGTGCAATCTCGGCTCACTGCAACCTCCA

>HIST1H4B_H_sapiens hg17_dna range=chr6:26135459-26136458 NM_003544 NP_003535 H4 1 bp downstream - 999 bp upstream -

TGTCTAACCAGCTGACAACAAAAACCAGGTACGCGAAAAGAAAGCAAGCCACGAGCATTTATACACGAACATCGGACCTTATTGAGAACTGAAAGCGGGAGCGAGGATAAGGAGGCGTTGCTGCCTCACTTTTTGCTCCGCCCCTCGAGGGGCAGTGACCTAAGGACTGCGAGGGAGAACACAATAGTTTCACTTTTTAATCCCTTTAGTTTTTCCCTCCCGTTTACGACACTACTATTTGAATCTGAATTTATACCCTCGACTGAGAATTTTAATAAGGGCTTATATTAAGGGCTTTCACTAATATGCCGGAGTGGTAAACTTTTTAAGTCTTTCAAGTGCTTGAAGACATATTGACTATTCAAAGGTACTTAAAAGAGCAGGCGTGAAAAGATCACTCTGGCCTTATGTTTTCTTGAAAGCTGACCTGTTTCTTAGAAGCAGTAGGTGAAATTCTCATATGAAAGATGTTCTCACTGTAGTTTAAAAAAAGCAACATTCTTCTCAAGGATAGGAGGCTGAGGCCAAGAGAATTCTGTACAAACCTTGCACTAGCCCTTGCTGGGCGCTTCTCTACACAGTTATATATTCTAGCCTAAATCCCTTTGCTTTAGCGCATTTTTACATTTTACTAATTGTCCAATTCATTATATAAGTAGCTAACTGCTTTTTTTGGGCTTTCATTACCTTATGAGAGCACCTGTGTCACGTAAAACCTGTGTTAAATAAATGCATATACCTTTCTCCTGTTAATCCATTTTACATTAATTTAATTTGCTGGTCCAGCCAGAGCCCTAAGAGGATGGGAGTGGAGTTTTGCTATCCTTCACACTGTACTGTCTCATTCAGAAGAGGGTGATAGCTCATTGCAACCGTGCCTTCATCTGTAAATCGGGTTATGATGATACTCAGGGGACTTTTAATTAGCTAATGTGAACGAGGCATGAGAAGAGACTGTGGAAAAGAAATAAATATTACATAATATGGTTTAATTTTAGAT

>HIST1H4H_H_sapiens hg17_dna range=chr6:26393706-26394705 NM_003543 NP_003534 H4 1 bp downstream - 999 bp upstream -

TGACTAAGCAACTTCTAAAACCAACTTAAGAAACCTAAACGCCAAGGCAAAGCAACGCCCTTATATGCCGGGAATTCGGACCTAATTGAAGACTGAAAGCGCGCATACCGGAACTTTGGTCCTGCTTCTTGGCCCCACCCCGCATAGCCCAGCCCTGGATGACGTCACCAACGACTCCAGCCTTTGGCATCTGAGCGCCGGCTCCGCGGAGGCGTCTCTTGCAATTGCTGGGAAATAGGAATGAGAGTTTTAAGGCTGTTTTAACAACCAAATCAGATTAAATAAATTAACATGTTTAAATTGTTATCAATTGTTCCTTCAGTTGCTTTAGGACGATTGGAGAGGAGCCTCGGTCCACAAGACCGGAAGCCTGAGGAGTGCAGCTTTGTCCCCAAGGTCACAGTGACTGGCTTTCTGCTGCCGGCTGTAAAGCACATGCGGACCGAAACCCCCCTTCGGAAGTGTTTGAAAACTGCAAGATTTCACCCTCTCCATCACCAAGCCAAGGGAAACATAACCAAAAGGGTTTTCTTCCCGATGGCGAAGGCATGATAGGTTAGTGATTCGTGGTAGAATTATTGCCCTCCGCGCGGAGGCCTGATTCTATTCCCAGTTTGTTCTCTTCTTTCCCTGCTTATTAAAATTTGTAATTGCCATTTAAATATCTAACTGCCCCTCACAACTGCACCCAGTGTGTAGGTTTCGGGGTGGGGGTTTTTTTTTTTCCTTTTTTTAATTTTATTGAGACAGGGTCTCGCTATGTTGCCTGCCTGGGTCTTCCAAAGTGCAGTGACTACAGGCGTGAGCTACCGCGCCTAGCCCACCAGTGTATTGATATTTATTTTTCTATCCCTAGTTTTGTTTTCTGTTTGATTCTGGTGATTCCTTTTTCCAAAGTGAGTTGGCAACCTGTGGTAGCCAAAGAAGTAGGCAACTGCTCGTAGGTTTTTTCTTAAATTACGAGGTAGTCTGAACGCATCTCCTGTAAGTAGTTAAGAGT

>HIST1H4D_H_sapiens hg17_dna range=chr6:26297283-26298282 NM_003539 NP_003530 H4 1 bp downstream - 999 bp upstream -

TCTTGAAACCACAGCTGTTAAATCTGTAACGCAATACGTCTGGCAGAGCCACGCAGCACTTTTATAATAGCATGACGGACCTGTTTGAAAACCTCATGAGAAGGGGCGGGAACCACAGTCAGTTCCCGCCCTTCTGTGCAATTCTCCCTTGCTCAGACTGGAGGAGACCAAGAGGGGCCTGAGGCAACAAAAGAGCCTCAGCTGGGATTTAATAAATACTGCTAGGTATATATATATATATACACACACACTATACTTGCAGCCATTAACTGAGCCATCAGTTCATAAACTTTCGACAAAGAAGCCTGACAATTGTCCCCCACTTTTCCCAGAATATCAAATAATTAGTACTGTTTCCCTCACAGGGTGCTTTTTTTTTTTTTTTTTTTTTTTTTTTTTGAGACGGGTTCTTGCTCTGTTGCCCAGGCTGGAATGCAGTGGCACGATCTAGGCTCACTGCATCCTCTGCCTCCCGGATTCAAGCAATTCTCCTGCCTCAGCCTCCCGAGTAGCTGGGGTTACAGGCGCCCGCCACCATGCCCAGCTAATTTTGGTATTTTTTTAGTAGAGACGGGGTTTCACCATAGGCCAGGATGGTCTCGAACTCCTGACCTTGTGATCCACCTGCCTCGGCCTCCCAAAGTGCTAGGATTACAGGCGTGAGCCACCGCGCCGGGCCATAAGGTGCTTTCAAAGTATTTTAAGTAACTCATATCTTTATTGTCTCTGAGATTATTTTTATTGATTTATGCATTCCTAGTTAACCAAGCAGGGCTCTTTGGCATGCAGGCTAGATAGTGCTACTGAGGGAACTAAAAGTATATGAAAGTTACACATTTAGAGGTCAAATTAGGATAAGATCACCGAGGGCCGATTGAAAACATGACTATGTACACCCGTAAAAGAAAACCTTGTTGGGCTGAAATTTATGAAAATTTATGCTTTGATTGTGCCTTTAATCATGGACAGATTGTAGCTAGCACTGCATCATTCACCATAT

>HIST4H4_H_sapiens hg17_dna range=chr12:14815332-14816331 NM_175054 NP_778224 H4 1 bp downstream - 999 bp upstream -

TGATTGCCAGTCGCAGACCAGGGCTTATAGTCTCTCGATGCGGACCTGTTTGAAAACATCAAAAAAGTGGGCGGAGCTCTAGGCCCCACCTCTGGAGACATGCTATAGTTGTTAAGTCCAGGAAGCAGTCAGCGAAACCAGCCTGATTTTTTTGTTGTTTGCTCTTTTGGGAAAATTTTTTCTGATGTTTGGAGAAAGGTTCATGGTGATACGTTTTAAAGTTAAAACAGTATGAGCTAAAAGAATTTATATTGCCTTTTCTCAGCGCTTAAATAAAAGTAGGCCAGATCCTATGCACGTTCCTTGTTCTGTCCCTCCCTCCTTCTGTTTTTTCAAACGGAATTCCTGACTTACTATGAAACTCTATAAAAACCTGAAAAGCTTTACATCCGAGATTTCAAAGCAATTACTTAAAATTTAAAATTTTTCACACGTGGGTCTTCTGAAGACATAGTAGGGGGAGAGGACTTTTTTCTCTTTTACGGCCCTGCAGATAATTAAAATTCTTTCAATTGAAAAAAGAAAGTGCAGCTTCCCGTCCCCCACCTACACTCCACCCCCACCTCCCCCCGACCCCCGCCATTCCCCTTTCAGCTGCTTCTGCGCAATTTGCAGGTTTTCCTGCCCTGCAAGAGCCTGTGTGCTTTTCAACCAAGTAACTTCCTGTGGTTATTTAGTTGAAATATTGTCAATGTCTTATGTCATTCAAATCTCAGGTTCTCATCTCACATTTTCCACTTAAAAATTCAAAGTTCTTGTAAAACAATGGATGCCACAATCATGACTTGGAATAATGATTTCTACTGCAAATCCCCTCAGGAGGTTCTGAATCCCTTCCTTTCTCAAGAGAATCCCTGAACAAAAACGAAAGTTTCTGTAAGGTGTCACCGCTCTTCAAAGGTGTCAGGACCCTGTCTTCTCTAAAGGTTTTACTGTGTTAAAAAGCAGCTGTTATTTAGAGGATGAGTCATAAGTCTTTCCCAGGTAGTTAATTAGGATT

>HIST2H4.1_H_sapiens hg17_dna range=chr1:146645798-146646797 NM_003548 NP_003539 H4 1 bp downstream - 999 bp upstream -

TGTCAAGGCGAGCACCGTCTTCCCCTGATATACAAGAGTATCGGACCAGATTGAAAACCGAAAGCGCGCCGGCGGGAAGCGTTCTCAATTGTCCCCGCCCTCTCGACATTTCGTCATTTCTTTTCTGTTTTCCTCTCCCTCCTCCTCCACTCTGCTCAGGTCCCTCTCACTCTTTTTTTTTTTTAACCGCTACGCCACAGTCCCCGGGAGAATTCAGATCCCAACCGGGGCTTCCGGATTCTGTAGTGGCTTTGGCCTGTGTCTGGTCTGAGGACGCCCGGAAGGCATTGCACTGAGGCTAAGGGAAAGGTCTCTGGAGGGAGCCTCAGGAAGAGCAAATGGAGGCCAGAGACTGGCAGGAGCGCGCCAGCGCAGGATTTAATCCCGACGAGCGGATTCAGAGCCGTGCTTATATAAAGCTTCAGGAAGCGCCGTTCCGACGATGAGGTCGACACGCGAGAGGCGACCTCAAGAGCGGCGGCGCCAGGGATCTGTGCGCCAAGGGAGGACGGGAGGGAGCAGGTTCGCCATAATTCCTGGCTCCAGGCTCTGTTTTGTTGGACCGAGCCACTGTATTTTAGCTCACACAGGAGAATTCTGGCCCTGGGAAAATTGGTCTCAGCATGCTGCCAAGCTTTCTCATGGACGTCAGCGAATCCCAACACACTGTCGGTCAAAGCCGTGCTGGAAGAAACAAAACAGTTCTCCCTCGGTAGAACTGAGAGGGGATTGGTCCAGGGCCCCCGCCGATACCAAAATCCAGGTTGCTCAAGTCTCTCATAGAAAGTGGCGTAGTATTTGCACATAACTATGCACATCCTCCCGTGTACTTTAAATAGTCTCTAAATTACTTCGTAACACCTAATCCAGTGTAAATGCTATGTAAGTAATTGTTATACTGTTTTTATTTTTACTATCTTTTGTTGTACTTTTTTTTAAAAAAGAAATTCATTTGTTTAATATTTTCGGTCTTGGGGAACCCGCGTATATGGAGGGCCTG

>HIST2H4.2_H_sapiens hg17_dna range=chr1:146616295-146617294 NM_003548 NP_003539 H4 1 bp downstream - 999 bp upstream +

CAGGCCCTCCATATACGCGGGTTCCCCAAGACCGAAAATATTAAACAAATGAATTTCTTTTTTAAAAAAAAGTACAACAAAAGATAGTAAAAATAAAAACAGTATAACAATTACTTACATAGCATTTACACTGGATTAGGTGTTACGAAGTAATTTAGAGACTATTTAAAGTACACGGGAGGATGTGCATAGTTATGTGCAAATACTACGCCACTTTCTATGAGAGACTTGAGCAACCTGGATTTTGGTATCGGCGGGGGCCCTGGACCAATCCCCTCTCAGTTCTACCGAGGGAGAACTGTTTTGTTTCTTCCAGCACGGCTTTGACCGACAGTGTGTTGGGATTCGCTGACGTCCATGAGAAAGCTTGGCAGCATGCTGAGACCAATTTTCCCAGGGCCAGAATTCTCCTGTGTGAGCTAAAATACAGTGGCTCGGTCCAACAAAACAGAGCCTGGAGCCAGGAATTATGGCGAACCTGCTCCCTCCCGTCCTCCCTTGGCGCACAGATCCCTGGCGCCGCCGCTCTTGAGGTCGCCTCTCGCGTGTCGACCTCATCGTCGGAACGGCGCTTCCTGAAGCTTTATATAAGCACGGCTCTGAATCCGCTCGTCGGGATTAAATCCTGCGCTGGCGCGCTCCTGCCAGTCTCTGGCCTCCATTTGCTCTTCCTGAGGCTCCCTCCAGAGACCTTTCCCTTAGCCTCAGTGCAATGCCTTCCGGGCGTCCTCAGACCAGACACAGGCCAAAGCCACTACAGAATCCGGAAGCCCCGGTTGGGATCTGAATTCTCCCGGGGACTGTGGCGTAGCGGTTAAAAAAAAAAAAGAGTGAGAGGGACCTGAGCAGAGTGGAGGAGGAGGGAGAGGAAAACAGAAAAGAAATGACGAAATGTCGAGAGGGCGGGGACAATTGAGAACGCTTCCCGCCGGCGCGCTTTCGGTTTTCAATCTGGTCCGATACTCTTGTATATCAGGGGAAGACGGTGCTCGCCTTGACA

>HIST1H4I_H_sapiens hg17_dna range=chr6:27214068-27215067 NM_003495 NP_003486 H4 1 bp downstream - 999 bp upstream +

AAATTTATTAAGGTACAGGGCTGAGGACCACAGAATATTACCCCAACCCCCCAGTGGGGTACAGAAGCTTATATACTCTTTCTCAGAGGCAAAAGAGGAGATGGGTAATGTAGACAATTCTTTGAGGAACAGTAAATGATTATTAGAGAGAAGGAATGGACCAAGGAGACAGAAATTAACTTGTAAATGATTCTCTTTGGAATCTGAATGAGATCAAGAGGCCAGCTTTAGCTTGTGGAAAAGTCCATCTAGGTATGGTTGCATTCTCGTCTTCTTTTCTGCAGTAGATAATGAGGTAACCGAAGGCAATTGTGCTTCTTTTGATAAGAAGCTTTCTTGGTCATATCAGGAAATTCCAGAGAAAGTCCCTCCCTGTATTTGGGGAAGAGAAACAGGACAAAGTTAGAGGGACCTTGATTCTTAGACTTGTTTCTGAGAACCCTCAATTTTCAAAAACACCCACCATTACCAAGCTCGATATTTGGGGGGATAATTCTCCACCCCAAACACTAGAAATGAAAATAAGTAGAAAAGAACTTAGCAATATACCTGAACGATCTTTAAATTCTATGAGTCTATCTTGTTCTATTGCTAGGAATTTAGCATATGGAGTATATTTCCATTGTATATTAAGGAGAAAAATGCCACAAAATAGCATAGTTCCATACAAATAATGTTAAAGGAAAACATGCATATATGCAGAGATAACATTCTTTTTGCTTTATGTATCTTCTATAATGTTAACATTTTTGTTATGAGGCAGGAATCATGTTAAATGAAAAAAGGAAAAACAGCCTAGGTCTTGAGGATTAAAAAGGACTGAAGGAGAACAAGAGGGAGTAGAGCACAGCAGGCCTGTTTCCCTTTTAGGTCCCCTCCCCCAATGCAGAGGGACTTCCCGCCAAAGCTCTTCCGGTTTTCAGTCTGGTCCGCAGAGGTTACCCATAAAAGAAAGCTGCCATCACAGGCAGCAGACCTTTGTTCTCTGACCACTTGATAA

>HIST1H4E_H_sapiens hg17_dna range=chr6:26311853-26312852 NM_003545 NP_003536 H4 1 bp downstream - 999 bp upstream +

GGTTTAGATGTGCGAGTTGGCTAGATATTTAAATGATAATTATAAATTTTAATAAACAGGGAAGAAAGAAAAATAAGCCAAAAATGTAATACGGGCGTCCACAGCGAGGACCAGAATTCATGGGATCAAAAATTTTTTTTTCTTTTCTTTGAGACGGAGTTCCGCTCTTGTTGCACAGGCTGGAGTGCAATGGTGTGATCCGGGCTCACCGCAACCTCTGCCTCCTGGGTTCAAATGATTCTCCTACCTCAGCCTCCTAAGTAGTTGGGATTACAGGTATGCGCCACCAGGCCCGGCTAATTTTGTATTCTTAGTAGAGATGCGGTTTTTCCGTGTTGGAGACTGGTATTGAACTCCCGACCTCAGGTGATCCGCCCGCCTCAGCCTCCCAAAGTGCTGGGATTACAGGCGTGAGCCACAACGCCCGGCGGGATCACTAATTATCCGTGTTTTTCTCTTCGGGAGGAAAATCCTTTTGGTCAGATTTCCCTTGGCTTGGTGGTGGAGAGGGTGAGATCTTGCAGTTTTCAAACACTTCCTAAGGGGGTTCCGTCCCCAAGAGAGCAAGCAGCAGAAATGCCAGAGGAACCGTGCCTATGGGGACAAAGCTGAACTCCTCATGGAAGGAGATTACTGCAGTTCCGGTCTTGTGAGCCAAAGCTCCTGTTCAATCGTCCTAGAGCATCCCAAGCAGCAAATGATTTTAAACATGTTAATGTATTTAATCTCATTTAGTTGTTAAAATATGCCTAAATTTCCTCTTTGGGAACGCAAGACTTGCAGAGATGACTCCATGGAGAGCGGACTCTGCCGGCGGGAACTGGAGTCGTTGGTGACGTCATCCCAGTCTGATCTGTGAAGGGTAGGGCCAGCAGGCAGCACCAAAGTTCCCGTATGCGCGTTTTCAGTCTTCATTTAGGTCCGAATTCCCGGCATATAAGAATACTACCGTCGCTTGTTTTTCAGATTTTTGCGGCTATTTTCGTTGGTGTGTTGGTCA

>HIST1H4F_H_sapiens hg17_dna range=chr6:26347634-26348633 NM_003540 NP_003531 H4 1 bp downstream - 999 bp upstream +

GTGGTGGCTCACGCCAGTAATCCCAGCACTTTGTGAGGCCAAGGTGGGTGGATCACCTGAAGTCAGGAGTTCGAGACCACCCTGGCCAACATGGCGAAACCCCGTCTTTACTAAGAAAATACAAAAATTACCCAGGTGTGGCGACGCGTGCCTGAAATCACAGCTACTCGTGAGGCTGAGGCATGAGAATCGCTTGAACCCAGAAGGCGGAGGTTGCAGTGAGCCGAGATAGCGCCACTGCACTCCAGCCTGGGCAACAGAGTGAGACCCCGTATCAAAAAAAAAAAATTAGTAAAAATACATTTTCAAAGAATCTTCACATTTAAAAATTAAAGCTAGGCGCCTGTAATCCCAGAACTTTGGGAGGCCGAGGCGGGTGGATCACCTGAGGTCAGGAGTTCGAGACCAGCCTAGTCAACATGGCGAAACCCCATCTCTACTAAAAATACAAAAATTAGCCGGGCGTGGTGACGCGCGCCTGTAGTTGCAGCTACTGCAGGGGCTGAGGCAGAAGAATCGCTCGAACCCGAGAGGCGGAGGTTGCAGTGAGCCGAGATCACGCCACTGCACTCCAGTCTGGGGCAGAGCAAAACTGTTTCAAAAGAAATAATAATTTTATTCTCTTGTCTATCCATTCGGTAGACAAGTGTAGCGTTACTGCCTCCTTTTTGCAAATAATAAGGATTTTAGGAGACAGACATTATCAGTTCGAGAGCCCACAGGCACTGGACTAGAAGACACAGAAACCTACACTCAAGACCCAAACAAGGAAACAGGAAGGAGTCAAATCAACTTGCATTTCCCCTACCCTCACCGCCCCCTTTCCTTCCCAAGGCAATAGTGTAGGGGACGCCCAGTAAGTTACGGAAAAGGCGGAGACAGAGGTTTCGTTCCCGCCCCTCCAATTCAGTCTCCAAAAAGGTCCGCATAATTGATATATAAGGGGCTTCAGTGTGTAGCAAAGTTGCAAAAGTTAAGAGTTGTTGTTTGTCTTCGATCA

>HIST1H4A_H_sapiens hg17_dna range=chr6:26128887-26129886 NM_003538 NP_003529 H4 1 bp downstream - 999 bp upstream +

GTAAACTACCTTTCCAGCGCCTGGTGCGCGAGATTGCGCAGGACTTTAAAACAGACCTGCGTTTCCAGAGCTCCGCTGTGATGGCTCTGCAGGAGGCGTGCGAGGCCTACTTGGTAGGGCTATTTGAGGACACTAACCTGTGCGCCATCCACGCCAAGCGCGTCACTATCATGCCCAAGGACATCCAGCTCGCCCGCCGCATCCGCGGAGAGAGGGCGTGATTACTGTGGTCTCTCTGACGGTCCAAGCAAAGGCTCTTTTCAGAGCCACCACCTTTTCAAGTAAAGTAGCTGTAAGAAACCAATTTAAGACAAAAGGGAATGCATTGGGAGCACTTTTCGTTTTAATGCTACTGAAGGCTTCAAAACCAATCGATTTCGGCCGGTCGCGGTGACTCACGCCTGTAATTCAAGCACTTTGAGAGGCTGAGGCGGGCGGATTACCAGAAATCAGGAGTTCGGGATCAGCCTGGCCAACATGGCCGAATCCCGTCTCTACGAAAAATACAAAAACACGCCGGGCGCGACGGCGAGCGCTTGTAATCCCAGCTACACTCTGAAGGCTGAGGCAGGAGAAACACTTGAACCTGAGAGGCAGAGGTTTCAGTGAATCGAGATGGCTCTAATGTACTCCAGTCTGGGCGACAGAGAGATTCGGTTAAAAAAAAAGTTCGACTTAAAATAATTCTGGAGTCAGAATGGGTTTACATTTAATTCTTAACCCAGTTCCTCAAAGCCTGTAGCTCTGTTAAGAAAATAAAGGCCATTGGTCAAGCCTGCTTGGTCCCACCCTCATCTCCCCACCCTCCCCCAATCGCTGCTCCCGCCATTTCCTGGGGCTTGGAGGAGGGGTTAAAGGAGCGGACTGTAGGCGTCACATTTCCCGCCTGCGCGCTTTTCAGTCTCAGTGTCCGCTGGAGGTGGGGGCAGGGGTAACGTAGATATATAAAGATCGGTTTCCTATTCTCTCACTTGCTCTTGGTTCACTTCTTGGGAAGTCA

>HIST1H4C_H_sapiens hg17_dna range=chr6:26211156-26212155 NM_003542 NP_003533 H4 1 bp downstream - 999 bp upstream +

GCAGGAAGCTAAGAATAAAATTTTGAATTGAGAAGTCCCTTTCTTCAAACCACATTCAGACCCAATTCTGCTATTCTATTTATTTTTCAAGGGGATTAGCCTTATTTTAACACCAATAATCTTATCACAAAAACCTCCCAGAGGAAGACCCTGTAGATTTTGTAATGACCTTAATCAAGTATTAGCCCTACACTTCAATTAATCCCCAACTGTACAAAACGAATGTTCTTTTCTCTAAAGCTGTAGCAAGTTGAAAGGGGATTAAAAACGGAGGGAAGGGAAGAGTGTTTGGAATTTCAGGCACAGCAAACAGGCACAGCAGACCAGGAAGAGCGTCCCGGGAAAACATATTATCCAGACTTAAGTTTATATTCCCTGTCTCTCTCAGACTTTTGCAGAAAAATGAGTCATTCAACAAATATTTGAATCGAGATAGGGAAAGTGACGAGGAAGAAGTTTGCACTTATGAGGTTTTAATTTGCAATTATTTGGCTACCTTTTTGCCTTCCCAAAACATAGGGTCTTTAGGAGTGAAACTTCATAGCCAAACTTATACCTTGTCCAGCACAGAGAAGGCCATCAAAATGCCTGGTTTAAATAAAAATATTAAAATGATTGGGAGGGTAAATCCCTTGACCTATAAATCTGACCTCCTTTAAACATTATTTGTATGTTCCCCAATAAACTATTCCGTAATTTATTAGTTAGCAAGTGGAAATAAAAAGAAATGTGGAATGGGGCTATGCTTAGCGTCATTAAGCTGACAGGAATACAGCGCATTCAACTTGCAAACACCCTTCCACTCCCACAAAGAGCAAGCTGTCACTGGCCAATCAAAACAATGAACCATAATGAAACAGTTTTTCTTGCTCCACCCACTTGGTGACCAAATTTGAAAAAAAAAAAAAACCGCGCCAACTCATGTTGTTTTCAATCAGGTCCGCCAAGTTTGTATTTAAGGAACTGTTTCAGTTCATACCTTCCACTGCGATAGGAATCA

>HIST1H4G_H_sapiens hg17_dna range=chr6:26355184-26356183 NM_003547 NP_003538 H4 1 bp downstream - 999 bp upstream -

TGATAAACAAGTCAGAACTATCTCTAAACAAAACGATTACTCGCTTTCCGCAGCCTTTTATATAACGGTAGAGTAGACCTTTTTGAAAACTGAAAGACCAGGAAAGGCGGGAAAACGTATTCAGTCCCCGCTCATCTACTTCTAGTAATAACAAGACATAAGAAATTCTCTTGTAGTAGGGAGATTTGCTGGCATTTAGAGAATAAGGATCTCTAAACGAAAGTGTCAAACATGTACCTAGAGGAAAGTATCTCGATTTTTGATGTCTGTATGTGTTTAAAGATTTACTCCTGGCCGGGCGCGGTGGCTCACGCCTGTAATCCCAGCACTTTTGGAGGCCGAGGCGGGCGGATCACGAGCTCAGGAGATCGAGACCATCCTGGCTAACACGGTGAAACCCTGTCTCTAATAAAAATACAAAAAATTAGCAGGGCGAGGTGGCGGGCGCCTGTAGTCCCAGCTATGCAGGAGGCTGAGGCAGAAGAATGGCATGAACCCCGTGGGGTGGAGCCTGCAGTGAGCCGAGATCACGTCACTGCACTCTAGCCTGGGCAAGGGCGAAGCCCGTCTCAAAAAAGAAAAAAAAAAGATTTACTCCTTCCCTGTATATTAGAGCATTTACCATGCTGAAGTTGAAACAAGCCCAAATTCCAAGACATAAAAGAATTCCTAATCATATTGCCAAGCCGCCCAGCTAAAGAGAAGTAGCCTGGTTTTTGGTTTCGAATCTGTGAGTGACTGCTCTCTCACCGATATTAGACATGTTAATCTGAGTTTCAATGGCCCTATCTGTAAAAAGGGTAAATACTTGTTAATAATTGAGTTACTTTAAGCAAAACACTGGCATATAGTTAGTAAATGATTTTTTTTTAAAATACGGAGTTTCGCTCTTGTTGCCCAGGCTGGAGTGCAATGGCGCAATCTCTGCCCACTGCAACCTCCACCTTCTGGGTTCAAGCTATTCTCCTACCTCGGCCTCCCAAGTAGCTGGAATTACAGG

>HIST1H4J_H_sapiens hg17_dna range=chr6:27898883-27899882 NM_021968 NP_068803 H4 1 bp downstream - 999 bp upstream +

GATGGAATATTAAGCAGATATAAATTACTGCTTTTAAAATTTTTCCTAATGTGAATTTTCTAAGCTTTTAACCGTGAACATAGATTCCCTTTGGCAAACCACCTTGATTAAATTATAGACAGAATAGAATTTTGGTAATAGTTCCGAGTATGTATAATGTTAAAGGTGGTACTTAATCCGCAGGGAAAAGATGAATTATTTAAAAAATCTGGTTGATTAATAAATTCCTAGGGAATTGGTTAACACTGTAGCAGGCAGAATTCAGGAGAAGAAATAATAAAGACATTAAAATCACTTTTGTAAACTTTTTAAATAAGGGAAATTGATTAAAACAACGCGAAGTTGTAAAATGCCATATACTGCATACATTTTGTGGGAAAAAATGTGCATTTATCTGCTCACCAAATTATCTTATTAGCTAGCAGTAGAGCTCTTTGTGTGATGGGAAGATGGGATAACAGGCTGTTGTTACTGTACTGGTTTTTTTTTTTAATATATTTAAAAAAAAGAGTGTCTCCTAAAGTGTAAGCGCTATCATGCTGGACTAATGTTTTAATTTAATTTAATTGTTGTCTTGGTAGAGATGGGGTCTCGCTATTTTGCTCACGCAGATCTCAAACTCCTGACGCCCAGGTACACTCCCATCTCGGCCTCCTAAAATGGTGGGATCACAGACGTCAGCCACCGAGCCCGGTCACTTTTTTGTATTCCCCACAGTATTGATGTATATCTTCTGCGTTCAAAAGCAATTTTTTAAAGCCTCATAACGTGGTAACAGAATACTTTGCACATTACAAAATTCAGAACACGGAAACAAGAAGCTCGCTTTTTTTTCCCCCCTATTTCGGTTTGGCCCTTTAGATTTCCCCTCCCCCACCGGGGCGGGACTTCCCGCCGACTTCTTTCAGGTTCTCAGTTCGGTCCGCCAACTGTCGTATAAAGGCGCTGCCTCAGGCCAGAGGCCTCACAAAGCGTTGGGTGAGACTCCTCTTGCTCGTCA

>HIST1H2AB_H_sapiens hg17_dna range=chr6:26141775-26142774 NM_003513 NP_003504 H2A 1 bp downstream - 999 bp upstream -

TAACTACTTCTGATAAGGGAAAATCGCCACAAGAAAATGTAATGAAACTACATTAGAACGCAAGGCAGAGAAGTATTTATACTGACTGGAGGTAGGCTGTGAGGAATTCTCCCATTGGCTAATGTCAAATACCCAATGGGAAATCAGAATCTGCATCCTTCATTTGCATGTAATCCTTCCGTCTGGTGTAAGGTTTATGTTTGACCCAATCCCCAGTCTGGCTTGACGAGCCTTCGACTTGAATACTAATAATAATTGGCCGAATTAGGATTTTGTCAAAATACCTTTTTTAAGCATGAGTGGAGGTTTTGTTCTGGTTATTTTGACTTTCAGCCGCTCGTGCTTTTCCCGGATTGTGACTCATGTTTTTGGAAAGGAGTGGACTCCGACCAATTTCTAAATAGATATTTAAGAGGTCCTTCAAATCGGGCGCAGTGGCTCATGCCCGTAATACCAGCACTTTGGGAGGCCGAGGACGGCGGGTCAGGCGTTCGAGACCAGCGTGGACAACATGGAGAAACCCTGTCTCCAGTAAAATAACCAAAAAAGAAACGGGGGAGAAAAAGAAAAAAAAAAGCCGGGCTTGGTAGTGCACGCCTGTAGTTCCAGTTACTCGAGAAGCTGAGGTGGGAGGATCGCTTGAACCCGAGAGGAGGAGGTTGCAGTGAGTTCACATAGAGCCACCACACTCCAGCGTGGGCGACAGAGCCAGAAGACTGTGTCTCAAAGACAAAAAAAGGGGAGGGGGAGTGGGAGGGAAGAAAAGCGAATACCCCAAATCCCAGTGAACTGTAGAAGCTTATAAGCTCTCTTGATTCATAAGGGAGAAAGAAGGGGGATGTAGGCAACTTAGGGGAGAGTATATGATTTTGGAGAAAAATAAATGGGTGTTTCAAAGAATAGGTGACAGCTGTGACAAAGTCTGTTTAGATGGTGTTAACCACCAGTCTCCTCTCCTGTGATACAGTTAATCTTCTCTGGTTGATGAGATTCCCCAGGG

>HIST2H2AA_H_sapiens hg17_dna range=chr1:146627391-146627620 NM_003516 NP_003507 H2A 1 bp downstream +

GCCGAAAACGCCGCTGCTTCACCTTTTTATAGACAGAACGGCGATTGTCCACGGAGCACTTTGATTGGCTCAAGCAAATTTTGTCCCGATAGCCAATAGGATAGCTCAGCCAGAATCCACTCATTTACATAATCTCGTCTCCCTCGCATTGCGCGCCGCGGAAAACTCGCGAACCATAACGCAGCGTCATGCGCACAGCCTCTGTAAGTACACAGTCGTTTCCGGTAGAC

>HIST1H2AL_H_sapiens hg17_dna range=chr6:27940087-27941086 NM_003511 NP_003502 H2A 1 bp downstream - 999 bp upstream +

TTCGCTTCTTGCCTTAGTAATAGTCTTCTTGGAACCCTTTTTCGGGGCTGTGGCCGACTTGGTGGGGTCAGGCGTGTTGTAGTTTAACTGCAGAAAGGGTACAAGGAATAGGAAAAAGCGGCCGGGCGCGGCGGCGCACGCCTGTAATTCCAGTACTTTGGGAGGCGGAGGCGGGCGGATCACTTGAGGTCAGGAGTTCGAGAGCAGCCTGAACAACATGGCGAAACCATGACTCTTCAAACAAATGTAAAAAATTAGCTGGGCGCGTGATGCACTCCTGTAACCCCAGCTACTGGGGAGGCTCAGGGAGGAGGATCGCTTCGGCCAGGAAGGCGGAGGTTGCGGTGAGCCACGATCGCGCCACTGCTCTCCAACCTGGGCGGCAAAGCCAGACCCTGTCTCAAGGGAAAAAAAAAAAAAAAAAAAAAAAAAAAGTCGCCGGGCGCTGTGGCTCACGCCTTTAATCCCAGCATTTTGGGAGGTTGAGGCAGGTGGCTCACCTGAGCTCAGGGGTTTGAGACCAGGATGACCAATATGGTGAAACCCTGTCTCTACTAAAAAATACAAAAATTAGCCGGGCTATGGTGGCTGACGCTTGTAATCTCAGCTACTCAGGAGGCCGAGGCAGGAGAATCACTTGAACCCAGGAGCGGACGTTGCAGCGACCGGAGATCGTACCACCGCACTCCAGCCCGGGCGAGAGGGAGACCCTGTCTTAAAAAAACAAAAAAAGAAGAAGAAGAAAAAGCTGTGTGTTGTAACGTCCTGTTCACGAATTCTTATGCAAATGAGGTGATTAATAAAATAGTGTGTAGGACTGGTGGCGATTCTAGATGACGTCATATACACGTTTGTCCAATCCAAAGAAGCATGTTTCATAACAGCGATTCCATTGGTTGAAATAAAACTGTAATTTGAGCCAATGGCACAGCTTTATTTTCGCGCCCAGTATTGACTATAAGTAGTGGAGCTCTGGTGAACTTCTCTCGGCTGTTCTCAG

>HIST1H2BB_H_sapiens hg17_dna range=chr6:26151864-26152863 NM_021062 NP_066406 H2B 1 bp downstream - 999 bp upstream -

TTGCTATTCCTAAACAGAATAGAAAAGCTACTAACACTCTCCACTACAGAGTAGTACAGAGAACAGTTCAGAGCCCATGTATTTATAGTCCTGAGATTCAAATGACGGTTTAAGATTCCTCACTTCTGATTGGACAAAAGAAACACGGTTTCACTGAGGGGTGGGGTTTATGCAAATATGGAATTTATGTTATCTTTTTCTATTGGATAAAGCACCAAACATAATTGACCAATAGGATAGCTTCCTATTGCAGCCTTGCAGTTTGTATAAAAGGATTTGTTCAGGCGCCATTCCAGCTTGCTTGTCTTTCACAGTTTTCCGCTGCTTTCATAGGTCGCTATTTGCGGACGTGGAAAATGGAGCTAAAGCAAAAACTTGTTCGTCGCTACCGGGCTTGCAGTTCCCAATAGGGCAGAGTCCGTCATCTTTTTCGAAAGGGCAATTATTTTGAGCCGGTCGGAGCCGGTGCGCCAGTGTACTTACAATACCTGGCCGCCGAGATCTTAGAACTGGTGGGCAGCGCCATACGTGACAAGACCCGCAGCATCATCCCCCGCCACCTGCAGCTGGCCATCCGAAACGACGAGGAGGTCAACAAGCAGCTGGGCAACGTCACTATTGCTCAGGGAGGCGTCCTGTCCAATATTCAGGCCGTCCTGTTGCCAAAATAACAGAGCCACGATAAGGCCAAGGTCAAGTAAACACTCAAATCAGAAAACGTAGCTTACACTTGAAACGGCATTTTTCAGAGCCGTCCATAGTTACACAAGAAAGGATGATAACTTGCTTCTGTTAGGGTATTTTTTGCTTTTCGTTTGGATTGGTTTGTTTTGAGACAGTCTAGTTCTGTCACCCAGGCTGGAGTGCAGCGGCGCGATATCGGCTTACTGCAACCTCCACCCCGCCGCTTCACGCGGTTCTCATGCCTCAGCCTCCTGTGTACTTGGGATTACAGGCGTCTGCTACCGCGCCCAGCTAGTTTTTGTATTTTTATGCGAGA

>HIST1H2BI_H_sapiens hg17_dna range=chr6:26380900-26381183 NM_003525 NP_003516 H2B 1 bp downstream +

GCAGGTGTTTAAAAAAATTTAAAAAAAAAAAAAAAAAAAAAACCACTTCTGACTTACATACTTATAGTTGAAAGGTCAAATTATGCAAGGTTGAGCTTCTGGGAGAGCCGATGAGATTTAGGAGTTCAGAAATGGCCTATCATATATTCAGACACTAAAAGAACCAATGAGAAACCGAAACTCAGCCAGCCTCATTTGCATATACACGAGGTAAATAATGAGGGCGTTTGGGCTCACCAGCATTTTCCTGTGGTCATTTGACGGTATCACTTCGGCTGCGAACA

>HIST1H2BD_H_sapiens hg17_dna range=chr6:26265329-26266328 NM_138720 NP_619790 H2B 1 bp downstream - 999 bp upstream +

TCAGTAAAAGAGCTGTTGCACTATTAGGGGGCGTGGCTCGGGAAAACGCTGCTAAGCAGGGGCGGGTCTCCCGGGAACAAAGTCGGGGAGAGGAGTGGGATTTTGTGTGTCTCCGGAGCTATTTTTGACTAAGGCGTCGCGTCGCCCAAGCCGGAGTGCAGTGGCGTCATCTCGATTTTGCGTTCTCGAGTGTCGGAGTTGAACCCATTTGGGCCTCCCTTGTGCTTTGCACTTTTAGCAGGCCCTGGCCTCCAGATAGCATGGGAAAAAAAATGTTGGGATTTTCCCGGGTTTCTAAGCTGGGTTTTTCCGAGTTCCAAACACGGCACAGTGTATCAGTTTCTGTGCTGGTTACAAGCCTACTGGTTATCCCTATCGAGTATGGCAGGCAGTGAGGGACTTCAGAGGAGTACGTCTTAGGACAAGTGGCATAGTACTGACATTATTTCCGAAGGGCTACATTTCAAGTGCTTGGGGAGACTACTGCCACATAACTGAAAATTAGAAACCGACACTGCAGAAAAATACTTGGTCCTTAAATGTGGCATTTGGATGGATTAAGGACTTGCCGAAACGTAAAACTGACAGACTTGGGGGGGGGGGATGTCCCAATTAGCACGGCTTCTGTATGCAACGAGTCCCATACTTTGTTAAAGGAAGAAAGGAATGTGAGTTCTCCTAATCTGTTAAGTATCTTTCGGTGTAAGTTCTGACACCACAATGTTAAAAAAGTCGGATCTCAAAAACCAACTGCTCCAAGCGAAGTGCACAGCTGTCTTGCCTAAAGAGGCCTATTTATAGTAGCCTCGGGTAGTCTGGTCTGGGCTTTCTCATTGGGTACAAGTAAAGGAACGAAATAGCCAATGAAAAGGTAGACTTTTAAGTGTCGTTTACATTGGCATTTGTGACGACACTCTAAAATTAATCCAATCATAAACGAAATCTGATTAACCTCATTTGAATACCGCATCTATAAATGAACAGGGCCTCGGCGGGAGTG

>HIST1H2BE_H_sapiens hg17_dna range=chr6:26291004-26292003 NM_003523 NP_003514 H2B 1 bp downstream - 999 bp upstream +

ACTTTTGTTAGATGCTAACCCACAGAGCGGAATACTTTTTGCGCAGCACCTTCATTCCTGGAGACTTAGGGCCCGGGAAATGGGAATGGTGGCAGGAGAGGAGAAGGGCTAGGGGGCTGGGGAGCGTGGGGTGAAGGAGTGGGGGCTTAAAAAGGACAGTCAAATTTAGGAAGAACAGTTTCTTAAACTGAAGTTAGCTAAGCAGCGGGCTTCAGCCAAAGATAATAATACTAACTTGAAAGTAAAGCTGTTTGTTAAATAGGCTTTCATTTTAATTTTTTAAAAAATATTTTCACTAGTTAGACGGAATATTTATTTCTGATCCTCGTTGTAGGGCTAAACTAGGTGTGACTTGCTTTTCCATTAAGGACTGTAGCCATTTTGGCTAAGAAAGTCTTTCCTGTCTTAGCTCTCTTCAAGAGATGCTATTTACTTTTTTGTTGTTGTTTTCTCCGGAGCTTTATTGCCTACCCTTTATTTTCACGATGTGTTTGTGCATTCTGTAAAAAGTGGGAAACACTGTTCTCCGGATTGTGTGGGTGATCGCCGGTCGCCTAGCTTGCCGTTCTTCAGGTATCTCCTGCTGCTCCAGAATCTACATCTGAATGTCAAGGCATAGCTTTTCCAGGAGCTTTTAAACCGACCTTTCAATTCGAAGACGTAACTGCGCCAGGAGCTTTGTCTCCCTGGCATATAAAAGCATAGAAGAGAGCAACTTCTGGTTTTTAAAATAAGTAAACTAATCTGAATTGTTTGCAATGGTAGGAACTTGTTATATAAAATGTTAATTAGGTGGCCCGTGCTCGAAAACTGCTCTCAGGATATGACCAATGGGAGAGTAGACCTAAGCTCCTTCATTTGCATGCAGACTTCACGACAAAATAACGAATCAGAGTTGGAGAAACTAAATCTTCATTTACATAACATTGTCTACAAATTCCGAGGATCAGGAGATGTAGATTTCATTTTCTTTCCTAACTGCAGAACAGCAAAGATAGCA

>HIST1H2BH_H_sapiens hg17_dna range=chr6:26358859-26359858 NM_003524 NP_003515 H2B 1 bp downstream - 999 bp upstream +

TTACTCCTATTTATAAAGGCAAAGTCCTTCTGATTGGTCCGAATCTTCCAGTTTCGCGCCCTGAATTATGTAGTGTGATAGGACTTGTGATCAGCTACTAGGTCTCATTTTAGAAAATCTTAACCTATCTAAATGCTTTTTTCCCCTAGTTCCTTACATTTTATTATGTTTTATGTGCCTACCTGTATTACGTACCTTCATTGATTTTATTTTAATCAGTTTGTTTGGGATTATGTTTGACAGACGGAAATGAACGTTCTGCAGTTCCGGAGCGATTCAAAAATACCGCGTTTGAGAGTGATTGGTCCAGCTCAATGTCTCCTTTATATTTTTTAAAAAACTCTTACTTTGAAGAAATATTTTATTTTTAATATAAGTACCCTATTTCATATATTCTGAACCTTTCAACAAACACTGGTTTCACTTCCAGACACTCCGTTCCTAGGGGATATTAAAATCCCATCGCTCTTGAGATGTGTTCATCCTTTGATTACTGAAAAAGCTTAAATTTTTATGTAAGAATGCTAATGACCTTGATCAGATTATAAAAGAAAGCCAATGGTATAATTCTGGAAACACGTAAATCTTTAGCTAATTTCCATTTTAGAATTAGTTATTCATAGATAATTTTAAATCCGGAAGCTGCAATTTGGTAGGAAACTAGAAACATAATATCCGTCTTTGTTTCTGTTTAAATTCTCACCTAAGGAGACAGAACTTATAGGTAGATGTTACTGGCAAGGCATTAGTTTTTCTAATGTTTTGAATGAAAAACTACGTTTTTTCCCCCGGGAGGTGTAGAAAAGGATTTAGGGTAGGTTTCGCATAAATATCCAATCAAAAAGTAGACTTGAATTTAACTTTTTTATTGGCTGATTTGGTCCAATCAGGGATTGAGAATGATTAAGCACCTATTTGCATAAAAGACCTACAAAAACGGCCAGCTGTGCTGTTGAGCCTTCACTTTGGGGTGTATTCTTACTCCTTTATCTTGTTGCAA

>HIST1H3J_H_sapiens hg17_dna range=chr6:27966549-27967548 NM_003535 NP_003526 H3 1 bp downstream - 999 bp upstream -

TAGTTGAGAAAGCTATGCTCTGAAAGCAAGCAGCTGAATGAGCAGTGGCTTCTACCAGAATTTATAACGATGATCTGATCCTGATTGGTCAGAACTGTTGTAGGAAAAACCTGGTTGGTTGTTAATACTATCTTCATTTGGTTCCTGCCACTTTTTTTTTTTTTTTTTTTTTTTTACCTGACGGCTTCTAATTCTGTCGCCTAGGCTGGAGTGTGGTGGCGCGATCTCAGCTCACTGCAACCTCCGCCTCCCGGGTTCAAGCGATTCTGCCTCAGCCTCCGGAGTAGCTGGGACTACAGGCACTTGCCACGACGCCCGGCTAATTTTTTTTTAGCAGAGAAGGGGTTTCACCATGTTGGCCAGGCTGGTCTCAAACTCCTGATCTCAAGTTATCCGCCTGCCTCGGACTTCCAAAGTGCTTAGATTACAGGCGTGAGCCACTGCGCTCGTCCTCCGCGCCACTTTTATTTATTGTTTTCAACCAATCATGTTAAGATTCCCCCAATGAAAAGATAATCTGACCTAGAATTTGTGTAGGTCAGTCGTAGTCTTAAACTCATTTCCAATGGGTCATTTTTCCTATTTCGGATCGTGTAAAAATACATACAGTAGAACAACGTATTTGCTAGTATTTCCAAAAAGTAGTTCCGTTTTGTAACTGCTTAATTAAATCTTTTAATGACTGTACCAAGACCCAGAACAAAAACAATTTAAACTTAGGCTGGCACATTCTCTAAAACACCAAAACTTTAGTTTTGTCCAGTGTGGTGTGGGGAAGGAGTTTGATAGGTGCTTGACTAAATGATACCAATAAATGGTATCTGACCTCTTCATTAACTGAGTTTAATGCAATCCAATGGCTGATTGGATTAATTATGTAAATTATTGCTTAATTTCATTAGCATGCGCGGAATTACAGGTTTGGGTCAGTGATTAGATTTTTGCCAGTGAATCTGGGTAGTTCTGACAACAGCATTTTATTAGGATGGAGATTTTAGTT

>HIST1H3F_H_sapiens hg17_dna range=chr6:26358814-26359813 NM_021018 NP_066298 H3 1 bp downstream - 999 bp upstream -

GGAATGGGTTTCAAGCAATGGTCACAACTGAACAAACAGTTGTTCTTACTCCTATTTATAAAGGCAAAGTCCTTCTGATTGGTCCGAATCTTCCAGTTTCGCGCCCTGAATTATGTAGTGTGATAGGACTTGTGATCAGCTACTAGGTCTCATTTTAGAAAATCTTAACCTATCTAAATGCTTTTTTCCCCTAGTTCCTTACATTTTATTATGTTTTATGTGCCTACCTGTATTACGTACCTTCATTGATTTTATTTTAATCAGTTTGTTTGGGATTATGTTTGACAGACGGAAATGAACGTTCTGCAGTTCCGGAGCGATTCAAAAATACCGCGTTTGAGAGTGATTGGTCCAGCTCAATGTCTCCTTTATATTTTTTAAAAAACTCTTACTTTGAAGAAATATTTTATTTTTAATATAAGTACCCTATTTCATATATTCTGAACCTTTCAACAAACACTGGTTTCACTTCCAGACACTCCGTTCCTAGGGGATATTAAAATCCCATCGCTCTTGAGATGTGTTCATCCTTTGATTACTGAAAAAGCTTAAATTTTTATGTAAGAATGCTAATGACCTTGATCAGATTATAAAAGAAAGCCAATGGTATAATTCTGGAAACACGTAAATCTTTAGCTAATTTCCATTTTAGAATTAGTTATTCATAGATAATTTTAAATCCGGAAGCTGCAATTTGGTAGGAAACTAGAAACATAATATCCGTCTTTGTTTCTGTTTAAATTCTCACCTAAGGAGACAGAACTTATAGGTAGATGTTACTGGCAAGGCATTAGTTTTTCTAATGTTTTGAATGAAAAACTACGTTTTTTCCCCCGGGAGGTGTAGAAAAGGATTTAGGGTAGGTTTCGCATAAATATCCAATCAAAAAGTAGACTTGAATTTAACTTTTTTATTGGCTGATTTGGTCCAATCAGGGATTGAGAATGATTAAGCACCTATTTGCATAAAAGACCTACAAAAACGGCCAGCTGTGCTGTTG

>HIST1H3B_H_sapiens hg17_dna range=chr6:26140267-26141266 NM_003537 NP_003528 H3 1 bp downstream - 999 bp upstream -

TGGCAAAACCACAGAAAAGCTTGCCTGCAGAGACGTCTGTGGAGGAAAGGAAAGAGCTACTCTTCTTTTATAGAGTCAGACCACCAACTATTGGACCCAAGAAAATTCAAAAATCCCCGCGCCCTTCTTGGATTGGTCCATCTCTGTGCCTGGTTGCAGATTAAGAGAGGCTCCTGCCCATTACCGTAGCTACTCTGACGTCATTTTGTTAACCCCTTAGCTGCTATATCCACTGTGGACAAGTCTTGTACTGGAAAAGTTTCCTGAAGTCTTAAAATTTACAACCACACAAAGCAACGCGGAAACCTCCAATTGTTTCTAGTTAAAATATAAAAAAGAAATCAGAGAATATTGGAGACGATTAGGGAAATTTGCATATGCGCTTTATTTAAAATTGTATTTTTCTGGGTGTCGCATAAGAAGTGTGGGCAATTAGAAAAATGCTCTTAGCCGGGCGTGGCGGCTCTCGCCTGTAATCCCAGCTACTCAGAAGGCTGTCAAGAGGATCGCTTGAGCCCGAGTTCGAGGTTACAGTGAGCTGTTATCACGCCGCTGCACTGCAGCTTGGGCGAGAGGGAGACTCCACCCCAAAACAAAGCAAAACATCCCCAAACTGGAAAAAAGCTCATTTTGGGAAATACATACTCAAATGTTCAGTGGTAATGTGTGTGCTCTCAATTGTGTATGCCATTAATTGCTACAGCAAATGGTATGACATATTCAAACTTGTGTGGGGCATGCGGGTTTTAACACTTCCATTCAAGATAGTTAGGAATGCACTCATGGGTATAATTTCCTTCCTCTAAAATGTAGTAACTGCTGTGTGTGAAACTTAACGCGAATCACCCCTGTAAACATGTTTTGTGCTGCATGGCACTTCTCCCACATACCTAGAATTCCTGAGGTTTCTATGGATCTAATTTCTGCAGGACAAATTACTAAAAGTGCCACACTCAAAGCCATTAAAAACACCTCAAAAACATCTTTATGGGCGGCATAA

>HIST1H3G_H_sapiens hg17_dna range=chr6:26379591-26380590 NM_003534 NP_003525 H3 1 bp downstream - 999 bp upstream -

TCTCAGACTACCTGAAAGAAAAACTCAGCCACTTGGCAGAGTGGAAACTGCCCTTATATATACTCAGAAACATTCTAATTGGCCTTGTTGATTTTCAAAAGACCCGCGCAACAAAACCATTGGCTGAAGAGTCCAACGATGTGATTGGTGAATTACTGGAAAATCTGATTGGATGAATCACTTCATTTTTGAATCCTCTCTTCTCGGTTAGTCTGCGATCCAAGAACGTTAACTTCTTAAAGTTCTAATTCTGCGCTTCTCAAGCTGTAGTCCGTAAACGACTACTTTAAGACTTGGAAGACGCTAATTCTCATTCAGTAGATGTGTTGTGGGGCCAGATTCGGAGGACCACGTTTTGAGAAGTTTATCTTGTGCCGTTTTCTTGAGTCACCTGGACTGCTGCTAGCTGGAAACCTTTGACCATTATAAGGCAGGATTCCTGGGTGATGTACCTGGGAAATCACGAGCCAAGAAGTCACCGACATACAACAGTGAGCAACAAATGTTAACTGAATTCTATTTTGTCCCAAATGGTGTTTTGCTTCGGTTGAGAATTTTTAAGGCACAGGCTAATTTTAGGTTTTCCAACGACTCTTATCATTTACTTTTCGTTGGGTCCTATTGATGATATTCCAGGATACCACCCCCACTAGCCTCTTCTGCAGGATAACTTGAACACTGCAGTGGCCCTGTCCATCTAAACTTTACAAGGTTTTGTAAACGTTACTGAACTTGCTTCTTCCCGACTACATTAAAGCACTTACTTGAGATATTAAACATTTTCTCATTAGTCCTGTTTGCCTAGTCAGTCACTTGTATAGACAGGGCAACAGCACGGCCTGAATATTGGGCAGGACACCGCCCTGCGCGATAGTAACTTTGCCCAACAGCTTGTTGAGCTTCTCGTCGTGGATGGCGAGCTGCAGGCGTCGGGGGGTGACGCGGGTCTTCTTGTCTCGGGCCGCGTTGCCCGCCAACTCCAGGATCTTGGCGGTCAGGT

>HIST1H3H_H_sapiens hg17_dna range=chr6:27884822-27885821 NM_003536 NP_003527 H3 1 bp downstream - 999 bp upstream +

CTCTCGAATCTGTATAATTAATTGCATTTGAACATTAATGATTGAGTTGAGAATAATTATTAGACTAATTAGAACAGTTACTTATGGTGCCAGGTAGTTCTCTTTGAAGCATTTTCTGAAAATTCATTGAAATTAAAATTTCCCGAGCTTTCCCACCCCCAGCCACACAAATTGATTTCTTAGTACAGAGATGACTTAATTTGTTTCAGCATGGTAAATTCCACTTTTAAAATATCTGACTTGATGTTTTTAATCTGAATCAGATCTGGATGTACGAATGGAATAGATTCTTAAAAGAAAATGAATTACATTTCTAGAGTATTGGAAAAATAAGCTGCAATGGAATTACTTTAATCTAAAAGTGAAAAGTTCACATAAAGTTGTTTCTTTGACATTTTTTGGCCTTGAATTTCTCACATGCGAACCAGCTTTTCTTGGTTATAAAAATAGAAGTTTTTTAAATTTAAGTGGTCAAAAGATAGAAAATTGAGGGGACAGGTGTATAAGGCTGTCCTGTCCAGTATTGCAGCCACTAGCCAATTGTGGATAGTGATCATTGGAAATTCCATAGCATGAATTTTATATAAAATACACATTGCATTTGGAAAAGTCAGTGCCAAAAAGTGTAAAATACCTAACAATTTTAATTTCGATAACATTGAAATATGTTGGCTATATTGGATTAAGCAAAATATTAAAAATTAATTTTTCCTTGTTTTGTTACAATGTTCTCCTAGATAAGTGTAAATTACTTATATTGCTCTCATTTCCACTGGAAATCGCTAGTATAGTGAACAACTGTATAGCGAAAATAGAAAATAAAATATGGAGACCATGAACTGCTAAGTCTGTCAGAGGAATAGGTGAACAACAAAAATTTGAGTCCTTCGCCAATCCGGTTACTGTTGGGTAGGCCTTCAGCATACTTTTGTCCAATCAGCTTCAGACTCTCACTATAAATAAGCGGCTAGCTTTCTCTTTCTCCTGAAGTGAATCTAGC

>HIST1H3E_H_sapiens hg17_dna range=chr6:26332363-26333362 NM_003532 NP_003523 H3 1 bp downstream - 999 bp upstream +

AGGGAATCCGATAGGTTAATTCACAATAGTGTTAGGCTGTAATGTCATTCTTTAAGAATCTTTTCCAGTAAACATTTGTTACTTTTTCAAAGTAATTGGGAGCTGTTGAGTGTGGAAGCGAAAACCAAGTTAAGAAGTCCTGTTATTTGGGTGAGTCCAGTTCCTATAAAAGTTCTGTTGGATGTGTCTTAAATTGTTCGGTGCACCTCAGCTCTCTCCCGCAGGGATCCGGAGAAACTGCGCCATTGTTGTCTTACAGAGAAACAACCTTCTAACACTCTCTTCGCCGCCATCTTTTTCAAGATTTTAAATTGAAAATACTTTTTTTGACGGCTCTTGCCATTATGTCTGGACGTTTTAATTTCAACAAAACGAAATAATAGTGTTAAGTTCTTTGAGTCAAACCCGATATTACAGAGGTAGGGATTCCAGGATATTAGAATTTTACTATAACACAAATGAAACACAAAGTTCCTTATGCTTAACAGTGTATCTTAGAAAACCTTGTTTGAGTGAATTAGCCTTGTACTCTATAATGAAACGAGGAATCATTGAGAGATACTTAGGCAACGGATTTTAAATGAGTGAATAAGGAAGGGAAAAACTTCAGGAAAATGCAGAAAAAAGCAGCGGAGATTTTGGAAAAAAAATTCTAAAGCACTTTCTAACGCAAACACATTCGATTTTTTATAGCTAAACAGGATAACAGATCAGTCTATTAAGTTAAATGATCAATGTCTTTGCAGGTCATGGTCTACGTGAAACATCAAAGAAACCTGTAACGGAAAACGACTTGTTTGAAAGAGCCAGTTCTCTATCCAAATTTACCAATCAGAATCTTGCACTGAAAAAATAAACCAATCGTGAATCTCTACGGCCACTTCCGGAATTTAGCAACCGATCACTAACAGGGATCGTCCACAATCCAATCAGAGTGATTCTGTTCCTATATAGAGGGGCAAACCAATCTTCCTAACTCATTTACTTTGCAGATGAACTA

>HIST1H3A_H_sapiens hg17_dna range=chr6:26127698-26128697 NM_003529 NP_003520 H3 1 bp downstream - 999 bp upstream +

TAGAATGTGGCCTGTCTCTTGTGAATTTAAAAAGGTCACAATCCACTTTTCAGTGTTTTGAGATTTTCAAAATGATTGCATTAGCTCTTGGCAATGCTAAATTATGTTCCTTGCGAACCACTATCCAGTTTCTCTTGGGCCAAGTCCACCTCCTGCTCCGCAAGAGGAACAACTCCCAGCTGGTGGTACCTGGCGGCAGTGCTGGAGAAACGCCATTTTGTGACTGGCAGAGTACACCTAGGCTTTAGAAAACAAAAGCTGCAGAACGCTGCAAGTTTAGGATTCAAAGAGCATAATCAAGAGAAAGACGTCTCATAGAAAATGTTTCTGAGTAATAGTGTAATCCTACTATGTTTGAGATGCTTTGTAGATTTCAATAACACTCCTAAGTCAATTAAAGCATTACAAAGGAATCCAATTCTTGTGAAAGGTTTCAGAAATTCCCGTAAAGGGTACATTTCCGGAGAGGAGGTGAGCAGTATTCCCTCTTTTTTTTTTTTTTTTTTTTTTTCCTAAAGAGCTGAAGGTTATACGGAATTGGGGAATTATAATACCTTTGGAATCAATGCCTTGTTTTATGGAAAATAAACACAGCCTTCAGGTTATGAAAACCAGATGTAGAAGAGGACAAGTTTAAAAAATTAAAGTCCAAGCCGGCGCAGTGGGGCTCCCCTGTAATCCCAGCTACTCTGGATGCTGAGGCGGGAAGATCCTTTGAGCCCAAGTTTAAGACCAGCTTGGGGAACAAAGCAAAAGTAAAATAAAATAATAGTAGTAATAAAATACCACTTAAATAATCATCTGTAGAGTTGGAATAGAATATAGTAGCCGGTGAAACTGCACGATTGTTGCTGGCTTAAAGATAGACCAATCAGAGTGTGTAACGTCATATTTAGCGTCTTCTATCATCCAATCACTGCACTTTACACACTATAAATAGAGCAGCTCATGGGCGTATTTGCGCTAGTGTTGGGTGTTCCGCTGTGCTGTTTTTCCGTCA

>HIST1H3C_H_sapiens hg17_dna range=chr6:26152619-26153618 NM_003531 NP_003522 H3 1 bp downstream - 999 bp upstream +

CCATAGTTACACAAGAAAGGATGATAACTTGCTTCTGTTAGGGTATTTTTTGCTTTTCGTTTGGATTGGTTTGTTTTGAGACAGTCTAGTTCTGTCACCCAGGCTGGAGTGCAGCGGCGCGATATCGGCTTACTGCAACCTCCACCCCGCCGCTTCACGCGGTTCTCATGCCTCAGCCTCCTGTGTACTTGGGATTACAGGCGTCTGCTACCGCGCCCAGCTAGTTTTTGTATTTTTATGCGAGACGGGGTTTCACCATTTTAGCCAGGGTTGTCTTGAACTCCTGGCCTCTAGTGATCGTCCCATCTCGCCCTCCCAAAATGCTGGGATTACAGGCGTGAGCCACCGCCCCCCTAGCCTAATGGTGTTAAAAAGTTAAGTTTCGAGAAAATAACACCTTCCTTTAGAAAGTACATTTTAGAGTATACAAAGTGAAACTTAAGGCCAACCAAAATAAGACATTTTGAGAACAGGCAGGGTGGGAATGTGACTTGGACTTAGAAAACAAAGGGCAAGGAAACTTGCTGTTCGCCAGTAACAAAATAGCATGGAATCTCATTCTCTGAATATAAGCGTTATTTCCCGACATGAGTCTGAACGTTTCTGGTGGTTTAGTGAGTGTTCACCAGCATTGATAACTTGCGAGACTGTCAGGAATGCAGAATTTCAAGTCCCACTCAAACTTACTGAATCGGAATTTACATTTTAAAAATCCTTAGATACCTTGTTATACACTCTGTTCTTTGGGACTGGATGAACTAGAATTTTAGACAATTTGTCGCTGCAGATAACTGAAACGAAAAGGACAGGATGGGCGGTGGGGCAACTCATCCAATAAGATTGTCTAGTAATGAACCAATCAGTCTGGTCACTCTTCAGCCAATGATTTTATCGCGCGGGACTTTTGAAATATTACAGGACCAATCAGAATGTTTCTCACTATATTTAAAGGCCACTTGCTCTCAGTTCACTACACTTTTGTGTGTGCTCTCATTGCAAA

>HIST2H3C.1_H_sapiens hg17_dna range=chr1:146625838-146626837 NM_021059 NP_066403 H3 1 bp downstream - 999 bp upstream -

GGGGAGAAAAACGAGCTCCTAGCCCCGCCGCAGCCGTCTTTATAAGCACAGTCTTTTCCCGATTGGGCGGAACAATAATTGAAAATCCCGCGCTGGCTGTCCATTGGCTGTGACGTCACCCGTCCTAAAGTCACCGGTTGGCTTGGGCAGATTCCTCCCTAATCCCGCCCACCCCGCCTCACTTTCTACTTACCAGTTGGCGAAGTTTCGCAAGCTTGTTTTTCCCCTTTCCTTCAGGGTGAAAGTCATTTTCCATTGCAGCTTTGTCAGATTGTTCCCTTTCCGCCCTTCGCTTCCTTAGAACACACTGCCCGCAATACCCCCCCTTTCTTGCTGCGCCCTACAGCTCCCGCGTGGAACCTCATTCTTCCATTTGCCCCCCGCCCCCAGTTGCTACTTGGCGGATCGTTTTTCGCCTGTCGGGTGCCTTTTGGGTGGGAGAGGTGCCCGCCCCCAGGACGCAAGATTCCGCCTCCGAGGCCCTTACTCTCCGCCGCCAGGGCGCTTTGGAAAACAAAACAGAAACTAAGACTTAAGTCAGCTCAGTGAAGCCAAGACCCAGCTCCGCCACACTAGCGAGCACTACCCGCTCAGGGCCCTCCCCATCGGGGGTGGGGACGAGGAGCACGTCCCGCTGGTGCCAGACTAGGTCTAAGTGTCCGGCCATAGCCTGCGGAAACCACTAGGGAAGCGCGCGCTCCGCGCGCAACACTTCAGCGCGTTGGGTACTGCCCGCGGCGCTGGTGTTTGTGACTGTGTGGAGCGGACCGTGGCGGCGCCCAGGACGTTTGGTGCCTGCACCTGCCCTGGGAAGTCCTAGGACTGGGGACCCACTCATCGAAGAGCCAAGGCAGTTACGTGCTCCAGATGGAAGACGGCGCGCACACACACACACCCCGCCCCGCCCCGCCCCGTCCCGTCCCGTCCCGTCCCGTCCCGTCAGCTCCAGGTTCGCTATTCACTACTGAGGAACGCTGGACTGAGAGTGGCCAGCATTAAC

>HIST2H3C.2_H_sapiens hg17_dna range=chr1:146636255-146637254 NM_021059 NP_066403 H3 1 bp downstream - 999 bp upstream +

GTTAATGCTGGCCACTCTCAGTCCAGCGTTCCTCAGTAGTGAATAGCGAACCTGGAGCTGACGGGACGGGACGGGACGGGACGGGACGGGGCGGGGCGGGGCGGGGTGTGTGTGTGTGCGCGCCGTCTTCCATCTGGAGCACGTAACTGCCTTGGCTCTTCGATGAGTGGGTCCCCAGTCCTAGGACTTCCCAGGGCAGGTGCAGGCACCAAACGTCCTGGGCGCCGCCACGGTCCGCTCCACACAGTCACAAACACCAGCGCCGCGGGCAGTACCCAACGCGCTGAAGTGTTGCGCGCGGAGCGCGCGCTTCCCTAGTGGTTTCCGCAGGCTATGGCCGGACACTTAGACCTAGTCTGGCACCAGCGGGACGTGCTCCTCGTCCCCACCCCCGATGGGGAGGGCCCTGAGCGGGTAGTGCTCGCTAGTGTGGCGGAGCTGGGTCTTGGCTTCACTGAGCTGACTTAAGTCTTAGTTTCTGTTTTGTTTTCCAAAGCGCCCTGGCGGCGGAGAGTAAGGGCCTCGGAGGCGGAATCTTGCGTCCTGGGGGCGGGCACCTCTCCCACCCAAAAGGCACCCGACAGGCGAAAAACGATCCGCCAAGTAGCAACTGGGGGCGGGGGGCAAATGGAAGAATGAGGTTCCACGCGGGAGCTGTAGGGCGCAGCAAGAAAGGGGGGGTATTGCGGGCAGTGTGTTCTAAGGAAGCGAAGGGCGGAAAGGGAACAATCTGACAAAGCTGCAATGGAAAATGACTTTCACCCTGAAGGAAAGGGGAAAAACAAGCTTGCGAAACTTCGCCAACTGGTAAGTAGAAAGTGAGGCGGGGTGGGCGGGATTAGGGAGGAATCTGCCCAAGCCAACCGGTGACTTTAGGACGGGTGACGTCACAGCCAATGGACAGCCAGCGCGGGATTTTCAATTATTGTTCCGCCCAATCGGGAAAAGACTGTGCTTATAAAGACGGCTGCGGCGGGGCTAGGAGCTCGTTTTTCTCCCC

>HIST3H3_H_sapiens hg17_dna range=chr1:224919761-224920760 NM_003493 NP_003484 H3 1 bp downstream - 999 bp upstream -

TGAATCCGAAACTGTTGGCCCCGCGGTGTCCTCTGCCCAGACCTCAGCGGATTGCTCGCTTTTATAGAGCTTGCCGCGTTCCCATTGGCTGGCCTCAGGTGGCGTGATGGCCCACTGCTCTCTGATTGGCCCACAGGGAACTCCACTCGGGCGCCTCTCCCTTATATTCATGCCTGTTGGTCGTGGCCACCAGAGCTGGCACCAGCATTGTGAAGGGTTGATGCGGGTAGGCCGCGGTGGTGGGGATCATCTCTCTCCAGCACAAGCTTCCTCATGTCTTCTCAGCGCTCCTCTTCTCCCTTCCCCATGACTTGGGTGGCAAGAGCCTTCCAGAAGCCAGCACATGGCATTCCACTTTTTTAAAAAATTAATTTATTTTTTTATTTTTTGAGATGGAGTTTCACTCTTGTTGCCCAGGCTGGAGTGCAATAGCGTGATCTCTGCTTACTGCAACCTCCACCTCCCAGGTTCAAGCGATTCTCCTGCCTCAGCCTCCCGAGTAACTGGAATTACAGGGATTACAGGTGTCCGCCACCATGCCTGGCTAATTTTTTGTGTTTTTAATAGAGATGGGGTTTCACCATATTGGCCAGGCTGGTCTCGAACTCCTGACCTCAGGTGATCCACCCGCCTCGGCCTCCCAAAGTGCTGGGATTACAGGCATGAGCCACCAGGCCCAGCTGGCATTTCACTTTTTTTTTTTTTTTTTTGAGACGGAGTCTCTCTCTGTCGCCCAGGCTGGAGTGCAGTGGCGCAATCCCAGCTCACTGCAAGCTCCGCCTCCCGGGTTCACGCCATTCTCCTGCCTCAGCCTACAGAGTAGCTGGGACTACAGGTGCCCGCCATCATGCCTGGCTAATTTTGTTTTGTATTTTTAGTAGAGACGGGGTTTCACCATGTTAGCCAGGATGGTCTCGATCTCCTGACCTCGTGATCCGCCCGCCTCGGCCTCCCAAAGTGTTGGGATTACAGGCGTGAGCCAGCGTGCCCGGCCGGCATT

>Hist4h4_M_musculus mm6_dna range=chr6:137576176-137577175 NM_175652 NP_783583 -

TATTAGTAAACAACCTAATAAAGAGACAGGAGAAACTACAGTAATCCGCCGCCGAGCAAGAGCTTATATTTCCTGGGAGCGGACCTGATTGAAAACAGCAAGGAGTGGGCGGAGCTCTAGACTCCACCCCCTGGCTCTTTGTGCTCTGCTGTCTCCCAAGAACCATAAAACCCTTTCGGTTCCCTCTCCAAATTCCAGGAACCCTAGCACATTTGAAAGTTTAAATAATATAAGGTAACAATCTATTTCTCCTCTTTTTTTTTTTTAGCACAAATAAAAACGGCGCATCATGCTTGTTATCTTCAGTGCACGCTGATTCGGTATTTTCTAAGCAGCATGCTTGTTTTTACACAACTTTAATGTGAAAATGAAAAGAAGCTAGCAATAGAAATCTAGCTTACTAGGTCTTATACCGAAGCAGTTTACATTTTCACGAGAGAGTCTTTTGAGGGGATAGAAGTGTTTTAGTGCCTTTCTTTTGTGGCCACGATAAGAAAATTATGCTAGTTCCCACTTCCTATTTGGTTTCAACTGTTTCTGCAGTGTATGGATTTTCTGCTCCTGCATTGCAAACACATTGGTATTCGCTTTTCAGCCAATTGTTCCTATAGTTACTTAGCTGAACTATTGTAACTTGTCATTTAGATCCGAGGTCCTTGTTGCTTGCGTTTAAAAGAGACCTGGTGACTCACATCTGTAATTTAGGCTCTCCGGAGGCTGAGGCAGGATTGCCAAGAGCTGGGGCCAGCCTAGGCTAGACAGACTCAAATACATTTCCCCTCCTCCCCACTCAAATAGCTTGTAAAGTACCAGAAGTCAAAAGCATGACTCCGAATAACAGGACTGTCCTTAAGTTTGTTTCGTTTTTAAAGACAGCAGGTTTTGGGCTGGAGAGATGGCTCAGTGGTGAAGAGCACTGACTGCTCTTCTGAAGGTCCTGAGTTCAAATTCCAGCAACCACATGGTGGCTCACAACCACCTGTAATGAGAACTGATGCCC

>Hist2h4_M_musculus mm6_dna range=chr3:95751166-95752165 NM_033596 NP_291074 -

TGAGGCATTGGAGGTGGAGTGCCACTAATATATGAGAGGATCGGACCAGATTGAAAACTGAAAGCGCGCGGGCGGGAACCTTTCTAAATTATCCGGGCTCTGGATGTTGCATCATCTTCCTGTTTTTCTTTTCTCTTCTCTGCCCTCTACCGGTTCCTTACAGTCTCAAGCCTTTATTTTTTTATTTTCGATTTTCTTAATTATGGTGCCGAAGTCCCTCGGAGAATTCAGATTCCTCCAAATAAAACTTAGAGATAACACAGACTTTAGAAAGCACTGACGCTAAAAAGACAAAAGACCGGAGAAAGAGAAAGTTAGAGATGAGGGGGGAGTGCGCTGCACTGAAGGGCCCGGAGCTCCTGTTAGGTAAAAACCGCAGCAAAAGTTCCAGTAACCAGCGTGACAATCGAAAGCAGAGGACCCAGGAATGTGTGTGGAAGGGAAAGGTCGCGGGCGTTTCTGCGTACAGTCGCCTGTTTCGTTGATAGAGACACATTATTCAGATCAAGCAGAAACTAGATCATAGGAAAACTGAGGATGCCGCCGATCCTTTTCACCGAGGTCAGTGGGTTTCCACGCTCTTCTACCCAAAGGCCCGCTGACTAGCCGAGCAGCCCGGGAATGCTGCTACGCTCGTTTCCACAAGTCCTTTGAGGCCCGCCCTTTGTTTTCAGTTATTCCAAACTGTCTCTACTATGCAGTAGACTAAAATTCACTTTTTCAAGTGTGTGTCTGCTTTATACAGTGACGCCCTGGGAAATGAACTGGAGTCTGTACTTCTAGTATGTCCGTCGTTCCCAGGGCCACCCATAGAAGAATCTTGAGAAAGCATTCTGCATCAGAAAGAACGGTAGTGGGTTGGTTGGGACTTGTTTTTAAAGTGATCTGATAGGCCTTGTTCTGACTGTTCCTTTATTCTAACATCTATATGTGCTCATGAATCCCCACCACCACCACCACCACCTTTTTTCTATCCAGATTTTCTGGCTCCAGACATGTA

>Hist1h4c_M_musculus mm6_dna range=chr13:23178327-23179326 NM_178208 NP_835515 -

TGGTTATGACAGCAGGGAGACAGCAATCGTCCCTTTAAATAGGCCTTCTGCGGACCTGGTTGAAAACTAGAGCGTTAGGCGGGGTTTCCGAATCCGGGTCTTGGAAGGGGGTGGAGAAAAAAGGATGCGTCATTGGAATCTGAATGTTTTCATTGGCTAGCAAGCCCCAGCTATTCTTTGCATGCGGTGTCTCTTAAAGACCACATTTAAATCCTGCAGAAAAACTTCATTCCCTCCTCCCCACCCCGACCCCATTCTTTCTCATGAAAGAAAGGCTTATTGGGGCCATTCCACAAAGCAGACATCCCCATTTCTCGTTTGCTTTTATTTCTGTCTTGTATTAAATTAGTTGATGGTTTCTTTGTGAGGACTCAGGTTAGATGTCCAAAACAGAGGTAAAATATGCCAAGACTGTCCCATGTCAGTAAAGTTTAATCTATTCCCGGCAGTTTTGTTTGGGTAGACATAAACATTGAAAAGGCATCTCAAATTGAGCACCCTAACTGCAAACTTCATATTCCCTATCTCGAGTCAGAGACCTAATGATTCCGTCTATCCTCTTTTTTAATAAAAACTGAGAAAGTCTACAAAAGAATAGTTAGGTTATCGGTTATCCTCTACCTGCTGTGATTCCAAGTTTGTTTCCCTTCCAGCCCTTATTAATCACAGCATGGCTTGCTCACCTTAAGAGAAGAAGTGTTTTGTAGAGCTGGTAATTACAGTACAGGGCTAATACTTGATTAAGGTCATTACAAAGTCTACAAGGTCCTGGTGAAGCTTGTGATATGATTAGTGATGTCAAAACAAGGCTAATCCGACCCCCGCCCCCCTTTTTAAAAAAAGGCAGAAGAGTAAGTAGCAGTAAGTCTCGATGCGGGTTTAATGTTGATTTTGGCATTCCAAATTTTAATTTAACAATGATGTAGAGGAGATGGCTCAGAAGTTAAGAGCATTTGATACTCCCCCAGAAGGCAAGATTTGGTTCCCAGAGCCTAAATGG

>Hist1h4a_M_musculus mm6_dna range=chr13:23241035-23242034 NM_178192 NP_835499 -

TGTCTTAGGAAGAATGGAAAATGGAGTCAGAGCTGAGAGTCGGTCCTTTATATAGCTGCTTGGCCTTGATCTCCCACCCCCAACAGAATCACACAAACGAAAACCCGCCCGCCGGACGGTGACGTCATAAACCCCGCCAATAAGACCTGGCGGGAAAGAACCGAAAGCGCCCGGGCGGGAAAGTGACGTCACAAATCCCTCCCATCAACCATGCCCCGAAAGCGCGCCGGCGGGAAAATGACATCACAAACCCCGCCCCTCAGCCAAGACCCGAAAGCGCGCCGGCGGGAAAGCGACATCACTGATCCCTCCTCTCAGCCATGCCGGGAAAGACGCAAAAGCCCGCCGGCGGGAAAGTGACTTCACAAATTCCGCCCCTCAGTCACGACCAGAAAGCGCGCCGGCGGGAAAGTGATGTCACATATCTCGCCCATCAGCCCCGCCCCGAAAGCCGGACGGCGGGAAAAGTGACATCACAGACCCCTCTCTGGATCGGAGCCCTATAAGGACCGAAAGCCTGCGAGTAGGTCACGTCAGAGATCCGACCTGTACACTTTACGCATATTCCACGGGTAAAACCAGACAATCAGTTGACCTATTTTGTACTTGATATCAACTTTGGGCTGTTATAAACTCAAGTTTTGGGGAATATCCACCGTTATAGACACTAGGGAATATATAGGTTCGTTTAAGGATGGAGTAAATTACAGCCATTTTACTTGAAATCGTGTGTGGCTCTGAAAAGAGCCTTTGGTTAATTCCGTAGAACTGTACGGAACTGTACAATTACGCCCTCTCCCCGCGGATGCGGCGGGCCAGTTGGATGTCCTTGGGCATGATGGTGACACGCTTGGCGTGGATGGCGCACAGGTTGGTGTCCTCAAACAGACCCACAAGGTAGGCCTCACAGGCCTCCTGCAGAGCCATGACGGCCGAGCTCTGGAAGCGCAGGTCGGTCTTGAAGTCCTGCGCGATCTCGCGCACCAGGCGCTGGAACGGC

>Hist1h4f_M_musculus mm6_dna range=chr13:23031712-23032711 NM_175655 NP_783586 -

TGGCTATGGAGCACTGAGTTGAAATTCCTTAAGCGCCCTCTACAGCTGGTATATATATGGCGAATGCGGACCTTTTAGAGGACTGAAAAGGCGCGGGCGGGAAGGTGATTCTGTATCCGCCCCTCTAACTCACTGGCTGAATCCTATTGCTAGGCGGAAGGCACAGCCCCTGTGGCGAGGGGAATATGAGTTTTCCGCTTTTCTCTTGGAGTTCTTAGTCACTAGTTTGTGTCCTTCAGGAGCGTACTTGTAGGTTCTGACAACCGATAGTTTATAATTCTCCCAGCCACTCATCATTTTTAAAAAGGAGGTTGTTATCCATACACTTATCTACAAAACCGGATATAGTACAGTAAGGTTATCTTTTAGGTGGAAGATTTACGAAAATGTAACTTCTAAAATTTTGCAAGCATTATTAAATCTCACTTATGAAGCTCTTTGCATTTCTCAGAGGCTTGCACATAGCTCTGTTTTCAAGTCTACAAAGGGCACTAGTATTGATTGAATATATACTGCTTCCCTTTTTTTTTTTTTTTTTCTCGAGACAGGGTTTCTCTGTGTAGCCCTGGCTGTCCTGGCACTCACTCTGTAGACCAGGCTGGCCTGGAAAGATGGAGACTTGCTCAGAGTTGAAGATCTAATCTTTTTAATAAACGCTATTGTTGCTGGGGAGGACTTTTGTGTTTCATGACCGGTAGCCACCGTTTTCTTTTTAGCCTCTGTAGTACTGGTTTGAGCCCCATCCTTGTGTAGAGAGAGGCCATCTTGAAGATGTGCTTTGGCTATTGCTTGTGTGAGACGTTTCTATGATAATGGTAGAGAAACAAACAAACAAACAAAAAACCCAAAAAAACAAAAAAACAAAAGTAACTACCATGTACTCAGGATGTGCTTATCCCAATTACCCCACAGAACTTTTCTATGCTTCTTTCCAGAAAATCACCAGTCCTCACCCCACTCACTCTGTACATACCAGAATGCCAAAACTAGTGTGAGTACA

>Hist1h4k_M_musculus mm6_dna range=chr13:21230570-21231569 NM_178211 NP_835583 -

TGACTGCACTCAGGACAGCTCAGAACAGAGATGCTGACAGGGAGCCTGAGCGCCGCCTTTTTATACTGCGGCGGGCGGACCGAAATGAGAACCTGCAGGGAAGAGGCGGGAAGTTGCGCCCGCAGCTGTGGGGGAGGGGAAGACTTACCAAAAAGAAAAAGGGCGCCTTTTGTTTCCCTGTCCGAGCTTTCTGAGGGAACTAGCTCCTCACGCCCTTTTAAGAGTTTGGAGAATCGAGCGCACAATCAGTACACTGACTGTACAAAATACAGAAAGCCAAGCGGCCCCTTAACAACCCGTAACCCCGTCCAGAGAGGACACTGCAGGCGGCTGCTAAGGCGCTTTGGTGCTCAGTACACACTTTCCAACGTGCAAACCGTGCTCTGAATTACCTTGTGTTCCTTCTCTATTGTAACCACATTTCAAATGTTTTTTGTTTTTGGTTTTTTGGGTTTTTTTAGTTCAAACTTACAAAAAGCCTCATAATACCTGTATTATTCTCTTGAGAACTTTTCATAAAAGCACCAATCGGTATTACTCATCTGCATCGCTTGGTGAATCATTTTAAGTTTAAAGTCATTTTTCACCGTTAAGTGAATTTTTCATCACTATACTTACGGTCACTTCTGATATTTCTATTGTGACCATATTAAATTTAAGCTTATCTATATTGACATTTCATCGCTCTTATTGCCAACCTCATTTCTCAAAGATTTTTTTTATTTAATTGACTTTCACTAATATTTTCAAGAAAATATCCAAGTGGGTTTGTGAAGTCTGAACAAAATATTTGAAGACAGGGGTTCATCTGTGACTACTATGCTCTGTACTAATACTCTCTGTACAGTACAAGTATAATGAAAAACACTTTTTTTTAATCTCCTGAAACTTTTCCCCCACATCTACTAAATTAGGATCTGTCAAGAAAATTGATGGAAAAACTATATTTACAAAAACTGGTTGATTGACAGGAACTACATATATGAGAGGTATGAAAACA

>Hist1h4m.1_M_musculus mm6_dna range=chr13:21292158-21293157 NM_175657 NP_783588 -

TAATTATGCTCACCTAGAGCTTCTTTCTCAACTAAAGGCCCTTTTCAGGGCCATCCAATTAATCCTCCAAAGGGTTGCAACATTGCAGTACGGGCGGGCAAGTTTTCCTAATGAGGCTCTTTCCAGCTACTTCTCGGGGGACGGGGGGGAAGGCGACGAGGGCAGTCTTTTTCAGGGTGAGCCCTCCGTCGTGTCACGTGTGTCCTGGAATTTGGACGGCTCCTTTGAGAGGAGGCAGAGCTCAGGCGCTGAGCAGTAGGAACTCCCATGCCCCGTCCCACCCCTGCCTCCTACGTAGAGGTTTGCTTCAGGACTACTGAAAGACCCCACCATAAGGCTTAGCAAGCTAGCTGCAGTAACGCCATTTTGCAAGGCATGAAAAAGTACCAGAGCTGGGTTCTCAAAAGTTACAAGGAAGTTCAGTTATAGATTAACAGTTAAAGATCAAGGCTGAATAGCACTGGGACAGGGGCCGAACAGGATATCGGTGGTCAAGCACCTGGGCCCCGGCTCAGGGCCAAGAACAGATGGTTCTCAGATAAAGCGGAACCAGCAACAGACACAGAAGCCCCGATAGACGTCAGTGTTAGCAGAACTAGCTTCACTGATTTAGAAAAATAGAGGTGCACAGTGCTCTGGTCACTCCTTGAACCTGTGTGTCTGCCAACGTTCTGACCAGATATGTGCCCATTGCTGAACCTTCATTAGACTCTTTCCTTGTACCCCTCCCCTACCCATTTCTTGAAAATAGACATTGTTTAGATCTAAAAAGTCCCACCTCAGTTTCCCCAAATGACCGGGAAGTACCCCAAACCTTATTCGAACTAACCAACCAGCTCGCTTCTCGCTTCTGTAACCGCGCTTTTTGCTCCCCAGCCCTAGCCCTATAAAAAGGGTACAAACTCCACACTCGGCGCGCCAGTCCTCCGATAGACTGAGTCGCCCGGGTACCCGTGTTCCCAATAAAGCCTCTTGCTGTTTACATCCGAATCGTGGACTC

>Hist1h4m.2_M_musculus mm6_dna range=chr13:21312223-21313222 NM_175657 NP_783588 -

TGATGAAGACAAAACTGTACTGCTCTAAGACGAAGCAGACGGCCTTCTGGAGAAGGCCGGCCCCTCTTATAGGGAACTTGCGGACCGGAGTGAAAACAGGAGCTCAGTGTAAGTGGGTGTGGCCGGGGGAAGGGCTTAGAGCAAATTTAACACAATAGGTTTTTATATGGAAAGGAAGCTAATTTCATCAACTCCCTGCACTACAGGTTTTCGTTAGCAGTTTTTTTCTTTTTTTTTTTTTAAACTTACGTGCATCAAACAACGCAAGATGCAGGTGCGCACTCATACGTGCACGCCTTTACCTTGTTCATTAAATGTGATCACTACGTCCTTATGGAACAAAAGTTATAGTATACAAAGGAAAAACATGTCGCTTTTACAGTAACACTATGTAGGAGTAAGTACACCAACTCTAAAGACGAGGTTCTAGGTCTGAATAATGTATGCCCCCTCGCTCCCAACCACATGGCAAATGGCGGTGATGGCACATGCCTTTAAACTCTACACTAGGGAGGCAGGGGCAGGGGCAGGGGCAGGCTAATAGGAGTTGAAGGCTGGACTGGCCTGGAGTTCTAGGACATCTAGGGAGACTCAATCCCCATCTGGAAACACGAAAGGAAAACAAGACACGGAGCTACTTTTCATGCTTACAAAGATCCCTAAGCATGAAAAGTGGCCAGTTTTCAGTACTTAACACCGTAGTTCAAAGGGAAATTAGTTCATTTATATTGGGTTCATGTATGCCAGTCTCTCTTGGGCAGAAACCACAACGTTTGTCTTCAATTCTGGTTCTAAAACCCTCACCTCTAAACCGCATACAAAATGACATCGATTTACGTAAGTATTCAGTTCTTTAAAAGACACTGTGGATGGCTCTGAAAAGAGCCTTTTTTTTTTTTCTTTTCGGGTGAGTGAACGGCTGGTCTTATTTTCCCTTGGCCTTGTGGTGGCTCTCGGTCTTCTTGGGCAGCAGCACGGCCTGGATGTTGGGCAGGACGCC

>Hist1h4h_M_musculus mm6_dna range=chr13:23010116-23011115 NM_153173 NP_694813 +

ATATCTATAGGGACTGATCAATGAATGTATTCCTACCACCAGACCCTTGAAAAATAAGGCTTTATGAATTATGTATAGAAGAAACTGAGCTCTGTTCCTCCACTCTTACACTTGTATTCACCTTCGTGTAGTGATTTTAGGTTTTATTGGGCAAAATAAATCTGACTTATTACCAAGAGAACACATGAGTTACCACTTGAGCGTCCATGTAAAAGTTCCAGTTCCGTTTTGCAGAGATCGATTTACCTCACGAACTTAACTTTAGGAGGAGGGAAAAAAGAAAAAAAAAAGTCCAGATAGCAGAGGATGGTTTCGATCCATCGACCTCTGGGTTATGGGCCCAGCACGCTTCCGCTGCGCCACTCTGCTACTTAAAGAGAGCCTTTCAAAATATTATACTTAACTTTATTTGCAGTTGGTGTTTCTCTACTTCATACAAAGGCCTGCAGGAAGGCACCTTCTAATTTTTGTCTACCTAAAGGCTAATATTTGTTGGGAATAATGTCGCTAATGAGTCAAAGAGAAAGCAAAATTTGGACTAGATCAGTAAACATTAACATACTAGTAAGTAATTCGACAAAGGAGAAAACAATACAGCGCGTCTTAGTGACCAACCACGCATTTACCTGCGCGAAGAGTTTGGGATCAGGTTTGTCCTTAAGTCGAGATAGTGGAAGAAATGGTGCTATTTTCTCCAGTGTTTAGTCCTTTACAGCCGAAAATAAAAACATGGGAAGCGAGACCTCCACATCAGTATTTGTGATCGCTTGGCTCACCAAAGATCTCCTGTCTTTCTAAATTTGTTACAAGTGACAAATATGTATTGCTAAAGAAACTGTTTAGTCTGAACTCGTTAAAATGGCCGGGAAGTCCGGAATGGGGACAAAGGACCCAGCTCAAGGAGCGAGTACAACGAACGTTGGTGACGTCACTAGGCATTGCCCATGCCAGGGGCGGGGCCATTGCAGGGGGTTTCAGTCTTCAATCAGGTCCGAAAACA

>Hist1h4d_M_musculus mm6_dna range=chr13:23060481-23061480 NM_175654 NP_783585 +

TATTTACTGTATTAGCATTTTATGGAAGTATTTGGTTAGATATTAGTGTGTGACTCTTCATTAGTACATGAAACCACCAAATCCTTTGACTAAGAACTGTCAGTGTGAGGTGGGGGTGCATGCATGATATTTTGAGGTACATATAACTATGATGTGCTAAGGATAATAGATTTTCTGTGCAATGTTTATTAACTAAAAAACATACATGCAAGAAATCCTGAGATTCAGTGAAAGCATAGTAAACATAAAGAGCAGCCCCTTTCCCCCATCCCTGTTCCCAGAGCTCCTGATGTCTATGTGTAATTCAAGCAGAAGGCCCACACCTTTGGGATGCATATCCTTTACATCTTCTAATCTTTCAGCGCTACAGTCCTGTAACATTTTGAGGTTTTACTTCCCCCTGGTCAGCCTGAATGCTAAAAAAAAAAAGTTCAGATTCTTAGGCATACGTACCTAAGTCAATAAAAAGTCTCAGAACAGGAAATAGTAAGTGTTCTTAAAATTGTACTTAAAACCTTGGATTCAGTGGTCCTAAACCTTCCTGATGCTGGGACCCTTTACTACAGCACCTCATGTTGTGGTTACCCTCACCCATAAAGTTATTTCACTGCTGCTTAACTGTAATTTTGATACTGTTATGAATTGTAATGTAAATATCTGAAATAATACGGGGGGTGTCCCGATTTATAGGTTCACGAGACTAATAACATTACTTTCTATTTGGTCTGATAAAAGACAAATTTCTTTTTTCACAATACATAAATTGTCACTCTTGAGGGCAATGTGTTGAATTTTCAACTGCTGGCTCTGCTCTTGTCTTAGGACCCGCCTTGTCTCCTCAGCCTTTTCATAGGATAAACGCCAGAAGGGCGGGAATTGACCATGGTTCACGCCCCCTTCCCTTCAGGTTTTCAAACAGGTCCGCATTGGTATTATAAAAGCCAGGAAGGCCGCTTGTTACAGTCACTGGCTAGTGAGCTTCCTTCCTAGTTTGCTAACA

>Hist1h4b_M_musculus mm6_dna range=chr13:23235900-23236899 NM_178193 NP_835500 +

CCAGGAACCGCACCACCTACAATGTGCAGGACCCTTCCCTCATAAGGCACTAATTAAGAAATCCTCTACTGATTTGTCTACAGATTCATTTTATGGAGGAATTTTTTCAAATGAGGTTCCTCCTGTATGATGGCTCTAGTTTGTGTCAAGTTGGAATAAAACTATCCAGCACAAATAGTAACAGGTAAAAATATTTATGTAGTACTTCTTTGAAACAATCTCTTCTAATGTAGTACCCAAGCAAACAAACTGTGGGCACCATTATCAGACAAGTTTTATAAGTATAAAGGGGGAACAGGAAAATGACCAGTTCCAATATCTCTGAATTCATTACTTTGAACGAGAGCATACACTGTGTAGGGGTGGCAAAATAGAATACAAGCATCATGCAGTTTTCCCGCTCTAATAAAAAATATAGCAATCCAAACCAGCAATGAATGGATTCCAGACAGGGAATAGCCAAGTGCATCTATATGACTAGGCAAAGGGGCTTGACCTATGTTGACAAGTAGAATTTTAATAAATTCTACTTATACCGATTCTATTTCAATTTCTATTAAAAAAATTCTCTTGTAATTTATTTTTAAAAGTATCTTTCACATTTGAATTTTTAGGTTTTAAGAAACAATAATCACATGGTCTTTTGGCACCTGTTGAATTTTTTAAAAAGATGTTTTAAACCTAAATACTTTACAGGGGTATTTGTTTTAATCCTCCAGGTACTGGTCCATAAAAATGTAACTCCTTGCTAAAATCCTTATAGAACAATATAACATATAATAAAAGCAGTATACTCAAATCTGGGGAAACCTGAGAGACTCCATGTCTGCATGTGCCCTATACAGTACTCCAGAGTCACCGCCCTGAAATGGGCTGAGCAAAAGAAGCCTTTGGTCCCCGCCCACTTGAAGTTCTCAACCAGGTCCGATAAGAGCCTATAATAACACAGCGAATTCTTAGTTCACCAGCTTCCTACTGAAAGCTTGTAAGAATTTAACTA

>Hist1h4j_M_musculus mm6_dna range=chr13:21214162-21215161 NM_178210 NP_835582 +

TGACGGTTGCTCGAGGTCTGAGTCCCTGTATGTCTGAAAATGCTGCAACCTTGGCCTAAGCCATCGAGCACTACAGACGGAGGGACAAGAATTCAGTTCCCCCAAAACGTGAACAAAGGGAGTCCAGACCATTCTTAAACCAGCAGAAAATTTCAAGATCACGAAGGATGTTATAGTCAAGAGTGCCCTCTAGTGGCCATCCAGACGGGTCAGCGTGTACTGAGGGCAGACCAAAGAACAAAGGAAAGTCAGGCATTTTGGATTCCAGCCCACTTTGGATAAATTTTGAAGTAGGCACCCCAAGGGCGACTGAGATAGTGTTTAGACCCTGTCTTGCCCTTTTCCAAGCCAGGGTTGGTTGCCAGAAACTCTTAGGTGAGGGTGATGACATGTTAGCCTGAAGTTTTCTCCAAGCATATGGGGTTGAGGGAAGGACAGCAGACAGGGTTTCCCAGATTGTGTGGTGTGAGCAGGGGTCCAAGCAAACTGTGAACTGAAATCGGCTTAAAGGTTTCTATAAATTCTTATAAACATATTAAAATGGCCTTTTGGAAGTTTGAACTAAAAAAAAAAATTGAAATGCGGTTACAATAGAGAAGGAACACAAGGTAATTCAGAGCACGGTTTGCACGGTGGAAAGTGTGTACTGAGCACCAAAGCGCCTTAGCAGCCGCCTGCAGTGTCCTCTCTGGACGGGGTTACCGGTTGTTAAGGGGCCGCTTGGCTTTCTGTATTTTGTGCAATCAGTGTACTGATTGTGCGCTCGATTCTCCAAACTCTTAAAAGGGCGTGAGGAGCTAGTTCCCTCAGAAAGCACAGACAGGGAAACAAAAGGCGCCCTTTTTCTTTTTGGTAAGTCTTCCCCTCCCCCACGGCCGCGGGCGCAACTTCCCGCCTCCTCCCTGCAGGTTCTCATTTCGGTCCGCCCGCTACAGTATAAAAAGACGGCGCTCAGGCTCCCGGTCAGCATCTCTGTTCTGAGCTGTCCTGAGTGCAGTCA

>Hist2h2aa1.1_M_musculus mm6_dna range=chr3:95728126-95729125 NM_013549 NP_038577 -

TACTCCGGCGCCGACGCCTCACCCTTTTATAGACGCAGACCCGATTGTCCGCCCGACGCTCTCATTGGCTCACACGCGTCCTGACGCCGTGGCCAATAGGAGCGCGCGGACAGAAGCCTCTCATTTACATAAGACCGGCTCCCTCGCCCCGGCGCCGCCCTGGAACGCGCGAATCACGACGCGCGTGCTCGGTGGCTTCATTTGCGTGCGGCCGTCGTTGCGACACTCCGCGGGTCGCCGGGCGGCTGGGCTTGCTCGCTGTTTCTCCTGCTCTGGAAGCTGGCAAAGTGTGCCCTGCATCCAAGAGGGCCTGAGTTAATCACTTACTTAAGTGTAGGTTTTCCTTCGAGGAATACTGGAAATTTAGTTTTTTATACCTAAGGAGTAGCGGATAATTCTCATAGCCTCTTTATTTTTCTACTTGAAACAGTGTAAAACCTACTTGTTTCATTTTAAGCGTTTATCGCTGGGAGCCTTACTTTAAAATCGTTAAATTTAAAGGTTATTTTCTTAATCCATACACATCTGAGAGTGCTTGAAAAGACCAAGTACAGTTCGCCTGGGGTTTACTAAAATTATTTTTTTGCCACTCAGATTTGTACATAAGGAATCGAAATAACTTAAAAAGTAAACCTTACGCTGCTGCTTTTGTCTATCATTTGTCCTGATGGCTCTCAGAAGCTCCTTTTAGGAGCCTTCCAGGCTGTCTGGGGCTAGAGTGACTCGGAGTACCATATCCGGACTGCCATTATTTTAGCATCGCATCAGTTATGCTCGGTAAAATACAGCCAGCATTTTTCAATCTCTGAAATTTGTAATAAGGTTCGTTATCGATGCATTAGAATCACTGATTAGGCATATTGAGTGTAAGCTTAGCTCTAGTAACAGGTATATTGTTACATAGTAACATAGTTACAATCTTTATATTTTACATTGTTACATATTGTGGAGATGGAATTCTAGTCTTGTGGAGGAATCGGCAAGAGTGTAGCACTAGGAT

>Hist2h2aa1.2_M_musculus mm6_dna range=chr3:95732372-95733371 NM_013549 NP_038577 +

AAACAAGCAACAATGTCTGTGTGGCTAAACAATTCTGATTGAATTTCTGGTGTGGTGCTTAAGACCTCGTATATAACTAGGAAAGTACTGTGCCATTGACCTACATCCCAGAATATGCCAAACTACAATGAAATAACATACAAAGTGCCATTAAGACTTCAATCTTTAACAAGCTGTACGTAAATCCCTCTTAAATCTTGTGACCTGAAACAGGAAATTTAGTCACCGTATGTAAACTGAAAACTGCACTAAAAAATAACAAGATCCAAGTTAAGCAATTTCTGACCCAAAAGACAAGTTACTTCACAGACGCCTCCGATGCTGATACTTTAATAAATGGACTGCCTAATGCTTTCCTACATTCTGACAATATATTAACACTAATTTTATGTAACTCCCCTGCAAAGTCGAAAGATACCCCTACACTTAATACTCCTCATTCAAGGGCACATGTAACTTGAGTAGAAATGATTTCTAATGTCAATGGGAGGTAGAAGAGAAAAAAACAAAAAAACAAAAAACCCAGGAAGCAGCAGAGACACCGTGGGTGGTTTCATACAGGAAACAGGGTAAGGCTGCCTGGCTCTGCCGGGACGACTCGCCAGTTTGGGCACAAAGATGGAGCTGAGGAAAGAACTCACAGCCTGTGCCAGTCACCTACACCTGGTACACCATGCCCTGTGGCCAAGTTTACGCGTGCTACCGGCTCTATTCCTGTTAGCTTCCTCAGCAGGAGAAACAGCGAGCAGGCCCAGCCGCCCGGCGACCCGCGGAGTGTCGCAACGACGGCCGCACGCAAATGAAGCCACCGAGCACGCGCGTCGTGATTCGCGCGTTCCAGGGCGGCGCCGGGGCGAGGGAGCCGGTCTTATGTAAATGAGAGGCTTCTGTCCGCGCGCTCCTATTGGCCACGGCGTCAGGACGCGTGTGAGCCAATGAGAGCGTCGGGCGGACAATCGGGTCTGCGTCTATAAAAGAGTGAGGCGTCGGCGCCGGAGTA

>Hist1h2ao.1_M_musculus mm6_dna range=chr13:21290250-21290532 NM_178185 NP_835492 +

CTCTGGAACTCTAGCACGGAAGCGACTTGGTCCTGTCTATTTGATGAGGTCTTATTCAAATGAGGCATGGGATCGGGCGCCTATGATTGGTTCATGATATACATGGCGACATCCGAAGTCTTGTCCAATTGACGTGCGCCCGCGAATGCTTCCCATTCGTCTAGAAGAAAAGAAGAAGTCAGCCAATCGCATAGGCCCTTTTTTGCGCCCAGAGAAAACTATAAGGGCCAAGCTCTTGCTCTTTGCAAGCGTACGGTTTTCAGAAGCTGTCTACTTTCTACCA

>Hist1h2ao.2_M_musculus mm6_dna range=chr13:21313540-21313820 NM_178185 NP_835492 -

TGGTAGAAAGTAGACAGCTTCTGAAAACCGTACGCTTGCAAAGAGCAAGAGCTTGGCCCTTATAGTTTTCTCTGGGCGCAAAAAAGGGCCTATGCGATTGGCTGACTTCTTCTTTTCTTCTAGACGAATGGGAAGCATTCGCGGGCGCACGTCAATTGGACAAGACTTCGGATGTCGCCATGTATATCATGAACCAATCATAGGCGCCCGATCCCATGCCTCATTTGAATAAGACCTCATCAAATAGACAGGACCAAGTCGCTTCCGTGCTAGAGTTCCAG

>Hist1h2ab_M_musculus mm6_dna range=chr13:23229967-23230966 NM_175660 NP_783591 +

CTTTCATCTATGTTGTCTCCAGTCACCAAGACAGCAACGCCTTTCAGTGCAAACTCTTCAGGACCTCCTTGGCCTCATATATTCCAGGTTCTCCTGGGTCAGTGACGGGGCAGGGTCTCCTTTTCCCCAGTCTCTATTTTCCAGGTAGTTTAAGGATAATTCAACCACGATAAAGAATAATTTGGCATGCTTATACTGTCTTTGATATTGGTGGAGCTAAAAGAAAGCCTGCCATAGAACCTAAAATTATGTACCTAATCCGTTATGAGAAGCCCACCAAGGATGACCATTCAGAAACAGTTTGTAGCTGATTGAAATGATTTATTCCTTGTATTGAGGATCACATCCTGGTATCTCACTCTGCTGCTAGAGGGAGAATTTTTTTCTGTTGTTGTTGTTGTTTGTTTGTAACAAGCAGTTTAAAAGAAGCTACATTAGCTAGGGAATCTTGAACTCTGGTTCATAAAACAGTTGGGTAATATTCTATAAGATTCTACGTCAAGATCAAGAAGTAGTTAAACCTGAAATGGCCTTACCAGGAATACAAGATGGAGGAAGTGCCACACTGACTTTCCTTGTCATAAAAACACAATTCTAAACCCTGAAAACAGGACAGTAGCTTAACAACTCAGAAACACTCCTACTCTATATTTCAGAGTTTCTAAAACTACAAAGACCTTGATGAAAACAACAAGAAAATCAGTCAGTTACTTATTTCCATTTAAGTAAAAACTGGAAAAGAAAGTAAAAAAAAGTTCTTAACTATTAATCATGAAAACCAGAGTTTAAATCCCAGAATCTACGTGGTTTTTATTTTTTAAAGAACGATATGAAAAAGGACTGGATGCGAAAAATAAAATGCAGGCCTTTATGCAAATGAGGAGCTCAAATTCTGGCTTTCTATTGGGTACGATATATTAACCAATGGGAGAAACACAAACAGAATACCTCCAGTTAGTATAAATGCTTGCTGTTCAGTTGCAGAATTTACTATATATTC

>Hist1h2be_M_musculus mm6_dna range=chr13:23101002-23101650 NM_178194 NP_835501 -

CACATTCATTTACATTACGCAGTATTCAAATGAGGTTAATAAGATTTCTTTCAGGATTGGGTTAGTTTTAGTGTTTCGTCACGAACAGGAACGTAAACGACACTTGAAGGTCTACTGTCTCATTGGCTGTCTTGTCACTTCGCTTTCACCAATCGGGACGTACGAATTAAACTGCACAAATCTGCTATAGCTAACCTATTTAAGCACGATCGCTATCAAACATGGGGGAACACAGAAAAAAAAGTACTCTAGTTGGGTTTGTATGCTTGATTTCAGCAGTCAGAACTGAGATGAGATGCGGGTGGGGTTGTCAGGACATCGATCCTGCTCTTTACGCACTGAATCGCTCAATGCTTAAGATTCTATCTCTAAATCATTCTGCTATCTCCCTTAAAAGTTATCGACGTGGCAGCCGGGAGTAAACTACTTCATTCCCATGGTCTCTAAGGGTCACTTGTGGGATATGAACTTACCCACCACCACCACACATACAGGAGATTATGTACAGACCTTCAGATAGCTAAGCTAGAGTTAACACCATTGCGCACACCCACATATCCCCAGCCTCCTCTGCTAAGCTCAGCCCACTAAGGTTTGTTCCCACCCTAACCACACCCCCTGTGAATGTGCAACAGCTCTTTTACTGAGA

>Hist1h2bm_M_musculus mm6_dna range=chr13:21201880-21202163 NM_178200 NP_835507 +

AAATGCTGTGTACAATAAAAGTGCTGAGGCGGAAGAGTAGCTCACTCTTTGTTATTGACTGAATGGCGCTAAAAATGAAGACGTAGCATTGGCTAAGGGTCTAATATTTTAACCAGTAGAAACACAAATTTGAACAATATGCATTCTCATTGGATAGGTGTAATTTTAAACTAACCAATAAACATGCGACTATCTCTCGTCTCTATTTGCATAACAGCGTGTATATAGGGCGTTCACTTCGCCTCCTCTATACAGACAAATTGCAGTTTATCTGTGTTGTAACA

>Hist2h2bb_M_musculus mm6_dna range=chr3:95756940-95757533 NM_175666 NP_783597 +

GCCCTGTTTCCCTTCCATCCCCCACAAAGGCTCTTTTCAGAGCCACCTACGTTGTCGATGAAAGTAGCTGTGTCAGCTTTGCAAATTGATTTTCAGTGTTTCAAATACGCCCTCAAGTGTGTCAGGCCTAACAGTTACCAGTAATCACATTCCTTATCCCAGGTTTATTCCATTTAGAGCTGGTCTTGAGCTCACTACTCAAATATGGGCCAGTCCAAAAAAGGAGTTCTAATTAGGGTCTCAGAACTGTGAACCTGAAAAATAGCCCCAAAATTCCAAGCGTGATACCTAAGAGCGATAGTTAACTTATTTCAACCTGGTGTTTTAAGGATCTGTAAGCGAAGGGAACATAAAGAACAGCTGAGGCAAAACTGCCGCTCTCAATAACAGGCTAGCATGAAATGGAAGGCTGCGATTGGCTAGAAAGAATTTCCTCTAAGCACCACCAAGGCTCGCAACCAGAATCGGTTTGAGTAGGCTCCGCCTCCTTGACGCTGACTCTGCTGTAAATCAACCAATAAAAATGGAATACCGTGTGAAACCTCATTTGCATACAAGCTCTATAAGTACCGCATAGCAAGCCGCGCTGAAACA

>Hist3h2ba_M_musculus mm6_dna range=chr11:58673542-58674541 NM_030082 NP_084358 +

AAAATGCATGTCGACGTGAATAGAGCTCATAGAAATTACGCACAAGTATGCACAGGGCACCTTGCCAGCCTCTTTACTTACATGAACTCACAGTATCTAAAAGGCATTACATCATCACATGAAGAAATGGAGATGCAAAGGACGTAGATTGCCTATCCCAGATATAGCAGCTAACTGACCTTCAGACCAGGATTTGTCAATCAGTAATATTCAGATCCTCACTCAGCGTATATTAAGGTAGGACATTAACAGTTTGCTTTTATAAAGCCAGCTCTCCAAGAGATGCTGAAATGTGAGCCATGAGAATATATAGCATTTTTTATTTTCTCTCAGTACCGTGCGGGGGCAGGGCAGGGCGGAGGGATGGTGTGCGTGAGCGCGCGCGCGCGAGAAATAGAGCGCTTCGTAACCTCCCTAACCTGCAGCGTCCTGTAATTATGGTACCCTTGCTTCAGCTCCAGACGTGATGGGCGTCCCAAGTATCATACCTTTCTCCAAATGAAAGTCTACCCAGTCACACACTTCCAGTAAAACAACCTTAGTTTGCTTTCAGATTTCCCAATTAACTGTTGGAATTTGGTCATGAATCCTCATAGAAATGATGGAGACTGACTAGAAACAAATGGAAGTCAGAAGCTGTCATTTCCTCCTCTAAGCAGGCAACAAGCTTCTCCCTCATTAGCTGAGGGTGAGAAAGAAATGGCTGGCGATTTAGCTCAACAAGGATCAGACCCAGCCGCAACGTTCCTTCACAGCCCGATGCACAGCCGGGGAAGACGCTAAATCGTACCCGTTGAAACAAAGGGTTCAGACAAAGATAAGACACCTGAGCTTGCTCTGCCAATACACTTTCGGATTGGTTGGGATACTGTCCAATAGGAAAGAAGAAGCGGGGTAACCTAGTTACAACATTTTATATTTGTCCAATCAGATAAGAGTACTTTGGAGCCTTCATTTAAATATAAAGATGACAAATAACCCTGCCTTCACCGTTTGCTTG

>Hist1h3i_M_musculus mm6_dna range=chr13:21263435-21264434 NM_178207 NP_835514 -

TCTCAGTACACTAGAAAGTGAATAAAGTGGTGTTGAGATGGAAAAGCCCAAGTATATATATAACAAGCCACCCTTTTGGATTGGACAGTAAACTGCTGACATCATAGTTCCAGACAGGGGATTGGTCCGCGTTTAAACCGAGTAAGGAAAGGAAAAACGTTGCCTACATTTTATATTTAACGTACAAATTTCATCTTTTTACATAACAATTACTCTAGCAACTCCCAGAATTTATCATCTCGAGATAATGTTGGCTTTGGAGGTCAAAAGACCTCTTCGCTATTTATTTAAACAAGAGCACCGTTATTTGGGGGCAGAAATTTAATGGAAAGAAATCTGAAAGGAAAATTAACAAAAAAAAAAAAAAAAAAAAAGTTATTCCTGGAGTCATGAGCACTTCTTACACTTGATTTCGCTCAAAAACTGCTTGCAACACTTTTATGACCGGAAACTTTTAGAAGTTGCAATTGATACTGCCTTTCAGCGGTTGCGATTTTGGCGCGAAAAAAATTCAAAATCAGTGTATCTGCAACTTACTAGGGCTTTGAGATCCAGAAGGCTTTGAGATTCTGTTACTCTTCTACAGCAACACTGTAGGGGACAGGGGCATTCCCAGGTTGGCATTCATTATTTACAAGTTACAAAGTTACACCATTCAAGGTTAAAAGCGAACGCTGGGAATTCACAGATAGCAGCCCCACCCCACCGCCCAACACACACACACTATGTATACAGCTCTAGGAGACTAAGGCAGCCTTTGGAGTAACTCAGCAAGGGGTGATCTGATGATCCTGCTGAGCCAGGAAGACGATGGCAGGCTTGGTGATATTCAGCACATGACTTTTCGGGAGTGCTTGGTGCCGTTTTTTTTACCCAGGCCCTTTCCCCCACTTAATGTGGTCAAATTTGCCAGAAACAAAAGCAATAAGATCAGACAGTAAGCATGTTGTTTTATGAACAGGCTTTTTGCCTAATTGGGAATATCAGGCACAAGCAATAT

>Hist1h3a_M_musculus mm6_dna range=chr13:23242264-23243263 NM_013550 NP_038578 -

TGGAGATGGTGAGTTCAGAACTACTATTTATAGTATGCAAAGGATAGTGATTGGATGAAAATACTGGTTATCTGTGACGTTTAAGACTCTGATTGGTTTATGTTTAAGAACTGCAGTTGAGGCTAAGGTAGTCTGCCGCGGTACTTATTTTTGTATGTGTGTGTTTTAAAGTAAATGGAATAAAATTCCAGTGACTGTTATCTTCTACAGCACATCTGCTTCCAAACACATTGCAGCTTGGACTTAGTGGCATTGAAGAATAGAGAACAATCTTGTTATCTTCATTATTGAAATGTAACAATGTGACTTCTACATTGAAGGAGAGTAGGCCAGATTCTACCTTTTGATTGGTTTACTGCCTTTCGTAAATAGCCACTAAAACAGCAGATTTAGAACTCTTCATTTGCGTGGAAAGTCTCCATTTTGTTGCAACCCACACTGTAAAGAAATGGAGAGAAACATGGGAGTAAAAGTACAGACGTCTGACTCGGGTTATTCTGAGCATTTCTTCATTAAATGGCTGTACTTGTATGGATGCTTTGAGTTCTTGCATTTGGAGCTTGAATTTTCAAAATACTTTCACGCATTTTTGGCCAGATATCACTGATACGTTCGAAAACAATTTTTTAACTATAAACTATAGAAATCGTCATACCATGTTAGGATTGCACTTTTAAATTCTTTAAAAAAAAAAATGGGCAAGACTTTCCCAGAGCTATTGTGTACTTGCATATCCTGGGAAAAGTCGTGTCCTGCCTTTTCTCTTTTTATTTGAGCCACTACTGGCTCACAATATGAAATCACTGTCGCAAGCTGACACTCCTTAAAAGAACACTAAGCCTCTTGCATAACTAAAATATAAAGTAACGATACATTGCTCCCATTACTGCCAAGAATCAATACTGAAACCCGCAGAGAAGGCCCACAAGCTGGCTTTCTGGGTTTTAATTGACTAATTTACAGCCACCTTTCACTAGTTGAGCTCCTTTTTGATAGGCAT

>Hist1h3g_M_musculus mm6_dna range=chr13:23014484-23015483 NM_145073 NP_659539 +

TGTGGAAAAACCTATATTAATGTTTCAAGCACAAAACCATAATTCTGGTAGGCTGAGGTAATTCTTAGGCAACTGCTTTGTCTTCCTCACGCAGAGAAATATTGGATGTAGCATAAACATAGATTTACACGTTGCACAAAACTTTTGGTCTTGCAGTCCTCTACATCTTCGTATTGCTCCGGAGAGGCAGCAGACATTCGGGGTTGGGAGAAAATACAAAGGGTCTTACGGAAATTGTTAGGCAAGTCTTTACAAAGTCGCAAGATTCAGGATACTTGACCTGTGGTTCCTGTTACATTACTGCTTGCTTTGTGTCACTTTAAACACTCCTGGAGCAAAGACGTAGTTCGGCTTGAGTCAAGGAAAAAGGACCTAGGTGCATATTTTACATATAGCAAACCTGGTCTCTTTTTCTTCCTCCTTGGTTGCCACCTCTTGATTTGACCTGTCTCAGACCTTGGGGCCAGGGAACTCACCCAAGAGCAGTTTCCCAATAAACCTCCCTTTGATATATTCAAATCTGGTTTGGATTGGCTCATTTCACCGGCTGAGAAATAACGTATCATGTGCAGTCTAGGCACAAGAAGCGGGAAAATAAAACCAAAGGCGGGTAGAGAACATATTTTCCAGAGTGGTTTGTGGATATCTAGGGTCATGAGATTTCAAATTCAAGATTTCTTTGCCTCAAATTCTTTAAGCGACTCGATACAAGGATGGAAACATAAATTTAAGAAAGCTCGGGTGTGCCAGACTAAAACATGAGTGTAGCAGTCTGTACTAGCAGGAGACTGTGTGCAGGAGTTAACCAATCGGGTGTGCAGGAGTTAACCAATCACCACTTGAATTCCTCAGCCAATAGTACTACTGCGCGGGACACTTGAAAAGCAGACACGCCTATCAGGATGCTTCTCGGTGGGAAGGAGGGGTACGAGCGCGGGTACGTGTGTTGCGCGTGTGCGAACGCAAGCGTACTTAAAGGCCAAAGTGCGCTACTTAGGTA

>Hist2h3c2_M_musculus mm6_dna range=chr3:95726960-95727560 NM_054045 NP_473386 -

CGCCACCTGACCCGCCCCCACCCGCCCCCCACGTCCAGCCCCGCGCCCGGTCCACTTGCCAACGTTCTGCGAGGGTGGAGCCACGTCTGGCAGCTGCCCGTAGAGGGCGGGTCCCGGCTCTGCCCAAGAGGAGCTCGCGCGCCCCCGCCCGCCGCCCAAAGTCCCAGGGCAGAACTAATCTAGTGTTTGTGCAGCACCGCCCAGCCACAGCCACAGTCCCAGCCCCAGTCCCCCATGATCCCTAGAGCCGGCCGCGTCCAGTCCTCGCGGGACACCTCCTCCTCCTAGACATTCCCCAAGGACCGGACGAGAGCGCGGCTGACTGCGACTTATGTCTAGATTGGCCTAGACGTCCTAGTTGAGTCTTAGTCCTGGGAAGGGTCAGGCGGGTGGACGCTGAGTGAGGGACAGTGTTGTGTAGGACAAGGGTGTGGGCAGATAGGGGCTAAATGCTTGGGACGGTCAGCGAGAACCGCGTATCCGCTCCGCCGAGGCAGGGCATGAACCAAAGAGCGACAACAGACTAACTACCAGGGGGAACCACGGGCAGGATGGGCAAGGAAGCAGAGGACACGACACTCTTACTTTTGGGGCAGGGCCA

>Hist1h3e_M_musculus mm6_dna range=chr13:23042434-23043433 NM_178205 NP_835512 -

TTGCAGAGGAGGAGAAACGGGAGCAGAGGGCTCGGGCTGCTGCTATTTATGCTGGAAATGCGTCGCTCTGATTGGATTCTGGCCAGCAGCACTCAGGCGTCATTCGGAATTTGCGGAAGTGCATCTGGGGATTGACGATTGGCCGATTCCTGTAGTACGATTTTTGATTGGTAAATAACCAATGGTAATTCCGTCCCCTTAAATAATCAGGTTCTAACTGCTGTTTGGTAAGCCCGGAGACATTTCACAGACGAACGTTACTCGTGTCATCTTCCGGTTGTGAACCTGACTTACTATACACAAAGCATGTTCCCAGAACAAAGTGCTCTAAAATTCTAGTTTAACCATTTTCCTGCCTCTGCCATTATCTATATATGTATGTATATGTATACATATATATATATACACACACACTCATGCACATATTTACACACACACACACACACACACACACACACACACACACACACACACACACACACACATGGAAATTGCTAATGCAGTTTATCTGGCGGCCACTACCTGCCAGGAGCTTTGCTAATTTCTACCTCCAGTTTCCCGGAACCATGCTCCACCTACGAAGCCTTTCCTTGTTTATTATTTTCCTCGGGTTAGTTAGTGTCACGTAAAGGACAAGTGTAGTTTTTAATGTGTTTAAAATGTAAGTTAAATTTCTTTCCAGAGACCAGCACGATCTCTTTGTTTTGTGTTGGAGCAGAGCGAGAAACTACCCGAAAACTCAAGAAAGATGGCGTCGCGGTGTTTCTGAAGATCCTTTGTGTTGACTTGCGGTTCATCTGTGGAGACCTGACGGTCCGAGATAAATAATACTTTGTTCTTGCCCACATGCTAGAACCTTTCTTATTTTCAGGCACAGATGAGTCAGCTTATTTTCAAATTACAGTATGGTCGGTTAATAGCAAGTATAAAACGAATCCACAGGGTACAAAAGTCACCTAGTGGTGGTCAAGCAATCTACACTATCCCTAGTCATTTCTTT

>Hist2h3b_M_musculus mm6_dna range=chr3:95755528-95756527 NM_178215 NP_835587 +

CACTCAGGAGGCAGAAACAGGCCAGCCTGGTCTACAGATGGGAGTTTCAGGAACCCAAGGCTACATACAGAAATCCTGTGGTGGCCCACGCCTTTAATCCCAGCACTTGGGAAGCAGAGGCAGGTGGATTTCTGAGTTCGAGGCCGGCCTGGTCTACAGAGTGAGGTCCAAGACAGTCAGGGCTACACAGAGAAACCCTGTCTCGAAAAGAAAAGGAAAAAGAATCAAATAAATAAGTGCTCATTTGCTTTGAAAGTTATTTTGACTTGTCATCTAGATGACAGGTTTAACTATTTAGTAGCTGTTCTCCACTGGAAAGGCGGTATTAATACTTCTAGTCTTATCCTTCTGTTATATAAACATTTCATCTGCAGGACTTAATTGGCCGTTAGTGTAGAAGGGAGGTGGAAGGAACGCACTCTAGACAGAAGGAAAGGCGGGAGCGCCATACATAACGATAGTCAGTGTGCAGTAAATGTAAGACCGGCTTTGGTAGGGTTTCGAAATAGGTAAAACTTAAGTTTGCATTTGGTTCCTTTCACTATACATCGTTTTGTCATCTTTCGGCCGTGGTACAATGCGTTCAAATCACTGGGCACAAAGCCTGAAGTTAAATAAATGCTAACTGTCAGAGAAGAGCTACAGTATCTGGTGACTCGCTACAAAGTGAATCGCAGAACATGAAAAGGGCAAGTTTGTTTCCAAAGAAACCTGTTAGAAGAGCACACTGCGTGAGAAACACTTTAAACACTGCATTTAGGAAAGTTACATCAACTGAAAAGCACTATCTTTTTAAAACCCTCTGCGGTTCATAAATACAGGAAGGAAAAGTATCAGGAGAGAAGGAATAGTGTAACCATCTTGAAACTCAGCAAATAGGATCGAATTCTACCCAATCAGACTGCAAGTCCAGAAACTCCTGCTTTCTGGGCCCAATCAAGACGAAGATAAGTCTATAAGTATGGCCACTTGGCGTCTGCAAGCCGAAGTCGCAAAAATA

>Hist2h3c1.1_M_musculus mm6_dna range=chr3:95726909-95727600 NM_178216 NP_835734 -

TTGGCCGTGGCGTCACCGGCCCCGACCTCGCCCATTGGCTCGGGCCCGCCACGCCACCTGACCCGCCCCCACCCGCCCCCCACGTCCAGCCCCGCGCCCGGTCCACTTGCCAACGTTCTGCGAGGGTGGAGCCACGTCTGGCAGCTGCCCGTAGAGGGCGGGTCCCGGCTCTGCCCAAGAGGAGCTCGCGCGCCCCCGCCCGCCGCCCAAAGTCCCAGGGCAGAACTAATCTAGTGTTTGTGCAGCACCGCCCAGCCACAGCCACAGTCCCAGCCCCAGTCCCCCATGATCCCTAGAGCCGGCCGCGTCCAGTCCTCGCGGGACACCTCCTCCTCCTAGACATTCCCCAAGGACCGGACGAGAGCGCGGCTGACTGCGACTTATGTCTAGATTGGCCTAGACGTCCTAGTTGAGTCTTAGTCCTGGGAAGGGTCAGGCGGGTGGACGCTGAGTGAGGGACAGTGTTGTGTAGGACAAGGGTGTGGGCAGATAGGGGCTAAATGCTTGGGACGGTCAGCGAGAACCGCGTATCCGCTCCGCCGAGGCAGGGCATGAACCAAAGAGCGACAACAGACTAACTACCAGGGGGAACCACGGGCAGGATGGGCAAGGAAGCAGAGGACACGACACTCTTACTTTTGGGGCAGGGCCAGGAAAGGCCACCAAGACCGGCTACCGTGACACAACTCTTT

>Hist2h3c1.2_M_musculus mm6_dna range=chr3:95733900-95734584 NM_178216 NP_835734 +

GAGTTGTGTCACGGTAGCCGGTCTTGGTGGCCTTTCCTGGCCCTGCCCCAAAAGTAAGAGTGTCGTGTCCTCTGCTTCCTTGCCCATCCTGCCCGTGGTTCCCCCTGGTAGTTTGTTGTCGCTCTTTGGTTCATGCCCTGCCTCGGCGGAGCGGATACGCGGTTCTCGCTGACCGTCCCAAGCATTTAGCCCCTATCTGCCCACACCCTTGTCCTACACAACACTGTCCCTCACTCAGCGTCCACCCGCCTGACCCTTCCCAGGACTAAGACTCAACTAGGACGTCTAGGCCAATCTAGACATAAGTCGCAGTCAGCCGCGCTCTCGTCCGGTCCTTGGGGAATGTCTAGGAGGAGGAGGTGTCCCGCGAGGACTGGACGCGGCCGGCTCTAGGGATCATGGGGGACTGGGGCTGGGACTGTGGCTGTGGCTGGGCGGTGCTGCACAAACACTAGATTAGTTCTGCCCTGGGACTTTGGGCGGCGGGCGGGGGCGCGCGAGCTCCTCTTGGGCAGAGCCGGGACCCGCCCTCTACGGGCAGCTGCCAGACGTGGCTCCACCCTCGCAGAACGTTGGCAAGTGGACCGGGCGCGGGGCTGGACGTGGGGGGCGGGGGGGGGCGGGTCAGGTGGCGTGGCGGGCCCGAGCCAATGGGCGAGGTCGGGGCCGGTGACGCCACGGCCAA

>Hist1h3b_M_musculus mm6_dna range=chr13:23231470-23232258 NM_178203 NP_835510 +

ACATCTTTCCATAAAATGAGCTGCCACCTCGTGAAACGTTCTTCCACTACAGTTTTTATACTACATATGAAAAAGTTACGAAGTAGCTTTCAATCTTAGTAAATTGATTTTAATACTGTTAGTCCCTGCGATAAATCTTACGACCTTCCTTAGTTTGAGTCAAAAGTGTGTAAGAGATGAAACCTTTAGAACATACTATATAAATTTTTAGTAGAAATTTGGCACCCAGGTTTGTCATTCACGTCACGATTGTCTAGAGCATAATGGTAGTAAGGGCTAAGGGCCATTAAATCCCACTTCCATAGTTTCGGCAGTATTTTAACTAACATGATCTGCCTGTGTGTATCGGTTTGCAAATATTTGATTTTAGAATTGATAAACTACATTTATTTAAACTAGGTCTTGCAAAGCTCACAAAACCTACTTTAAATTTCTATGTTCACTTGTTTGATTACCTGAATTGAGTTTTCAAAGCTTGAGACAATTTGATGTATGTATGTAAGTAAAATTATGTAAAACTTTCTTATGTAAAATTTCTGTTCACAGGAGTTATATACACTACAGTGTAAAATTAAAGTGATAGCAAAGATGGAGGGGAAGAGAGGCGGAGAGTGTGCATGTTCTGAAAGCTTTTTAACCAGGATTTAGAAGCAGAGGCTGACCAATCCCAACAAAGCGCGGGCCCTTTGAATGTTCTTCGGTCCAATAGCGGATAGTCTGATTGTATAAAAGGTGGACAGCGCCTTGCAGCTCACTATAGTGTCAGTCTATTTTCCCTTGTTAAAGTCA

>H3f3b_M_musculus mm6_dna range=chr11:115845597-115846596 NM_008211 NP_032237 -

CCAAGCCCCCCGACGCCTCCATATTTATAGAGACGCTGCACGCTGCGTCAGTGCGTCACAGAACCCTGATTTGCATACACCAACGACTTTTTCGATTGGCGGGAGCTTTCGCCGACGCGCTGACATCTCCCGACACAAAGGCCGTACTTCGGGGCCGTTTCCTGATTGGCCGGTGTTCAGGCCACTGATTGGCTGTTACAATTGCTTCAGGATTGGCTGGATGCAAAGAGGAACAGACGAATCAGAGTTTAGCACTAGGGAGCTCTTCTGATTGGATAAAATGGAGCTATGTTTGGATCCTTCTCTTGGTTCCAAAGCTCTACAATAGAAAGTCAATGCAAATGAATTGCACTAGTCTCCTGGAATTTAAGAAAGTTGGGTCGGCAAGAGTCTACGACTTTTTTGCATCAAGAGACCATTTTAGAGAGATTTTGCGTAAATATAATAATAGTAATTATTACTATTATTTTAATGTAGGATGAAATTATTCCCTGAATAATTTACTTTCTGTAATTTTCATCGTTTAATATAGTATGAGAAATGTAATCCAGTCAGTCATCTTACAGCATATAGTGTTTTGTGCACTGTAAATGCATACACATTCTAAAGCATAAAGCAATTGTTTGATGGGGAAGGGTAATAGACACAAAGAATCAAAATTCAAAAAAATTATTTCCGAGTTGGGTGGTGCTGCAGGCCTTTAATCTCAGCTTTTAGGAAGCTCAAAGGCAAGCAGATTTCTGTGAACATGAGGCCAGCCTGATCCACAGGGGGTGGGGGGTTCCAGGCCAAGCAGACCTACCTACAGAGATCTTGTCTTTTATAAATCCTGGTTTGTTTGTTTTTTCTCCAGGACTACATAAACCAAGCTCCAGGTGCATGAAGGTAATATCAGAGATGGAATCAGGATCCAAAATTCAAGGTCATTCACAGCTACTTAAGTCAAGGCCAGTCTAGGATATATAAAACAGTTTCAAGAAAACAAAAAACAACAAAAGGG

>H3f3a_M_musculus mm6_dna range=chr1:180767424-180768423 NM_008210 NP_032236 -

CCCGCCCCGCGCCGCGCTCCAGCCAATCACACACAATGGAGGGAAGGCTCGGCCCCGCCCAGGGCCCCCGCGCGCCCCGCCCCTCCCCTCCCCCCGCGCGCTCGTTCGCTGGCGCGCGCTCGCTCCTCCCCCGCGCGCCGCCCGCCCATTCCTCCCGCCGCCTCTCGCGGTGTGTACAAACACAAAAGACACGCCGAGGGGCCCGCCGAGGGGCCGTCTGCCCTAGTCCGTCCCCGGCCCACACTCCCTCCCCGTCGGACGCAGGCGTTAAGTGAGCCCCCGAAGCCCAGCTTTGGGGTGCACAGCGGGGGGTGTGGGGGCGGGGAGGGCGGCGCGGGCTGCGGACCAGCGCCCGGCCTGCGGGGGGAGGGGCATCGCCCCCGCGCGCGCCCCCGAGGTTCCCGGCCCGTGTGGGGGCGGGGAGAAGGCCGGGACCGTACGGTCGCCGCGCCCCAGTTGAAAACGCGCCCAAGCGGGAAGCTAGCTGGCAAAGTGGAAGCCCCAGTCCCGCGCCCACTTTCGGACCGCGCGGCGCGCCAGGGGCGGGGGACCCGCGGGCCTCTTCCCGCCTTCGCTCCCCGCCGAGCGCGGGCCGGTCCGGAGGCTCGGGCCAAGTCGCCTGAGGACAAGTCGGTCGCAGACTCTAGCCCCGGGAGAGGAGGGGCAGCCTGCGGGGCAGTGCGGAGACTGTCAGCGCGTCGCAGGTGGTGGCACCGGGGCCGAGGGACAGGGCTCGCCCTCTTCCTAGGCCGCGGTTCTTGGTGCAAGATGGCTGTTATTTTGTAAGCCTCTGAGACAATGTGCAACACAGCTGTAGCAGTCTAAGGCAGAGTAGGCCTTTCCACCCCTTCGCTGTTTGGAGGAGCGCTGCAGAAATCTCGTGACATAAGAGTCTAATGTCAGACACAGGAGGCTGAAACGGCAGGTAGAAGGAAAAGCTGTTTCCCGCTTTCCTTCTGCCCAGTAGCTTGGGCAACAGACTGCATAGCTCAAGTTCTCA

>AT3G20670_H2A_A_thaliana ref|NC_003074.4|chr3:1-23470805:7228479-7229478 Arabidopsis thaliana chromosome 3, complete sequence

CGGACATCGTTACCGTTTCTATCCTCCGGAGATTCCATAATTTATGTTTTGATTTTTACAGTGTTTTTGTTTTTGTGTTTGTGTTTTTGTTTAAAAGGCGTTCTTGATTTGGTTTGACGCGAAGAAGGAGGTGGCTTCTTTGTCGTATTTATATCTCTTGTTTATCGGAAAACAATTTTTCCTTCTTTTTTTCTCTAGCGTTTTTTATTCTCTTTTGATTTAGTAACTATTTTTTTTTGTTGACGATATTGACTACATGCTCTGCTTATATATATTTTAACTATGTCATGTTATTAACGTTTATGAGTTGTCACTTTGAAAATTAGTAAGACACATCATGTATTATTTCTCAAATTAGTATTGTACATTTCATTTCAAAGTCAAAAACACATGATTCTATATATGGTTGATTACCTAATGTGTAATCGTGAATTAGTAATTAATACGACATGCTAAAAATTGATTAATCATGTTTAGAAAAATATATACTATGATAAACCTGAAATTGTGTCACACAATTTTGATGAATGTATATACCACATTTCCATATTATACGTTTTAAAAGTAAGATTTTCATAAATTTTAAAATTATTCATAACATTCACTAAAATTAGATGTGTATAATTAACAAACTAAAAATATCATTAATCTACTATTTTAGTAGTTATTTTGCGAAAATATGTTTGAGTTACAAAATATTTTCACTATTTAAATCATGTCGATTATACCCACTGAAGGGTATTTCCGTCAATCCCAATTCTAACAATGAATTCAGGAGTATAAAAACGTAAATTCAAGCGTGCCAATTATAAACCGTCGATCATAATCTAATCCAACGGCAGTAACATCGATCCGCGTGATTGTTTATTATTGGATAAGAATCACTCAACCGTCTCTACACAGTATATATAATAACCAAAGAGCGTCCTCTTACGCTTATCTTAATTTCCCTCGCATTGAGAATTTTCAACTTTTTCTATCTCTCTTCCCAAATCACAAA

>AT3G54560_H2A_A_thaliana ref|NC_003074.4|chr3:1-23470805:20207146-20208145 Arabidopsis thaliana chromosome 3, complete sequence

TTTAACCGCCAAAGCGTAGGTTTCCCCCCAAGATTTTGAAAATATTTAAAAACTCCCACCAAACTTTTTAATTTTAAAACTCTAATCCCATTCTATTCAACCAGATTTCGTTTCTTTCGTCCTTTTTTTCCTTTTGCATTCTCTCGTCGTCGTCTCAAGGTACTTTACTTCTCTTTTTCTCTCTTCCAATATTCGAGATCTGTTTCTGTCTTTCTTGGATCGATTCTCGATTCTGTTCTTCGATTTAGTCTTCTTTCGAATAGATCTGGTAGATTTAAGCATTATACTCTTCTTTTTCTGATTTCGTTTTTGTTTGACTGTGTACGGTTAGATCTAGAAGAAGGAAACAACAATTTCAAGAGACATGGCAGGCAAAGGTGGAAAAGGACTCGTAGCTGCGAAGACGATGGCTGCTAACAAGGACAAAGACAAGGACAAGAAGAAACCCATCTCTCGCTCTGCTCGTGCTGGTATTCAGGTCATCTCTTAAACCCTAATTTCGACGACCTTGTTTGACTCTGATTCTTTCCTAATTCATCAGTACCATTTACATTTTTAGGAATAGATTTGTTTTTTTGGTTCTATGTAAAAGCATGAGGAAGTAAACTTGCTGGATATGTGTAATTTCTTTTACTCGGTACCATGTTGATGTTTTTGTCAATGTTTGTGCTAATTATACAAATTTGTGTTGCTTGCTCACTGGTTGCTTGGTCATCTGAGAATACATGTTGTTGTTGTTTTTGTCTCCCCATTGTTTAGGTAGTGTCTTATGGTATGTGCCCAAATGTTCCCTTACTCTGTAGCTTACTATTGATATTGATGAGTCATGAGGGTTTTAATATGTTTTGTTTGGTTCTAGTATGTGCAATGTTCTGTTTTTTATTAAGTTATACTATTTTAATGGAACTATTTGGTGTGCGCTGATACTGTTTTGACATTGATGCTGTGCATAGCCATACAAGTAGAGAGATTGGTCACACCGATACTGTTTTTTTTTTTC

>AT1G52740_H2A_A_thaliana ref|NC_003070.5|chr1:1-30432563:19648588-19649587 Arabidopsis thaliana chromosome 1, complete sequence

TTGAAGGCTTTCACATCAACAAAGAAGGAAAAACCGCAGAGAAACCATCTGATACTTAAGCTAAACTGAGCGTACAAAAAGCCTCTATATGTCTTAGTTCATGATTTGCTATGTTTTGTTTCCAGACTGAATGATTATACAGAGAAAACAAACAAAGATCTCCCTCTCTTCTTTTGAATCAAAACATGGGTGTTAAAATTTAATAGTTTTCTTTCAAGTGTCTTTTTCAATATTGAACTAAATTTAGGGACGAATTTGTGATTTATGATTATTTGACTTTAGATTGGGCTTGGGCTTTTTTCGCAGGTTGGGGTATAAGGGTAAAATCGTCATTTGACAGACCGACTTGTCTCTCTCTATCTGGGGAAAACGTCTATCGGGAGACTCCTCTTCGAGCTCATCTTCTTCTCTCTCTTTTTATCTTTGGTTGTGCGATCTCCTTTCTCTTTCAATCTCCAAGGATTTTACTGTGAGATATTTGGCGGGAAAATGTCGGGGAAAGGTGCTAAAGGTTTGATTATGGGGAAACCCAGCGGTAGCGACAAGGATAAGGACAAGAAGAAGCCTATCACTCGTTCTTCTCGAGCTGGTCTCCAGGTAGATTATAATCTCCCTCACACTCTAAGTCTTCCGTGTCTGTTTCTTTGGGAATCGAAATGGTCTTATACACCTGAACGATTAGTAGATCGCGTTTAAGTGGTAGATCGATGAGATTCTGAGCTAGATTTGGTAATTTCAGCTGAGAATTAGAGACATTGGGATGCGAGATTTGGTTTTCTATTGTGTTATCTGCTGGAGAATTGTTTCATTAAGCTTTTATGGTTGATATTGAACCCGATCTTTGATTTCACGGAGTCTTGTTGTTACAGCTACCTTGTGAATTGAATTCGGAGTTTTTTTTGTAGAGATTTATTGTCATATATGAAATGTTTCTGGGAGCAATTGAGATTTGAGTATTCATTTAGGTTCCATTGTTGTGGCTAATTGAATTTACATTGTG

>AT1G51060_H2A_A_thaliana ref|NC_003070.5|chr1:1-30432563:18929885-18930884 Arabidopsis thaliana chromosome 1, complete sequence

GAAAAACCAGAGAGACACAATGCACACAACTCTCAGATATAAAAGATTGAAACTTTTAAGTTTGATTCAGAAAGCAAATTACAAACCACAAAGATTCATATCATATAATGTCAATCACTTACTGGAAACTTCATATAAAATCAAAATTGAAAAAAAAAAACTGAATTCGAATTATGAAAATCAAAAAGGAATGAAGCGGGAACAAAACCTTGGGGATTTAGTTTGAATCGTGATGAAGAAGGAAGATCAGAGCTTGAGGGAGATTCGAAATTTCCTCGCTTCATAACAAAATCTGAGAAATAGATTTGAAAAACAGACAACACTAGGTTACAAAAACTGTTACTCGATGAATAAAAAAAGAGGACTTTTTCAAATCTTCACACACAAATTTCACAAAGAACCCGGATTCAATTTTTGAAAATTGGGCTCTTTGGTAAAATGTAAAACGTTTGGGCCGAAAAAAGAAGAAAAAAACAAAACTGTAAAGAGGCAAAGAGGATATTTTGGTAATTCACTCTGACGCGGATCCTGAATCTCGAATTATTCACCGTTGATTATAACATTATCTAACGGTGATAAACAGCGATCCGCGTAGTTTCTTCTTATTGGTTAAGACGAATCTAAAACAGTATATAAACTCTGGAGAAGATGGAGAGAGTCCATAACAACAAATTCGATTCTTATAACTGTTTCCCTCTCATCTTTACACAAAAGTATTCTAATCGATTTCAATGGCGGGTCGTGGTAAAACACTCGGATCTGGGTCTGCGAAGAAGGCAACAACAAGAAGCAGCAAAGCCGGTCTCCAATTCCCTGTGGGTCGTATCGCTCGTTTCTTGAAGAAAGGCAAATACGCCGAACGTGTTGGTGCCGGAGCTCCGGTTTACTTAGCCGCCGTTCTCGAATACCTCGCCGCTGAGGTAATTCCTCTTCCCTATTCTTCAAATTTTCGATCTTTTAGTTCAATTTCTATAAACCCTAATTTTGACTGATTTTGGGG

>AT1G54690_H2A_A_thaliana ref|NC_003070.5|chr1:1-30432563:20418495-20419494 Arabidopsis thaliana chromosome 1, complete sequence

CACTCTTTTTTTTTTTTGACATAAGTGTTTTGACTTTTTAAGTTTGACTTTATAAAAACATGCAGAAATGTACAAAGAATATATACATATAATTATCTTAATTAATTTAATAACTATCAATCTGTCATACTACACCACTATCAATCTATCATCATCACCACCATTATGCTTGACAGTCACTTTTTAGTTGGCCCATGTTAAAGCTGTTTGTGTTATTTGTTATTGGGCTTATCCTTCACTACCATTTGATTGAAATTTATCTCATGACCCAACAAATTGAGCTAATTTCGGTTCAACATTGGATGTTAATTTTTTTTCAAACCGAACCGAATTATAGTTTTGGTGCATTTTTTCTAAACCGAATTTTAACACAAATAGTAATCGTCTTAAAAAATTCACCGACTTGTTAAAAAGAGGCGGAAAAAAAAACCCGCGAGAACTTACAATGGTGCCACGCTGGCAATCCGCGTGACTCACAACTAACCAATCAAAATCCATTATCTCAACGCTATATATTTCAGAAATCACAACCTAAACCCTAAATCACTCCACTCACAAAATCCTCAGCCATCTCTAATCACATTTTACAATCGCCTCTTCAAATTTCCCGATAAACAAAAAATGAGTTCCGGCGCCGGCAGTGGAACAACTAAAGGTGGCAGAGGAAAGCCAAAAGCTACAAAGTCCGTCTCTCGATCTTCTAAAGCTGGTCTTCAATTTCCCGTTGGAAGAATCGCTAGATTCCTTAAAGCCGGTAAATACGCCGAACGTGTTGGTGCCGGTGCTCCCGTTTATCTCTCCGCCGTTCTCGAATACCTCGCCGCTGAGGTAACAAACAATCTTCTGTTTGGTATTTAGTCTTTTAGTCTCTATGATGAGAATCACTCGTAATTGATATATCACTAGATTTTTCGATGTTTACCGAATCTTTGATTTTGATTTGATGTTAAGGTGTCTTCTAGAGTCTGATCTCTTATATGATGTTGATATAATCATTAGG

>AT1G08880_H2A_A_thaliana ref|NC_003070.5|chr1:1-30432563:2847368-2848367 Arabidopsis thaliana chromosome 1, complete sequence

TTCCTTCTCCTAATTGCTTTTCCCTGTATTTCTCTGTTCTTTAATAGTCTTCACTCTTGAAGCTTGTATTTGGCAGTGACTCATTGGTCGTGTCCCATGTGAACAACTGTTATAGATATTTGTTGACTATATATAGTAAGATAATAGAAATCTCTTTATGTCGGCTATCGTATAAATTATTGATTTATTCTTGTTGCTTGATCAATAAAGGTTACATCATAGAACTAAAATCATATGAAACCGAATCGATCAACCCTGGCCATCTTTTAAATAACCATCAATACATTGGGATGATCAATCCACAATAAATGTATTGATGTAAATTAAAAATATGAACTTGTAACAGATCAAGATTCAGGGTCTAAAATTATAGAAAGCTTAATAATGGAGGACTATTTCACTAAAATCACTTTTCGTTTGTACATTATTTTCAAAAAGTAAAAGGAGATGTACGAACCATAGATCACATAATAATTGAAAGGGTAGATGATCTGCCACGTTGGCAATCCGTGTGATCTAAAGTCTAACAAATCACAATCAATCTTAGTAGCCTATACATATAGAGAAGAGCAAAACCCTAAAGCCCACTCATCTTCTCAATTCCCAGATCATCTACAATAGTCATTTCTCTTCGATTTCTTCAAACTCTCATCAAATCGTTTATCTGTTCTAAATTTCGAAGAAGACGATGAGTACAGGCGCAGGAAGCGGAACAACCAAAGGTGGCAGAGGAAAGCCAAAGGCCACCAAATCCGTCTCTCGATCATCTAAAGCCGGTCTTCAATTCCCCGTCGGAAGAATCGCTAGATTCCTCAAATCCGGTAAATACGCCGAGCGTGTCGGTGCCGGAGCTCCGGTCTATCTCTCCGCTGTTCTCGAGTACCTCGCCGCCGAGGTAATTTATTTTTCTTGTCTTCCAATTTGGTTTTCAATTTCGATTTGGTCACATCTGAATTGGATCTTGTACTGATTTGATTTTGATTTGGTTTGGGTTGATAGG

>AT1G51060_H2A_A_thaliana ref|NC_003070.5|chr1:1-30432563:18929617-18930616 Arabidopsis thaliana chromosome 1, complete sequence

AACACACTTTTAATCTGAATATAAAAACTAATCAAAGATATGAATACATAACTCCTTTTGCTATTACAAATTTCCATTAGAAGAGATAAAGATCAGTCATTAACTAAGTAAGTAACTAACCCAATTGTTAAGTTTTTTTAAATGAATGATGGGACTTGATTCTCTTTCTTCTAAAGTTTGATTTGTTCTTCTACTATAATGATCAGCGACCTTACGAGCAAAATTCTTTGTGTTATCATCCTCTTTTAAAATCTCAGAATCACCTGATGAAAAACCAGAGAGACACAATGCACACAACTCTCAGATATAAAAGATTGAAACTTTTAAGTTTGATTCAGAAAGCAAATTACAAACCACAAAGATTCATATCATATAATGTCAATCACTTACTGGAAACTTCATATAAAATCAAAATTGAAAAAAAAAAACTGAATTCGAATTATGAAAATCAAAAAGGAATGAAGCGGGAACAAAACCTTGGGGATTTAGTTTGAATCGTGATGAAGAAGGAAGATCAGAGCTTGAGGGAGATTCGAAATTTCCTCGCTTCATAACAAAATCTGAGAAATAGATTTGAAAAACAGACAACACTAGGTTACAAAAACTGTTACTCGATGAATAAAAAAAGAGGACTTTTTCAAATCTTCACACACAAATTTCACAAAGAACCCGGATTCAATTTTTGAAAATTGGGCTCTTTGGTAAAATGTAAAACGTTTGGGCCGAAAAAAGAAGAAAAAAACAAAACTGTAAAGAGGCAAAGAGGATATTTTGGTAATTCACTCTGACGCGGATCCTGAATCTCGAATTATTCACCGTTGATTATAACATTATCTAACGGTGATAAACAGCGATCCGCGTAGTTTCTTCTTATTGGTTAAGACGAATCTAAAACAGTATATAAACTCTGGAGAAGATGGAGAGAGTCCATAACAACAAATTCGATTCTTATAACTGTTTCCCTCTCATCTTTACACAAAAGTATTCTAATCGATTTCAA

>AT1G08880_H2A_A_thaliana ref|NC_003070.5|chr1:1-30432563:2847649-2848648 Arabidopsis thaliana chromosome 1, complete sequence

GCTATTGAACAATTACCAAATTCAAAACAGATTTTGATTAAAAAAATAAAAATTCCAATCTGAGGCATGTTAGTTGAGATCCATGAGGAACACTAGAAAACCAAATGAAAGAAAAGGAAAGAGTGATGATGAAATCTCACAGCGCAGCCATAAACGAAAGAGCCAGTGACAGCGACGGAGGTACTAAGAAGAAGACCACAAGTGATGGAAGAAGAGCTTCTTGCTTCTTCTTGATTATTCACCAGAGGTGTTTTCATACTTCCAGATTCCATCTTCTTTTGTTCCTTCTCCTAATTGCTTTTCCCTGTATTTCTCTGTTCTTTAATAGTCTTCACTCTTGAAGCTTGTATTTGGCAGTGACTCATTGGTCGTGTCCCATGTGAACAACTGTTATAGATATTTGTTGACTATATATAGTAAGATAATAGAAATCTCTTTATGTCGGCTATCGTATAAATTATTGATTTATTCTTGTTGCTTGATCAATAAAGGTTACATCATAGAACTAAAATCATATGAAACCGAATCGATCAACCCTGGCCATCTTTTAAATAACCATCAATACATTGGGATGATCAATCCACAATAAATGTATTGATGTAAATTAAAAATATGAACTTGTAACAGATCAAGATTCAGGGTCTAAAATTATAGAAAGCTTAATAATGGAGGACTATTTCACTAAAATCACTTTTCGTTTGTACATTATTTTCAAAAAGTAAAAGGAGATGTACGAACCATAGATCACATAATAATTGAAAGGGTAGATGATCTGCCACGTTGGCAATCCGTGTGATCTAAAGTCTAACAAATCACAATCAATCTTAGTAGCCTATACATATAGAGAAGAGCAAAACCCTAAAGCCCACTCATCTTCTCAATTCCCAGATCATCTACAATAGTCATTTCTCTTCGATTTCTTCAAACTCTCATCAAATCGTTTATCTGTTCTAAATTTCGAAGAAGACGATGAGTACAGGCGCAGGAAGCGGAACAACCA

>AT1G54690_H2A_A_thaliana ref|NC_003070.5|chr1:1-30432563:20418843-20419842 Arabidopsis thaliana chromosome 1, complete sequence

TCGAGAATGACGTCATTCTTCTTCACAAACTGGTTACCAAGTGCATGCTGATTGACCTCAGTCACTACAACACCGGTCATGATCTTCTTCATCTTAAGATACTTTCGCATCATCGCATTCTTCATCGACTGTAGAGAGTAAACAAAACCCTATCTTGTCTCCTTCATCAATACTAGCTAAGAATAGAATAATTTTCTTTGTACTGTTATTATTTTGATGAAGATAAACACCCTTGTATACAAAACATATCGACCTTGCATAAGTCGCAACCATTCCATGTATGCTATAATTATTTGTCAATAAATTCATTAGTTGTAACATGTGGTCAAAGTTTGAGTTTTAGTTGTTCACTCTTTTTTTTTTTTGACATAAGTGTTTTGACTTTTTAAGTTTGACTTTATAAAAACATGCAGAAATGTACAAAGAATATATACATATAATTATCTTAATTAATTTAATAACTATCAATCTGTCATACTACACCACTATCAATCTATCATCATCACCACCATTATGCTTGACAGTCACTTTTTAGTTGGCCCATGTTAAAGCTGTTTGTGTTATTTGTTATTGGGCTTATCCTTCACTACCATTTGATTGAAATTTATCTCATGACCCAACAAATTGAGCTAATTTCGGTTCAACATTGGATGTTAATTTTTTTTCAAACCGAACCGAATTATAGTTTTGGTGCATTTTTTCTAAACCGAATTTTAACACAAATAGTAATCGTCTTAAAAAATTCACCGACTTGTTAAAAAGAGGCGGAAAAAAAAACCCGCGAGAACTTACAATGGTGCCACGCTGGCAATCCGCGTGACTCACAACTAACCAATCAAAATCCATTATCTCAACGCTATATATTTCAGAAATCACAACCTAAACCCTAAATCACTCCACTCACAAAATCCTCAGCCATCTCTAATCACATTTTACAATCGCCTCTTCAAATTTCCCGATAAACAAAAAATGAGTTCCGGCGCCGGCAGTGGAACAACTA

>AT5G59870_H2A_A_thaliana ref|NC_003076.4|chr5:1-26992728:24133337-24134336 Arabidopsis thaliana chromosome 5, complete sequence

CTTAGTTTAAATTGGATGGTTGATTACCATTTACCAATGTTAACAACTTAAAACTTCAATAACGTAACGAGCATGTAAAACTAACCAACCAAAATTTAAGAAAATGTATATATTGATTCCACTTTTCACTTATCAATCTGATTGATTTATTAAGTGTAAATTGCAAAGTTTAATCTCATTTTTTTTTCAAATTTTTTATTTTTCAAGACCAAACCAAAGTAATTAATTAGATCATACCAAAAAGCGCAAAATGGCGCCGTTTCGAGAAAAAACCCACTCCATATACTCTTATATTTTTCCATATAATTCTTTCAGAAAAAATAAAAGTTCGAATTAATTGGGTTTGCATAAGTTTTGTTGACATTGTTGTTTAATGTAATGATTGAGTCAAGTCTATAAACTATTAAACTCTAGGGTTTAATATGTACAAATTCTCTTAGGCTACTTTTGATTAGGACTCCCTTGTGAATGTCAAAACATAATGCGACCCCAAAATATCTTTATAAGTATAATTGTTAATCTTTTGATTCTAAAATATTGTTCATTGTTTTCCAATTAGGGCTTCAAAGACTCTTGAGAAGCATCATTAAACATTTAAATGTCAATGACTAACTTTACATTTAACATATAATTAATCTACCGAAAATTAGTGTAAGTTGCAAGAAATTATCCAAAAACCCAAAATAAAGCAAGCGCTAAACTTTTAAAATGCTACAAAAAAACTGGCGCCGTTTCAAAAAGCATACCTCTTTTTGATTGGTTAATACATAGTCACGCGGATCGTGCTTTATTTGAACATCCACCGTCGATAGACTAAATCCAACGGATAATAATCCTCTCCCTTCTTTTTTTTTCATTTACCTATAAATATCACAGAGTACCCTTCAACTTTAAATCACAAATCTTCAACTTCCGATACTTTCAATCTCTCTAAACTCTCAATTTCAGTAATCGATAACCGTAGCAATGGAATCCACCGGAAAAGTGAAGAAAGCTTTCG

>AT5G54640_H2A_A_thaliana ref|NC_003076.4|chr5:1-26992728:22213300-22214299 Arabidopsis thaliana chromosome 5, complete sequence

ACAATGGTTGTTCAATAAAAATATGAACAACACAATAGAATTAGTAAAAGTGACTATGTTAAATCATTTTCTTCGCTGGGGTTTGGTGGGCGAGTTCTAAACCCATAAGCGGCCCATTTACTTCGTAAACTCAATTCGATTTGTTCAGCGTTCCAAGCCCATAATATTATTTTCAAGGGCATAAAATAAATTGAGGTTTATATGGAAAATTTGGAAATTCCCTCGTCCAGAAGAAACCAACAAAAACTGCAAAAGTTCAAGCGGTGGGAGAAAAAACTTCAGATCGTAGCCATTCATTAAATTATAATCAACGGTTTAAACCTCTTCGATCCGCGTACTCTATTCTTATTGGTCAAATAACTTAATCCTCCAACATATATAAACAACAATCAGATTTCTCTCTGTTAATTTCGTCAAGAAAAAAATTCGATTTTTTTGCGCTCTTTGTGGGTTGTTGTTGTTGAAAATGGCTGGTCGTGGAAAAACTCTTGGATCCGGTGGGGCGAAGAAAGCTACATCTCGGAGTAGCAAAGCCGGTCTTCAATTCCCGGTGGGTCGTATCGCTCGTTTCTTAAAAGCCGGTAAATACGCCGAACGTGTTGGTGCCGGTGCTCCGGTTTATCTCGCCGCCGTTCTCGAATATTTGGCCGCCGAGGTAAAATTACATCGTCTTTTCTCTCTTTCCCATTCCGTTTCCGATCTTATTCGTCTGACTCTGTTTTTGCGTGATCGATTACGAATCTAGGGTTCTTACATTTTCCGAATTTGACATGCAAAAATTGAATTAGATTCGTGTTTGAATTGAATTGTTGTAGTTCTGTAATTGACCTAATTTTGGGTTTGTTCTGATTGGTTGATGGTAATCGAGATCATATGAATCGTTGTAGTTTTCTCGCAAGATTCTAAATTTTTTTCAATTATGGTAACCAATTTGATTTGAGTTGTTAAAGTTCTCAAATTTGGAAAGTTTGATCATGAATTGTGTGTTTTGAATTTGTTC

>AT5G02560_H2A_A_thaliana ref|NC_003076.4|chr5:1-26992728:575180-576179 Arabidopsis thaliana chromosome 5, complete sequence

TTTGGAGTATTGCTATTAATCCTTTTAATGTGAACAAAACATTGAAGCGAAGGTTGCCAGATCAGCAAATCATAGCCGTTGATTCACTTCCAATCCAAAAGCTAACATTCATCAACTGACAAAACCAACCAACCAACCAACTTCTTTCGCTATCTTACGCCAAAGCTCTCTTAATTCCTCCGTTTGCATATTTTCCGGTCAGATCAAAATCAGAATCAGAATCAAATTTCTCGTCGTGTCGGAGTAAATCAAGCCATGGATTCCGGAACCAAAGTGAAGAAAGGAGCCGCTGGAAGAAGAAGTGGTGGAGGTCCTAAGAAGAAACCGGTTTCCCGTTCGGTTAAATCCGGTCTACAGTTTCCTGTCGGTAGGATCGGTCGGTATCTTAAGAAAGGTCGTTATTCGAAGCGTGTCGGAACCGGAGCTCCGGTCTATCTCGCCGCCGTCCTCGAGTATCTTGCTGCTGAGGTAATAAAGTTCTGAATTCAGATCAGCTAATCATTTCATCGGAATTATCGCAGTTTCATCGATTTCACTAGAATTCTTGTGGGTTTTGTTCTGTTGCTTCGTTGACCATCTATAGGTGTAGAATGTCTTCTTCTGATTTTAGGGTAAATTGATAATCATCTGAGGTTGTAAAATTGAATTTGTTAGATACTATATCACGAGTAGATCAACCTCAAGACATGGTTTCACTTTCAATTAGGTTTAACATCTTTGCTTTGCAAATCTCAAAATCTTAGATAGAGATATATTAGCGTTACATAAAAACTAAAGTTGCATAGTCAATAAAACCTAAATAAAACATCTGCAAGTAAACTTCATTGAGAATCTATCATCATGTAACACCGTTTTGAGAATCTGAATACCTTGGACTGATGTGCATGTTACATGTAACTCTTGTCAACAAATCTCTGAGTAACTAGGATATGCAAATATTGCATACTAATCTTTTTGATCGAATGTGACAAAACCCCATTTTAAAGTTTACAAGTCTGAT

>AT5G27670_H2A_A_thaliana ref|NC_003076.4|chr5:1-26992728:9793046-9794045 Arabidopsis thaliana chromosome 5, complete sequence

GCGGAAGAGAACGTAACAAGGCTTCAAGAACGTCATGATGTGATTCTCAAAGAGATATGTTCTTATTATCTCGTTAACACCGAGCTCCATGAAGCTTTGGTTTCATCTCGTGCAGCCATTAATGATGCTTCTTCATTTGTCATGGAGCTTAGAAGATTACAGTTGAATATCTTAAACTCTCTTTCTTAATCACATTTTTTGTACTTATTACACTAATTAAAACCAGAGTTTGGGTAATAATTTTTGTTTCCTTAATTTTCCGAATTATCCGCTAATTTTCTACTCTAATTCTCTGGATATTTTAAATAATAGTAATAATCTGCTGTCAAAATAAGATAAGAAAAAGACATAAAGCTGATTATCTTGTAGAACGTGTGGGGAATGAATCTAACGGCTGATATCACTCAAGTGTTCTTTTCCACCTTCCTTTTACAACACCCACGTGTAATGTCATACAAAGAAGTCATTACGACCGTTAGATCAAAGCCAACAAGATCCAATCTTAACGGCTAAGATAAATTACTACACGGATCGCCAACGTGGCAATACGTGGTATATACATACACGTCGTTCTTTCCTCATTTTAAGCAAATCGTAAACCGCCACAAAACCGAAAAAAACACTAATTGTGCTTTCCCTTTAGATTCATTTGTATTTTCTTTTGGAGCTTTTGAACAATGGAGTCATCACAAGCAACGACGAAGCCAACGAGAGGAGCAGGAGGAAGGAAAGGTGGAGATAGGAAGAAGAGTGTTAGTAAATCTGTTAAAGCTGGTCTTCAATTTCCCGTTGGTCGTATCGCTCGTTACTTGAAGAAAGGTCGGTACGCTCTCCGATACGGTTCCGGTGCTCCGGTTTACCTCGCCGCCGTTCTCGAATACCTAGCCGCCGAGGTATATTCAATCTCAGATCTCGTTGCATTTTGAATCGATTTATTTTGTGTATCTATTAGATCTGTTTAATTTTGAAGTTCTAATGAATTGAACCGGTTTGGTTTAGG

>AT5G54640_H2A_A_thaliana ref|NC_003076.4|chr5:1-26992728:22212767-22213766 Arabidopsis thaliana chromosome 5, complete sequence

TCGACTTCAAAACATGAAGGAATGGATGACAATAACAGTTCGTTAATGATCAAAACACAATAACTCTAATCTGAATTGTGAGTTTGTCAAACTTTGAAAGTGCATTTATTACGAATTGTAGATATCGCAAAGGGTAGATAATGGAAGTAGGTGTGGGTGTCGTTATAGCCATATTGAATTCAAAAGGAAAGACATTAAATTAGAAATTGAATTTTGAAACATGTTGATAGATCATGTCCTTCTTCTGGGTTACCCAGTTTTGCCCTAAAACCTAAAACCAACAGGACCATCATTTCGACCACACCACATTGACTGGTCTGCCCCAATCTAGCTATGATATATCTTAATTTCCGTATGACTTGGATCCATAAATATTGAAATAGATTTGGTGAACACAATTTACTCTTAAAACTTCTTCTCTTTCATGCATGTTCTTTTTCTCACTTTAACATTTTTATATAGTGACATTTTTAGTAATCCAACGTTATTTATATGATTAGTAATTCATCAAATTTATATAGTGATAAAATTCCACAATGGTTGTTCAATAAAAATATGAACAACACAATAGAATTAGTAAAAGTGACTATGTTAAATCATTTTCTTCGCTGGGGTTTGGTGGGCGAGTTCTAAACCCATAAGCGGCCCATTTACTTCGTAAACTCAATTCGATTTGTTCAGCGTTCCAAGCCCATAATATTATTTTCAAGGGCATAAAATAAATTGAGGTTTATATGGAAAATTTGGAAATTCCCTCGTCCAGAAGAAACCAACAAAAACTGCAAAAGTTCAAGCGGTGGGAGAAAAAACTTCAGATCGTAGCCATTCATTAAATTATAATCAACGGTTTAAACCTCTTCGATCCGCGTACTCTATTCTTATTGGTCAAATAACTTAATCCTCCAACATATATAAACAACAATCAGATTTCTCTCTGTTAATTTCGTCAAGAAAAAAATTCGATTTTTTTGCGCTCTTTGTGGGTTGTTGTTGTTGAAAA

>AT5G02560_H2A_A_thaliana ref|NC_003076.4|chr5:1-26992728:574478-575477 Arabidopsis thaliana chromosome 5, complete sequence

TTCATTGCATTTAGTGGAAACAAAATTGTTCATTGCATTTGATGAGAATATAGATAATTGGTCTGACAGATCTTATATATATTGATAATATGAACAAACTTCCGATAAGCTTTGTCAACATAAATAAATTAAGAACCCAAATTATTGTAATTTGTTTTAGTTTTTTTTTGTCTAGAAGACAAACATTTCTCCCCCATGTGAGGAGATGTCAGTCTCGTTGGAATTTAATGTTGTTATACGTTCCATAGAGCCATACGACACGAATACAAACATGTATTCAATTTTCTGTGTCTGATTTTTCTTGTTGATAAGATAGATTTAAATGTGACACACAGATGGTGATTTCATATGGGATTGCTGATTAAAAAACTCAAGTAATTTTACAAAAGTACTAGTGCTAGTGCTAGATATTATATCTAGCTAGTAACTTCGATAGATCATCAGTGTCCAGTGACACTGATTTAGTGTCCAATAGAAAGCATCCAAGTTTTTGCCAAAAAAAAAGAAAGAAAGCATCCAAGCAATACATATAAGTTTCATTTGCATTATATTCAACAGTACCATTTTCATATCTTGTTTCAAAAAATACATCAAATTATTTTCCAAACCTTCACATATAATTTGAGAAGAAATATTACAAATTTAATATAGGTTCAGCATAATTTAGAAAATATTATTCAATGTTTAAAACTTCTCCTAAATTTTGGAGTATTGCTATTAATCCTTTTAATGTGAACAAAACATTGAAGCGAAGGTTGCCAGATCAGCAAATCATAGCCGTTGATTCACTTCCAATCCAAAAGCTAACATTCATCAACTGACAAAACCAACCAACCAACCAACTTCTTTCGCTATCTTACGCCAAAGCTCTCTTAATTCCTCCGTTTGCATATTTTCCGGTCAGATCAAAATCAGAATCAGAATCAAATTTCTCGTCGTGTCGGAGTAAATCAAGCCATGGATTCCGGAACCAAAGTGAAGAAAGGAGCCGCTGGAAGAA

>AT5G27670_H2A_A_thaliana ref|NC_003076.4|chr5:1-26992728:9793332-9794331 Arabidopsis thaliana chromosome 5, complete sequence

GCCTCGAAGAGAAAGGAAACCCCGAAAAAAACGAACACGAGGACGTTCTTCGACGAAGTTACATCCATGGAGAAGCAAAGAATAAGAATCTCAACCCTCTTTAAGTAATGAATCCACTTTATATTCTTTTACCAAACCCATTATTCCATTCATGGATCTTTATGGAAGCATAATACTTCTTTCTACAATTGCTATATCAGGCTCCTTCTTTGCTCTGAGTCCCATCTTGAGGCATAGATACCGCGCCATCGAGAAGCTGAGGCTTGTAAACGAGGCTTTGGTAACGGCGGAAGAGAACGTAACAAGGCTTCAAGAACGTCATGATGTGATTCTCAAAGAGATATGTTCTTATTATCTCGTTAACACCGAGCTCCATGAAGCTTTGGTTTCATCTCGTGCAGCCATTAATGATGCTTCTTCATTTGTCATGGAGCTTAGAAGATTACAGTTGAATATCTTAAACTCTCTTTCTTAATCACATTTTTTGTACTTATTACACTAATTAAAACCAGAGTTTGGGTAATAATTTTTGTTTCCTTAATTTTCCGAATTATCCGCTAATTTTCTACTCTAATTCTCTGGATATTTTAAATAATAGTAATAATCTGCTGTCAAAATAAGATAAGAAAAAGACATAAAGCTGATTATCTTGTAGAACGTGTGGGGAATGAATCTAACGGCTGATATCACTCAAGTGTTCTTTTCCACCTTCCTTTTACAACACCCACGTGTAATGTCATACAAAGAAGTCATTACGACCGTTAGATCAAAGCCAACAAGATCCAATCTTAACGGCTAAGATAAATTACTACACGGATCGCCAACGTGGCAATACGTGGTATATACATACACGTCGTTCTTTCCTCATTTTAAGCAAATCGTAAACCGCCACAAAACCGAAAAAAACACTAATTGTGCTTTCCCTTTAGATTCATTTGTATTTTCTTTTGGAGCTTTTGAACAATGGAGTCATCACAAGCAACGACGAAGCCAACGAGAG

>AT2G38810_H2A_A_thaliana ref|NC_003071.3|chr2:1-19705359:16226821-16227820 Arabidopsis thaliana chromosome 2, complete sequence

CGACGGATCTGGAAATTCTGAAATTTTGTGAAGCTCTTTTCTTTTTGTTTGGTTTCTGTAGATATGGCTGGTAAAGGTGGGAAAGGGCTTCTAGCTGCGAAGACGACGGCAGCAGCTGCAAACAAAGACAGTGTTAAGAAGAAATCCATCTCTCGCTCTTCTCGTGCTGGTATTCAGGTATCCCTCAAACCCTAGCTCCTTTTTTGAGAATCGAGTGGCTCGGAGTTTGAATGTGCGTTAGGTTTTTTTGATTATGTTCAATTGTGAATTGGGAACCAGATTTGTATTTCGTTCTGTGTTTAATGCATTTTTGGGAAATTGCTTCCTCTCTGATTTCTGGAAATATGTTTTACTCTGTGTTTCTTCATTAAAGTTACAATGTGTGCTTGATACTGGACTTTTATTGTCTCTATGACTCTATGCCAAGTAGCATTATTTTTGGTGTGTCTCATTTTATGACTGTGATATGGTAGCTTGCATGTTCTATACGGTTGATACACACAAGCTTGATTTCTCTGTGTGCACTTCTTGTAGTTGCGTATGAAGAAAAACAGTGCTATCTATCTAGATTCTAGAGTAATTTGTATACAATAGAGTACTACCAATTGATACTGAGCCTTAATGGGAGCATCTACTTGTCCTCTCTGTGTGTGTGTTCTGGAAATCTAAGCCAAACATTGTCCTGTTATTGTCATTAGTTTACTTTTGGGATTCTTCCTTGTTAAAGCCGAATTGTACATATCATTGAATCCATGTTACTTATATGGCTTATTGCTGCAGTGTCTTTTATTATGATAATCACTTGATACGTTGTAATATCTATCTATAAGATGTAGTAAGTGAATGATCAAGCAAATTAAAGGACTGTGTGGTTAGTTTAAGTGTCTTATTAATATATATCTATCTACAAGAAGATCTGTCTCAGTCTGATTAATGGGAAGCCTTTCTCTGTGCCCTAAAGTTATGTGCTTATTTTGTTTTCTCAATGTGGTATTCTTTC

>AT4G27230.1_H2A_A_thaliana ref|NC_003075.3|chr4:1-18585042:13637727-13638726 Arabidopsis thaliana chromosome 4, complete sequence

ACAAGAAGTGAAGAAACAATTTATTTGGGCCGAACAGTGTTAAATTTTGGGCCAGATAACGTTAAAATAAAAAGGAGTATTTCTATTTAACAAGCCCAATATAGCCCATATAACAATCCATTGAAATCATCGGAGAACCAAAAAAAGGACAAAGCAGGTGGGCGCACGAATCTCAAATCACGTCCCTTAAACTTGTAACAATCTGACGGTGTAGATTATCGATCCATGCAGTGTCATATCATTGGTCAGAAATATTTTCTATCTCGCCACTATATTAATCATCATGGCGGGTTTCGCTGATACTCATTATTGTTATTTTTGACAGAGAGAAATTTCTCAGTTACGCTTCATCCTCCTCTAAGAGATCTTTTTTCTATCTTGGGTAGTAGAGAGAAATGGCGGGTCGGGGAAAACAACTTGGATCTGGTGCAGCGAAGAAGTCTACTTCTCGTAGTAGCAAGGCTGGGCTTCAATTCCCTGTTGGTCGTATCGCTCGATTTTTGAAAGCCGGTAAGTACGCCGAGCGTGTTGGTGCCGGAGCTCCGGTCTATCTCGCCGCCGTTCTTGAATACCTCGCCGCTGAGGTAATCAGTCTCTTCTATTTATCACCTGTTTAATTTACTCTTTTTACCGAATTAAATGGTTATAGCTTGCATCTAGGGTTCTGGATTTTAGATTTTCTTACCCCTTTCGTTAAATTATGCGAAATTTGGAATATTTTAGAATGCATTAGTTCCTTAGTTTGTTTTTTTCTTGGGAAAAATTGTCCATTTTTTTTGTGTAGTTTTGAGCTCAATTTGTGTTTCTTTGTGCTCATCATGCTTATCGAAATTAGGGTTAAATTTGTTCCTTACTACTTTGAGTTATCATAGTTGGCACTGATTGATACTGTCAATTGTGTTCTCAAATTCGAAAAATGTTGTTGTTCACTTAGTTTTGTCTTTGGATATGTGAACATGTCTGCTTGGGAACTGAATTTGGTGCGCTCACTTTCTATAGG

>AT4G27230.2_H2A_A_thaliana ref|NC_003075.3|chr4:1-18585042:13638331-13639330 Arabidopsis thaliana chromosome 4, complete sequence

TTTGTATTAAAAATTCTCATTTCTACAGTAACCTAAAAAGCTGTCTGTTTTCATAAATTTCCAATTACTTTTTCCTTTTCTTTCTAACACGAATAATGATTTTTTTAGGTCATGAAACGAATAATGACTATGTTGTGATTTTCAAGCATTAAAGACATTTCAACATCCTACACACTAAAGAACAATTCAAGTATTGTACATCCACCGATCTTTTGTTAACATCATAACCATCCCACCACGTTCACGTCACTCATTTTTTCAAATGTCTCTCATTGGGTTTACTTTCTTTGTTATCCATCTTCGGAGAAATTAGCTTGACATTAATACTTTTTTGACAAATTGTAACGTTTTCTCCGGAGATGGATAACAACGAAAGTAACGTTGTCTAAATTAATATTACAACTTTTGAGTTCTTACTAACTGAGAAGATTTGTGGCTTTTCAGCCACCACAATATGTCATACAACTTGCAACTGTTATTATCCAAATTTAAACCCACATAAAGAATACGTCTAAAAAGCAAACAATAATCATTACAACACTTAGTAAGTTATAACTTCTCCCTAACTTCTTTGAAATTTTGATAAAAAGGAAAATACATATGTACAAGAAGTGAAGAAACAATTTATTTGGGCCGAACAGTGTTAAATTTTGGGCCAGATAACGTTAAAATAAAAAGGAGTATTTCTATTTAACAAGCCCAATATAGCCCATATAACAATCCATTGAAATCATCGGAGAACCAAAAAAAGGACAAAGCAGGTGGGCGCACGAATCTCAAATCACGTCCCTTAAACTTGTAACAATCTGACGGTGTAGATTATCGATCCATGCAGTGTCATATCATTGGTCAGAAATATTTTCTATCTCGCCACTATATTAATCATCATGGCGGGTTTCGCTGATACTCATTATTGTTATTTTTGACAGAGAGAAATTTCTCAGTTACGCTTCATCCTCCTCTAAGAGATCTTTTTTCTATCTTGGGTAGTAGAGAGAAA

>AT4G13570_H2A_A_thaliana ref|NC_003075.3|chr4:1-18585042:7884392-7885391 Arabidopsis thaliana chromosome 4, complete sequence

CTCAAGAATTTTCCAAATCTAGAAACCTTCTTCTATGAAGTAACATACACATTCTTGATATTAACAACTGACATGATTTTACACAGTAATAAATTTTGAAACGGTCTCATTTTATGTTTCATGGTGTGCAACACGAATATACTAAAAGATGTGTCGACGAAGATAAGTGCTTTTGAAAATGTTCGGATGATTATGGTGGAGGGAGAGATGGTATGATAGGAGAAGTCTTTTGCTCATAGAAGTAGAGTGCTAACAGTTCACAATGACTTTACAATCTATGTGGCTCCTTGAAACAATAAACTATGGATGTGCATAACTAATGGACAATCTTCATATTTAGGAATGACTAAAATATCTTAACTAATGCTTAAACACTCATGTGTCACCAAATAACAATACATGGAACATGAGTGTCAATAATGACCTTGTATTGTAATGGGTCGCTGGTTTAGTTGAAGTTCCAGTAGCACATACCGAAACTACATTCCTTTTTTATGGAGTAATTCTGTTTTAGGATATTTTTAGGGTTTTTGGATTTTGTATAAGACAAAAAAAAACACAAACACAATAAGCTACTTAACTAGAAAATAACATCATCATATAATTTGACTAAATAAACAAATCACTTCTTCGTGGTTTTGTTGATGAGAGACATGTGGATGTGAGAGACTACTCCTTATCCACCAATTGTTACTTTGATAAATGGATCAAGATCCCTATCTCCTGCGATCACCAACTATAAATGCATTAGAGTAATCCTCTTTATTTTCTTATCATTGATTGTGTTTTTCGGTAACTCAATAACCTATGAAGTTAGGCACTCTAGGATTGAAGCCATGTAGTCAACAACAATAGCACCAAGTCGACCATGTTGTAGATACTCTAGTCTTGAGTTGCATGTGAATACGACCCACTAGAAATTGAAATAAACAAAGAAATTTCATTTTTTGTAGTATAATTTGATAAAATTTTATACTGATATTGTTTCTTTGTTTCTTTC

>AT5G22880_H2B_A_thaliana ref|NC_003076.4|chr5:1-26992728:7652510-7653509 Arabidopsis thaliana chromosome 5, complete sequence

TAGACGATATCTCAAGAAATGACACGTGCATTCACTCCCTCTCTTTTTTTTGTTCTTTTTCTTTTCACGAGTTTCTATATTCTCTAAATTAATGTCACAAAGATAAGATAATATACAGATTTGTATCATAGTGACTTGTGCATTAACGTTAATGTTACAAATGATTTTGGTTCTGATGGGTATCATGCATAGATTTTCACTAATCTCAGAAATATGTGGAATCAGTGAGAATCAAAATTTATGTTTAATACGTCTCTCACTTCTCAGATTAATCAACAACTATCTATTGATATATCATGCAGAATTTTAAGAAAATATAAGAAACATCGTTAACTCTGTTAAAATACAACTACATATTAACTTATTAAGATGAGTCCAACTTATTAAGCTGAACTAAATGTTTGTATTTTAGAGAAATTCAAAATTTGTATCTATAAAAAGTTGGTTATTTTTGAAGAATTTTGAAAGAGTTGAAAACTTATAATTATGCATTAGTTCTTCATTTCCATAAAAATGTTAGATAGGAAAAAGTTGTTACCAACTTTGTAGATACGGTAAAATCATAGCCGTTCATTGGTAGACGGATCAAAAGTAACCACGTCAGCTATCCACGCCAAAAACTTTTTCTCTCTTTGCCGCTTCATGAACTTATGGCGGATAACAAAAAGCGCAACAGAAAATTCGAATTTTTCAATATTTCGAAAAAGAAATTTAAAATCACTCGCGCATTAAAGTAAAATAGATCTGAACCATCCATTCACCAAAACCCTTCATCGAACAGCACTCATATCTCCACGTCACGCATCCGCGTTTCAGTATCTTAACTAATCAGATCTCTTCCTATTCTCTTATATAAACAAACACACAATTCTCGAAGATAACAGTATCAAATCAAAACCATCACAGCAAAAAAAACTCACTCCCTAGAAAAGTTTGAAAATGGCGAAGGCAGATAAGAAACCAGCGGAGAAGAAACCGGCAGAGAAAACTCCGGCAGCCG

>AT5G02570_H2B_A_thaliana ref|NC_003076.4|chr5:1-26992728:577117-578116 Arabidopsis thaliana chromosome 5, complete sequence

AACAACATTTAACATTTTGATATACTTTGTAAGGCAGGAATGAGTTCGATGTACTTAAGAGTTTCCCCACATCCTTGGGAAGGCTTTGGTTGGATTCTCGAACATTTTTAGGGACCACCAAGTTTGGTAACTGTATGTGTCGAAAGAAGTGTTTCTTGGAAACACATTAGCTGGTACAAAGTTTACATCATCAAAAGTTCATAAAATCACTTATACCATAATGTCAAATATACATTATACTTCTTACAAAACTTATCCATCAGCTGGTACCACTTTCAACGTATCTTTTGCATGTGAATAAAGTTAACTTTTAAAATATTTGATTAACTATATGGTACTGAATTACTGGTCCTCTATTTTATTTTTTTTACATTGTTGTTCATGCATATATATACCTAAGAAAAACTCATCGAAGTTTATTTGCTAAGTGATCATAGGAATTGATTGGTAAACTCTTGTTATTATGTTAAACATAATGATAATATATATTCAAAAGGTTAATTAAACGGTAGAAAGTTATTTGTAAAAAAGGAAAACGACAAAATAAAACTTTTATCTAAAAAAAAAAAAGGAAAAGACTACTGGAATTTCGTAAAGCGTTAAATACCAAGAATAATTAATTAAGAAAATAATTGTATTTAGTTTAAAATGTTCCAAACACCAAAATCAATTAAATTCCGAAGAAAACACTCCAAATTCACGTGGCGACTTTCTCAACGGGCCTAAAATATTTTCACCTATTTTATGTAAATATATTAGTCAAGGCCCATTAAAGCCCACATTAACCGCCGACTTTAGCGTTAACCGTTGATTAGTCTGACAACAATTCCTCATCTAACGACACGTCATCGATCTATGTCATATTTCAGGATCCAATTATACATCATTACACGAAACGTATAAATTCAAAACCGAAACCCTCAAAAATCATCTTTCATTGCCCAGAAGTATTCATCACAATCGCTGTATCGTGGATTAATGGCGCCGAAGGCAGAGAAGA

>AT5G59910_H2B_A_thaliana ref|NC_003076.4|chr5:1-26992728:24143496-24144495 Arabidopsis thaliana chromosome 5, complete sequence

CTAAAAAGCACATTAAAAACGTCACTTGGCTTCAGCGCACGAAGCAGAAGCGTCTGCAACAGTGATGAAGCAGGCCAGAAGAGATTAGCTTTGACCAGAGCATGGATCAAAATACAAAACGACGCCGTGGAATGGTCGAATCCTCGATGCAAACCGAGGAAATTGAAAAACCTCAAACCCAATTTGGGATCATCAATGGTTCCAATCAGAATCTCTTCAACATGAACAGTCTTCAATCTTCTCGAGACTAGCTCACTGCTCAATGCGATCTCCCAGCTCCGTTTCCCTCGCACGATTCTTTTGACAGCATCGACGAATTGCTTATCTTCAGCGATTCTACATTCTTCTTGTGCAGAATCGATCACAGAGCTCACATTTCTAAAGCTATTGCGAAAATGAGAGCTTGTTATTGATCGAATCGTGCAAGGGAGCTTCATCAAAGAGGTCCACCGTGAAAATCAGAACCAGCATTGGTCTGTGTTAGTGTCGCAACAACGAACCGATATCTGGGGACCCGGACGGGTCGGGTCGATCTCACTGGTTATAATTGGGCCTACAACTGGGCTCATTACAATACATGGGCCGATGCAGAATTTTTCGAGGATGGCGGGAATGGAAAATTTCACTAGATAGCGCCAATCTGGGACACGTCAATAGTCCTGGCCACCAAATGTATCCAATAAGAACCGTCACGTGTGAGAGCTTTCCTCCTTTTCAAGATTCCTTTTCAACCGTCGATATCATTCTATTAAAAAGCAGATCAAACGGTACAGATATCGATCCGCGTCAACTTAATGTATCCAATCATATCACTCCATAGGATCTATATAAAAGCAATATCTCAATTTTTTCTAGGTCATCAAGCAATCCAAAGCGATTAAACCTACTCAATCTCAGATCTCGTTAAACCTAGAAACCTCGAGAAAAACCGTATCAATGGCGCCGAAAGCAGAGAAGAAGCCCGCTGAGAAGAAACCAGCTTCCGAGAAGCCGGTGGAGG

>AT3G53650_H2B_A_thaliana ref|NC_003074.4|chr3:1-23470805:19899343-19900342 Arabidopsis thaliana chromosome 3, complete sequence

CAGCTTCTTGTTCTTCAACTCCCCAAACAACATCTTCACCGTCATCATCATTATCAACTTCAGACCTTTCAACGTTGCCCTGAGTTTCACCGTTAGGTTTATGGCTTTTTAAAGAATCTAAGTCATCTTCTGCATCATGCCTAACTTCCCCATATCCATCACCACCACTTTTATAGATCTCCTGATCACTCTGGTCTTCTCTTCTTCCTCTATCCCTATCTTGATCCTTACGTCTCTCTCTTTCATTCTCCCTCCCAGTCTCTCTACTACTAGACCTAGCTTTATCTCTACTACTCCTACCTCTGTAGTTGTCTCTATTGCTTTCTCTATCAATGCTTCCCTTCTTCTTCAAATTCTCCCCTTCCTCCAAATCAAGTTCACCATCGTTACCAGGATTCCCCAAAAATGGATAAACATTCTTTTCATTGCCGTCCTCATCAAGATGATGACTGTGTCGATGCTTGTGATGATGGTGCTTGTGCCGCTTGGAAGATTTAACGACTTCGTCGGAAGGAGAATACGACCTACGGTGTTTGCGGTGGCTTGATTCTACTATCTTATCGCTCACCATCAGATCGTCGGAAAAGATAGACAATCGCAAAATTAGGGTTAGGGTTCGATTTTCTCCCGCGCTCTCTCTGTCTCCGTGAACAACAGACTTCCATTAAAAAAATTCACATTATATAAAAATTACAGTCGGCATCGGCAAATTAAAAGGAAATCAGAATCGTCTATTTAAAAAAACAAAGGGCCCAGGCCCATTAATAATAAATGATTAAGAGGATAGAGCCATCATGTCCGTTGATTATTCGAAACCTTTCCGATGGATGGATGACACGTCATCGATCCGCAAGAAACTCGGGTAACCAATGAAAGCCAGCAGAAGCCTCTATATAAACAAATGTCTCCTCAGTTTTTCCATCAGACGCATCTTTCAATTGTCAAGTGATTGTATTTAACCACAATTTCTTCTCATCTCTAAAGTCAGCATCAATGGCTC

>AT3G46030_H2B_A_thaliana ref|NC_003074.4|chr3:1-23470805:16924988-16925987 Arabidopsis thaliana chromosome 3, complete sequence

GGAACTTGATGATAACTTTAGAAGAAGGCCTGATCATAACCTGCCTCTTTCCTCTCTTCTCTGCATTGTACATACTCTTAAGACCATCATTGAGCACACTGATTCTCACCATTCTTCAAAATTTCAAAAACTGCAAAAACATAAATCAACAGCTTCACGAATCTTATTGACATGTACATAAAAACATCTACAGAGGGAACGAATTCGATTCCCTAAAAATTACACACTCTGAAAATGACATTGGAATCTAGATTCTCTGGAGAAACATCAAATTGGGGAGATTAACTAAGCCGGTGTTCCAACAAAACAATAAAGCAGTTCATCGGATTAGAGGAGAGATGGAGATTCCGGCGATTACTAACCTGAAACCTAAGTAGTAGCCGCCGCTCCGACGTCTCTTGCTCGCTTGGCCTTGAAAAAGAGATTAAACAGAAGACGGAACTATTTTTAGGGTTTTCGCTTTTATCGCTTTCACGGGACAGATTCATGTATAACTAAGCCCATTATAAATACATAACTAGGCCCATTATTGATACATAATTAGGCCCATTTGTAACCAAACATGGACTTTGTATCAACATTGATGGCATTCTCTTAACTGATTCACGTCTAGGACTAACAGATTATTATTTAGGTATTAAGCCCATTTTAGCCCGTTAAAAGATAAAACTCGAAATTAATTTTGATTCGCTGCTTAATGGCCCATTTAAAAAGGTGAGAGATGCTTAGCCGTTCACTGCCAACGACGGATTTATTCTAATCTAACGGTACGTAATATATTCCACGTCATCAATCCTCGTGAAACGAGAATTAACCAATCTGTATCTAATTCATGGTCATATATATACATGAGCTACCGAATCGTCTAAACAACAAACACAAAACTTGATCTCACGACTCCATCTTCTAAAATTCAGAATCTTTCTTTGAAACTTTTTTCGTGTGAAGAAAATGGCACCAAAGGCAGAGAAGAAGCCGGCAGAGAAGAAACCAGTAGAGG

>AT3G45980_H2B_A_thaliana ref|NC_003074.4|chr3:1-23470805:16908866-16909865 Arabidopsis thaliana chromosome 3, complete sequence

TCCTTTCGATGTTTTGCGGCTGTTGATTGTGTTTTCAGTTTTCACTTATGTGAAAAGGTAGATAGAGTTTTTCATTTATGTGAAAAACATTCCATTTAAAATACCTGAAAAAAAGATTTGGATTAACTTTTAATGCCATGCTAAAACACACCAAAAAACTAAATTATTGATATACAATGTTGTGGATTATATATTGTTCCCATGATTAAATATATATGTTCTAGTCAATAGTCATACATCATGCATATATAGTAGCCTAATGTAGCTAGTTTCTAGATTTTACTACTCTCCACTGAAGATAATTTGAACCATAAACTGGAGTATTAACACATATTGCTCAATATAGCCAAGCACAATTTTTTTACTTCTTGTTTATGCAAATACAACAAATTCTTTATATTTTAGCTGTCTCATATTATTAGTCAACAAATTCATTTTAAATCAGCTTGTAAGTTGTAATCAGGTTCAGTTTTACAGCAAAATATATTGTGTTAAACTGTTAGTGCAAGCCTACTAGTATATATATTCTATACTCTGTTTCTTCACAGATATGTTTAGTTAGTTTTTTCATAATTGTATAGACCACATTAATCAGATCTGCTTTGGATCAGGCTGATATTAAAATTTTAAGCCCAATAAAAGCCCATTTACAATACAGTGGAGCGGGAACGGAGATTTTAACACCGCGCTAAAACATAAACCAATATTGACACGTCATCGATCCCGCTTCATAAGTTTATAAACCGTCGATTAAAAACAATTTCAACGGTTGCGATCTCCCATCTCATCGATCCTCGTCAACATCTAATAACCAATCATATCAATTATTCTAACTACTATAAAATCAAAACTCGTCTTCATCAAATTAGATATCAAAATCACTCAGATCTGTGCTTCTTCTTCTTCACCCAGAAAAGTAAAACAAAAGTCAAAACAATGGCGCCGAGAGCAGAGAAGAAGCCCGCGGAGAAGAAACCAGCCGCCGAGAAACCAGTAGAGG

>AT3G09480_H2B_A_thaliana ref|NC_003074.4|chr3:1-23470805:2915255-2916254 Arabidopsis thaliana chromosome 3, complete sequence

TCCTCTTCTCTATCTCTGTTAATTTAGGTTTAGAGTTGTAATACTCCATCACCAAGTAATGATAAGCATCGACGCGTATCGGATCCTTTTCGAGAATCTCCTCGAAAGCTTCGATTGCTGATTCACTTTTCCCGTGGTATGAGAGAATCCGAGCCTTCATAGCCGGCCATTTTCGTTCATATGGCTCGATCTCGATCAGACGGTCCAGAAATACAATCGCCTGCTCATGTTTATCGGACTTAAATTTGATTTCCGTCAGCAATGATAAAGCATTCACATCGTTTGATTGTGTCTCGAGGTGCTTCTCGAGTGTCGTCTCTACCACAGCAACTGGAGTCATCGCGGCGCGTTTGTTGACGAAGTAAAACGATGCTGAGAACAGAGCTGCGGCTGCGGTGAGGCTGATGAAGGTGGTTTTAAGCAGTGAAGGTTTATGATTTGACGCCGACGAAGATGATGAAGCTCTTATGACGGGAAATCTGATTGGTGTTGACTTTGCTTGGTCTCTGAATAAAAGAGGAAACGAGAGTTTTGAAGGAGATTTTCGCTCGACGAGGTTGATCTGAAGGTTAAGTTTTGTTCTAGAGAATTGAAACGTCGCCGGAGACGCCATTTTTGCTTCTGAGAAATCCCAATAACTAAGTCAACGCTGAAATGGAAAGAGAAGAACAGAGCAGATTAAAGAAGAGCAGAGCAAATAAGTTGGAAATCGACGAAATTTTTTAAAAAGGATGCCGTCTTGTAAAAGGCCAAGGCCCATTAGATGAGCCCACGATTAGTTCGGTTCCTCTCGTGAATTCAACCGTAGATTATCTTGTATCAACGCACGTGATAACGCCACGTAGACGATCCGTGAGATTGATCAAAAACCAATCATGTTCGTATCAGCACCTCTATATATATCTCGGTATCACTCGGAGAGCGATCATTAATCGTCAATTTACAACTAGTCGCGAAATCCGAATTTTATCCAAAGCAATGGCACCGAAGGCAGAGAAGA

>AT2G28720_H2B_A_thaliana ref|NC_003071.3|chr2:1-19705359:12333217-12334216 Arabidopsis thaliana chromosome 2, complete sequence

AACAAAATAAGCTTATTACATGAGTTACATGGAATTCAATAGAGGAAGTAAGGACTCTTCTGTCCAGAGTTCAATTTTAATTATAAATAATAAAGTCATGGTATTTTTAGTAGGTTTCGGTCCAAGATTAACAAGATGGCTTGCTTTTTCAATGAGTCATTTCACCATAGTATTAGTCAGACTTGTATGTGATGTGTCGGATGGTTGTGTCGCTATGGATGGTCCTTGCAAAGCAATTTGAATTTTGTTCTCTCCAATACGATGTCGACTTAGATCACTTAGTAAGAAAAGAAAAATCTAATGATTTGACAACATGCGGTTTGTTTGTTTTTTAATGGACGAGTAAACATGTCCATTGCTCGTATTCAGCAATTGTTAAAATCTAGTCTATGTTCGAAAGTTTGTTACCGACAATGAGTATGCAATAGAATTTGAGGTTGGCTTTTGTATTATGAATCTTCACACTCATCTTAAGCTTATATGATCGGTTCTATATGTAATAAACTTATCTTTTGTTTCGGTTCCAGTTTCAATTCTTTCAATTCGGTTATTTTGTCCGGACCTAGTCCGAATCCTTTAATCTCGGTTTAATTTATAAGCCCAGGTCTAACGTGATCCACGTCACCAACGATACTGAACATAACTCCACATTAGCAATCCGATGCCTTCTCCAACATAAAAGAAAAACACTTACTTATCATCATCTATCAGCCGTAGATTAAGATATTTAGTATCCTCAATCTAACAAACACAAAATCACCACGTCATCAATCCTTGTCAAATTCTCTCATCCAATCAAATCCATTACTTCCTCCTTAATATCTTTCACCGCTTCATCATCTTCTTCATCATCATCAATCACAAAAATCCAAATCCCAAAACTTTCCCGAGAAAACTAAAAAAATGGCACCAAAAGCCGGAAAGAAACCAGCAGAGAAAAAGCCCGCAGAGAAAGCTCCAGCAGAAGAAGAGAAAGTAGCCGAAAAAGCACCAGCAGAGA

>AT2G37470_H2B_A_thaliana ref|NC_003071.3|chr2:1-19705359:15742917-15743916 Arabidopsis thaliana chromosome 2, complete sequence

CTCTTGTTCCCTACTAGAAGAATCTTGTTGACATTGTTAGAAGCGTGCTGCTCAATGTTACGGATCGGATTCCTGATGTCTACTCACAGTAATAAAAAAGAAAAGAAAAGAATGAATGTGATCTTAGTAGATAATATTTGCTAACTAATATGTAGAAGTAAGAGAAAAAATAGATCTGTTGCAAATGTAATTTTTTGTTGCACAAGGCCTGAAATTGAACTAATTAAAACTGTGATCTAAAACTAAGAGTAAAGATAATAGTTTAGTTTAGAAAGATGATTCAACTGTGACTTGATACACAAGTAAAATGCCAATAGCTCCAATATGTAGTATGTTACGATAAGATCAAACCAGTTACGCGATATTTCCAGATTCCCACCATAATAAAGAAGGAACACATCCATAAAACAAACAGCTAAATAAAGGATAAAAAAGCAAAATTAAGTGGATAGATTCTTAGAGAATATCACAACTAAACATGTTGTGATTATAAAAAAAACGGTCTTATCACAAATCTGATGCTTAATTCACTTCCCATCAAGCTGAATGGTCCATAACTTGAAATTAATATTGTCAACACGCAGTTCCACTAATAAGTGGAAATAGACAAAAACTTTGTCTCAAAGAAAGGAGAAGCAGAGAGTTTTTGCTCGTTGGCAAAAGCTGTGACTGTGATGAAGTGAAGGCTTTAGCCTCACTAGTCATGGGCCTTGAGCTATAACTTTCAGATCCAGCCCAATAAAAGGAGCGATGCATCAGATATTCAGATGGCAGAGATCCTAAATAATCTCAACCGTTCTAGAGATCCTAAATAATCTCAACCGTTTATTTATTTAAGCCACGTCATCGATCCTCGCGAAAAACAAATACCCATTTACAAGCAGTTACATATCTGTATATAAACAATTATCATCATTCAGCATCAACGCGTCTCTCAATTCTCACATTCTCAAAATCACTAAAATCAAACTCAATTTTCATCAAATATCTCTAATGGCGC

>AT1G07790_H2B_A_thaliana ref|NC_003070.5|chr1:1-30432563:2412122-2413121 Arabidopsis thaliana chromosome 1, complete sequence

TTCTTGATCAATAATTCATAAAGATAGATAATTGATCAAATATTCAAAATAAATATTTAAGTTTCTTACCATCTTGCCTTAAGCTCAATCCTCTACTAAACAAGTCAAACAACAAACAAGAGCTTGCGTTAAAATAAAATCTGCAAAGCACTAAAAATTTGATACAAATGCACAGATTTACTCATCATGAATATTTATTTCAATGGGAATAAATCAAGCTTTTAAAATGTAAACCGAAATCACTTATAGAAATTGAAACAAAGGTCTCGTGTCCAGAAAATACACACACAGACAAAATAGAGCTAAGACTGACTAATTCGAAAAGCGTTCTTAAGTTCACTATGAAAAGCTCTAAGATCCTAAATTTTCTGATGTGAAGCAAGAGAAAAAAACAAAGTAAAGACCAATTAGCAGCTATCTCACACTTCTGAATATTTCTATAAGCAGATCAGATTCTGAACGACTATAGTGAAGATCAATTGTACCTTAAGATTGGGAGCTCGAGAGGCAAGGTACTCAGTGACGGAGAAAAGCAATTAGAGGAAGAAGAGAAGGTGAGCTAGAGTTTTCTTATCGCTTTTTATACTAGGGAAAATCAAAGAAACCAATTGACATGTGTTATGCGCTGCGTCTCAAGCTTCATTTCAGGCCCAATGGGCTTCTTTGTCACGACCATTGAAGCCTATCAAAGCTATAACTTGAACCTGTAAAAATGAATGATGTTTCTCTCAGCCGTCAGATCTAATTTGAGCATTGATAAATTTAACGAATGAGAAATATACACGTCAATGATCCTCGTGAAGATTTCTATCCAATAGAATACGTTTGTTCCTTCTCTATAAAATCTAAACTATTATCACCATTCACCACTAAACTACTTCAATCAACTTCCTTTTCCCGAGAAAATCAGAAAGAAAATTCACGATGGCACCAAGAGCCGAGAAGAAGCCCGCCGAGAAAAAAACCGCCGCTGAGAGACCGGTGGAAGAGAACAAGGCTG

>AT1G08170_H2B_A_thaliana ref|NC_003070.5|chr1:1-30432563:2563348-2564347 Arabidopsis thaliana chromosome 1, complete sequence

AATTAAAGATTAGATTCATAATGATATCTGTGTTATGATAATAATTACTCGTCCCTAATTACTCTCTCCTAAGTCCCAACGTTCTTTATATCTGAGATCCTTTTTTTTTTCTGCTACCACAAAACCATGAAATAGAAAGAAAAATACAATACGAACTAGAAAAAAAAAGTTTTTTAGCGATCAGATAATTAGCGGCCAGTCCAAACATGTTTCAAATTTGTAATGAAATGGAATGAAACCTAATTACATTGTTTATGGGAACGAAATTCTTTTCTAAACTGGTGCTGGTGAAAGGGTGATCGTGGTGTCGTTTGGTCACATATAATTTCATTAAAAATATATAATGGAAAACAAAAATTATATTGTGTAAAATTAAGTACGGTTAAGGATGTTGTATTGGGCTTTTGAGACCCGTTTAGATCTAGTCATTTAAGCCCATTTATATGCCCACCGGGCTTTGGGCTAGTTATTTAAAACAGATACGAATCACCGGATAAGTTTTTCTTACTTTTTAGTTTTTATAACCACCGCGTAGTAGACAGTTAATCACCAATGCTAAGGTATTCGAACGACACGTGGCACCACATGTCAATCGCAGAAGTACTGTTACGACCCATCCCAAAACGCCCTTTTTAAATCTTAACCACATAACATTTTCGGTTATTTCTAACGAAGAATATGGCGCCGAGAAAACCAAAGGTTGTTTCCGTGACGAAGAAGAAGAAAGTAGTGGAAGAGACAATCAAAGTAACCGTCACGGAAGAAGGAGATCCGTGTGTGATCACCGAGACAGCGAACGATCAAGAGACGCAAGATCTCACTTTCTCCATTCCCGTCGGAGAAAACGTTACTACCGTTGAGATTCCAGTGGAGGTTCCAGACGAACGGTCACTTCCCGTCGGAGAAAACGTTACCACCGTTAAGATTCCGGTGGATGATCGAGATGAAAGTTCACCTCAGCCGCCGGAGACTCCGGTGGAGGTTCGAGATGAACCTTCAC

>AT5G65360_H3_A_thaliana ref|NC_003076.4|chr5:1-26992728:26137735-26138734 Arabidopsis thaliana chromosome 5, complete sequence

TTGAAAATTAAATACACATGGATTTTATTGCAATAACTATTTAATTTATTATTATTTTATCTATTTATAAACTAAATTTAATGTTATTTTCTTAAAGTTTCATAAATGAATACTAAAGGTTTAATTTACCTCTATCTAGAGTACTTTGATTATGATATATTTTTGATAAAAAGTTCATTAAGAAAATCTGACAACATTTACATACGTATATGGATAAGATTTCTATTAATTGACTTATCTCTCAATTTATTTTAAGTGAAAATTGAACTAAAAAAACTTTTAAAATGACGTTTTCTCAACACATTTTGTAAGTATTCACATTATACAAAATTACAGACACTTTAATTTGTGAGTATAACACGTATTATTAAATAAACAGAAATGTAAGAAATATAAAACATTATTGCTTCAGTAATCAATTTCAAATCAATCAACTAACTCAACTCTCAATCTTTGTATCTAGATTTAAAGCCTGATTGGCTGATTTTACACCACTCACACGTTAATAATAGTTAACGTGCGTCAATGATATTATTATTTCTCGACTTATGTATTCTACATGGGCCACATCAGCAGAAACTAAGATTATAATTTCGTGGACTGATACACAACATATTAACGGGCTTAACCTAGACTCTACAACATGGGCTTTTATTTCATAGAGGCCTTCACTGTCAGGTTCCAAAATTTAGCGAAAAGGTTTAAAAGACGCGCCTAAATAAAAAAACTAAAAAATTGTGAAGGAAGCTAAAAAACTTGGGCGCTAAAGAAAACTAACCTGATTGGCTGCTTTAGACGTGACGCGGATCACTAAAATTAAAACAATCACAACCGTTGATTAAATAAAAGATCTAACGACGCAAAACAAATACTTCCCTTGTATACGCTTCGCTATAAAAACCAATCCAGTATCCCTTTAGTCTCAACATCTTCTTCTTCTTCATCAAATCTCACAAATCTTCAACACTTAATCACAAATCTCAAAGCTTCGGATACCAAA

>AT5G10400_H3_A_thaliana ref|NC_003076.4|chr5:1-26992728:3270889-3271888 Arabidopsis thaliana chromosome 5, complete sequence

TTTTTATAAGCGTCCAATGTTTGATTCGCTTTCCATTTTCTTATAGTTTCCTTTTAAATCTTTTTTATATACGTGAGTTTCCTTTTGCAATCATTCAAAATTAACATTATTTTTTTGGTCGACATCCAAATTTGATTAATTTTACTTTTCTCAACTATTAATTTGGTAATATATCTTTTCATTCTAAAGTCGTCGTTCATTCATTTTATAAAACTTATATTAAACATGACGAGTTCATATTCAACCTCATAAGACCAATAAAATGTTGTTATTTGTTAAATAAAATGTTGATAGGAATGTTATAGAATAAGTCTGGCAACTACTTTGTTATTTCTATTACATATATAGTTTATTTGTATTGACTATTGACTTTACACCAATGTTTTTTTTATTTGTATTGTAAATGAATAAGTTTACCAATGTTATATTTAACGAATAGGTTTACCAGTATTGTCATTTATCTAGTATCTAATTAAAGATTAGAAAAGTCAATACAAATTAACTATATAAGTAATAGTAATAACAACCACACTGCAGCCACCAGCAAATTCCATTAACAAAATAAAGATCTCGAAAATTTGAACACCAATTTACGATTTTTTAACGTCAACATGAATGATATATTCATAAAAATGTGTTGAGAACAAATTTGGTCAAGTCAAGCTTTAGATCCGTAAGAATAATTGATTCATGGCTTCTTCCGCATAAACACATAAATCAAATTGTATATGTGAATTTCGGCATTGACAATTTTGGTAACATATGACGTGGATGCTGAGAAAGTAATTCTTATTGGTTATTAACACAGGACGCGGATCGTAAAGATACAAACATTTCTAACCGTTGATAAGCCAAACAGATCTAACGGACAAAACACAATCACTTCCTCTCTACCTTTGAGTATAAAAAAGCCTCACAACATCACTCACTTTCCCATCGATCGCATTCTCACCGAATCAAATCCAAAAGAGCAAATTTACAATCTTGCGAACTCCTAAAA

>AT5G10390_H3_A_thaliana ref|NC_003076.4|chr5:1-26992728:3269476-3270475 Arabidopsis thaliana chromosome 5, complete sequence

AACGAAGATGATGAAGATTAAGAAGACTAGTCGTTTAGTTATGGATTAGCTTGTTTGGTTTCAATGGATATGTAAAGCTGTCTCTTTTCGTTTTCGAGTGTTATTAGTTTTTTACTTAGATGTGTCTTCCAGATGTTCGATGAAATGCCTAAAGGAAATTTTAATCTACTGTTGTCTAATCTTTTGATTCGTTCTGTCGATCTCCATATTTCTGCCACGCTTAAGAATCGTTTATCAACGGAAGAGTTAATTTTTTGCTCGATGAAATTGTCCAAATTGATTTGCAATAGTAGCTGCTTAGGGATTCTCAGTCTTCTTCTCCATGAACAAAACATTCTACAAATTCTTATTTAGAACTAAATCATTCACTAAACGTAAGAAAAGTATCGGTTCTTTACAAAATCAAAGTGAGAATTCGCAGATTGTTCTATTCCTGGTTATACCATCACAGTAAGAAATCCTCCAAATTATTTTACTTAATGCGTAGATTTGCTGAATAAGAGTAATGTAATTTCTCGAAATTTGTAACTTCAGGCTAAATTTGTTATGTAAATTTTGAAATAATTTCATCCTTATTTTCTACGATTGAATTTTTTTGAGACCCTTCATAGCCAAAATTTTCGATTTGGCGCCTAAAATTAAAAGATTTGGAAGTAGCGCAAAAGCCTACGTGGACTGAAAAGGATGATTTAAATTGGTTGATAGTAAATGACACGGATGGCCGGAATTTTAGTCTATTTTAATTCGTTGATAAGGTCTCACACGGATTTATACCTGATGATCTCAGCCGTTGATTGAAATATTTAATCCAACGGACAAGAATACAACTTTCCCTCCAAAAACAAAACTCCCTCCCATGTTTTTTTCACCTCACCGCCAAATTTCTCTCCCCATAAAAGCAGTCCCTCTTCTGCGTTGATCTTCACTACAAAATTTCTCATTCGCATCCTCCACAAGAAGAAAATTCGATCGGTCGATTAGTCTTTGTTTGATTTCGAAA

>AT5G65360_H3_A_thaliana ref|NC_003076.4|chr5:1-26992728:26137051-26138050 Arabidopsis thaliana chromosome 5, complete sequence

AAATTTAGCGAAAAGGTTTAAAAGACGCGCCTAAATAAAAAAACTAAAAAATTGTGAAGGAAGCTAAAAAACTTGGGCGCTAAAGAAAACTAACCTGATTGGCTGCTTTAGACGTGACGCGGATCACTAAAATTAAAACAATCACAACCGTTGATTAAATAAAAGATCTAACGACGCAAAACAAATACTTCCCTTGTATACGCTTCGCTATAAAAACCAATCCAGTATCCCTTTAGTCTCAACATCTTCTTCTTCTTCATCAAATCTCACAAATCTTCAACACTTAATCACAAATCTCAAAGCTTCGGATACCAAATGGCTCGTACCAAGCAAACCGCAAGGAAATCCACCGGAGGAAAAGCCCCAAGGAAACAACTCGCAACAAAGGCGGCGAGGAAATCAGCTCCGGCGACCGGAGGAGTAAAGAAGCCACACAGATTCCGTCCTGGAACTGTTGCCCTAAGAGAAATCAGGAAGTATCAGAAGAGCACTGAGCTTCTGATCCGCAAGCTTCCGTTCCAGCGTTTGGTTCGTGAGATCGCTCAGGATTTCAAAACAGATCTGCGTTTCCAGAGCAGCGCCGTCGCAGCACTTCAGGAAGCGGCTGAAGCATACCTCGTTGGATTGTTTGAAGACACCAATCTTTGCGCGATTCATGCTAAGAGAGTCACTATCATGCCTAAGGATATTCAATTGGCGAGGAGAATTAGAGGCGAGAGGGCTTAAGAAGGAGATTGAAGTACTCTAGACTGTGATCGTTATGCTTATGTATATCTTTCGTTTTCCCTAATTTCGTGTTTTAGGGTTGGATTAGGTTTTGCGTTTATGTTGTTCGATATCTAACGGATCAAAATCTCTCCTTCCTTAGCAAAGTTTGAAAACTCCCTCCACATTTTCATCTCCTTTTCCACATAACCAACTCAACACTTTTCAATTTCTATAACAATTTCGAGAAATCAAAACCAGAATTAATAAAATTAACCAGTTGATTCTTTCTATA

>AT5G10980_H3_A_thaliana ref|NC_003076.4|chr5:1-26992728:3473023-3474022 Arabidopsis thaliana chromosome 5, complete sequence

GTCGATCGGATTTTAGTTAAGAGTTGGGCTGGAATGTTTTCATGTTCTAATACCAAACCGAATTTTAATTATGATGAGGTTATAAACTTATAATTCAACCATGTCCATGTCATATATGTGATGGTTTTAACCCAACCAACTTGAAATCTCGGATCGGAGCGGCCGTACCAAATTTGGTTCGGATCGATATTCAGAACACAGCTGACGAACTCTCAATGCCTTATCAGTTGGGCCTGAACCCAACGCAGTACTCCTACCAAGGGATACGACTCGGCCCATCATCGCTAATTACCAAACATGTAGATCAAATCGTCATTACCTTTTTTCTAACCCAACTAAAATCGTGATTACTTCAAACGACAAACGTAGAAAAGAAACACAAAACTAGCCACGTCATCGATCCTCCCAAGATTAAACCAATCAGAAGCGAGGACGCAGATCGCCAACGTCAGTATTCGCTCTCATAAGTCGAATCTTGACCGTCGAAAATGTGAAGATTGGACCACTTCTGTATTTCCTAATATTAACCGACTAAAACATTCACCTATATAATCATAATCACCTTTTACTATTCCCACACAATCATCAAGAAAACAAAACTCGCTTCCTGATAATCAAATCAAAAGCTTCTCTATCAAGCTTATCGAAAAGTCACATCTTTTGGAAGAAACAATGGCTCGTACCAAGCAAACCGCTAGGAAATCCACCGGAGGTAAAGCTCCCAGGAAGCAGCTTGCCACCAAGGTTTGTCCTTCTATCTTTCATTCATACCGAAATAGTAGATCTAGGGTTCAAATTTGGGTTGAATAACGAGTTTTTGTGGACGAAATCTGAAAAATATGATCTAGGTTTTCTATATTGTGTTGAATTTTTAGTTTATGATCTAATTTAGTATGAATCTTGCGGTTTTGATTTTGTTTATGATGAAATTGGTTGGAAGGGGATCTAGGGTTTATCTAATTTGAGTATTCGATTGCGTCTCTAACGAATTCTTCAATAC

>AT5G12910_H3_A_thaliana ref|NC_003076.4|chr5:1-26992728:4076166-4077165 Arabidopsis thaliana chromosome 5, complete sequence

AACCAAAATAATTTTTTAAATATACGATTACTGAAAAAGATTTGTATAATCTCTACTTAAGAATCACCTGTTTCACTTCCATTGAGATCCAGCACTAGTATAGACCTATTATACTATGGGCTTTTACATCATTGTTCTATCTACCGGGCCATACATGCTATTAAAGATCTATTGAAGCATTATAGTTTCGAGGATAGGTTTTTTCAAAACCTAGATATATAGAAACTTTTTCTTTTGGACAAAAGATAGAAACTTTATTGGTAAGTATTTATGAATGCTTATTGGTGCAAACTGACGATGGGTTAGTTTTAGTAATTCTATCAAAGTAGTTTGCATTTTGATTGGTAGTTATAGTCTTACAGAGCCCGTCTTGAGGGCAAGCAAATAAAGCATGTGTTTGCCTCTGTAGTATAAGTAAGTTCTCGGTCCATTTTTCACAAAATATCCTTTAGGTAAGATAGTGTACATAATTGTATACGTGAAAGTACTCTAGTGTTGGAATCTCACATTTACCTCATTTCTTACTTTTGTCTTTTAGTCGATTTTTTTTTTTTTTTTTTAACGTCTGAATTTTTAATTAGTTGATTATTTCATAGGCCCAATATGTTTTATTTATTGTAGCCCATATATTTTCAAGGTCAGTTGACACAGTCGCAACGGAAAAAAAGATCTCAAGCGTTGATTTTGAGACTAATCGACGGTTGTTTTAGTTTTGAAAGGTGAAAGCAAATCAATGATCTATATTTATCGAGCGTTGGTGCTCAAACTTATAGACGGTTATCTTAGTTTTGAAAGATGACAGCAAACCTATGGTCTATATATAGTTGTTAAATCATTATTCATGCGGATAAGAAAATCTTAAAATTCCTTTTAATCATAGTTTCTTCCTAATTTTCATTTAGATTCTCCACAAATAAATAAATGTCTCCACCCACAAACTCTCAATACATATCTTTCACCATCAAAACTTTTTTACATATCAAAGTTGATTTGCTCACTA

>AT5G10980_H3_A_thaliana ref|NC_003076.4|chr5:1-26992728:3473350-3474349 Arabidopsis thaliana chromosome 5, complete sequence

TCTTATTTTAAAACACGTAAAACTTAAAACTGTATTTTGTGCATTCAAATTTAGGCTTCAATGGCCATAAGCAGAAGTGATAAACTTGAATAATGTTGGGTATTAAATATATATAGCTTTTTCCGTACAACTTTTGTATAGTACGTGAATTCGTTAACGTTCCACACTTACACTCCAATTCTTTTTAAAAGAAACCTTTTGTAAAGATCTATGATACATGTGGAGCAGGAAAACATATGTTCTGTTAAAAACTTGCATTTTCACGTTATAAAAGCCAATTAAGAATAGATTTAACTCAACACGATATCATGCATGAGCAACTTTTTGGTCGATCGGATTTTAGTTAAGAGTTGGGCTGGAATGTTTTCATGTTCTAATACCAAACCGAATTTTAATTATGATGAGGTTATAAACTTATAATTCAACCATGTCCATGTCATATATGTGATGGTTTTAACCCAACCAACTTGAAATCTCGGATCGGAGCGGCCGTACCAAATTTGGTTCGGATCGATATTCAGAACACAGCTGACGAACTCTCAATGCCTTATCAGTTGGGCCTGAACCCAACGCAGTACTCCTACCAAGGGATACGACTCGGCCCATCATCGCTAATTACCAAACATGTAGATCAAATCGTCATTACCTTTTTTCTAACCCAACTAAAATCGTGATTACTTCAAACGACAAACGTAGAAAAGAAACACAAAACTAGCCACGTCATCGATCCTCCCAAGATTAAACCAATCAGAAGCGAGGACGCAGATCGCCAACGTCAGTATTCGCTCTCATAAGTCGAATCTTGACCGTCGAAAATGTGAAGATTGGACCACTTCTGTATTTCCTAATATTAACCGACTAAAACATTCACCTATATAATCATAATCACCTTTTACTATTCCCACACAATCATCAAGAAAACAAAACTCGCTTCCTGATAATCAAATCAAAAGCTTCTCTATCAAGCTTATCGAAAAGTCACATCTTTTGGAAGAAACAA

>AT3G27360_H3_A_thaliana ref|NC_003074.4|chr3:1-23470805:10131106-10132105 Arabidopsis thaliana chromosome 3, complete sequence

TATTATCAATCATAGCTTAATCATAGAAAAGTATCAATCATAACTTCCTTGACATTTATGATTGACTGCGTAAGTATAACTGCAAGTACTGATCACACAACAAACAAGAAAGATGACACCGGCAACACTAGCTATATTCTTTGTCTCTTTGGTTACTATTGGAACAGCAACCGATGTTGCTCCAGCAACTAAACAACAGTTTGAGTTCTTTCCGAGCTTGCGGGAGCATCAGAAGTCTCTATTTGTTACTGTGGAAGGTACCATAGCTTCGAATACTAGTTCTAGAAAGTAAAAACAACGATAATGTTTTTTAACTGTGGAAGGTACCATTGCTTTTTGTTGTTGTTCTTCACGTGCAGGCTGCAACAACCATTGCCTAACAGCTTGCTGTGACTGCAACATAGAAAAACAGCCTCCCGTTTGTGTTCAGTGTTGCCCGATTTGAGCCTTGAACTCCTTATGTTCATGTATGTTTGAAGACTAGAGACAATGTAGAAGATTTAAAACACAATTCAGGATGTCAAACTTGTGAAACAGAGGAAGCTTATGTGATATTTTATAAGTAAAAAAAACAGATATCACAAATAGAGAGAGATCAGAGTTTGTATTGTAAAAGCCTACAAATTTTCGCAATCTGTTGATGGAATAAATTCATTGAACAGATCGTAATATCAAAGATGAAAACACTTCTGTCTCACTCAGAGTCTTTGGATCACAAACACCGTTACACATCGCAATAATTAACACGTCATCATAAATATCCCACGTGTAGAATCCTCGACAACTGAAAGAATCACTCTGATTGGTTACTTAACATACGACGCAGATAGCAAAGATAAAAACAATCTTGACCGTTTAGAGGAAAACACATCTAACGGACCAAATTCTCTCTTCTTTGAGTATAAAAAAGCATCCCAACCTCACTCTGTATATACCAATCAAACACGACAGTCTCAACAATCAAAAGCTTCAAATTTTTCATTGTCAAGAATTCTCAAAA

>AT1G09200_H3_A_thaliana ref|NC_003070.5|chr1:1-30432563:2972134-2973133 Arabidopsis thaliana chromosome 1, complete sequence

TGTCGGAGATAGAAACAAGTTTTAGCAAAACTAAAAGGGGGATACAATGTCTGTCAGATGCTCATTTTAGACTTTAAAAAACCATGAAGAAGTTTTGCAGTTGCTTCTCTTTCTCTACTCTTGTTTCTCTGCTAAGCCAATGTCCGCCAAAATCGGATTCTGGTGTATTCGTTCACTGCTCCCCACCCTTTGAGAATTTGTTGTTAGTCTCTTGTCAGTCAATACAGACCACCAAGTCCATAATCCAACAGTCCAACGTCCCTGCAAGTTGATTGATACTAGAGTAGTAGTAATGTAGAATGTCTTTCACTTTCCAAACAATTTCCACATTGTCTATACACTTATACTGGGTTCACTTCCATTGTGAAGACAGATGTTGAGATAACAAAAATTATATAGAGATAATTGTACCCCAACCACAGATGCTTAGAACGCTAGAGAACGAATGCAGTGCAATCTTTGTTTTACCTTAACCTTAGACAGCAATATTTTAATTCATGATGTCATTCTCAAAAGATGAAGTCACAATCTTTATACAAAACAGCGTCCTAGCATATTGAGATGGTATTTAAGCCCAGAAAAAGCTGATTGAGAACCAAAAAAAGAAAAACAATTACGCAGGCCCTATTAAGTAATGGGCTTGGTTTTTTGGTAAAGATAATATATTTAGATTATTTCTTTTATTTCTATTGGTCTTCGTACCTTCACGCGGATCAGTGTTAGAAAAGAATGAAATCTGTCCGTAGAATTGTATTAATCTACGGTTGCTGTGATTTCACGCGGATCGTGATAGAAACCTCTTATATCCGTTGATTAAAAACCAATGAACGATCCAGATCTAATCCCGCTGAAAAAGAAAAGCGAAATATTTTCCCTCCCACTTCTTGTAATTTCCAGACGATAAATATCCCCTTCCTCAATCGAAACAAACTATCCAGATCTCAACTTTCTCTCATCTTCAAATTAAAATCAAACAGTTTCTTAATAACATTTTACTTCA

>AT1G13370_H3_A_thaliana ref|NC_003070.5|chr1:1-30432563:4588519-4589518 Arabidopsis thaliana chromosome 1, complete sequence

TGATTTCTCAACCAATATTTTGATATTCAAATTTGACAATTTCAGGATGACATTTCTGGTAGCAGAAGCTTGTGTAATAGCAGGAGCGACCAAGAACGCATACCACACAAAGTACTTAAGTTCGCAAACCTTTTCGTGTGCATCATTGCGTAAAGGAATTTTCATTGCAGGTGCAGTCTTTATAGTTGCTACTATGGTTCTTAATGTCTACTACTACATGTATTTTACCAAGTCCGTCTCTTCGCCGCCTGCTCACAAAGCCAACCGCTCTAGTTCCAATATTGGTATGGCGGGTTATGCATAAATGTCAAAAAGAGGAAATGGTTAGTCATTTGCATTGTAGATGTAAGAAAGGAAAGAGATTGTTGCATTGTTACAACACTTGGTTACTCTTATATCTTTTGCTTATATATCTCTTACTTATTTATGAAAATTTTCGCTATTTGCTCTCAATTCAATAGGCAACTTTCGGTCATAAGTAATGGATGTTTGTATTGACTAGATTTGTCATAATCATTGTTATTTAACTACATATTTTAAAAGGTATGTGAACAATGCAAAATGTTCATCCTATAATGGGCTTCTGGGCCTATTTAAACTGGATTATAGATCCTCCCGTAGACTCAAATATAAAGCCTACTCATTTTAAGAACATTACTTCTTTTTTTCTGCAACAAAAACGTTACTTAAACCAAAGAAGAAGAAGATTCAACAAACTCAACAAGAAAATGGACCAATAAGAATCGAAAGCGCAGATCGCCACATATGCATAATCTTAATCAAAATCAAATATGGACCGTCGAACATGCCTTTAATCGCACGTCTATCAACTCCTCTCCCCAAAATTAACCGACCTAATTCTCACACCTCGTCACTATAAAATTCACTAATCTTTTTTACTTTTCTCTACATCAAAACACAACATTAAAAGCTTTGGCGATTTTTCTCTCTGATTCAATCTTTTCATAGTTTCTAAGCTCTCAGATTCTTGAAGAAGCCA

>AT1G75600_H3_A_thaliana ref|NC_003070.5|chr1:1-30432563:28393953-28394952 Arabidopsis thaliana chromosome 1, complete sequence

TTGGGAAGAACTTGCATATGTGTTTTTATGATGTGGCTGCTACAATGGGCTTCTGGGCCTGTTTTAACTGGACTGTAGAGTCCATTCATGATCAATAGAGGCCCGAATATAAGTCTACTCATTTTAAAACCATTACTTAAACCATAAGAAAACACTAACCTTAAAACACACAAGATTTGGACCAATAATAATCGAAAACGCAGATCGCCACGTATGCTAATCTTATCCTTAATCAAATATGGACCGTCGAAATGGCCTTTAATCGGACGTCTATAAACTACTCTCCCGTTAATCTCCCAAAATTAACCGACCTAATAATTCACCTCATCACTATAAATTCACTAATCTGCTTCACTTTTCTCAAAATCAAAACAACAACATTCAAAAGCTATTAGCGATTCTTCTTCTCTCTGATTCAATCTTCTTCATAGTTTCTAAGCTCTCAAGTTCTTGACGAAGCAATGGCTCGTACGAAGCAAACTGCAAGAAAATCACACGGAGGAAAAGCTCCGAGGACTCTGCTCGCTACCAAGGTTCGTTCGTTCGTTCTTAGTTTTGAATTTGTAACGTAGTTTTGAGAAATCTAGGGTTTCAGATTGATTGATTGTGTAATCGTAACATAGGCGGCGAGGAAATCTGCGCCGACTACTGGAGGAGTCAAGAAACCTCACCGTTACCGTCCCGGAACCGTCGCTCTTCGGTAAGTTTCACGATTTCTCCTCTTTCAAAATCACAATCTCTGTTTCTGAAATCATTCCGTCTTCGATGATGATGAAATCAGTTGGTTCCTGTTTGATGAATCAGTGAGATTCGTAAATACCAGAAGAGCACAGAGTTGTTGATCCGTAAACTTCCTTTTCAACGTCTTGTTCGTGAAATCGCTCAAGATTACAAGGTACAATTTTTGTCTGTCAGTTATCGTAAGAAATTGATTAGTTATTCCTAGATTGATGAAGAAGATTGGTCTTGATTATAATTGTTTCGTTTTTGGTGATGTAGA

>AT1G19890_H3_A_thaliana ref|NC_003070.5|chr1:1-30432563:6904542-6905541 Arabidopsis thaliana chromosome 1, complete sequence

ATAGAAAATGTTATATTCAAACTGAAAAAGACAAGTATGGAAAACAATAGAGAAATTAATAAACCAATATACTATAGAGTTGACCAGTTTTAATTCCTAATTAAGGCAAGAATATTACATTTTAGGCCTTTTATCATTTTACAAAATTCACCTGAGAAAATTTGATAAACCCTATGGATATATTAGGCCCACTGGCCCAGAGCGAGACAAAAACCCATTAGTAACCACGTCATCTATCCAACGCATTAGTCAACCAATTAAAATTGACTTTGAGGATCTCCACGTCAGCCTTCTATTTCAAAAATTCCAAAATTGAATACCAAAACCAAATAACCGTCTTTTGAATAATTAAAAAACCGCTATAACATGAGAGACACTATATAAGTGAAATCTCTAGTACATTGTACCCATTACTCTCGCAAACAAAAGAAACTGTCGCAAGTTATTGACGGTTATCATCATCATCATCGTTCTCTCGAAGAAATGGCACGTACGAAGCAAACTGCGAGGAAATCTACTGGTGGAAAAGGTCCAAGGAAGGAACTCGCTACCAAGGTTCTTTATATATCTTTGATTTTCTAACTTTTGTATCAGTGATATTAAGATTATGTTACGTTGTTGAATATTTTGAGAATGGGTTTCTAAGGTTTACGATTCTGTTTTTATCAGGCGGCGAGGAAAACTAGACGACCGTACCGTGGTGGAGTCAAGAGGGCTCACCGTTTCCGTCCTGGAACCGTCGCTCTTCGGTCAGATTCTCTTTTCTCTTTCTGTTTTTTTACCCGAATAATCTTTTCTCTCATGTTCTCAAAAATGGTTGCTGTCTTGTTGATGTGGAGGATAACAGTGAGATCCGCAAATACCAGAAGAGTACCGACTTGTTGATCCGCAAATTGCCTTTCCAACGACTTGTTCGTGAAATCGCCCAAGATTTTAAGGTTTATTCTAACTTCTCTCGATCAAGTTCGTTTACTAGTTTTAGTGATTTCGTGTGATATTT

>AT1G75600_H3_A_thaliana ref|NC_003070.5|chr1:1-30432563:28393415-28394414 Arabidopsis thaliana chromosome 1, complete sequence

AAGGCATAGTAAAGATGAAGGATCTAACTGTAATTACGGGTAGTTGTGGAATATAATGGCCGAATACTTAGAAAATATCATTTGGAATTAAACGTAACAATCAAGAAATGGTATAGCTAGTAGCTTTTATGTTGGTCATTAGCAGAATCACTAAACATAAAATTGTGATTGTGCACCATTGAGGTTTAGACCTTTATATGAACAATTGAACTAAACTTACCAAAGCGAAACCTTTATATGTCAATGATAGGGTCGTCACTATCAAGAAACGTTATTGGGAAGTAGGACTTATAGTTACATCAAATATCAAATACAATCATATTCTGTTTAAAATATAGCAACTCTAACCAATACTCCATACTATGCTTAGAATAAAACTTAACCACATTTTAAAATACCTAAACAATCTCATGATCATGATCATGAGAAGATCTTGAGTGTCCTTATATTTGTTTTATATGATATCATATCAAATATGTCTGCATCACGATATATGTTTGCTTTCTCTACGTTTCTAAGATTTATGTTTCTTGAATTCTTGGGAAGAACTTGCATATGTGTTTTTATGATGTGGCTGCTACAATGGGCTTCTGGGCCTGTTTTAACTGGACTGTAGAGTCCATTCATGATCAATAGAGGCCCGAATATAAGTCTACTCATTTTAAAACCATTACTTAAACCATAAGAAAACACTAACCTTAAAACACACAAGATTTGGACCAATAATAATCGAAAACGCAGATCGCCACGTATGCTAATCTTATCCTTAATCAAATATGGACCGTCGAAATGGCCTTTAATCGGACGTCTATAAACTACTCTCCCGTTAATCTCCCAAAATTAACCGACCTAATAATTCACCTCATCACTATAAATTCACTAATCTGCTTCACTTTTCTCAAAATCAAAACAACAACATTCAAAAGCTATTAGCGATTCTTCTTCTCTCTGATTCAATCTTCTTCATAGTTTCTAAGCTCTCAAGTTCTTGACGAAGCAA

>AT1G19890.1_H3_A_thaliana ref|NC_003070.5|chr1:1-30432563:6904026-6905025 Arabidopsis thaliana chromosome 1, complete sequence

TAGTACAGAGAAAAACTACGAAAGAAGTAAAAAAAAGAGAGAGAGATAAAGTGAGAGGAAAATATAAAAAGAGGGATTAATTGAAGAGATTTGGGGATTTTCTCTATTACCAAGAATATACATATTTCTTTTATTTTAATTTCTTCGGCTCTTCATTGGGTCTTTTGCTTTTTTTTTGTGTAACGTTTTGTCTCTACTTTTTTTGTCACTATCTTTTGTTTTGTGCACTCGTGGAAATTTGTTTTAGCTTAATGTGTTTTTTTCTTGTTGTTATTAAAATACAAAGTTTGTCTTCCTAAGAAAATGAATTAAAAGATATTTTTCTCTCCCTGTTTTCTTTTTTTCTTTTTTTTGCTAATGTGTTAAAGTGAAATTGTTTGTCAGTTTATTGTTTTACTATATGTTAGTGAATAGAAATAGTTTTTGATGTTTTTATACATGAAAAAATAATAAACATTGCATAAATAAATAAAATATTTTACTCATAATATAAATGCAAAAATTCAGGAGATTGAGATAGAAAATGTTATATTCAAACTGAAAAAGACAAGTATGGAAAACAATAGAGAAATTAATAAACCAATATACTATAGAGTTGACCAGTTTTAATTCCTAATTAAGGCAAGAATATTACATTTTAGGCCTTTTATCATTTTACAAAATTCACCTGAGAAAATTTGATAAACCCTATGGATATATTAGGCCCACTGGCCCAGAGCGAGACAAAAACCCATTAGTAACCACGTCATCTATCCAACGCATTAGTCAACCAATTAAAATTGACTTTGAGGATCTCCACGTCAGCCTTCTATTTCAAAAATTCCAAAATTGAATACCAAAACCAAATAACCGTCTTTTGAATAATTAAAAAACCGCTATAACATGAGAGACACTATATAAGTGAAATCTCTAGTACATTGTACCCATTACTCTCGCAAACAAAAGAAACTGTCGCAAGTTATTGACGGTTATCATCATCATCATCGTTCTCTCGAAGAAA

>AT1G19890.2_H3_A_thaliana ref|NC_003070.5|chr1:1-30432563:6904391-6905390 Arabidopsis thaliana chromosome 1, complete sequence

AAGTGAAATTGTTTGTCAGTTTATTGTTTTACTATATGTTAGTGAATAGAAATAGTTTTTGATGTTTTTATACATGAAAAAATAATAAACATTGCATAAATAAATAAAATATTTTACTCATAATATAAATGCAAAAATTCAGGAGATTGAGATAGAAAATGTTATATTCAAACTGAAAAAGACAAGTATGGAAAACAATAGAGAAATTAATAAACCAATATACTATAGAGTTGACCAGTTTTAATTCCTAATTAAGGCAAGAATATTACATTTTAGGCCTTTTATCATTTTACAAAATTCACCTGAGAAAATTTGATAAACCCTATGGATATATTAGGCCCACTGGCCCAGAGCGAGACAAAAACCCATTAGTAACCACGTCATCTATCCAACGCATTAGTCAACCAATTAAAATTGACTTTGAGGATCTCCACGTCAGCCTTCTATTTCAAAAATTCCAAAATTGAATACCAAAACCAAATAACCGTCTTTTGAATAATTAAAAAACCGCTATAACATGAGAGACACTATATAAGTGAAATCTCTAGTACATTGTACCCATTACTCTCGCAAACAAAAGAAACTGTCGCAAGTTATTGACGGTTATCATCATCATCATCGTTCTCTCGAAGAAATGGCACGTACGAAGCAAACTGCGAGGAAATCTACTGGTGGAAAAGGTCCAAGGAAGGAACTCGCTACCAAGGTTCTTTATATATCTTTGATTTTCTAACTTTTGTATCAGTGATATTAAGATTATGTTACGTTGTTGAATATTTTGAGAATGGGTTTCTAAGGTTTACGATTCTGTTTTTATCAGGCGGCGAGGAAAACTAGACGACCGTACCGTGGTGGAGTCAAGAGGGCTCACCGTTTCCGTCCTGGAACCGTCGCTCTTCGGTCAGATTCTCTTTTCTCTTTCTGTTTTTTTACCCGAATAATCTTTTCTCTCATGTTCTCAAAAATGGTTGCTGTCTTGTTGATGTGGAGGATAACAGTG

>AT1G75600_H3_A_thaliana ref|NC_003070.5|chr1:1-30432563:28393753-28394752 Arabidopsis thaliana chromosome 1, complete sequence

CAACTCTAACCAATACTCCATACTATGCTTAGAATAAAACTTAACCACATTTTAAAATACCTAAACAATCTCATGATCATGATCATGAGAAGATCTTGAGTGTCCTTATATTTGTTTTATATGATATCATATCAAATATGTCTGCATCACGATATATGTTTGCTTTCTCTACGTTTCTAAGATTTATGTTTCTTGAATTCTTGGGAAGAACTTGCATATGTGTTTTTATGATGTGGCTGCTACAATGGGCTTCTGGGCCTGTTTTAACTGGACTGTAGAGTCCATTCATGATCAATAGAGGCCCGAATATAAGTCTACTCATTTTAAAACCATTACTTAAACCATAAGAAAACACTAACCTTAAAACACACAAGATTTGGACCAATAATAATCGAAAACGCAGATCGCCACGTATGCTAATCTTATCCTTAATCAAATATGGACCGTCGAAATGGCCTTTAATCGGACGTCTATAAACTACTCTCCCGTTAATCTCCCAAAATTAACCGACCTAATAATTCACCTCATCACTATAAATTCACTAATCTGCTTCACTTTTCTCAAAATCAAAACAACAACATTCAAAAGCTATTAGCGATTCTTCTTCTCTCTGATTCAATCTTCTTCATAGTTTCTAAGCTCTCAAGTTCTTGACGAAGCAATGGCTCGTACGAAGCAAACTGCAAGAAAATCACACGGAGGAAAAGCTCCGAGGACTCTGCTCGCTACCAAGGTTCGTTCGTTCGTTCTTAGTTTTGAATTTGTAACGTAGTTTTGAGAAATCTAGGGTTTCAGATTGATTGATTGTGTAATCGTAACATAGGCGGCGAGGAAATCTGCGCCGACTACTGGAGGAGTCAAGAAACCTCACCGTTACCGTCCCGGAACCGTCGCTCTTCGGTAAGTTTCACGATTTCTCCTCTTTCAAAATCACAATCTCTGTTTCTGAAATCATTCCGTCTTCGATGATGATGAAATCAGTTGGTTCCTGTTTGATGAA

>AT1G01370_H3_A_thaliana ref|NC_003070.5|chr1:1-30432563:143447-144446 Arabidopsis thaliana chromosome 1, complete sequence

AGACCAGGGGTAAAACAGGAACTAAAGAAGGCTAACAATCGAGTCGAACCCTCTATGTGAAGCCACAGGTTTAGTGCAAATTGTAATAAGTTGTTCAGAGAGACTCTTGACTGAAACAAATTGTGAAGCAGATTCGATTTTAAAATCAAAATTTGAGTGTCGAGCGGGAAAGTAAAAGTTCCGCTCCAATCTTCTAATCTTTTCGTATCTAGCGGGAAATTTCTCAGCAGGTGACTTTCATAATCGCAGTTTTCGTCGATTCTCTTTTCCGATTTTACGATTCCTCTCTCTCTCTCATGGTGCGATTTCTCCAGCAGTAAAAATCAATGGCGAGAACCAAGCATCGCGTTACCAGGTCACAACCTCGGAATCAAACTGGTATCTTAAATCTGCTTTCTCTTTCAATTTTTACTTCTGATTTTACCCAGAATTTTAGGTTTTTTATTTCGATTTTGTTAACCCTAGATTTCGAATCTGAAATTTGTAGATGCCGCCGGTGCTTCATCTTCTCAGGCGGCAGGTCCAACTACGGTACGGCATCTTTTTCCGTCTTAGGGTTTCCAATGTTTCTTCCTTTTATCGTTATGATCAAATTTGTTTATCTATCGAAATTGAAGACCCCGACAAGGAGAGGCGGTGAAGGTGGAGATAATACTCAACAAAGTGAGTTTTTTATATTTGAAGTCTTTTTTTTCCCTCTTTTCATCTCTTTTGTTTGTGAAGTTATTCTTTTGTAACATCTGCAGCAAATCCTACAACTTCACCAGCTACTGGTACAAGGGTAAGATTTTTGTGACCATTGCTTATGAACTGCTTCAACTTTGATTTCGTTATTAAGCTGACAAAATTCTCGTTTTGGTTTGTCAAGAGAGGGGCTAAGAGATCCAGACAGGCTATGCCACGAGGTTTGTTTTAAAAAAAAAACCAATCTCTTGTGATATCCCTGAGAATACAGGACACTTAGTGTGTTTAAAACTAATCTTCGGTGTTGTCCTTGTAG

>AT4G40030_H3_A_thaliana ref|NC_003075.3|chr4:1-18585042:18556094-18557093 Arabidopsis thaliana chromosome 4, complete sequence

TGTGTTGAGAAAACACAATGTACAATAGTTTGAACCGTAGGATAAAGAATGTGAGAAAGCACAAAGGAGCGTATCGTGATAACCCTTTCGTGAGTATTATAAATAATAATTCAACTACATTGCGGCCACAACCACTACTTCAATCATCCCCTATTACTGCTTTTGTTACGAAAGACAAAAAAACAAAACAAAACAAAAGTCAGCTCAGGCGAAGAACAGGTATGATTTGTTTGTAATTAGATCAGGGGTTTAGGTCTTTCCATTACTTTTTAATGTTTTTTCTGTTACTGTCTCCGCGATCTGATTTTACGACAATAGAGTTTCGGGTTTTGTCCCATTCCAGTTTGAAAATAAAGGTCCGTCTTTTAAGTTTGCTGGATCGATAAACCTGTGAAGATTGAGTCTAGTCGATTTATTGGATGATCCATTCTTCATCGTTTTTTTCTTGCTTCGAAGTTCTGTATAACCAGATTTGTCTGTGTGCGATTGTCATTACCTAGCCGTGTATCGAGAACTAGGGTTTTCGAGTCAATTTTGCCCCTTTTGGTTATATCTGGTTCGATAACGATTCATCTGGATTAGGGTTTTAAGTGGTGACGTTTAGTATTCCAATTTCTTCAAAATTTAGTTATGGATAATGAAAATCCCCAATTGACTGTTCAATTTCTTGTTAAATGCGCAGATGGCTCGTACCAAGCAAACCGCTCGTAAGTCCACCGGAGGTAAAGCTCCAAGGAAGCAACTTGCTACTAAGGTTTTGTTCCTTCTTGTCTCTTTTTTCAAATAATACTTGTGTTGTGAAGTTGAATGTTAATCTCCTTCTTTATTAACCTCAGGCTGCTCGTAAATCTGCACCAACTACTGGTGGAGTCAAGAAACCACATCGTTACCGTCCTGGAACTGTTGCTCTCCGGTTTGTCCCTTCTTCGATTTGTATGTGATTCTTTGAGATTATGTAACATTGTGTGTTAACATTCCTCTTATCTTTTGGTGTTCAGTG

>AT4G40040.1_H3_A_thaliana ref|NC_003075.3|chr4:1-18585042:18558230-18559229 Arabidopsis thaliana chromosome 4, complete sequence

ATTTGAATCGCACAGATCGATCTCTTTGGAGATTCTATACCTAGAAAATGGAGACGATTTTCAAATCTCTGTAAAAATTCTGGTTTCTTCTTGACGGAAGAAGACGACGACTCCAATATTTCGGTTAGTACTGAACCGGAAAGTTTGACTGGTGCAACCAATTTAATGTACCGTACGTAACGCACCAATCGGATTTTGTATTCAATGGGCCTTATCTGTGAGCCCATTAATTGATGTGACGGCCTAAACTAAATCCGAACGGTTTATTTCAGCGATCCGCGACGGTTTGTATTCAGCCAATAGCAATCAATTATGTAGCAGTGGTGATCCTCGTCAAACCAGTAAAGCTAGATCTGGACCGTTGAATTGGTGCAAGAAAGCACATGTTGTGATATTTTTACCCGTACGATTAGAAAACTTGAGAAACACATTGATAATCGATAAAAACCGTCCGATCATATAAATCCGCTTTACCATCGTTGCCTATAAATTAATATCAATAGCCGTACACGCGTGAAGACTGACAATATTATCTTTTTCGAATTCGGAGCTCAAGTTTGAAATTCGGAGAAGCTAGAGAGTTTTCTGAGGTACGATTCTTCGATCCTCTTTGATTTTCCTGGAAATATTTTTTCGGTGATCGTGAAACTACTGGAATCGCTCGATAGGTGGTACGAAATTAGGCGAGATTAGTTTCTATTCTTGGCCATTATCTTGTTTCTTCGCCGAATGATCTTCCGGATAAAGATTTTAGGTTAGAGATGAATCGTATAGCTAGATTTCATCACCAGATAGTTTCTTTGTCTAGAATCTCTGAAATTCTCGATAGTTTTCACATGTGTAAATAGATTGTTCTTATTCGGCGATTGTTGATTAGGGTTTTGATTTTCTTGATTATGCGATTGCAATTAGGGATTTTCTTTGGTTTTGTGTTGATCTTACGATACATTCCGGCAATTGAATACGTATGGATCTAAATCTTGTTAATTTGTTGAACAGA

>AT4G40040.2_H3_A_thaliana ref|NC_003075.3|chr4:1-18585042:18557802-18558801 Arabidopsis thaliana chromosome 4, complete sequence

CATTGATAATCGATAAAAACCGTCCGATCATATAAATCCGCTTTACCATCGTTGCCTATAAATTAATATCAATAGCCGTACACGCGTGAAGACTGACAATATTATCTTTTTCGAATTCGGAGCTCAAGTTTGAAATTCGGAGAAGCTAGAGAGTTTTCTGAGGTACGATTCTTCGATCCTCTTTGATTTTCCTGGAAATATTTTTTCGGTGATCGTGAAACTACTGGAATCGCTCGATAGGTGGTACGAAATTAGGCGAGATTAGTTTCTATTCTTGGCCATTATCTTGTTTCTTCGCCGAATGATCTTCCGGATAAAGATTTTAGGTTAGAGATGAATCGTATAGCTAGATTTCATCACCAGATAGTTTCTTTGTCTAGAATCTCTGAAATTCTCGATAGTTTTCACATGTGTAAATAGATTGTTCTTATTCGGCGATTGTTGATTAGGGTTTTGATTTTCTTGATTATGCGATTGCAATTAGGGATTTTCTTTGGTTTTGTGTTGATCTTACGATACATTCCGGCAATTGAATACGTATGGATCTAAATCTTGTTAATTTGTTGAACAGATGGCTCGTACTAAGCAAACAGCTCGTAAGTCTACTGGAGGAAAGGCTCCTAGGAAGCAGCTTGCTACAAAGGTAAGACTCGGGCTCTCACATGTGATCTGAGTAGCTTGATAAACACATTTCTAGATTTGTTCTAATTGGTGGATGTTTTAATTTAAGGCTGCACGTAAGTCTGCACCAACCACTGGAGGAGTCAAGAAGCCCCATCGTTACCGTCCAGGAACTGTTGCACTACGGTATGCAATCTGTTTCTTCCCCAAATTCAATTGTGTTTTTACTTTATTGATATCGTCATTGATTAAACTCTGTTTCTCTGTTTTATTGATCTTGATTGACAGTGAAATTCGTAAGTACCAGAAGAGTACCGAGTTGCTGATCAGGAAGCTCCCTTTCCAGAGGCTAGTTCGTGAGATTGCCCAGGATTTCAAG

>AT4G40030.1_H3_A_thaliana ref|NC_003075.3|chr4:1-18585042:18556260-18557259 Arabidopsis thaliana chromosome 4, complete sequence

TTTCTGACTCTTGTTTCATCTGTATCTGTTCGATATTGTATGATTGAGTTTTATTGTTTATCAATAAGCTTTCTGTTTCAAAGTTCTTTTCAATATCAACAACTTTTACGTTCACGAAATTGGTAATCCGTGTGACACTTAAAATCTCTAATCGTGGCCGTTGGGCTGTGTTGAGAAAACACAATGTACAATAGTTTGAACCGTAGGATAAAGAATGTGAGAAAGCACAAAGGAGCGTATCGTGATAACCCTTTCGTGAGTATTATAAATAATAATTCAACTACATTGCGGCCACAACCACTACTTCAATCATCCCCTATTACTGCTTTTGTTACGAAAGACAAAAAAACAAAACAAAACAAAAGTCAGCTCAGGCGAAGAACAGGTATGATTTGTTTGTAATTAGATCAGGGGTTTAGGTCTTTCCATTACTTTTTAATGTTTTTTCTGTTACTGTCTCCGCGATCTGATTTTACGACAATAGAGTTTCGGGTTTTGTCCCATTCCAGTTTGAAAATAAAGGTCCGTCTTTTAAGTTTGCTGGATCGATAAACCTGTGAAGATTGAGTCTAGTCGATTTATTGGATGATCCATTCTTCATCGTTTTTTTCTTGCTTCGAAGTTCTGTATAACCAGATTTGTCTGTGTGCGATTGTCATTACCTAGCCGTGTATCGAGAACTAGGGTTTTCGAGTCAATTTTGCCCCTTTTGGTTATATCTGGTTCGATAACGATTCATCTGGATTAGGGTTTTAAGTGGTGACGTTTAGTATTCCAATTTCTTCAAAATTTAGTTATGGATAATGAAAATCCCCAATTGACTGTTCAATTTCTTGTTAAATGCGCAGATGGCTCGTACCAAGCAAACCGCTCGTAAGTCCACCGGAGGTAAAGCTCCAAGGAAGCAACTTGCTACTAAGGTTTTGTTCCTTCTTGTCTCTTTTTTCAAATAATACTTGTGTTGTGAAGTTGAATGTTAATCTCCTTCTTTATTAACCTC

>AT4G40030.2_H3_A_thaliana ref|NC_003075.3|chr4:1-18585042:18556411-18557410 Arabidopsis thaliana chromosome 4, complete sequence

TTTCTTTTCTTTCTGTTTCTTGTCTAGTCGGTTTATGATTTGCTTGTGTTCTTCTTAGCTACGTTTTGTGGTGTGTATTGCTTTCTTCTTTGGCAATGTTGAACACCTTTTGTTGTTGAATTCATCTCTGAAACATATGAATATGTTAAAGTTTCTGACTCTTGTTTCATCTGTATCTGTTCGATATTGTATGATTGAGTTTTATTGTTTATCAATAAGCTTTCTGTTTCAAAGTTCTTTTCAATATCAACAACTTTTACGTTCACGAAATTGGTAATCCGTGTGACACTTAAAATCTCTAATCGTGGCCGTTGGGCTGTGTTGAGAAAACACAATGTACAATAGTTTGAACCGTAGGATAAAGAATGTGAGAAAGCACAAAGGAGCGTATCGTGATAACCCTTTCGTGAGTATTATAAATAATAATTCAACTACATTGCGGCCACAACCACTACTTCAATCATCCCCTATTACTGCTTTTGTTACGAAAGACAAAAAAACAAAACAAAACAAAAGTCAGCTCAGGCGAAGAACAGGTATGATTTGTTTGTAATTAGATCAGGGGTTTAGGTCTTTCCATTACTTTTTAATGTTTTTTCTGTTACTGTCTCCGCGATCTGATTTTACGACAATAGAGTTTCGGGTTTTGTCCCATTCCAGTTTGAAAATAAAGGTCCGTCTTTTAAGTTTGCTGGATCGATAAACCTGTGAAGATTGAGTCTAGTCGATTTATTGGATGATCCATTCTTCATCGTTTTTTTCTTGCTTCGAAGTTCTGTATAACCAGATTTGTCTGTGTGCGATTGTCATTACCTAGCCGTGTATCGAGAACTAGGGTTTTCGAGTCAATTTTGCCCCTTTTGGTTATATCTGGTTCGATAACGATTCATCTGGATTAGGGTTTTAAGTGGTGACGTTTAGTATTCCAATTTCTTCAAAATTTAGTTATGGATAATGAAAATCCCCAATTGACTGTTCAATTTCTTGTTAAATGCGCAGA

>AT_H3_A_thaliana ref|NC_003071.3|chr2:1-19705359:10153892-10154891 Arabidopsis thaliana chromosome 2, complete sequence

GTTATTCATAACTACCGTTGCAGATAATGTTTAAAGACACAAAAAAAAAAAAAAAATGTTCATTGTTCATGTGTTGAAAACAGGAGGAGGGAAAAAAACATTATTTCGTATAACATTGTTACATTTCATAACATTATAATTCAACATGTAAGTTTAGATGATAGTTCTATTCATTTCACTATAAAATCATTATTGACTATTACATGCGTTTATTAACTCACAAAAAAACCAATATTTTCCAGAGTTTCTAAAAGTGACTATGTTTTCTATCTAGAATAAAACTGGTTATAAATAATCGTTATTCAAAAAAATGTTGTCGACGAGAAAATAAAATAAAAATAAATGTTGGTTTGGTTGCAATTGGAAGGTTGACTAAATAAATAAGAACTCAAGAAGATAAGAAAAGAACAAAATACAAACCAAGCAACTTTTATATCTTCAAGATAATTTCTCACAAATACATTAAATTGTGAATAGTAGTGATTCCAAATTATAAAACACACAGTATCATGATTTCAACTAGATCGTCTAACTAGCAAGCTGTAGGATAATCTCTCTTTTGACATCTATATCTTTAAACAAATCAACAAAATAAGGTTCAACAAGTTCTTGGAGAGCCTCAACAACGATGTTCTGTAATTTTATCTGTCATGATATCTCTTTCCCTACTATGTGGAATAAAATTTTTCTTCATTGACTTTGTGTTTTTATGATATTTTTGAACCTCTCTTAGTGCAATTGTTTCTGGCAAGTTTTTGTGTGACTTATCACTTATCTAATAAACTTTTTTTCCTTTCCAAAATTGACTGAGTGACGAAGGTAGGAAATAATCAATTTTAGAAAAGAAAAGAGTTAAAAAGATACGACAAATACATGCCTGCCGAGGACAGTGGCACTGAGAGAGATCAGAAAATACCATAAAAAGCACAAAACTAATAATAAGAAAATTTCAATCCGAGAAACTAGTGAAAAAATATCATGAAAATTTGCGATTTCAAAA

>AT5G59970_H4_A_thaliana ref|NC_003076.4|chr5:1-26992728:24163889-24164888 Arabidopsis thaliana chromosome 5, complete sequence

GAAAAAGAGAGAAATTCAAACCTCAGCTTTCTCACAGGCATAATCGAAAGCGTTCTGATTCATCGGCCTAACAGCAATAATATCATAACTCTTTAGAATCGGATTCCCAGAATTCAAACTCTGACACTGAGCATTACTCTCCACATGAACTGTGAGACGCGTGTACTGCCGAAACGGAGTAGTTCGCGGAACACCGAGTAAATCGCGATGGAATCCGACAGAAGAAGCTAACCGCGGAGCGACTTTGATTAGAGACCCAAGAGTGAGAAGAGGGATCGTACAAGAGTCTTTGTCAGACATTACGCCTTTGATCGAACGGTTATGTGCGATCCCAACATAGCCTAGCTCCATGGCTTTCGTGGCGAGCTTTAATCGTAAGGTTTTCCCGCCGGCGATTTCCTTACCACCTGATCGTGGCGGCTCATTGTACGGAATGCTAAGATCGAAGAATCCCATTAGTGAGTCGGAGAGAACAGTGGCGGAGAGATTTTCCGATTAGGGTTTAAGCATTAAATAACGGGTTTTTAGATACTTCACCGATTTTAGGAGAAGAAATATAAATACGACCAAAAAAAAAAAAAGAGAAGAAAAAGAAATAAATTTATTTTACTATGCAACCGACGACGTATGAGGCAGTTACCGAAACGCCTGCGTATTCGAAAATAAGAGAAGTTTGGTTTCGATCCATAACCGGCCCATTAAAAAGTAAGCCAGCCCATGCAAACTAGTAACTACAAAGCGAAGCTCTGAAGAACACTTATGTAGTTATCATGCGGATCACAATTAATAACCGTTGATTAAAATCAATCGACGGCTATCATGTCATATCAGTATTGACAAGGATTGAGCTTAACAGCCGTTAGATGGGTTATTAAATCGGATGGTTGAAAACAAAAGCCCTCTTATATATAAACAAACACGTCCAGTCAATATCTGAAAATCAAAAGCAATTCTTCTTCACACAGTAGCGAAAAAAAGAAATCTAATTCTCTCGAGAAAA

>AT5G59690_H4_A_thaliana ref|NC_003076.4|chr5:1-26992728:24067876-24068875 Arabidopsis thaliana chromosome 5, complete sequence

GACGACCAACCATGTCTCATGTTGTTATTGAACTAAAAGAGTGTCTCGTGTCTGAAAACTCGAGGAGAAATATGAGTCGAGGCATGGATACACTAAGTTCCCCTGAAGTGAGCATGATCTTTGATGCTGAGATGATTCCCAGAGCAAGATAGTTTGTGCTGCAAGTGACACAATTGTAATGAAACCACCACTCAACGAATTTACTTGTGGCTTTGACATGTCGTGTGCTCTGTTTGTATTTGTGAGTGCCGGTTGGTAATTATTTTTGTTAATGTGATTTTAAAACCTCTTATGTAAATAGTTACTTTATCTATTGAAGTGTGTTCTTGTGGTCTATAGTTTCTCAAAGGGAAATTAAAATGTTGACATCCCATTTACAATTGATAACTTGGTATACACAAACTTTGTAAATTTGGTGATATTTATGGTCGAAAGAAGGCAATACCCATTGTATGTTCCAATATCAATATCAATACGATAACTTGATAATACTAACATATGATTGTCATTGTTTTTCCAGTATCAATATACATTAAGCTACTACAAAATTAGTATAAATCACTATATTATAAATCTTTTTCGGTTGTAACTTGTAATTCGTGGGTTTTTAAAATAAAAGCATGTGAAAATTTTCAAATAATGTGATGGCGCAATTTTATTTTCCGAGTTCCAAAATATTGCCGCTTCATTACCCTAATTTGTGGCGCCACATGTAAAACAAAAGACGATTCTTAGTGGCTATCACTGCCATCACGCGGATCACTAATATGAACCGTCGATTAAAACAGATCGACGGTTTATACATCATTTTATTGTACACACGGATCGATATCTCAGCCGTTAGATTTAATATGCGATCTGATTGCTCAAAAAATAGACTCTCCGTCTTTGCCTATAAAAACAATTTCACATCTTTCTCACCCAAATCTACTCTTAACCGTTCTTCTTCTTCTACAGACATCAATTTCTCTCGAGATAAACTAAATCTTCGCTGAAAAAA

>AT3G53730_H4_A_thaliana ref|NC_003074.4|chr3:1-23470805:19923948-19924947 Arabidopsis thaliana chromosome 3, complete sequence

ACTTACACGCAGGTCTGGCGGTGGTGGTGCAACTGAGAAGAAGAAGTGAACGTCATCTTAAGTTTGTGAATCGCTCGTAAAGAGTTATGGTTTCTTGTTGCAAAATCGTTATTATGATTCCTAAGCTTCTCGAATTATGTTTTGTTAGAATATCGGACTTAAAGAGAGTTTTGTCTGAGACCAGTGACTCTGCCTTTAAACTATTTGCCTCTTTTGATCAATTATCTTTCTTTTGCCTCTGAATTTATTGGAATATTGAAAAATCAGGCGGCCTTATGAATATCAGTTACCACGGATGATCAATTGAATTGGTTACCAACTTCTTTCAATGAAAGCTATAGAGATCATCAATGGGTTAATATTGAAGTCCATGATATATGTTAACAATTTTCTCTTCCTCTTAAGTCTCTAAATTAACTCAAAATATCAAAATTGCTTGAAGACTAAACTTAAATCAACCATAACACATTCTCACTACAGATTTTCATTAACCTCAAAAGTTTTGATTCCAAGATCAATGTTCTTAATCCTCCCTATTTTCCCAAATAAACACACAACTAACCTAATTTCAAGGGAGAACAAAACTAGCATTCTCCTTAATCACATGTATATCTATCACATACAATGTTAACCAAACGAAGCAAATAATTTTGTGAAAGAAAACACAACTTTCCCAGAGCGCTCCTTTAACTTGATGAATCTTACCAATTGCGTATCAACGGCTCAGATGTCATCAGGCATAATGACGCGGATAGCAAACAGATCCAACGGTCCAAAATCATTAAATTATAAACACGCGGATAGATGATAACGACCGTCGATAAACTCAGATCCAACGACACACAACTTCCTAAAGTTTTGTAAAAAACACAAGCACTCCTCCTCTTCTCGTCGTCTCATCCTATATAACAAACTCAATTTTCATTTCAATCAATCAAAACCATTCGGCAAAATCAAATAATCTTTTTCTTAGTTCGAATTTAATTTCCCAAGAGAAAAA

>AT3G46320_H4_A_thaliana ref|NC_003074.4|chr3:1-23470805:17031339-17032338 Arabidopsis thaliana chromosome 3, complete sequence

GAAATGTAAATTATATGAATCACACTCCATTTTCTTGCCTTTACAGATATTACTTAACAAGTGAGTTATCCGAGAAGAGTGATGTCTACAGTTTCGGGATCTTGTTACTAGAGATTATCACAAACCAACGAGTGATTGATCAAACCCGTGAAAACCCTAACATAGCAGAATGGGTTACATTTGTGATCAAGAAAGGAGATACTAGTCAAATTGTGGATCCTAAACTTCACGGAAACTATGACACCCACTCTGTCTGGAGAGCTCTTGAAGTAGCAATGTCATGCGCAAATCCTTCTTCAGTCAAACGACCAAACATGTCTCAAGTAATCATCAATCTTAAAGAGTGTCTTGCATCTGAAAACACGAGGATAAGTAGGAATAATCAAAACATGGACTCTGGTCATAGCTCGGATCAGCTTAATGTGACTGTGACCTTTGATACTGATGTGAAGCCTAAGGCAAGATAGTATATACGTGGATCTTCTCTAATGTGACATAAACCAAACCTTTCGATGTATAACTTTATGTTATTAGTTAACCATTGTGAAATAATTTTGTTTTATTTACTTGTGTTAAATATGAATATTACCCGTTCGTAATATATATATATATATATATATATGTGAAATGTTATAACGATTTTTTTTGTTCTATGATGATTTAATTTCGTTAGTAACGGTGAAAAGAATTTGGCGCTATATTCCTCAATCCGTGGCGCTTATTTGAAATTTTCATAAACTTTCACTTACAGGCATCACGGGGATCGACAATATTAAGCGTCGATAAAATTAAATCAACGGCTAATAAGTAATCTCATTGTTCACCCGGATCGTTATCTGCAACCGTTAGATCTAAAAACACATCTGACGGTTCAAATCAATTAGATCCATCTTGTATATAAATGACATCTCCTCTTAACAAGATTCTCAAATCATCTCAATCTCAATTAAATCTTCAAATCGAAAAGTTTCCGAAGAAAAATTCAGGTTCCTTTAGAAAA

>AT3G45930_H4_A_thaliana ref|NC_003074.4|chr3:1-23470805:16893376-16894375 Arabidopsis thaliana chromosome 3, complete sequence

CAAGTGGTTAGAGAATTGTAGATTAAATATGAATCACACTCCATTTTCTTGCCTTTACAGATATTACTTAACAAGTGAGTTATCCGAGAAGAGTGATGTCTACAGTTTCGGGATCTTGTTACTAGAGATTATCACAAACCAACGAGTGATTGATCAAACCCGTGAAAACCCTAACATAGCAGAATGGGTTACATTTGTGATCAAGAAAGGAGATACTAGTCAAATTGTGGATCCTAAACTTCACGGAAACTATGACACCCACTCTGTCTGGAGAGCTCTTGAAGTAGCAATGTCATGCGCAAATCCTTCTTCAGTCAAACGACCAAACATGTCTCAAGTAATCATCAATCTTAAAGAGTGTCTTGCATCTGAAAACACGAGGATAAGTAGGAATAATCAAAACATGGACTCTGGTCATAGCTCGGATCAGCTTAATGTGACTGTGACCTTTGATACTGATGTGAAGCCTAAGGCAAGATAGTATATACGTGGATCTTCTCTAATGTGACATAAACCAAACCTTTCGATGTATAACTTTATGTTATTAGTTAACCATTGTGAAATAATTTTGTTTTATTTACTTGTGTTAAATATGAATATTACCCGTTCGTAATATATATATGTGAAATGTTATAACGAAATTTTTTGTTCTATGATGATTTAATTTCGTTAGTAACGGTGAAAAGAATTTGGCGCTATATTCCTCAATCCGTGGCGCTTATTTGAAAATTTCATAAACTTTCACTTACAGGCATCACGGGGATCGACAATATTAAGCGTCGATAAAATTAAATCAACGGCTAATAAGTAATCTCATTGTTCACCCGGATCGTTATCTGCAACCGTTAGATCTAAAAACACATCTGATGGTTCAAATCAATTAGATCCATCTTGTATATAAATGACATCTCCTCTTAACAAAATTCTCAAATCATCTCAATCTCAATTAAATCTTCAAATCGAAAAATTTCCGAAGAAAAATTCAAGTTCCTTTAGAAAA

>AT2G28740_H4_A_thaliana ref|NC_003071.3|chr2:1-19705359:12337031-12338030 Arabidopsis thaliana chromosome 2, complete sequence

ATAACAGAAATTTTGAGTTCATTCTTCAAGATTTAGATAAGTCTTCAACTTGTTGAGAATTCTATGACAAACACTCATGGTATTCACTTTGGAGATTAAATCAAGAACACACGTTTTACGAGTTTTTCGAGATCGATATGATCCTAGTACATTTCAGCAAGAACAACACCTTGTAATTTACCATCAGTGGAGTTTTTGAGTTATAGAAAAAAAGGAGAAAACAAAAAGACTAGATATACAAGAGCTTTGGAGCTCAAGGGCTTTATAACAATATGGTTTTCAGTTAGAATAATAATCGTCACCTCCTTACAACGACATTGCTTCATTCTAGCTAAAGCACACATCCTTACGTTGTCCTGTAAAAATGCTTCAAACCAGAACATTCCAACAAAAATAGAGTCTCGTCTCTTCTTACGGTTAGTACACAGTAATTGTTTCTATTAATTCAATGGAGTTATGTTCAACATATCCACTATACCACATTTGGATTCAAACCCTTATAGTAGAATATGTTTTTTTTTTTTTTCCAAGTGCATTCAACTTGTCCAAATAATAATAATGTAAAAAAACAATTTTTAAATTTTTTAAAATATATGGATAGTAAAAAAAGAGATCTGAGAATTTACCTTTTTTTATGGTAGCTGAAAAGTAAAAGTAAAATATACTCCATAGTTATACTAATTATTTTCGATATCCATTGTAATTTAGTACATATAACCGGTCCGGTTGACTCACTTGGTTCAAAAGGAAAAATCAAAAATCCACAATCCGTGACTAACATAATAACCCAATCTACAACCGTCGATTCATTAATAATCAAGGATCCATATATCTCATAAACCTTTCTCACGGATCGTTATCTTTGACCGTCCGATCAAACAATAAAACGAACGGTCACGATTCATTTTCTAACCCAAACAATCTTCTTTATAAATAAAAAATAAATTCTTCTCTACTCATCAGAAAACTCAAATCTTAAAACTTTCTGGAAAAACAAAAA

>AT1G07660_H4_A_thaliana ref|NC_003070.5|chr1:1-30432563:2368210-2369209 Arabidopsis thaliana chromosome 1, complete sequence

AATACAAACCTGTGGGATTCGTCAAGACGACGGACGTTGTGACCAAAAGCTCTAGAGTAGAATTCTACAGATTTAGCTACGTCCTTCACGTACACAACAGTGTAAGCAAACGCCGGTCTCATCATATTAGCTGCCATTTTTTCCCAGTCTTATGATTCTGATTTCGATCCGATGAAATCCTTGGAAATGCTCATCATCAAACCTCACATAGGATCTCTCTTTACGGTGAGCTCAACACGTGGTCGACAACATGCACCTTCGTGTGTAGGGTACTTGCTGCCACGTGAGCTTTTTGCTCCCTTTTTTGGGTCAATTTGGGCCTACTACTCACATATGGGCCTTTAGATTAGAAAAAAGTCTTGGGTTATCTGTTATGTTGGAGGCTTTTTTTAGTTGTTTAATGGAGTTTACACAGAGAATGACGAAAAGTCGAAAGCCATCTACTACCGTTGATCTTTAAGAACCAACGATCCATATTCTTCTTACAGATTTCACGCGGATCGCAATCTTGGACCGTTGAAAGAAGATGAATCGTTACGGCCGAAAATATATGTCCTGGGTAGTCACTTCCTTTATAAAATGTTAATCCCACCACTTTTTTTTTTTTTTTTTTTTTTTTTGTCAAAACACAACTTTCATTCATTAAGGCCTCAAAGCCTCAAGAGAGGAAAGTTGATACAAGCTACGATAATACAACAGAAAGAAAGATACAAGTTAATCCCACCACTTTGTACTCGAAGTAACATACTTTTTACAATCACATTTTTGAAGAACTTTCATTCCATAAATCTACAGGTAATTCATATATTTCTGCCTTGCTTAGGCGTAGATTTCAATTTAATGTTAGCTTAATTTCTTCAAAGTTTTTCTCGATTTGTCACATTTTCGAAGAGTTTAAATGTTTCGATTCTCAATTCAATTGATTGATCTGGAAATCTAGGGTTTCTGAATAGTTTCCTTCTGTTATTTTTCACAGATCTTTGTGTATCAAGTAAAGAGA

>AT1G07820_H4_A_thaliana ref|NC_003070.5|chr1:1-30432563:2421743-2422742 Arabidopsis thaliana chromosome 1, complete sequence

TGAGTGGCAGAAAACAGAGAGAAGCTACCATAGACAACAGGTTTATCTTCGTCTTGGCTTCTGTCGAACTCAAAGAACTCTTCGAGTGGATTTGTTGGCGTCCGAATCGTCGAGGCAGCTGCTGCTGCTGTAGTGGACTCCGATCGCGCCGATGCAGCCGCCAAGAATCTCCTCCCGACAAATCGCGTAAGAAACATTTTAACAACAAACAATTCCTTTGAGCTTGCAGTTATATAATCTGATCATCAACAACAATCAGAAAGAGAAATCGAGATTGGATAACAAAATGGTAAATGGTTAATGAAAGTCGTACAAAATATTTCAGGCTTCCGAAGAAGTGAGAGATCGTGTACACAGAAAAGCACATCTCGCAGAGGAATAAATAAGAACTCACCGGAGATAGAGAGAGGAGAGCTATTTCACGGCGACGGTGACGGCCACAGAGAATGGAAAACCCTCTCTCAACGTTTCTCCGAGAATAACAGGTTTACGGAGACGGAAACTTTTAGGGCCCAATACTAGCTCTGTTAGTATTGATACTATTATTGGGCCAGGTTTCATTTTCTCTTTTAAGGCCTAATAAGACCCAACACTTGCAAATTATTGGAAGACGAATGCTGTTTTTGTTTTGTTTTTCAATAAAACAACATCTTTCAATTAAATTGGGAGCAATCCACGTCATCGATCCGTCAAGTATTGTTAACGATGTATAACCATTGGATCAGTAATCAACGGTTGATATGAAAAATCACACGGATCAACGCAGTTTTATATATTAAGACATTAGTTAGCCACTTCATATCCGTCAAATTCAATTGATCCTCTCTCCAAATCATCTTAAAAGTATCTTCTTCTTCTCCATCTGGTTTATTGTTCGATCGTCAATTCAATTGATTGATTTGGAAATCTAGCGTTTCTGTTCGTTACCATTCACTGAATAATTTTTCTTCTGTTCTTCACAGTCACAGATCTTTGGGTATCAAGTAAAACTGTGAAGTGA

>His4_CG31611_D_melanogaster dm2_dna range=chr2L:21412566-21412843 NM_165383 NM_165384 >dm2_dna range=chr2L:21412566-21412843 NM_165383 NM_165384

ACACGCACAGCACGAAAGTCACTAAAGAACTAATTTCAACGTTTCTGTGTGCCCCTATTTATAGGTAAAACGACAAAAACCCGAGAGAGTACGAACGATATGTTCGTTCGCTTTTCGCTCGTCAAATGAAATGGCCTCCGTTTTTCTCTCTCTCTCTCTCTCTCTCTCTTTCACCGTCCACGATTGCTATATAAGTAGGTAGCAAATGCTCTGATCGTTTATTGTGTTTTCAAACGTGAAGTAGTGAACGTGAACTTTAGTGAAACCCAAATCGGAGA

>His4_CG33909_D_melanogaster dm2_dna range=chr2L:21407504-21407779 NM_165383 NM_165384 >dm2_dna range=chr2L:21407504-21407779 NM_165383 NM_165384 >dm2_dna range=chr2L:21441763-21442038 NM_165383 NM_165384

ACACGCACAGCACGAAAGTCACTAAAGAACTAATTTCAACGTTTCTGTGTGCCCCTATTTATAGGTAAAACGACAAAAACCCGAGAGAGTACGAACGATATGTTCGTTCGCTTTTCGCTCGTCAAATGAAATGGCCTCCGTTTTTCTCTCTCTCTCTCTCTCTCTCTTTCACCGTCCACGATTGCTATATAAGTAGGTAGCAAATGCTCTGATCGTTTATTGTGTTTTCAAACGTGAAGTAGTGAACGTGAACTTTAGTGAAACCCAAATCGGAGA

>His3_CG33803_D_melanogaster dm2_dna range=chr2L:21472058-21472331 NM_165383 NM_165384 >dm2_dna range=chr2L:21472058-21472331 NM_165383 NM_165384 >dm2_dna range=chr2L:21487297-21487570 NM_165383 NM_165384

ACACGCACAGCACGAAAGTCACTAAAGAACTAATTTCAACGTTTCTGTGTGCCCCTATTTATAGGTAAAACGACAAAAACCCGAGAGAGTACGAACGATATGTTCGTTCGCTTTTCGCTCGTCAAATGAAATGGCCTCTGTTTTTCTCTCTCTCTCTCTCTCTCTTTCACCGTCCACGATTGCTATATAAGTAGGTAGCAAATGCTCTGATCGTTTATTGTGTTTTCAAACGTGAAGTAGTGAACGTGAACTTTAGTGAAACCCAAATCGGAGA

>His4_CG33901_D_melanogaster dm2_dna range=chr2L:21477207-21477480 NM_165383 NM_165384 >dm2_dna range=chr2L:21477207-21477480 NM_165383 NM_165384 >dm2_dna range=chr2L:21482252-21482525 NM_165383 NM_165384

ACACGCACAGCACGAAAGTCACTAAAGAACTAATTTCAACGTTTCTGTGTGCCCCTATTTATAGGTAAAACGACAAAAACCCGAGAGAGTACGAACGATATGTTCGTTCGCTTTTCGCTCGTCAAATGAAATGGCCTCTGTTTCTCTCTCTCTCTCTCTCTCTCTTTCACCCTCCACGATTGCTATATAAGTAGGTAGCAAATGCTCTGATCGTTTATTGTGTTTTCAAACGTGAAGTAGTGAACGTGAACTTTAGTGAAACCCAAATCGGAGA

>His2B_CG17949_D_melanogaster dm2_dna range=chr2L:21411198-21411330 NM_165381 NM_165382 >dm2_dna range=chr2L:21411198-21411330 NM_165381 NM_165382

GTAATGTGGGCCCGAACGCGTTCACGTTTATACTTTTTTTCGAGCAGTCAATTCAGGTCTAAGTCACCCACCCCTAACTGAATGCGCAGGCAAACGGAAAAGTATAAATATTTCGCTGTCTGGGTTAGGCGAG

>His2B_CG33890_D_melanogaster dm2_dna range=chr2L:21465603-21465828 5'pad=0 3'pad=0 revComp=FALSE strand=? repeatMasking=none

TGTTCACGTTACTTATATTTTCACAAACACAATTCACTTATCGTAATGTGGGCCCGAACGCGTTCACGTTTATACTTTTTTTCGAGCAGTCAATTCAGGTCTAAGTCACCCACCCCTAACTGAATGCGCAGGCAAACGGAAAAGTATAAATATTTCGCTGTCTGGGTTAGGCGAGCATTCGTGTTCCGTGCGTAAAGTGAACTAAGTGAAATAAACGCAAAGCAAA

>His4_CG33899_D_melanogaster dm2_dna range=chr2L:21446802-21447097 H4.D_melanogaster.copy1 H3.D_melanogaster.copy7 >dm2_dna range=chr2L:21446802-21447097 H4.D_melanogaster.copy1 H3.D_melanogaster.copy7 >dm2_dna range=chr2L:21456905-21457200 H4.D_melanogaster.copy3 H3.D_melanogaster.copy8

TTTTTCACTGTTCTATACTATTATACACGCACAGCACGAAAGTCACTAAAGAACTAATTTCAACGTTTCTGTGTGCCCCTATTTATAGGTAAAACGACAAAAACCCGAGAGAGTACGAACGATATGTTCGTTCGCTTTTCGCTCGTCAAATGAAATGGCCTCTGTTTTTCTCTCTCTCTCTCTCTCTTTCACCGTCCAGGATTGCTATATAAGTAGGTAGCAAATGCTCTGATCGTTTATTGTGTTTTCAAACGTGAAGTAGTGAACGTGAACTTTAGTGAAACCCAAATCGGAGA

>His4_CG33887_D_melanogaster dm2_dna range=chr2L:21517146-21517445 H4.D_melanogaster.copy7 H3.D_melanogaster.copy12 >dm2_dna range=chr2L:21517146-21517445 H4.D_melanogaster.copy7 H3.D_melanogaster.copy12

TTTTTCACTGTTCTATACTATTATACACGCACAGCACGAAAGTCACTAAAGAACTAATTTCAACGTTTCTGTGTGCCCCTATTTATAGGTAAAACGACAAAAACCCGAGAGAGTACGAACGATATGTTCGTTCGCTTTTCGCTCGTCAAATGAAATGGCCTCTGTTTCTCTCTCTCTCTCTCTCTCTCTCTTTCACCCTCCACGATTGCTATATAAGTAGGTAGCAAATGCTCTGATCGTTTATTGTGTTTTCAAACGTGAAGTAGTGAACGTGAACTTTAGTGAAACCCAAATCGGAGA

>His4_CG33897_D_melanogaster dm2_dna range=chr2L:21451862-21452156 H4.D_melanogaster.copy2 H3.D_melanogaster.copy13 >dm2_dna range=chr2L:21451862-21452156 H4.D_melanogaster.copy2 H3.D_melanogaster.copy13

TTTTTCACTGTTCTATACTATTATACACGCACAGCACGAAAGTCACTAAAGAACTAATTTCAACGTTTCTGTGTGCCCCTATTTATAGGTAAAACGACAAAAACCCGAGAGAGTACGAACGATATGTTCGTTCGCTTTTCGCTCGTCAAATGAAATGGCCTCTGTTTTTCTCTCTCTCTCTCTCTCTTTCACCGTCCAGGATTGCTATATAAGTAGGTAGCAAATGCTCTGATCGTTTATTGTGTTTTCAAACGTGAAGTAGTGAACGTGAACTTTAGTGAAACCCAAATCGGAG

>His4_CG33885_D_melanogaster dm2_dna range=chr2L:21512303-21512601 H4.D_melanogaster.copy6 H3.D_melanogaster.copy14 >dm2_dna range=chr2L:21512303-21512601 H4.D_melanogaster.copy6 H3.D_melanogaster.copy14

TTTTTCACTGTTCTATACTATTATACACGCACAGCACGAAAGTCACTAAAGAACTAATTTCAACGTTTCTGTGTGCCCCTATTTATAGGTAAAACGACAAAAACCCGAGAGAGTACGAACGATATGTTCGTTCGCTTTTCGCTCGTCAAATGAAATGGCCTCTGTTTCTCTCTCTCTCTCTCTCTCTCTCTTTCACCCTCCACGATTGCTATATAAGTAGGTAGCAAATGCTCTGATCGTTTATTGTGTTTTCAAACGTGAAGTAGTGAACGTGAACTTTAGTGAAACCCAAATCGGAG

>His2B_CG33910_D_melanogaster dm2_dna range=chr2L:21406253-21406450

TTGTTCACGTTACTTATATTTTCACAAACACAATTCACTTATCGTAATGTGGGCCCGAACGCGTTCACGTTTATACTTTTTTTCGAGCAGTCAATTCAGGTCTAAGTCACCCACCCCTAACTGAATGCGCAGGCAAACGGAAAAGTATAAATATTTCGCTGTCTGGGTTAGGCGAGCATTCGTGTTCCGTGCGTAAAG

>His2B_CG33888_D_melanogaster dm2_dna range=chr2L:21490939-21491166

TTGTTCACGTTACTTATATTTTCACAAACACAATTCACTTATCGTAATGTGGGCCCGAACGCGTTCACGTTTATACTTTTTTTCGAGCAGTCAATTCAGGTCTAAGTCACCCACCCCTAACTGAATGCGCGGGCAAACGGAAAAGTATAAATATTTCGCTGTCGGGGTTAGGCGAGCATTCGTGTTCCGTGTGTAAAGTGAACTAAGTGAAATAAACGCAAAGCAAAA

>His2B_CG33876_D_melanogaster dm2_dna range=chr2L:21506073-21506300

TTGTTCACGTTACTTATATTTTCACAAACACAATTCACTTATTGTAATGTGGGCCCGAACGCGTTCACATTTATACTTTTTTTCGAGCAGTCAATTCAGGTCTAAGTCCCCCACCCCTAACTGAATGCGCAGGCAAACGGAAAAGTATAAATATTTCGCTGTCGGGGTTAGGCGAGCATTCGTGTTCCGTGTGTAAAGTGAACTAAGTGAAATAAACGCAAAGCAAAA

>H3.1_D_simulans droSim1_dna range=chrU:13085550-13085831 CG31613-RA CG31611-RA >droSim1_dna range=chrU:13085550-13085831 CG31613-RA CG31611-RA

TTTGGGTTTCACAAAATTTCACGTTCACTACTTGACGTTTTAAAACACAATAAACGATCAGAGCATTTGCTACCTACTTATATAGCAATCGCGGATGGTGAAAGAGAGAGAGAAAAACAGAGGCCATTTCATTTGACGACCGAAGAACGAAACGAACATATCGTTCGTACTCTCTCGGGTTTTTGTCGTTTTACCTATAAATAGGGGCACGCCGAAACGTTGAATTTAGTTCTTTAGTGACTTTCGTGCTGTGTGTGTATAATAGTATAAAACAGTGAAAAA

>H4.1_D_simulans droSim1_dna range=chrU:12262772-12263039 CG31613-RA CG31611-RA >droSim1_dna range=chrU:12262772-12263039 CG31613-RA CG31611-RA

TTTGGGTTTCACAAAATTTCACGTTCACTACTTCACGTTTAAAAACACAATAAACGATCAGAGCATTTGCTACCTACTTATATAGCAATCGCGGATGGTGAAAGAGAGAGAGAAAAACAGAGGCCATTTCATTTGACGACCGAAGAACGAAACGAACATATCGTTCGTACTCTCTCGGGTTTTTGTCGTTTTACCTATAAATAGGTTGAATTTAGTTCTTTAGTGACTTTCGTGCTGTGTGTGTATAATAGTATAAAACAGTGAAAAA

>H2B.1_D_simulans droSim1_dna range=chrU:3636871-3637098 CG31618-RA CG17949-RA >droSim1_dna range=chrU:3636871-3637098 CG31618-RA CG17949-RA

TTTTGCTTTGCGTTTATTTCACTTAGTTCACTTTACACACGGAACACGAATGCTCGTCGAACCCCGACAGCGGAATATTTATACTTTTCCGTTTGCCGGCGCGTTCAGTTAGGGGTGGGTGACTTAGACCTGAATTGACTGCTCGAAAAAAAGTATAAAGGTGAACGCGTTCGGGCCCACATCACGATAAGTGAATTGTGTTTGTGAAAATATAAGTAACGTGAACAA

>H2B.2_D_simulans droSim1_dna range=chrU:554407-554634 CG17949-RA CG31618-RA >droSim1_dna range=chrU:554407-554634 CG17949-RA CG31618-RA

TTGTTCACGTTACTTATATTTTCACAAACACAATTCACTTATCGTGATGTGGGCCCGAACGCGTTCACCTTTATACTTTTTTTCGAGCAGTCAATTCAGGTCTAAGTCACCCACCCCTAACTGAACGCGCCGGCAAACGGAAAAGTATAAATATTCCGCTGTCGGGGTTCGACGAGCATTCGTGTTCCGTGTGTAAAGTGAACTAAGTGAAATAAACGCAAAGCAAAA

>H4.1_D_simulans droSim1_dna range=chrU:13274632-13274890 CG31613-RA U.D_simulans.copy1 >droSim1_dna range=chrU:13274632-13274890 CG31613-RA U.D_simulans.copy1

ATTTGGGTTTCACAAAATTTCACGTTCACTACTTGACGTTTTAAAACACAATAAACGATCAGAGCATTTGCTACCTACTTATATAGCAATCGCGGATGGTGAAAGAGAGAGAGAAAAACAGAGGCCATTTCATTTGACGACCGAAGAACGAAACGAACATATCGTTCGTACTCTCTCGGGTTTTTGTCGTTTTACCTATAAATAGGGGCACGCCGAAACGTTGAATTTAGTTCTTTAGTGACTTTCGTGCTGTGTGTGT

>H4.1_D_yakuba droYak1_dna range=chr2R:1272947-1273237 CG31613-RA CG31611-RA >droYak1_dna range=chr2R:1272947-1273237 CG31613-RA CG31611-RA >droYak1_dna range=chrU:44961988-44962278 CG31611-RA CG31613-RA

TTTCCGATTTGAGTTTCACCACAGTTCACGTTCACTACTTCACGTTTCAAAACACAATAAACGATCAGAGCATTTGCTACCTACTTATATAGCAAGCGTGGATGGTGAGAAAGAGAAAGAGAAACAGAGGCCATCTCATTGACGAGCGAGGAACGAAACGAACATATCATTCGTACTCTCTCGGGTTTTTGGCGTTTTACGTATAAATAGAGGCACACAGAAAATGTTGAAATTAGTTCTTCAGTGACTTTCGGACTGTGTGTGTGCAGAATAGTATAAAACAGTGAATAA

>H2B.1_D_yakuba droYak1_dna range=chrU:33422246-33422469 CG31618-RA CG17949-RA >droYak1_dna range=chrU:33422246-33422469 CG31618-RA CG17949-RA

TTTTGCTTTGCGTTATTTCACTTAGTTCACTTTACACACGGAACACGAATGCTGGCCAACCCCGGCAGGGAAATATTATACTTTTTCGTCTGCCAGCGCTTTCAGTTAGGGTGGGTGACTTAGACCTGAAATGATTGTTCGTAAAAAAGTATAAAAATGAACGCGTTCGGAGCCACATTATGATAAGTGAATTGTGTTTGTGAAAATATAAGTAAAGTGAATAA

>H2B.2_D_yakuba droYak1_dna range=chr2R:1277452-1277679 CG31618-RA CG17949-RA >droYak1_dna range=chr2R:1277452-1277679 CG31618-RA CG17949-RA

TTTTGCTTTGCGTTTATTTCACTTAGTTCACTTTACACACGGAACACGAATGCTGGCCGAACCCCGGCAGGGAAATATTTATACTTTTTCGTCTGCCAGCGCTTTCAGTTAGGGGTGGGTGACTTAGACCTGAAATGATTGTTCGTAAAAAAGTATAAAAATGAACGCGTTCAGAGCCACATTATGATAAGTGAATTGTGTTTGTGAAAATATAAGTAAAGTGAATAA

>H2A.1_D_yakuba droYak1_dna range=chr2R:1275077-1275554 U.D_yakuba.copy5 U.D_yakuba.copy6 >droYak1_dna range=chr2R:1275077-1275554 U.D_yakuba.copy5 U.D_yakuba.copy6

TCTGAAGAGGCAAGGCCGCACTCTCTACGGATTTGGCGGTTAAAAAAAAAGTCCTGTACTTTTATTAAGCAATCGGTCCTTTTCAGGACCACCACTCACTTTTTAAAAGGAGGTACATTTTCAAAAAATATTTCCTTTTATTGGTTGGGAATCCCAAGAATGAGGTAGTCCGCTATTGCATCAAACTAAATGTCGATCATTGATTGCTTCTTCAGGTAGACCAAATTGAGAGTATAGTGTGTATATATTGATAGATTCATAACATGGCTTTGTACAGACTGTACTGAATTCAGACACCGTGTGTATATCAACGCATCGTGGAGTGCGAAAAATAAAAAAACTACTTTATATTTTATATAATAGAAAATTTGAAAAATCTTACTGCATATATAAATAAAATATGACAATTCCTAAAATAATTTTTTGTATAATTTATTCAATACAACATAATAGTAACGTAGTTAAAAAATTTTTCAAA

>H2A.1_D_ananassae droAna1_dna range=2448756:7696-7969 U.D_ananassae.copy1 CG31613-RA >droAna1_dna range=2448756:7696-7969 U.D_ananassae.copy1 CG31613-RA

TTTTTCAATCTTTTTCTTTTTATATTAACGAAAATCACTGAAGAACTAATTTTGTCAGATCGGTGCGCTGCTCTTTATACCGATTCAATGAGAATACGAAGAGGTCCGAGCGATATATTTTTCTCGCTCTTCCGAAACCCGATTTATGGCATCTCGTTCTCTCCCCCTCTTTTCAACCCTCGGCGTTTCTGGTATATAAAGAGGTAGCGCAACGGGCAGCACGTTTATTGTGTTATTAGAGGCGCGAAGAGAACAGTGAATTTGGAAATTAAAA

>H2A.2_D_ananassae droAna1_dna range=2448756:7719-7969 U.D_ananassae.copy2 CG31613-RA >droAna1_dna range=2448756:7719-7969 U.D_ananassae.copy2 CG31613-RA

ATTAACGAAAATCACTGAAGAACTAATTTTGTCAGATCGGTGCGCTGCTCTTTATACCGATTCAATGAGAATACGAAGAGGTCCGAGCGATATATTTTTCTCGCTCTTCCGAAACCCGATTTATGGCATCTCGTTCTCTCCCCCTCTTTTCAACCCTCGGCGTTTCTGGTATATAAAGAGGTAGCGCAACGGGCAGCACGTTTATTGTGTTATTAGAGGCGCGAAGAGAACAGTGAATTTGGAAATTAAAA

>H2A.3_D_ananassae droAna1_dna range=2447780:997-1223 CG17949-RA CG31618-RA >droAna1_dna range=2447780:997-1223 CG17949-RA CG31618-RA >droMoj1_dna range=contig_9922:2029-2255 CG17949-RA CG31618-RA

TAGTTACTTTCAAATTCACTTCACTTCACAATAATATTTGCGCCCAACAGCCGTTCGGCTTAGTACTTTTATTCGAGCAAGGTTTTCAGGTCTACTTTTAAAAAATTTCATCAGAAACGACCTACCCCCAACTGAAAGCAGGGATAAAGGCATAAGTATAAATTGGTGGTCCATAGTTCGCCACCATTATTCGTGTGTGAAGTGAATTATCGTAAAGTAAAAAAAAA

>H2B.1_D_ananassae droAna1_dna range=2452363:514-747 CG17949-RA CG31618-RA >droAna1_dna range=2452363:514-747 CG17949-RA CG31618-RA

GGCGGCATAGTTACTTTCAAATTCACTTCACTTCACAATAATATTTGCGCCCAACAGCCGTTCGGCTTAGTACTTTTATTCGAGCAAGGTTTTCAGGTCTACTTTTAAAAAATTTCATCAGAAACGACCTACCCCCAACTGAAAGCAGGGATAAAGGCATAAGTATAAATTGGTGGTCCATAGTTCGCCACCATTATTCGTGTGTGAAGTGAATTATCGTAAAGTAAAAAAAAA

>H2B.1_D_pseudoobscura dp3_dna range=chrXL_group1e:10045565-10045715 U.D_pseudoobscura.copy1 CG17949-RA >dp3_dna range=chrXL_group1e:10045565-10045715 U.D_pseudoobscura.copy1 CG17949-RA

TGTACTGTACATTTGTACAAGACCCAATTCAAGATAAATAAATTTATTAGTATGCTAAATTCTATTTATTTATGACAAACATTTTGGGCCCTGCCAAGAGCTTAATTACTCGCACATTCGTATACATATTGCTGCAGGGAAGAGCATTTAC

>H2B.2_D_pseudoobscura dp3_dna range=chrXL_group1e:10046014-10046536 U.D_pseudoobscura.copy2 U.D_pseudoobscura.copy3 >dp3_dna range=chrXL_group1e:10046014-10046536 U.D_pseudoobscura.copy2 U.D_pseudoobscura.copy3

TGTCGGTAGTGTTGATGTGGATGTTCTTCTGTGCCTTTCCAGCCTTCTTGGTTGCGTTCCCGCTTGCAGTTTTCGGTGGCATTCTGAAGTTTCACGATTTCGAAATTCACTTAATACTCAACACAACGCAACGCTTGCCGTGCGGGGCGCTCAGCATTATACTTCTTTGCGAGCAGTCTATTCAGGTTTAATGCAAACTAAACTACCAAGTAAATGAGTCGGAAGATGCTCTCCAGCTGGCTGTGTGATAGCCTGCTACCTGGTACTTTGCATTGCAGACTGGCTACGATTCACTTGCACCCGAAGACACCGAAATGCAGCCGAGTGAGGGTCAGTCTTCTATTTATAGCACACACCTGAAGACACGAGTGATGCCGAGGAGTGCGCCTATGTCCGACTCGCTCGCATACTCGTGTGCAATCGAAGCTCCACAAACTGCACATATAATGGATCAGCAATTCCTCCGACCACTTATTCTGCGCTTAGACGCTACGACGTGCATTGTGGTACATGAAACTGAAAA

>H3.1_D_virilis droVir1_dna range=scaffold_2274:1255-1554 CG31611-RA CG31613-RA >droVir1_dna range=scaffold_2274:1255-1554 CG31611-RA CG31613-RA

TTTTTCACTTTCTTTTTTTTACTTCACTTTACACCACGAATGTCACAGAGATACTAATGCTAGCTCTTCGGGCAGCGCTTATATTTATACCAAAAAATCAAAAAGACGAGCGAGTAAAAACATATTTCCATCTCGCTCACATACTACCCTTGTAACATATTCGACAAAACGGCGAACAGCGAATATATCGATCTCTTTCTAACTTATCACTCATTTTCTATATAAGCGATACACAAACGAGACGCACGATTATTGTGTTTTTAACAGTGACAGTGTGAAGTTAGAATTGTGAAAGAAAGA

>H4.1_D_virilis droVir1_dna range=scaffold_2413:850-1152 CG31613-RA CG31611-RA >droVir1_dna range=scaffold_2413:850-1152 CG31613-RA CG31611-RA

TCTTTCTTTCACAATTCCAACTTCACACTGTCACTGTTAAAAACACAATAATCGTGCGTCTCGTTTGTGTATCGCTTATATAGAAAATGAGTGATAAGTTAGAAAGAGATCGATATATTCGCTGTTCGTCGCTTTGCCGAATATGTTACGAGGGTAGTGTGTGAGCGAGATGGAAATATTCTTGTACTCTTTCGAGTTTTGGATATTTTTGGTATAAATATAAGCGCTGCCCGAAGAGCTAGCATTAGTACCTCAGTGACATTCGTGGTGTAAAGTGAAGTAAAAAAAAGGAAAAGTGAAAAA

>H2B.1_D_virilis droVir1_dna range=scaffold_1633:2653-2898 CG31618-RA CG17949-RA >droVir1_dna range=scaffold_1633:2653-2898 CG31618-RA CG17949-RA

TTTTCTTATTTTCTTTATTTCACTGTTTCACTGCTAGCACACAAATATCGAATGTTATTGACATCAAACCTCACGACTATTGTATTTATACTTTTTTCAACTAACCACTTCTTCAGTTTAGAGTAGAGAGTTGAACGGGCTGTTAACATGAAAGCGTCGTTCGAAAAAAGTATAAATGCTAAGCAACTGTAGGGTGTCTGTCAAACATTTGTGGAGTGAATTAAAGTGAACGTTTGTGAATAATAA

>H2A.1_D_virilis droVir1_dna range=scaffold_18:1618868-1619110 CG31618-RA CG17949-RA >droVir1_dna range=scaffold_18:1618868-1619110 CG31618-RA CG17949-RA

TTTTATTTTTCTTATTTCACTGTTTCACTGCTTACACACAAATATCGAATGTTATTGACATCAAACCTCACGACTATTGTATTTATACTTTTTTCAACTAACCACTTCTTCAGTTTAGAGTAGAGAGTTGAACGGGCTGTTAACATGAAAGCGTCGTTCGAAAAAAGTATAAATGCTAAGCTACTTTGGGGTGTGTGTCAAACATTTGTGAAGTGAATTAAAGTGAACGTTTGTGAATAATAA

>H2A.1_D_mojavensis droMoj1_dna range=contig_4973:7684-7933 CG31618-RA CG17949-RA >droMoj1_dna range=contig_4973:7684-7933 CG31618-RA CG17949-RA

TTATATCCCTTTTATTCTTCTTACACTTTTTCACTCAACAAACAAGAATCGAATGCCCAAAACCCGAACAATGTCAACTTATACTTTTTTACCTACCTACTTTTTCAGCTAAAGAGGGTGAGTTGTGCTGTGCGCAAACATGAAAACGTTGTTCGAAAAATCTTACAAACGTTAACCAAGTTCTATATTTCTGTCAAACATTTGGGAAGTGAGTAAAAGTGAACTTGAGTGAATAATACAATAATAGTAA

>H2A_H2B_S_purpuratus ref|NW_791222.1|SpuUn_WGA357_1:140158-140678 Strongylocentrotus purpuratus chromosome Un genomic contig, whole genome shotgun sequence

TGATGAGTATCTCTACGAAGTAAACGATGAGAATGAACTGCCAAGCGAATCCACTTCTATTTATACAGCGAGCGAGGATCCAGCGGTTATACGTTTATGAAAATTAGTCCGACTGCACGCGAGAAACACCAATCACTGCAAGCCATTCAGCGCGGTTTCGCTCTGTGTACGAGAGAACGACGGCCCCCTGAATTAATTCATTATTCATGAGGTCCGAATGCACGCCTGAGCCACCAATCACACAGAGCGCTCTACGTAAATACGCAGGGCCCCCGCTGTTCGGGCGACACATTTGCATACACCCGTGCAAAAGCATGTGCGTACACTCGCACGTATATGCAAATAATAGTGTGTTCGCTTGCCGTTACTCATCGGCCCCGCATCTGATTGGCTCCCGTTGGATCCTCGCTGTGCGTTTCGATCCTCCACAGACGTATAAATCCCTAGCTCGCACCAATTTGGAAGCATACAGCGATTCTCATCTTACTTGCCATAGCATAACCAAATCTTTCAAATCATCA

>H3_H4_S_purpuratus ref|NW_783816.1|SpuUn_WGA29134_1:41995-43009 Strongylocentrotus purpuratus chromosome Un genomic contig, whole genome shotgun sequence

TTTTGATTTGCTGAATGTGATATCAACTGATGATTCAAAAATCCCGCACTCGTATTTATATCAAAATTGTACGAAGATGATTGGTTAGTCGTGCGACAAGCAGTGATTGGCCATTCGCTATATCAAGCACAGTATGTGATTGGGTACTGTACGCTTTCCTAATATATTCATGATTTCTTCGGGCCTCGTCGTGTGATTTTGTACATGGTCATTGTAGTAAACGTTATAAGATTATGAAATATTAAACTTAATTACATCCTAAATTGCATCGTTATCGCTGTAAAGTAGTATTCGTAATTGTATTACAGAAATTTATACGCTAATACGTAATATTCGTTAGGTTTTAAGAGAAGAAAGGTCGTACTGGATTGGATTTTCGAGATCGCTGTAAAAATGAATAATTCATTAACAAACCGGCTTTCCTGTCGTCGACATTTTGGCTTTTTGAGAAGTATGTTATCATTTATCAATAACAGCATCCATCAAAATTGAAAATGAAATGATCGATATTGAATAGAAAATGATATGGACTATTTTTTCACCAAACAAAAGTAGAAATTGTAAGAATATTTCCACGTTGTTTTCTATTTTGGCGAGCAAACGACTTCAAGGAGTCCTGATGAATTATTCATAAGGTGCTGGCGACCTGTTGTAAACGATTTTGGTCAAGTAAAATACATTCATGTTCTCTTGTCTACTGTGCAATATGTAAACTAATACGCTTAAATACTGTATTGAGATGAATTAAAGTGTTTAAATAGTATTTGATAACCGGAACTCTGTTCTTGAATTTTTTATACCGATTTGAGTAAAGAATTTGTTCATAACGAACGTGCGATAGTTTATGCAAATATTGCATCGACGAAGCGACGAGATTTTAGTCCCCTTGACCGTAGGCGGCTCACTCTCAATAGAAATTATTTAGTACACTCGCGTCCGCAAAGGAGTTATATATACCCGCCGAAACTCACTTTTCAGTACAGATTTCTTCAATCGTCAAATCTACAAGTCAATA

>his-67_H4_his-68_H2A_C_elegans ref|NC_003279.3|:9988751-9989108 Caenorhabditis elegans chromosome I, complete sequence

TTGTTGAGTGATTGGCTGAAGACTCACGAATGATGATGAACTGGGAAAACAGTTCTTCTTATATACTCCCTATGCAGATACAGCGGTGCAAAGGCGGAGTCAGTTTGCGCCATCCTGCAGGGACACATTTCCGTGCCTGCCGCTCTTGCATCTGCAGAGATATCTGCCGTTTTGGCCTCTAGTAGGCGAGACATACAGGAAGGTGAGAGAGACGCAGAAACGAGATAAATGTGTCCCCGCAGGAATTTACCGAATGTTCAAAAAGAGGTGTCCCCGCAGGAAACTCCGCCCCGCCACCGTAAAAATAAATATAAAGCGACTGTCTTGGTTCATTCTTCAGATTAGTTTTACGATCACC

>his-10_H4_his-9_H3_C_elegans ref|NC_003280.3|:13823272-13823545 Caenorhabditis elegans chromosome II, complete sequence

TTGTTGACAATTGATGAAGACTCACAAGTGAGATGAAGAACTGATTGAAATTGCCTTCTTTATATACCCCTAATGTCTACTAACAGGTGGGAAGAGGTGGAGTCACATTAAGCCATCCTGTGGGGACACATTTCATAGTCTACCTAACACACCGAATGTCTAACTGTCGTCTGTCTCTTCCTTCCCTCCGCCTACCCACCCCACCTGCATATAAAAGAAACGATAAGGTCTCCCTTTTCTTCACAGTCCCCACGGATTACCAACCAAAGCATCA

>his-12_H2A_his-11_H2B_C_elegans ref|NC_003280.3|:13821478-13821706 Caenorhabditis elegans chromosome II, complete sequence

TTGTTTGATTGATAGAATCGAATCTGATGAGGCAACTGCAGAGACACTATTATATACAATTTCTGTGGTGGGTAGGCGGAGACACTGCGGGGACACATTTCAAGCGATAAGCATCAGACCGAATGTCACCTGTCCCTTCAATGACTCCTCCTTCCCACCACAGACACAAATAAAAGAGAAGGGAGACAAGGGTCTCCTCACACTTGTTTCAAGTCACCAACTCTCAACA

>his-14_H4_his-13_H3_C_elegans ref|NC_003280.3|:13819834-13820107 Caenorhabditis elegans chromosome II, complete sequence

TTGTTGACAATTGATGAAGACTCACAAGTGAGATGAAGAACTGATTGAAATTGCCTTCTTTATATACCCCTAATGTCTACTAACAGGTGGGAAGAGGTGGAGTCACATTAAGCCATCCTGTGGGGACACATTTCATAGTCTACCTAACACACCGAATGTCTAACTGTCGTCTGTCTCTTCCTTCCCTCCGCCTACCCACCCCACCTGCATATAAAAGAAACGATAAGGTCTCCCTTTTCTTCACAGTCCCCACGGATTACCAACCAAAGCATCA

>his-16_H2A_his-15_H2B_C_elegans ref|NC_003280.3|:13818040-13818268 Caenorhabditis elegans chromosome II, complete sequence

TTGTTTGATTGATAGAATCGAATCTGATGAGGCAACTGCAGAGACACTATTATATACAATTTCTGTGGTGGGTAGGCGGAGACACTGCGGGGACACATTTCAAGCGATAAGCATCAGACCGAATGTCACCTGTCCCTTCAATGACTCCTCCTTCCCACCACAGACACAAATAAAAGAGAAGGGAGACAAGGGTCTCCTCACACTTGTTTCAAGTCACCAACTCTCAACA

>his-25_H3_his-26_H4_C_elegans ref|NC_003280.3|:13825156-13825429 Caenorhabditis elegans chromosome II, complete sequence

TGATGCTTTGGTTGGTAATCCGTGGGGACTGTGAAGAAAAGGGAGACCTTATCGTTTCTTTTATATGCAGGTGGGGTGGGTAGGCGGAGGGAAGGAAGAGACAGACGACAGTTAGACATTCGGTGTGTTAGGTAGACTATGAAATGTGTCCCCACAGGATGGCTTAATGTGACTCCACCTCTTCCCACCTGTTAGTAGACATTAGGGGTATATAAAGAAGGCAATTTCAATCAGTTCTTCATCTCACTTGTGAGTCTTCATCAATTGTCAACAA

>his-43_H2A_his-42_H3_C_elegans ref|NC_003280.3|:13827606-13828184 Caenorhabditis elegans chromosome II, complete sequence

GGCATCCTTCAACACACCTAACCGAACCCAACGGCCCTCTTTAGGGCCACAAATGTTATAAATCCTATTATAAACTGAATAAATCTTATCAAACATGTTTTGCATTGCCATAATATTACCTGAAAAACTGAAAATTAAGAAATTCGTATCTAAAATTTAGAATAGTCACCAGTATCATCAGGAACGATTACTAATTGTGACGTCGAAATATCTGAATTCGGGTAATCTTGAGCTGCAGATAACGTTTCCTTCTTTGAAAATTGCAATAGCAGTGAATTTGGCACTTGTTGGATAAATTTATTGTATAACGCGGCCTGAAGTTGTTTTTTTTAAACGAGCACATTTACAGAAATAATTGCAAAACTTTTTGAGACGGATTCATTCCTACTTTTGCCATTAAATAAATGAACTAAATTTCAAAAGTGTACACTAGAAATATTCAATACACAATGACAAAAAACAACAATACAAATTTATTAGAAATCAAGCCGGATTATATTACATTTGTGGCCCTAAAGAGGGCCGTTGGGTTCGGTTAGATTTTGAGATCAAGCTGGCTCAGTACCATTGGAAGGCATT

>his-29_H2B_his-30_H2A_C_elegans ref|NC_003282.3|:8331394-8331648 Caenorhabditis elegans chromosome IV, complete sequence

TGATGAGAATGGACTTGTGACAAGTATAAAGAAATGGGGTTGTATAGAGATTATTATATACAAACAATGCGGTGGGAAGGCGGAGTCTCTCTGCAGGGACACATGTCTCACTTGCACCTGTACTGCCGAATGCACATAGACAATAGATGGTGTCTCCCCGATAAGACCCTCCCCCACCATCACCGCACAGAATACATAAAAAGGGGTCATTGAATACACCTCACCAGTTTACGAACAAAAACACTCAACTAGCAA

>his-31_H4_his-32_H3_C_elegans ref|NC_003282.3|:8333965-8334280 Caenorhabditis elegans chromosome IV, complete sequence

TTGTTGAGTGATTGAATTGAAGACTCACGAATGAGATGAAAAAATGAAGAAATGAGGCTGTCTTTTATACCAGCTGCGGTGGATGTAGGAGGGATCACATTGAACCATCCTGTTAGGACACATGGTGTACCACTCTCTCACTCTCTATCTCTTCCATTTTCTGTAGATAGGTCATTCGGTTAGAATGCAAACACTGAGATGTGTCCCTGCAGGGGAATGAATGAGGTCTCCGCCTTCTCACCCCAATATTACACTTCTTCGTATAAAAGGGGACAGACTAAAGGTTTTTTCACAGTCCCCTCGGATTTTCATCTCA

>his-33_H2A_his-34_H2B_C_elegans ref|NC_003282.3|:8335700-8335954 Caenorhabditis elegans chromosome IV, complete sequence

TTGCTAGTTGAGTGTTTTTGTTCGTAAACTGGTGAGGTGTATTCAATGACCCCTTTTTATGTATTCTGTGCGGTGATGGTGGGGGAGGGTCTTATCGGGGAGACACCATCTATTGTCTATGTGCATTCGGCAGTACAGGTGCAAGTGAGACATGTGTCCCTGCAGAGAGACTCCGCCTTCCCACCGCATTGTTTGTATATAATAATCTCTATACAACCCCATTTCTTTATACTTGTCACAAGTCCATTCTCATCA

>his-46_H4_his-45_H3_C_elegans ref|NC_003282.3|:11326300-11326528 Caenorhabditis elegans chromosome IV, complete sequence

TTGTTGAATTCGTTGAAGACTCACAAGTGAGATGAAGAAATGACATCACAGTATTCCTTTTGTACACAGCTGTGGTGGCTAGGCGGAGTCACATTGCACCACTCTGCGAGGACACCTTTCCTTTGTCTGTAACACACCGAATGTTCTAATTGCCACAAGGACAGAAGTGCGCCCGCCCGCCCTCCCACCGCAGCTGCGTACAAAGGCAGTCGTTGGTTTCTCTTTTTTA

>his-47_H2A_his-48_H2B_C_elegans ref|NC_003282.3|:11324410-11324712 Caenorhabditis elegans chromosome IV, complete sequence

TGATGAGCGATTGGTGTTCGTAAGACGAATCTGAAGAATGAATCGAAACTGTCTGTTTATATTCATTTCTACGGTGGAGGGGCGGAGTTTCATGCGGGGACACCTCTATTTGAACATTCCGCACTCTCTGGTAGGACACATTTCTCTCACTTCTCTTTGTCCCCCTATTAACCTCGGTACATTCGGAATTTTGTGTGCACTCAGTAATGTGTCCCCGCGGGAAGCTCCGCCCCTCCACCGCAGTTTTTACATAAATAGGTGTCTCTCCAACCATTTCCTCATACTTGATTCAAGTTCATTCTC

>his-55_H3_his-56_H4_C_elegans ref|NC_003282.3|:11338234-11338493 Caenorhabditis elegans chromosome IV, complete sequence

TTGCGTTTAGGTTGATAATCCGTGGGGACTGTAAAAAAGAGAAACCAACGACTGACTTTGTACGCAGCTGCGGTGGGAGGGCGGGCGGGCGCACTTCTGTCCTTGTGGCAATTAGAACATTCGGTGTGTTACAGACAAAGGAAAGGTGTCCTCGCAGAGTGGTGCAATGTGACTCCGCCTAGCCACCACAGCTGTGTACAAAAGGAATACTGTGATGTCATTTCTTCATCTCACTTGTGAGTCTTCAACGAATTCAACAA

>his-58_H2B_his-57_H2A_C_elegans ref|NC_003282.3|:11340077-11340383 Caenorhabditis elegans chromosome IV, complete sequence

TGATGAGAATGAACTTGAATCAAGTATGAGGAAATGGTTGGAGAGACACCTATTTATGTAAAAACTGCGGTGGAGGGGCGGAGCTTCCCGCGGGGACACATTACTGAGTGCACACAAAATTCCGAATGTACCGAGGTTAATAGGGGGACAAAGAGAAGTGAGAGAAATGTGTCCTACCAGAGAGTGCGGAATGTTCAAATAGAGGTGTCCCCGCATGAAACTCCGCCCCTCCACCGTAGAAATGAATATAAACAGACAGTTTCGATTCATTCTTCAGATTCGTCTTACGAACACCAATCGCTCATCA

>his-63_H3_his-64_H4_C_elegans ref|NC_003282.3|:11406832-11407087 Caenorhabditis elegans chromosome IV, complete sequence

TTGCGTTTAGGTTGATAATCCGTGGGGGACTGTAAAAAAAAAGAAACCAAGCTGCCTTTGTACGCAGGTGCGGTGGGAGGGCGGGCGCACTACTGTCCTTGTGGCAAGTAGAACATTCGGTGTGTTACAGACAAAGGAAAGGTGTCCTCGCAGCGTGGTGCAATGTGACTCCGCCTACCCACCACAGTTGTGTACAAAAGGAATACTGTGATGTCATTTCTTCATCTCACTTGTGAGTCTTCAACTAGTTCAACAA

>his-66_H2B_his-65_H2A_C_elegans ref|NC_003282.3|:11400757-11401063 Caenorhabditis elegans chromosome IV, complete sequence

TGATGAGAATGAACTTGAATCAAGTATGAGGAAATGGTTGGAGAGACACCTATTTATGTAAAAACTGCGGTGGAGGGGCGGAGCTTCCCGCGGGGACACATTACTGAGTGCACACAAAATTCCGAATGTACCGAGGTTAATAGGGGGACAAAGAGAAGTGAGAGAAATGTGTCCTACCAGAGAGTGCGGAATGTTCAAATAGAGGTGTCCCCGCATGAAACTCCGCCCCTCCACCGTAGAAATGAATATAAACAGACAGTTTCGATTCATTCTTCAGATTCGTCTTACGAACACCAATCGCTCATCA

>his-2_H3_his-1_H4_C_elegans ref|NC_003283.4|:16044866-16045103 Caenorhabditis elegans chromosome V, complete sequence

TGTCAATTGGAATCCGATAAGGACTGTGAAGAAAGGAGACCCATACACTGCATTTATACAAGGTTGTGGTGGGAAGGCGGGGAGGTGAGAGAGTGGGCAAAAGTGAGAGAGTGCAAGGATATCAAGACATATGTATCCCCGCAGTGTCTCCGCCTTCACACCGCATAGTGGTATAAAAGGGGCAGCCAACTGCCCATTTCTTCATCTCACTTGTGAGTCTTCAAGTCAACTGCAACAA

>his-4_H2B_his-3_H2A_C_elegans ref|NC_003283.4|:16043152-16043377 Caenorhabditis elegans chromosome V, complete sequence

TTTCCTTTATAATAATTCATCGGTGGGAAGGAGGAGCCTATGGACATACTGCGGGGACACATTTCTATGTAGTCTCCCCAGCCGAATACGCAGAGTGCACAGTTTTTCTCGGTCACTCTGCCACCTCCTCCACCTGCCTTGTGTCTATGTGCCCGCCTAGTCACCGCAGTTGTGTTTAAAAGAGTGTGGTCTAATGGTCTCCTTCAGATCGTTTTTACATCTACCA

>his-6_H3_his-5_H4_C_elegans ref|NC_003283.4|:8537565-8537792 Caenorhabditis elegans chromosome V, complete sequence

TTTCAATTGGAATCCGATAGAGACTGTGAAGAAAGGAGACATGTACACTGCATTTATACAAGGTTGTGGTGGAGAGGCGGGGAGGTGAGAGAGTGGGTGAGAGTGAGTGAGTACAAAGATAACAAGACAAATGTGTCCCCGCAGTGTCTCCTCCTTCACACCGCAAAGTGGTATAAAAGGGGCAGCCAACTGTCCATTTCTTCATCTCACTTGTGAGTCTTCATTCAA

>his-8_H2B_his-7_H2A_C_elegans ref|NC_003283.4|:8535675-8535968 Caenorhabditis elegans chromosome V, complete sequence

TTTCGATTGATTGGACTGGTCGTTGGGCAAGTGTAAGGAATGGGTTGATGGTAATTTCTTTTATAACAATTTTTCGGTGGAAAGGAGGAGCCTATTGACATTCTGTGGGGACATCTTTCTATGCTGTCTCTCATGCCGAATGTACAAAGTGCACAGTTTTTCTTGGTCACTCTGCCACCTCCCCCATCTGTCTTATCTCTATGTGCCCGCCTAGTCACCGCAGCTGTGTTTAAAAGGGTGCTGTCTAATGGTCTTCTTCAGATTGTTTCTACATCTACCAACCTATCACCACCA

>his-17_H3_his-18_H4_C_elegans ref|NC_003283.4|:8900579-8900815 Caenorhabditis elegans chromosome V, complete sequence

TTTCAATTGGAATCCGATAGGGACTGTGAATAAAGGAGACATGTACACTGCATTTATACAAGGTTCTGGTGGAGAGGCGGGGAAGTTAGAGAGTAGGTAAGAGTGAGTGAGAACAAAGATAACAAGACAAATGTGTCCCCGCAGTGTCTCCTCCTTCACACCGCATAGTGGTATAAAAGGGACAGCCAACTGTCCATTTCTTCATCTCACTTGTGAGTCTTCATTCAACTGCAACAA

>his-20_H2B_his-19_H2A_C_elegans ref|NC_003283.4|:8898059-8898352 Caenorhabditis elegans chromosome V, complete sequence

TTTCGATTGATTGGACTGGTCGTTGGACAAGTGTAAGGAATGGGTTGTTGGCCATTACCTTTATAACAATTTTTCGGTGGGAAGGAGGAGCCTATTGACATTCTGTGGGGACACCTTTCTATGCTGTCTCTCATGCCGAATGTACAAAGTGCATAGTTTTTCTTGGTCACTCTGCCACTTCCCCCATCTGTCTTATCTCTATGTGCCCGCCTAGTCACCGCAGCTGTGTTTAAAAGGGTGCTGTCTAATGGTCTTCTTCAGATCGTTTCTACATCTACCAACCAATCATCACCA

>his-21_H2A_his-22_H2B_C_elegans ref|NC_003283.4|:8894853-8895146 Caenorhabditis elegans chromosome V, complete sequence

TGGTGATGATTGGTTGGTAGATGTAGAAACGATCTGAAGAAGACCATTAGACAGCACCCTTTTAAACACAGCTGCGGTGACTAGGCGGGCACATAGAGATAAGACAGATGGGGGAAGTGGCAGAGTGACCAAGAAAAACTATGCACTTTGTACATTCGGCATGAGAGACAGCATAGAAAGGTGTCCCCACAGAATGTCAATAGGCTCCTCCTTCCCACCGAAAAATTGTTATAAAGGTAATGGCCAACAACCCATTCCTTACACTTGTCCAACGACCAGTCCAATCAATCGAAA

>his-28_H4_his-27_H3_C_elegans ref|NC_003283.4|:8892386-8892625 Caenorhabditis elegans chromosome V, complete sequence

TTTTGTTTGCAGTTGAATGAAGACTCACAAGTGAGATGAAGAAATGGACAGTTGGCTGTCCCTTTTATACCACTATGCGGTGTGAAGGAGGAGACACTGCGGGGACACATTTGTCTTGTTATCTTTGTTCTCACTCACTCTTACCTACTCTCTAACTTCCCCGCCTCTCCACCAGAACCTTGTATAAATGCAGTGTACATGTCTCCTTTATTCACAGTCCCTATCGGATTCCAATTGAAA

>his-50_H4_his-49_H3_C_elegans ref|NC_003283.4|:8850291-8850530 Caenorhabditis elegans chromosome V, complete sequence

TTTTGTTTGCAGTTGAATGAAGACTCACAAGTGAGATGAAGAAATGGACAGTTGGCTGTCCCTTTTATACCACTATGCGGTGTGAAGGAGGAGACACTGCGGGGACACATTTGTCTTGTTATCTTTGTTCTCACTCACTCTTACCTACTCTCTAACTTCCCCGCCTCTCCACCAGAACCTTGTATAAATGCAGTGTACATGTCTCCTTTATTCACAGTCCCTATCGGATTCCAATTGAAA

>his-51_H2A_his-52_H2B_C_elegans ref|NC_003283.4|:8852758-8852997 Caenorhabditis elegans chromosome V, complete sequence

TGGTGATGATTGGTTGGTAGATGTAGAAACGATCTGAAGAAGACCATTAGACAGCACCCTTTTAAACACAGCTGCGGTGACTAGGCGGGCACATAGAGATAAGACAGATGGGGGAAGTGGCAGAGTGACCAAGAAAAACTATGCACTTTGTACATTCGGCATGAGAGACAGCATAGAAAGGTGTCCCCACAGAATGTCAATAGGCTCCTCCTTCCCACCGAAAAATTGTTATAAAGGTAA

>his-54_H2B_his-53_H2A_C_elegans ref|NC_003283.4|:8856018-8856257 Caenorhabditis elegans chromosome V, complete sequence

TTACCTTTATAACAATTTTTCGGTGGGAAGGAGGAGCCTATTGACATTCTGTGGGGACACCTTTCTATGCTGTCTCTCATGCCGAATGTACAAAGTGCATAGTTTTTCTTGGTCACTCTGCCACTTCCCCCATCTGTCTTATCTCTATGTGCCCGCCTAGTCACCGCAGCTGTGTTTAAAAGGGTGCTGTCTAATGGTCTTCTTCAGATCGTTTCTACATCTACCAACCAATCATCACCA
