# Supplementary material for: Multiple independent evolutionary solutions to core histone gene regulation
Source: Genome Biol. 2006 Dec 21;7(12):R122. doi: 10.1186/gb-2006-7-12-r122 (PMC1794435; doi:10.1186/gb-2006-7-12-r122)
Supplement: Additional data file 2 — Core histone protein sequences used in the study [file gb-2006-7-12-r122-S2.doc]

>H4_H_sapiens gi|4504323|ref|NP_003539.1| histone 2, H4; H4 histone, family 2; histone IV, family 2; H4 histone family, member N [Homo sapiens]

MSGRGKGGKGLGKGGAKRHRKVLRDNIQGITKPAIRRLARRGGVKRISGLIYEETRGVLKVFLENVIRDAVTYTEHAKRKTVTAMDVVYALKRQGRTLYGFGG

>H4_S_cerevisiae gi|6324298|ref|NP_014368.1| One of two identical histone H4 proteins (see also HHF1); core histone required for chromatin assembly and chromosome function; contributes to telomeric silencing; N-terminal domain involved in maintaining genomic integrity; Hhf2p [Saccharomyces cerevisiae]

MSGRGKGGKGLGKGGAKRHRKILRDNIQGITKPAIRRLARRGGVKRISGLIYEEVRAVLKSFLESVIRDSVTYTEHAKRKTVTSLDVVYALKRQGRTLYGFGG

>H4_S_bayanus MIT_Sbay_c805_21528

MSGRGKGGKGLGKGGAKRHRKILRDNIQGITKPAIRRLARRGGVKRISGLIYEEVRAVLKSFLESVIRDSVTYTEHAKRKTVTSLDVVYALKRQGRTLYGFGG

>H4_S_mikatae MIT_Smik_c144_1254

MSGRGKGGKGLGKGGAKRHRKILRDNIQGITKPAIRRLARRGGVKRISGLIYEEVRAVLKSFLESVIRDSVTYTEHAKRKTVTSLDVVYALKRQGRTLYGFGG

>H4_S_paradoxus MIT_Spar_c197_1029

MSGRGKGGKGLGKGGAKRHRKILRDNIQGITKPAIRRLARRGGVKRISGLIYEEVRAVLKSFLESVIRDSVTYTEHAKRKTVTSLDVVYALKRQGRTLYGFGG

>H4_S_castellii WashU_Scas_Contig671.8

MSGRGKGGKGLGKGGAKRHRKILRDNIQGITKPAIRRLARRGGVKRISGLIYEEVRAVLKSFLESVIRDAVTYTEHAKRKTVTSLDVVYALKRQGRTLYGFGG

>H4_S_kluyveri WashU_Sklu_Contig2251.2

MSGRGKGGKGLGKGGAKRHRKILRDNIQGITKPAIRRLARRGGVKRISGLIYEEVRTVLKTFLESVIRDAVTYTEHAKRKTVTSLDVVYALKRQGRTLYGFGG

>H4_S_kudriavzevii gb|AACI01001558.1| Saccharomyces kudriavzevii IFO 1802 YM6553-Contig334

MSGRGKGGKGLGKGGAKRHRKILRDNIQGITKPAIRRLARRGGVKRISGLIYEEVRAVLKSFLESVIRDSVTYTEHAKRKTVTSLDVVYALKRQGRTLYGFGG

>H4_C_albicans gb|AACQ01000120.1| Candida albicans SC5314 chromosome 1 Ctg19-20087

TGRGKGGKGLGKGGAKRHRKILRDNIQGITKPAIRRLARRGGVKRISALIYEEVRVVLKQFLENVIRDAVTYTEHAKRKTVTSLDVVYALKRQGRTLYGFGG

>H4_K_waltii gb|AADM01000056.1| Kluyveromyces waltii NCYC 2644 cont55

MSGRGKGGKGLGKGGAKRHRKILRDNIQGITKPAIRRLARRGGVKRISGLIYEEVRAVLKSFLESVIRDAVTYTEHAKRKTVTSLDVVYALKRQGRTLYGFGG

>H4_A_gossypii gi|51890951|gb|AE016817.2| Ashbya gossypii (= Eremothecium gossypii) ATCC 10895 chromosome IV

MSGRGKGGKGLGKGGAKRHRKILRDNIQGITKPAIRRLARRGGVKRISGLIYEDVRAVLKSFLESVIRDAVTYTEHAKRKTVTSLDVVYALKRQGRTLYGFGG

>H4_C_elegans ref|NM_076830.1| Caenorhabditis elegans K03A1.6 (histone-H4) mRNA, complete cds

MSGRGKGGKGLGKGGAKRHRKVLRDNIQGITKPAIRRLARRGGVKRISGLIYEETRGVLKVFLENVIRDAVTYCEHAKRKTVTAMDVVYALKRQGRTLYGFGG

>H4_D_ananassae scaffold_13030

MTGRGKGGKGLGKGGAKRHRKVLRDNIQGITKPAIRRLARRGGVKRISGLIYEETRGVLKVFLENVIRDAVTYTEHAKRKTVTAMDVVYALKRQGRTLYGFGG

>H4_D_simulans chrU

MTGRGKGGKGLGKGGAKRHRKVLRDNIQGITKPAIRRLARRGGVKRISGLIYEETRGVLKVFLENVIRDAVTYTEHAKRKTVTAMDVVYALKRQGRTLYGFGG

>H4_D_melanogaster chr2L

MTGRGKGGKGLGKGGAKRHRKVLRDNIQGITKPAIRRLARRGGVKRISGLIYEETRGVLKVFLENVIRDAVTYTEHAKRKTVTAMDVVYALKRQGRTLYGFGG

>H4_D_virilis scaffold_13209

MTGRGKGGKGLGKGGAKRHRKVLRDNIQGITKPAIRRLARRGGVKRISGLIYEETRGVLKVFLENVIRDAVTYTEHAKRKTVTAMDVVYALKRQGRTLYGFGG

>H4_D_yakuba chrU

MTGRGKGGKGLGKGGAKRHRKVLRDNIQGITKPAIRRLARRGGVKRISGLIYEETRGVLKVFLENVIRDAVTYTEHAKRKTVTAMDVVYALKRQGRTLYGFGG

>H4_D_pseudoobscura chr2

MTGRGKGGKGLGKGGAKRHRKVLRDNIQGITKPAIRRLARRGGVKRISGLIYEETRGVLKVFLENVIRDAVTYTEHAKRKTVTAMDVVYALKRQGRTLYGFGG

>H4_D_mojavensis scaffold_6584

MTGRGKGGKGLGKGGAKRHRKVLRDNIQGITKPAIRRLARRGGVKRISGLIYEETRGVLKVFLENVIRDAVTYTEHAKRKTVTAMDVVYALKRQGRTLYGFGG

>H4_A_nidulans (AN0734.3) hypothetical protein similar to histone H4 (translation)

MSGRGKGGKGLGKGGAKRHRKILRDNIQGITKPAIRRLARRGGVKRISAMIYEETRGVLKTFLEGVIRDAVTYTEHAKRKTVTSLDVVYALKRQGRTLYGFGG

>H4_S_pombe ref|NC_003423.1| Schizosaccharomyces pombe chromosome II, complete sequence

MSGRGKGGKGLGKGGAKRHRKILRDNIQGITKPAIRRLARRGGVKRISALVYEETRAVLKLFLENVIRDAVTYTEHAKRKTVTSLDVVYSLKRQGRTIYGFGG

>H4_A_thaliana ref|NP_563797.1| DNA binding [Arabidopsis thaliana]

MSGRGKGGKGLGKGGAKRHRKVLRDNIQGITKPAIRRLARRGGVKRISGLIYEETRGVLKIFLENVIRDAVTYTEHARRKTVTAMDVVYALKRQGRTLYGFGG

>H4_S_purpuratus ref|NW_783816.1|SpuUn_WGA29134_1 Strongylocentrotus purpuratus chromosome Un genomic contig

MSGRGKGGKGLGKGGAKRHRKVLRDNIQGITKPAIRRLARRGGVKRISGLIYEETRGVLKVFLENVIRDAVTYCEHAKRKTVTAMDVVYALKRQGRTLYGFGG

>H4_M_musculus ref|NP_835515.1| histone 1, H4c [Mus musculus]

MSGRGKGGKGLGKGGAKRHRKVLRDNIQGITKPAIRRLARRGGVKRISGLIYEETRGVLKVFLENVIRDAVTYTEHAKRKTVTAMDVVYALKRQGRTLYGFGG

>H3_H_sapiens gi|4504281|ref|NP_003520.1| H3 histone family, member A [Homo sapiens]

MARTKQTARKSTGGKAPRKQLATKAARKSAPATGGVKKPHRYRPGTVALREIRRYQKSTELLIRKLPFQRLVREIAQDFKTDLRFQSSAVMALQEACEAYLVGLFEDTNLCAIHAKRVTIMPKDIQLARRIRGERA

>H3_S_cerevisiae gi|6319482|ref|NP_009564.1| One of two identical histone H3 proteins (see also HHT2); core histone required for chromatin assembly, involved in heterochromatin-mediated telomeric and HM silencing; regulated by acetylation, methylation, and mitotic phosphorylation; Hht1p [Saccharomyces cerevisiae]

MARTKQTARKSTGGKAPRKQLASKAARKSAPSTGGVKKPHRYKPGTVALREIRRFQKSTELLIRKLPFQRLVREIAQDFKTDLRFQSSAIGALQESVEAYLVSLFEDTNLAAIHAKRVTIQKKDIKLARRLRGERS

>H3_S_paradoxus gb|AABY01000101.1| Saccharomyces paradoxus NRRL Y-17217 contig_256, whole genome shotgun

MARTKQTARKSTGGKAPRKQLASKAARKSAPSTGGVKKPHRYKPGTVALREIRRFQKSTELLIRKLPFQRLVREIAQDFKTDLRFQSSAIGALQESVEAYLVSLFEDTNLAAIHAKRVTIQKKDIKLARRLRGERS

>H3_S_mikatae gb|AABZ01000043.1| Saccharomyces mikatae IFO 1815 contig_144, whole genome shotgun

MARTKQTARKSTGGKAPRKQLASKAARKSAPSTGGVKKPHRYKPGTVALREIRRFQKSTELLIRKLPFQRLVREIAQDFKTDLRFQSSAIGALQESVEAYLVSLFEDTNLAAIHAKRVTIQKKDIKLARRLRGERS

>H3_S_kudriavzevii gb|AACI01000340.1| Saccharomyces kudriavzevii IFO 1802 YM6553-Contig1789, whole genome -Partial-

KPGTVALREIRRFQKSTELLIRKLPFQRLVREIAQDFKTDLRFQSSAIGALQESVEAYLVSLFEDTNLAAIHAKRVTIQKKDIKLARRLRGERS

>H3_S_bayanus gb|AACA01000594.1| Saccharomyces bayanus MCYC 623 contig_527, whole genome shotgun

MARTKQTARKSTGGKAPRKQLASKAARKSAPSTGGVKKPHRYKPGTVALREIRRFQKSTELLIRKLPFQRLVREIAQDFKTDLRFQSSAIGALQESVEAYLVSLFEDTNLAAIHAKRVTIQKKDIKLARRLRGERS

>H3_S_castelli gb|AACF01000045.1| Saccharomyces castellii NRRL Y-12630 YM476-Contig671, whole genome

MARTKQTARKSTGGKAPRKQLASKAARKSAPSTGGVKKPHRYKPGTVALREIRRFQKSTELLIRKLPFQRLVREIAQDFKTDLRFQSSAIGALQESVEAYLVSLFEDTNLAAIHAKRVTIQKKDIKLARRLRGERS

>H3_C_albicans gb|AACQ01000218.1| Candida albicans SC5314 Ctg19-20133, whole genome shotgun sequence

MARTKQTARKSTGGKAPRKQLASKAARKSAPSTGGVKKPHRYKPGTVALREIRRFQKSTELLIRKLPFQRLVREIAQDFKTDLRFQSSAIGALQEAVEAYLVGLFEDTNLCAIHAKRVTIQKKDMQLARRLRGERS

>H3_S_kluyveri gb|AACE01000155.1| Saccharomyces kluyveri NRRL Y-12651 YM479-Contig2251, whole genome

MARTKQTARKSTGGKAPRKQLASKAARKSAPSTGGVKKPHRYKPGTVALREIRRFQKSTELLIRKLPFQRLVREIAQDFKTDLRFQSSAIGALQESVEAYLVSLFEDTNLAAIHAKRVTIQKKDIKLARRLRGERS

>H3_K_waltii gb|AADM01000056.1| Kluyveromyces waltii NCYC 2644 cont55, whole genome shotgun sequence

MARTKQTARKSTGGKAPRKQLASKAARKSAPSTGGVKKPHRYKPGTVALREIRRFQKSTELLIRKLPFQRLVREIAQDFKTDLRFQSSAIGALQESVEAYLVSLFEDTNLAAIHAKRVTIQKKDIKLARRLRGERS

>H3_A_gossypii gb|AE016818.1| Eremothecium gossypii ATCC 10895 chromosome V, complete sequence

MARTKQTARKSTGGKAPRKQLASKAARKSAPSTGGVKKPHRYKPGTVALREIRRFQKSTELLIRKLPFQRLVREIAQDFKTDLRFQSSAIGALQESVEAYLVSLFEDTNLAAIHAKRVTIQKKDIKLARRLRGERS

>H3_C_elegans ref|NP_507033.1| HIStone family member (his-2) [Caenorhabditis elegans]

MARTKQTARKSTGGKAPRKQLATKAARKSAPASGGVKKPHRYRPGTVALREIRRYQKSTELLIRRAPFQRLVREIAQDFKTDLRFQSSAVMALQEAAEAYLVGLFEDTNLCAIHAKRVTIMPKDIQLARRIRGERA

>H3_D_ananassae scaffold_13030

MARTKQTARKSTGGKAPRKQLATKAARKSAPATGGVKKPHRYRPGTVALREIRRYQKSTELLIRKLPFQRLVREIAQDFKTDLRFQSSAVMALQEASEAYLVGLFEDTNLCAIHAKRVTIMPKDIQLARRIRGERA

>H3_D_simulans chrU

MARTKQTARKSTGGKAPRKQLATKAARKSAPATGGVKKPHRYRPGTVALREIRRYQKSTELLIRKLPFQRLVREIAQDFKTDLRFQSSAVMALQEASEAYLVGLFEDTNLCAIHAKRVTIMPKDIQLARRIRGERA

>H3_D_melanogaster chr2L

MARTKQTARKSTGGKAPRKQLATKAARKSAPATGGVKKPHRYRPGTVALREIRRYQKSTELLIRKLPFQRLVREIAQDFKTDLRFQSSAVMALQEASEAYLVGLFEDTNLCAIHAKRVTIMPKDIQLARRIRGERA

>H3_D_virilis scaffold_13047

MARTKQTARKSTGGKAPRKQLATKAARKSAPATGGVKKPHRYRPGTVALREIRRYQKSTELLIRKLPFQRLVREIAQDFKTDLRFQSSAVMALQEASEAYLVGLFEDTNLCAIHAKRVTIMPKDIQLARRIRGERA

>H3_D_yakuba chr2L

MARTKQTARKSTGGKAPRKQLATKAARKSAPSTGGVKKPHRYRPGTVALREIRRYQKSTELLIRKLPFQRLVREIAQDFKTDLRFQSSAVMALQEASEAYLVGLFEDTNLCAIHAKRVTIMPKDIQLARRIRGERA

>H3_D_pseudoobscura chr4_group4

MARTKQTARKSTGGKAPRKQLATKAARKSAPSTGGVKKPHRYRPGTVALREIRRYQKSTELLIRKLPFQRLVREIAQDFKTDLRFQSAAILQLQEASEAYLVGLFEDTNLCAIHAKRVTIMPKDIQLARRIRGERA

>H3_D_mojavensis scaffold_6540

MARTKQTARKSTGGKAPRKQLATKAARKSAPATGGVKKPHRYRPGTVALREIRRYQKSTELLIRKLPFQRLVREIAQDFKTDLRFQSSAVMALQEASEAYLVGLFEDTNLCAIHAKRVTIMPKDIQLARRIRGERA

>H3_A_nidulans (AN0733.3) histone H3 (translation)

MARTKQTARKSTGGKAPRKQLASKAARKAAPSTGGVKKPHRYKPGTVALREIRRYQKSTELLIRKLPFQRLVREIAQDFKSDLRFQSSAIGALQESVEAYLVSLFEDTNLCAIHAKRVTIQSKDIQLARRLRGERS

>H3_S_pombe ref|NC_003423.1| Schizosaccharomyces pombe chromosome II, complete sequence

MARTKQTARKSTGGKAPRKQLASKAARKAAPATGGVKKPHRYRPGTVALREIRRYQKSTELLIRKLPFQRLVREIAQDFKTDLRFQSSAIGALQEAVEAYLVSLFEDTNLCAIHGKRVTIQPKDMQLARRLRGERS

>H3_A_thaliana ref|NP_568227.1| DNA binding [Arabidopsis thaliana]

MARTKQTARKSTGGKAPRKQLATKAARKSAPATGGVKKPHRFRPGTVALREIRKYQKSTELLIRKLPFQRLVREIAQDFKTDLRFQSSAVAALQEAAEAYLVGLFEDTNLCAIHAKRVTIMPKDIQLARRIRGERA

>H3_S_purpuratus ref|NW_783816.1|SpuUn_WGA29134_1 Strongylocentrotus purpuratus chromosome Un genomic contig

MARTKQTARKSTGGKAPRKQLATKAARKSAPATGGVKKPHRYRPGTVALREIRRYQKSTELLIRKLPFQRLVREIAQDFKTELRFQSSAVMALQEASEAYLVGLFEDTNLCAIHAKRVTIMPKDIQLARRIRGERA

>H3_M_musculus ref|NP_038578.2| histone 1, H3a [Mus musculus]

MARTKQTARKSTGGKAPRKQLATKAARKSAPATGGVKKPHRYRPGTVALREIRRYQKSTELLIRKLPFQRLVREIAQDFKTDLRFQSSAVMALQEACEAYLVGLFEDTNLCAIHAKRVTIMPKDIQLARRIRGERA

>H2A_H_sapiens gi|10645195|ref|NP_066390.1| H2A histone family, member A; histone H2AE [Homo sapiens]

MSGRGKQGGKARAKAKTRSSRAGLQFPVGRVHRLLRKGNYSERVGAGAPVYLAAVLEYLTAEILELAGNAARDNKKTRIIPRHLQLAIRNDEELNKLLGRVTIAQGGVLPNIQAVLLPKKTESHHKAKGK

>H2A_S_cerevisiae gi|6320431|ref|NP_010511.1| Histone H2A (HTA1 and HTA2 code for nearly identical proteins); Hta1p [Saccharomyces cerevisiae]

MSGGKGGKAGSAAKASQSRSAKAGLTFPVGRVHRLLRRGNYAQRIGSGAPVYLTAVLEYLAAEILELAGNAARDNKKTRIIPRHLQLAIRNDDELNKLLGNVTIAQGGVLPNIHQNLLPKKSAKATKASQEL

>H2A_C_elegans ref|NP_507032.1| HIStone family member (his-3) [Caenorhabditis elegans]

MSGRGK-GGKAKTGGKAKSRSSRAGLQFPVGRLHRILRKGNYAQRVGAGAPVYLAAVLEYLAAEVLELAGNAARDNKKTRIAPRHLQLAVRNDEELNKLLAGVTIAQGGVLPNIQAVLLPKKT

>H2A_S_paradoxus gb|AABY01000107.1| Saccharomyces paradoxus NRRL Y-17217 contig_117, whole genome shotgun

MSGGKGGKAGSAAKASQSRSAKAGLTFPVGRVHRLLRRGNYAQRIGSGAPVYLTAVLEYLAAEILELAGNAARDNKKTRIIPRHLQLAIRNDDELNKLLGNVTIAQGGVLPNIHQNLLPKKSAKATKASQEL

>H2A_S_mikatae gb|AACH01000044.1| Saccharomyces mikatae IFO 1815 YM4906-Contig2815, whole genome shotgun

MSGGKGGKAGSAAKASQSRSAKAGLTFPVGRVHRLLRRGNYAQRIGSGAPVYLTAVLEYLAAEILELAGNAARDNKKTRIIPRHLQLAIRNDDELNKLLGNVTIAQGGVLPNIHQNLLPKKSAKATKASQEL

>H2A_S_kudriavzevii gb|AACI01000731.1| Saccharomyces kudriavzevii IFO 1802 YM6553-Contig1379, whole genome

MSGGKGGKAGSAAKASQSRSAKAGLTFPVGRVHRLLRRGNYAQRIGSGAPVYLTAVLEYLAAEILELAGNAARDNKKTRIIPRHLQLAIRNDDELNKLLGNVTIAQGGVLPNIHQNLLPKKSAKATKASQEL

>H2A_S_bayanus gb|AACA01000411.1| Saccharomyces bayanus MCYC 623 contig_500, whole genome shotgun

MSGGKGGKAGSAAKASQSRSAKAGLTFPVGRVHRLLRRGNYAQRIGSGAPVYLTAVLEYLAAEILELAGNAARDNKKTRIIPRHLQLAIRNDDELNKLLGNVTIAQGGVLPNIHQNLLPKKSAKATKASQEL

>H2A_S_castelli gb|AACF01000001.1| Saccharomyces castellii NRRL Y-12630 YM476-Contig721, whole genome

MSGGKGGKAGSAAKASQSRSAKAGLTFPVGRVHRLLRRGNYAQRIGSGAPVYLTAVLEYLAAEILELAGNAARDNKKTRIIPRHLQLAIRNDDELNKLLGNVTIAQGGVLPNIHQNLLPKKSAKATKASQEL

>H2A_C_albicans gb|AACQ01000148.1| Candida albicans SC5314 chromosome 3 Ctg19-20259, whole genome

MSGGKGKAGTSEKASTSRSAKAGLTFPVGRVHRLLRKGNYAQRIGSGAPVYLTSVLEYLAAEILELAGNAARDNKKSRIIPRHLQLAIRNDEELNKLLGDVTIAQGGVLPNIHQNLLPKKSGKGGVKASQEL

>H2A_S_kluyveri gb|AACE01000740.1| Saccharomyces kluyveri NRRL Y-12651 YM479-Contig1763, whole genome

MSGGKGGKAGSAAKASQSRSAKAGLTFPVGRVHRLLRKGNYAQRIGSGAPVYLTAVLEYLAAEILELAGNAARDNKKTRIIPRHLQLAIRNDDELNKLLGNVTIAQGGVLPNIHQNLLPKKSAKATKASQEL

>H2A_K_waltii gb|AADM01000048.1| Kluyveromyces waltii NCYC 2644 cont47, whole genome shotgun sequence

MSGGKGGKAGSAAKASQSRSAKAGLTFPVGRVHRLLRRGNYAQRVGSGAPVYMTAVLEYLAAEILELAGNAARDNKKTRIIPRHLQLAIRNDDELNKLLGNVTIAQGGVLPNIHQNLLPKKSAKPGKASQEL

>H2A_A_gossypii gb|AE016818.1| Eremothecium gossypii ATCC 10895 chromosome V, complete sequence

MSGKGGKAGSAAKASQSRSAKAGLTFPVGRVHRLLRKGNYAQRIGSGAPVYLTAVLEYLAAEILELAGNAARDNKKTRIIPRHLQLAIRNDDELNKLLGNVTIAQGGVLPNIHANLLPKKSAKATKA

>H2A_D_ananassae scaffold_13030

MSGRGKGGKVKGKAKSRSNRAGLQFPVGRIHRLLRKGNYAERVGAGAPVYLAAVMEYLAAEVLELAGNAARDNKKTRIIPRHLQLAIRNDEELNKLLSGVTIAQGGVLPNIQAVLLPKKTE

>H2A_D_simulans chrX

MSGRGKGGKVKGKAKSRSNRAGLQFPVGRIHRLLRKGNYAERVGAGAPVYLAAVMEYLAAEVLELAGNAARDNKKTRIIPRHLQLAIRNDEELNKLLSGVTIAQGGVLPNIQAVLLPKKTE

>H2A_D_melanogaster chr2L

MSGRGKGGKVKGKAKSRSNRAGLQFPVGRIHRLLRKGNYAERVGAGAPVYLAAVMEYLAAEVLELAGNAARDNKKTRIIPRHLQLAIRNDEELNKLLSGVTIAQGGVLPNIQAVLLPKKTE

>H2A_D_virilis scaffold_13196

MSGRGKGGKVKGKAKSRSNRAGLQFPVGRIHRLLRKGNYAERVGAGAPVYLAAVMEYLAAEVLELAGNAARDNKKTRIIPRHLQLAIRNDEELNKLLSGVTIAQGGVLPNIQAVLLPKKTE

>H2A_D_yakuba chrU

MSGRGKGGKVKGKAKSRSNRAGLQFPVGRIHRLLRKGNYAERVGAGAPVYLAAVMEYLAAEVLELAGNAARDNKKTRIIPRHLQLAIRNDEELNKLLSGVTIAQGGVLPNIQAVLLPKKTE

>H2A_D_pseudoobscura gi|54637696|gb|EAL27098.1| GA18930-PA [Drosophila pseudoobscura]

QAGGKAGKDSGKAKAKAVSRSARAGLQFPVGRIHRHLKSRTTSHGRVGATAAVYSAAILEYLTAEVLELAGNASKDLKVKRITPRHLQLAIRGDEELDSLIKATIAGGGVIPHIHKSLIGKKEDTVQDPQRKGNVILSQAY

>H2A_D_mojavensis scaffold_6583

MSGRGKGGKVKGKAKSRSNRAGLQFPVGRIHRLLRKGNYAERVGAGAPVYLAAVMEYLAAEVLELAGNAARDNKKTRIIPRHLQLAIRNDEELNKLLSGVTIAQGGVLPNIQAVLLPKKTE

>H2A_A_nidulans (AN3468.3) histone H2A (translation)

MTGGKSGGKASGSKNAQSRSSKAGLAFPVGRVHRLLRKGNYAQRVGAGAPVYLAAVLEYLAAEILELAGNAARDNKKTRIIPRHLQLAIRNDEELNKLLGHVTIAQGGVLPNIHQNLLPKKTPKAGKGSQEL

>H2A_S_pombe ref|NC_003424.1| Schizosaccharomyces pombe chromosome I, complete sequence

MSGGKSGGKAAVAKSAQSRSAKAGLAFPVGRVHRLLRKGNYAQRVGAGAPVYLAAVLEYLAAEILELAGNAARDNKKTRIIPRHLQLAIRNDEELNKLLGHVTIAQGGVVPNINAHLLPKTSGRTGKPSQEL

>H2A_A_thaliana ref|NP_175517.1| DNA binding [Arabidopsis thaliana]

MAGRGKTLGSGSAKKATTRSSKAGLQFPVGRIARFLKKGKYAERVGAGAPVYLAAVLEYLAAEVLELAGNAARDNKKTRIVPRHIQLAVRNDEELSKLLGDVTIANGGVMPNIHNLLLPKKT

>H2A_S_purpuratus ref|NW_678601.1|SpuUn_WGA103292_1 Strongylocentrotus purpuratus chromosome Un genomic contig

MSGRGKGGGKARAKAKSRSARAGLQFPVGRVHRFLRKGNYAARVGAGAPVYLAAVLEYLAAEILELAGNAARDNKKTRIIPRHLQLAVRNDEELNKLLSGVTIAQGGVLPNIQAVLLPKKTSKASK

>H2A_M_musculus ref|NP_835492.1| histone 1, H2ao [Mus musculus]

MSGRGKQGGKARAKAKTRSSRAGLQFPVGRVHRLLRKGNYSERVGAGAPVYLAAVLEYLTAEILELAGNAARDNKKTRIIPRHLQLAIRNDEELNKLLGRVTIAQGGVLPNIQAVLLPKKTESHHKAKGK

>H2B_H_sapiens gi|4504257|ref|NP_003509.1| H2B histone family, member A [Homo sapiens]

MPEPAKSAPAPKKGSKKAVTKAQKKDGKKRKRSRKESYSVYVYKVLKQVHPDTGISSKAMGIMNSFVNDIFERIAGEASRLAHYNKRSTITSREIQTAVRLLLPGELAKHAVSEGTKAVTKYTSSK

>H2B_S_cerevisiae gi|6320430|ref|NP_010510.1| Histone H2B (HTB1 and HTB2 code for nearly identical proteins); Htb1p [Saccharomyces cerevisiae]

MSAKAEKKPASKAPAEKKPAAKKTSTSTDGKKRSKARKETYSSYIYKVLKQTHPDTGISQKSMSILNSFVNDIFERIATEASKLAAYNKKSTISAREIQTAVRLILPGELAKHAVSEGTRAVTKYSSSTQA

>H2B_C_elegans ref|NP_505464.1| HIStone family member (his-41) [Caenorhabditis elegans]

APPKPSAKGAKKAAKTVSKPKDGKKRKHARKESYSVYIYRVLKQVHPDTGVSSKAMSIMNSFVNDVFERIASEASRLAHYNKRSTISSREIQTAVRLILPGELAKHAVSEGTKAVTKYTSSK

>H2B_S_paradoxus gb|AABY01000107.1| Saccharomyces paradoxus NRRL Y-17217 contig_117, whole genome shotgun

MSAKAEKKPASKAPAEKKPAAKKTSTSTDGKKRSKARKETYSSYIYKVLKQTHPDTGISQKSMSILNSFVNDIFERIATEASKLAAYNKKSTISAREIQTAVRLILPGELAKHAVSEGTRAVTKYSSSTQA

>H2B_S_mikatae gb|AABZ01000661.1| Saccharomyces mikatae IFO 1815 contig_225, whole genome shotgun

MSAKAEKKPASKAPAEKKPAAKKTSTSTDGKKRSKARKETYSSYIYKVLKQTHPDTGISQKSMSILNSFVNDIFERIATEASKLAAYNKKSTISAREIQTAVRLILPGELAKHAVSEGTRAVTKYSSSTQA

>H2B_S_kudriavzevii gb|AACI01000731.1| Saccharomyces kudriavzevii IFO 1802 YM6553-Contig1379, whole genome

MSAKAEKKPASKAPAEKKPAAKKTSTSTDGKKRSKARKETYSSYIYKVLKQTHPDTGISQKSMSILNSFVNDIFERIATEASKLAAYNKKSTISAREIQTAVRLILPGELAKHAVSEGTRAVTKYSSSTQA

>H2B_S_bayanus gb|AACA01000323.1| Saccharomyces bayanus MCYC 623 contig_499, whole genome shotgun

MSAKAEKKPASKAPAEKKPAAKKTSTSTDGKKRTKARKETYSSYIYKVLKQTHPDTGISQKSMSILNSFVNDIFERIATEASKLAAYNKKSTISAREIQTAVRLILPGELAKHAVSEGTRAVTKYSSSTQA

>H2B_S_castellii gb|AACF01000001.1| Saccharomyces castellii NRRL Y-12630 YM476-Contig721, whole genome

MSAVAEKKPASKAPAEKKPVAKKTSTGGDIKKRTKARKETYSSYIYKVLKQTHPDTGISQKSMSILNSFVNDIFERIATEASKLAAYNKKSTISAREIQTAVRLILPGELAKHAVSEGTRAVTKYSSSTQA

>H2B_C_albicans gb|AACQ01000148.1| Candida albicans SC5314 chromosome 3 Ctg19-20259, whole genome

MAPKAEKKPASKAPAEKKPAAKKTASTDGAKKRTKARKETYSSYIYKVLKQTHPDTGISQKAMSIMNSFVNDIFERIATEASKLAAYNKKSTISAREIQTAVRLILPGELAKHAVSEGTRAVTKYSSAS

>H2B_S_kluyveri gb|AACE01000740.1| Saccharomyces kluyveri NRRL Y-12651 YM479-Contig1763, whole genome

MAPKAEKKPASKAPAEKKPAAKKTASVDSTKKRTKARKETYSSYIYKVLKQTHPDTGISQKSMSILNSFVNDIFERIASEASKLAAYNKKSTISAREIQTAVRLILPGELAKHAVSEGTRAVTKYSSSTQA

>H2B_K_waltii gb|AADM01000056.1| Kluyveromyces waltii NCYC 2644 cont55, whole genome shotgun sequence

MSAKAEKKPASKAPAEKKPAAKKTASSIDSNKRRTKVRKETYSSYIYKVLKQTHPDTGISQKSMSILNSFVNDIFERIASEASKLAAYNKKSTISAREIQTAVRLILPGELAKHAVSEGTRAVTKYSSSTQA

>H2B_A_gossypii gb|AE016820.2| Eremothecium gossypii ATCC 10895 chromosome VII, complete sequence

MSSKASKAPASKAPAEKKPAAKKTSSSVDASKKRTKTRKETYSSYIYKVLKQTHPDTGISQKSMSILNSFVNDIFERIASEASKLAAYNKKSTISAREIQTAVRLILPGELAKHAVSEGTRAVTKYSSSTQA

>H2B_D_ananassae scaffold_12943

VNLKVTMPPKTSGKAAKKAGKAQKNITKNDKKKKRKRKESYAIYIYKVLKQVHPDTGISSKAMSIMNSFVNDIFERIAAEASRLAHYNKRSTITSREIQTAVRLLLPGELAKHAVSEGTKAVTKYTSS

>H2B_D_simulans chrU

PPKTSGKAAKKAGKAQKNITKTDKKKKRKRKESYAIYIYKVLKQVHPDTGISSKAMSIMNSFVNDIFERIAAEASRLAHYNKRSTITSREIQTAVRLLLPGELAKHAVSEGTKAVTKYTSSK

>H2B_D_melanogaster chr2L

PPKTSGKAAKKAGKAQKNITKTDKKKKRKRKESYAIYIYKVLKQVHPDTGISSKAMSIMNSFVNDIFERIAAEASRLAHYNKRSTITSREIQTAVRLLLPGELAKHAVSEGTKAVTKYTSSK

>H2B_D_virilis scaffold_13047

PPKTSGKAAKKAGKAQKNITKNDKKKKRKRKESYAIYIYKVLKQVHPDTGISSKAMSIMNSFVNDIFERIAAEASRLAHYNKRSTITSREIQTAVRLLLPGELAKHAVSEGTKAVTKYTSSK

>H2B_D_yakuba chrU

PPKTSGKAAKKAGKAQKKKKRQRKESYAIYIYKVLKQVHPDTGISSKAMSIMNSFVNDIFERIAAEASRLAHYNKRSTITSREIQTAVRLLLPGELAKHAVSEGTKAVTKYTSSK

>H2B_D_pseudoobscura chr2

PPKTSGKAAKKAGKAQKNITKNDKKKKRKRKESYAIYIYKVLKQVHPDTGISSKAMSIMNSFVNDIFERIAAEASRLAHYNKRSTITSREIQTAVRLLLPGELAKHAVSEGTKAVTKYTSSK

>H2B_D_mojavensis scaffold_6583

PPKTSGKAAKKAGKAQKNITKNDKKKKRKRKESYAIYIYKVLKQVHPDTGISSKAMSIMNSFVNDIFERIAAEASRLAHYNKRSTITSREIQTAVRLLLPGELAKHAVSEGTKAVTKYTSSK

>H2B_A_nidulans (AN3469.3) histone H2B.1 (translation)

PAGKAPAEKKEAGKKTAAAASGEKKKRGKTRKETYSSYIYKVLKQVHPDTGISTRAMSILNSFVNDIFERVATEASKLAAYNKKSTISSREIQTSVRLILPGELAKHAVSEGTKAVTKYSSS

>H2B_S_pombe ref|NC_003421.1| Schizosaccharomyces pombe chromosome III, complete sequence

AEKKPASKAPAGKAPRDTMKSADKKRGKNRKETYSSYIYKVLKQVHPDTGISNQAMRILNSFVNDIFERIATEASKLAAYNKKSTISSREIQTAVRLILPGELAKHAVTEGTKSVTKYSSS

>H2B_A_thaliana ref|NP_197679.1| DNA binding [Arabidopsis thaliana]

EPAAAAEKKPKAGKKLPKEPAGAGDKKKKRSKKNVETYKIYIFKVLKQVHPDIGISSKAMGIMNSFINDIFEKLAGESSKLARYNKKPTITSREIQTAVRLVLPGELAKHAVSEGTKAVTKFTSS

>H2B_S_purpuratus ref|NW_832924.1|SpuUn_WGA73331_1 Strongylocentrotus purpuratus chromosome Un genomic contig

PAKAQPAGKKGSKKAKAPRPSGGKKRRRRRKESYGIYIYKVLKQVHPDTGISSRAMSIMNSFVNDVFERIAAEASRLAHYNKKSTITSREVQTAVRLLLPGELAKHAVSEGTKAVTKYTTSK

>H2B_M_musculus ref|NP_835503.1| histone 1, H2bg [Mus musculus]

MPEPAKSAPAPKKGSKKAVTKAQKKDGKKRKRSRKESYSVYVYKVLKQVHPDTGISSKAMGIMNSFVNDIFERIAGEASRLAHYNKRSTITSREIQTAVRLLLPGELAKHAVSEGTKAVTKYTSSK
