# Supplementary material for: Multiple independent evolutionary solutions to core histone gene regulation
Source: Genome Biol. 2006 Dec 21;7(12):R122. doi: 10.1186/gb-2006-7-12-r122 (PMC1794435; doi:10.1186/gb-2006-7-12-r122)
Supplement: Additional data file 3 — List of species used in the study [file gb-2006-7-12-r122-S3.doc]

Supplementary Table 1.

| **Species used in this study** |
| --- |
| *Saccharomyces paradoxus* |
| *Saccharomyces mikatae* |
| *Saccharomyces cerevisiae* |
| *Saccharomyces kudriavzevii* |
| *Saccharomyces bayanus* |
| *Saccharomyces castellii* |
| *Candida albicans* |
| *Saccharomyces kluyveri* |
| *Kluyveromyces waltii* |
| *Ashbya gossypii* |
| *Caenorhabditis elegans* |
| *Drosophila ananassae* |
| *Drosophila simulans* |
| *Drosophila melanogaster* |
| *Drosophila virilis* |
| *Drosophila yakuba* |
| *Drosophila pseudoobscura* |
| *Drosophila mojavensis* |
| *Aspergillus nidulans* |
| *Schizosaccharomyces pombe* |
| *Arabidopsis thaliana* |
| *Strongylocentrotus purpuratus* |
| *Homo sapiens* |
| *Mus musculus* |
